# Supplementary material for: Nickel-catalyzed synthesis of 1,1-diborylalkanes from terminal alkenes
Source: Nat Commun. 2017 Aug 24;8:345. doi: 10.1038/s41467-017-00363-4 (PMC5571201; doi:10.1038/s41467-017-00363-4)
Supplement: Supplementary file 1 — Supplementary Information [file 41467_2017_363_MOESM1_ESM.pdf]

File Name: Supplementary Information
Description: Supplementary Figures, Supplementary Tables, Supplementary Discussion and Supplementary Methods

File Name: Peer Review File
Description:

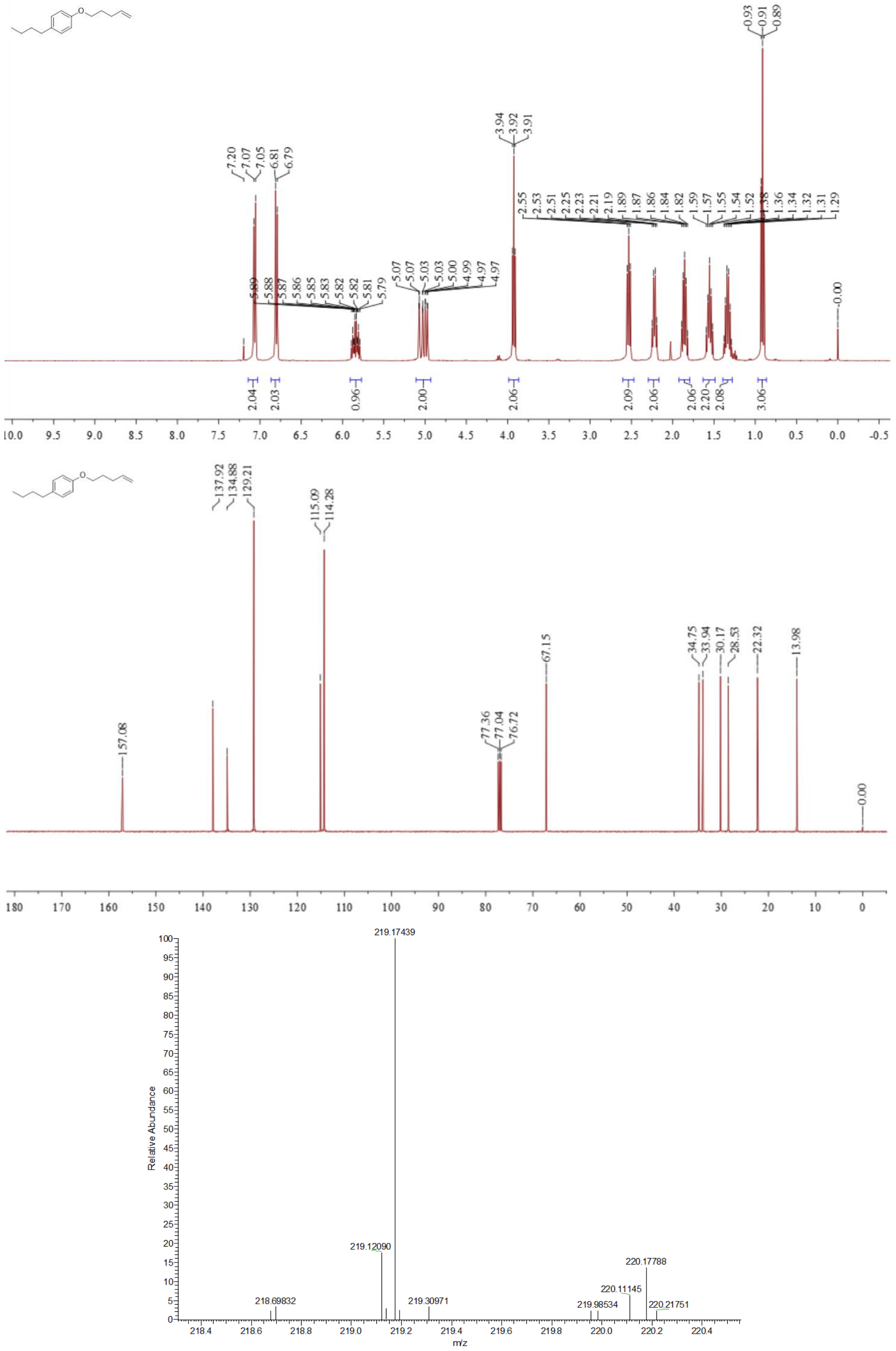

Supplementary Figure 1. ^{1}H, ^{13}C NMR and HRMS of butyl-4-(pent-4-en-1-yloxy)benzene

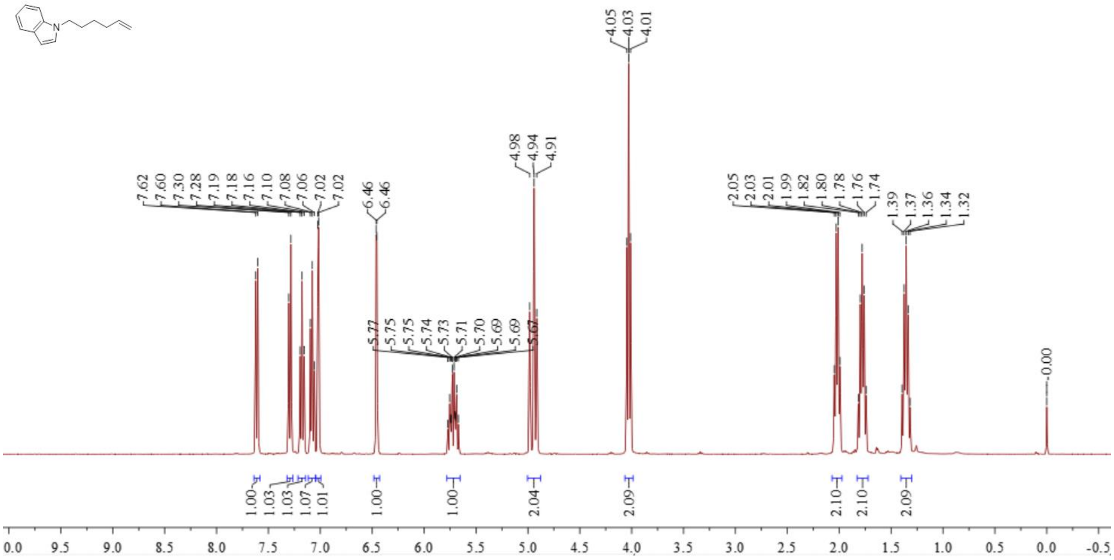

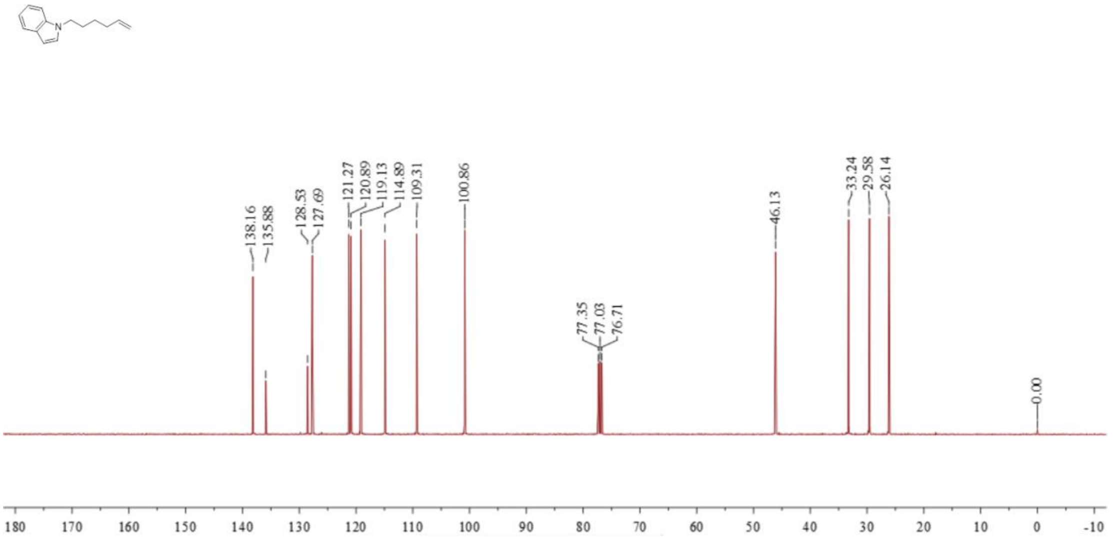

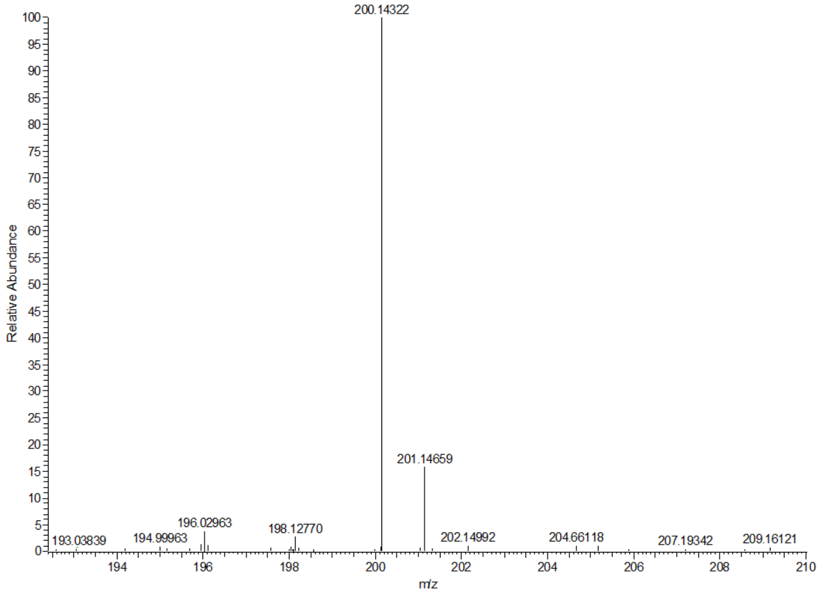

Supplementary Figure 2. ^{1}H, ^{13}C NMR and HRMS of 1-(hex-5-en-1-yl)-1*H*-indole

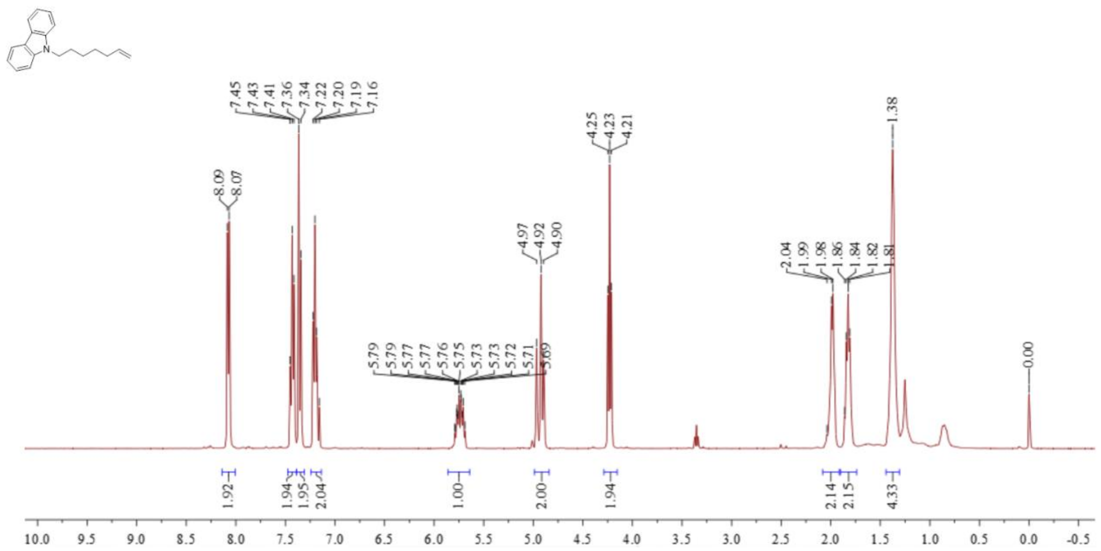

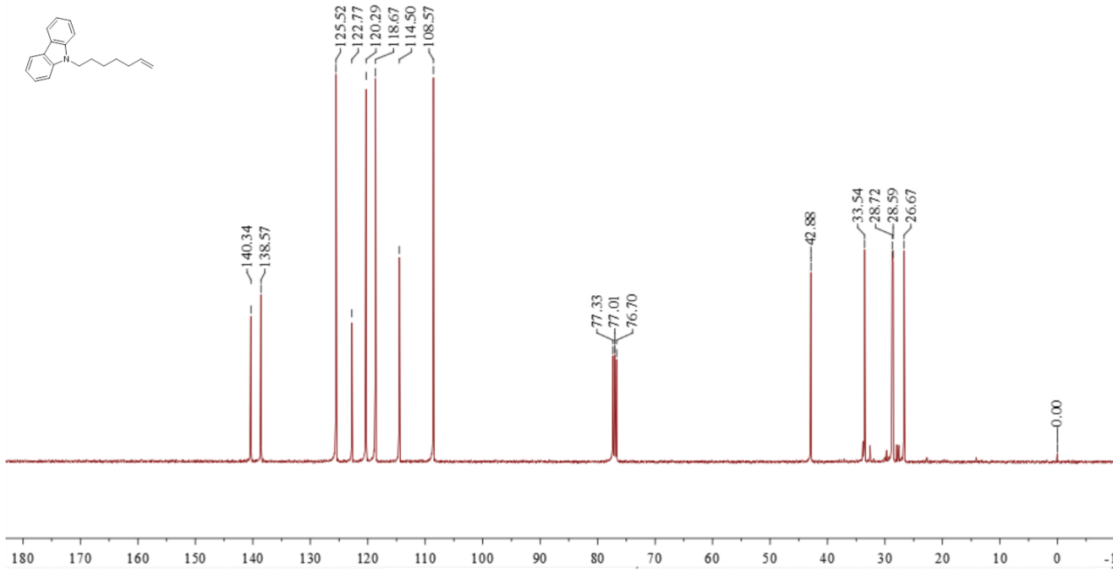

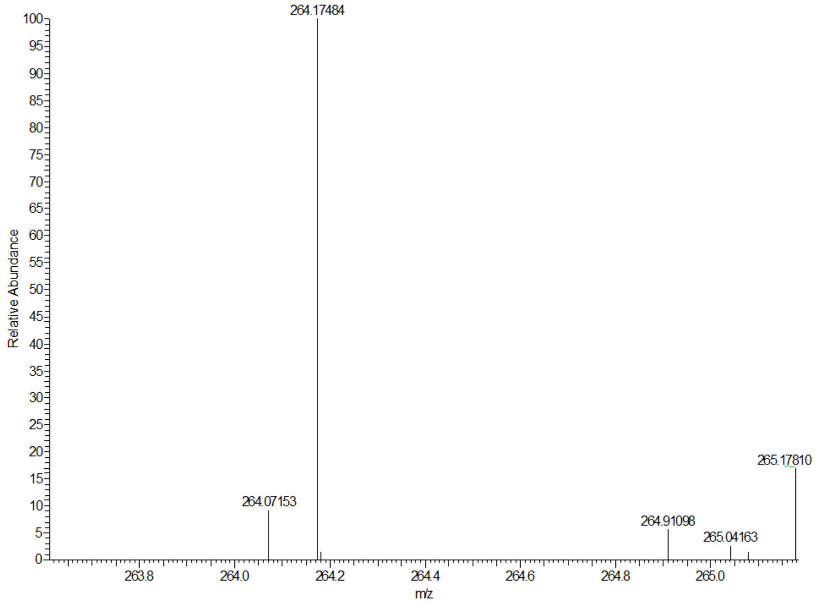

Supplementary Figure 3. ^{1}H, ^{13}C NMR and HRMS of 9-(hept-6-en-1-yl)-9*H*-carbazole

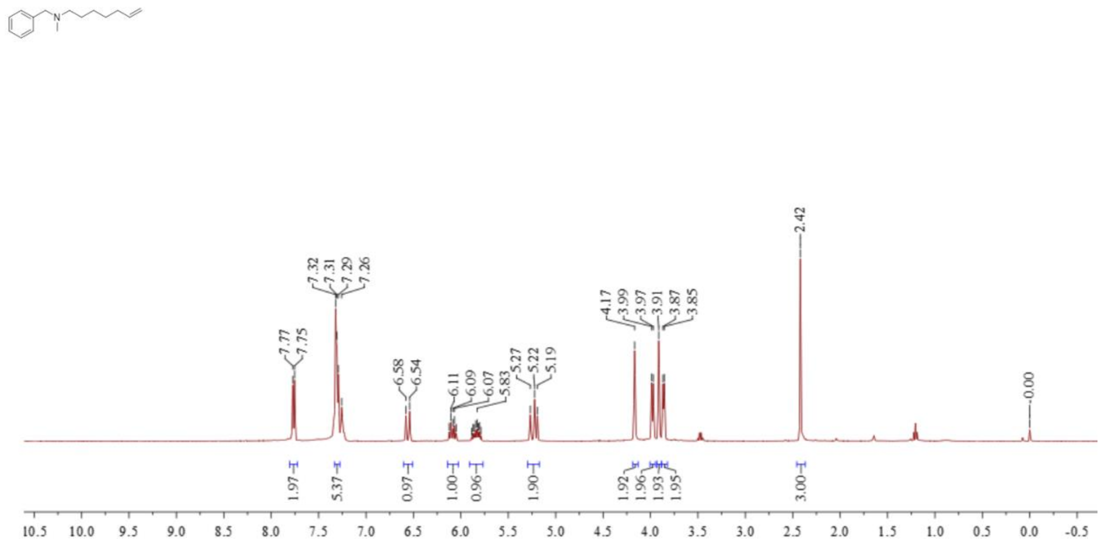

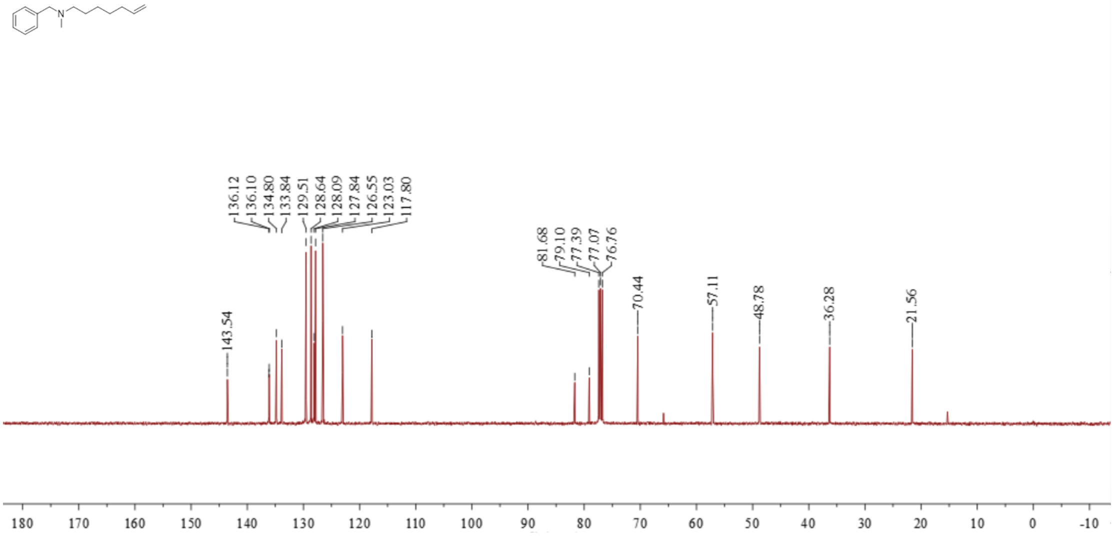

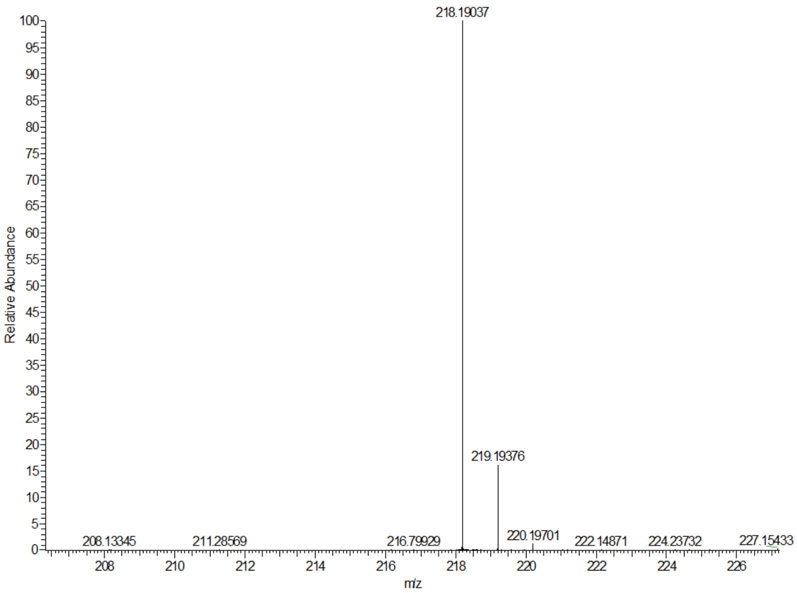

Supplementary Figure 4. ^{1}H, ^{13}C NMR and HRMS of *N*-benzyl-*N*-methylhept-6-en-1-amine

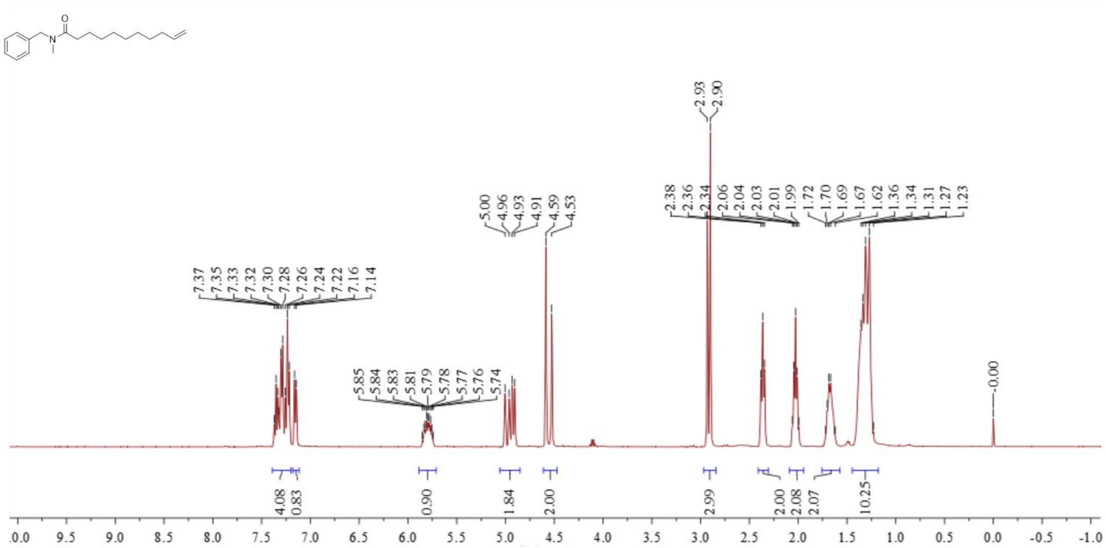

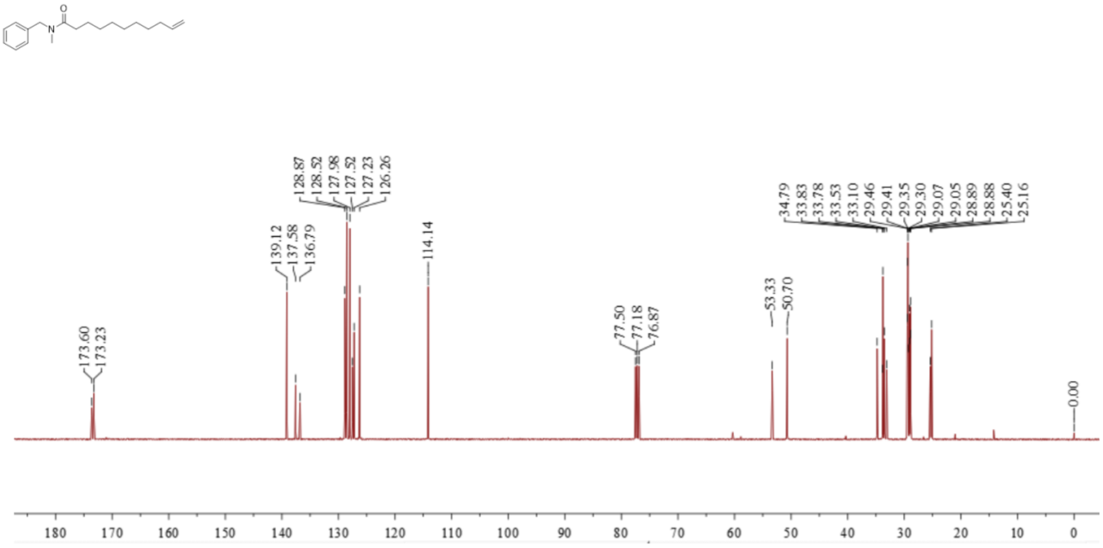

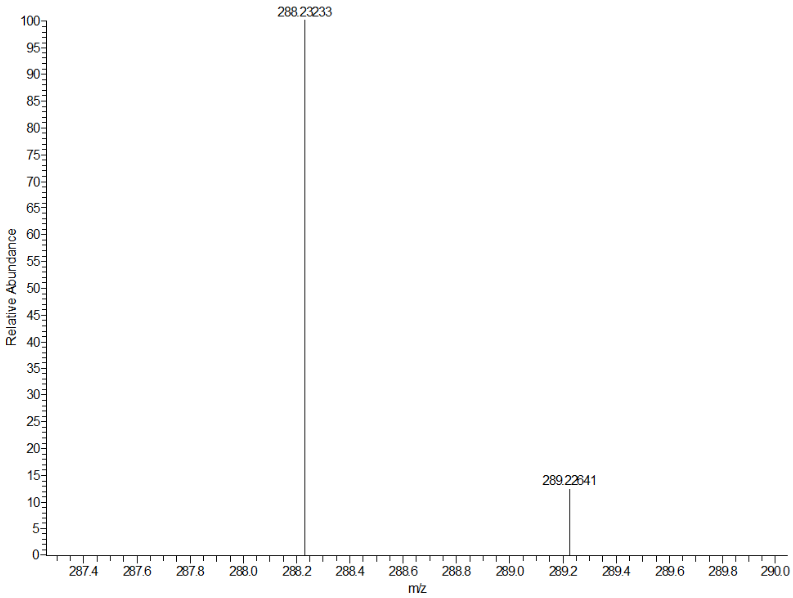

Supplementary Figure 5. ^{1}H, ^{13}C NMR and HRMS of *N*-benzyl-*N*-methylundec-10-enamide

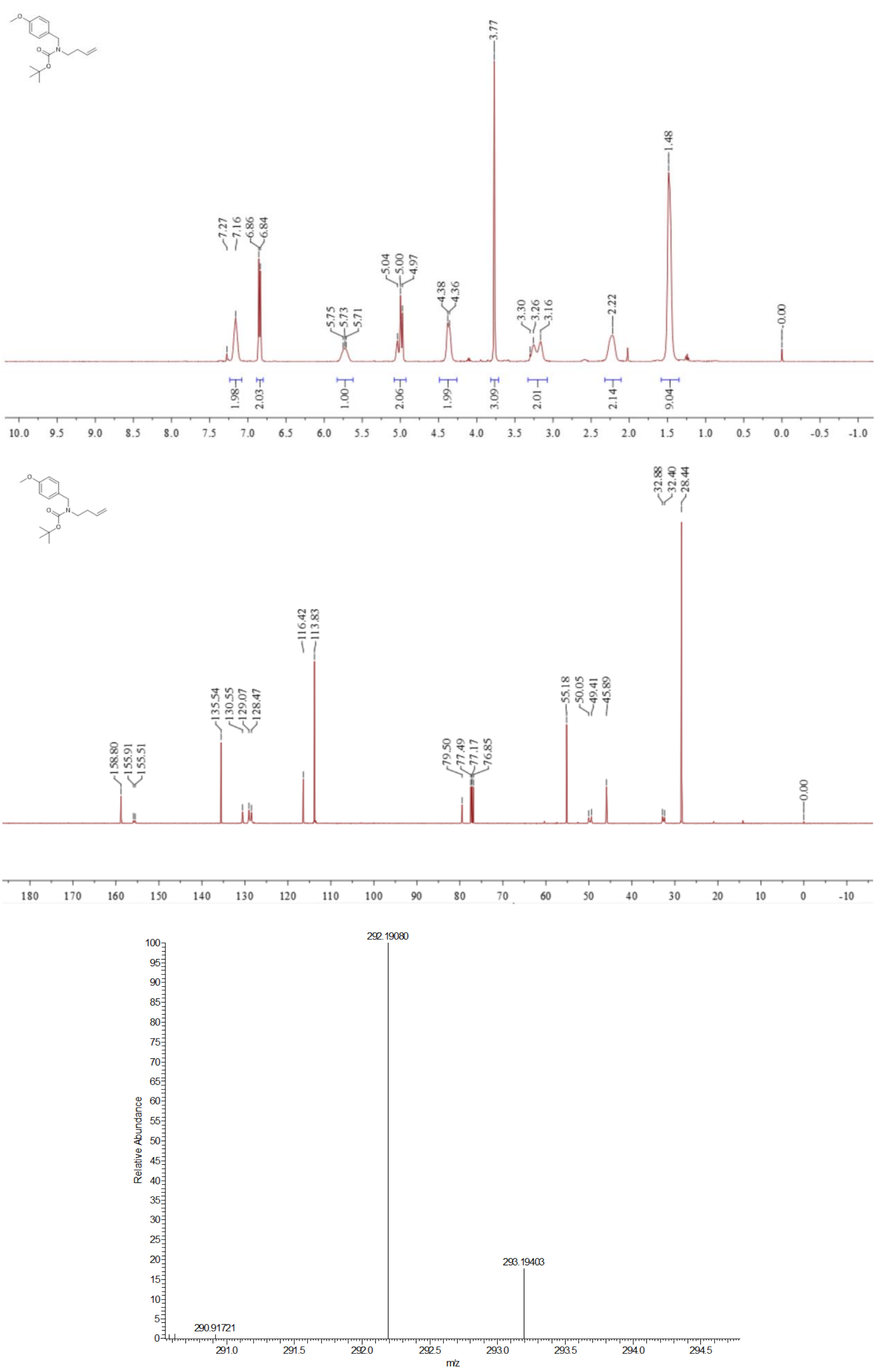

Supplementary Figure 6. ^{1}**H,** ^{13}**C NMR and HRMS of** ***tert*****-butyl but-3-en-1-yl(4-methoxybenzyl)carbamate**

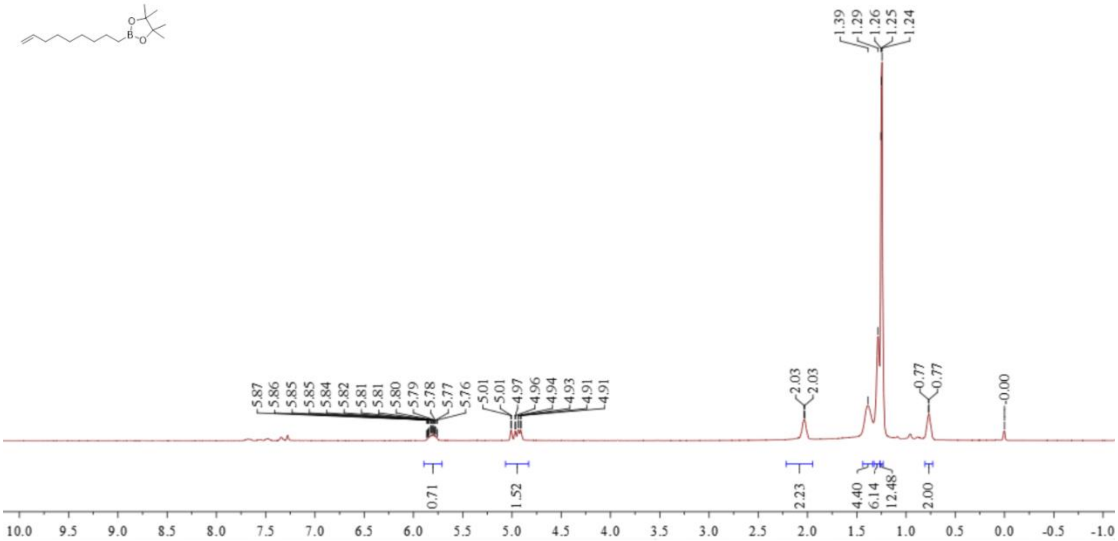

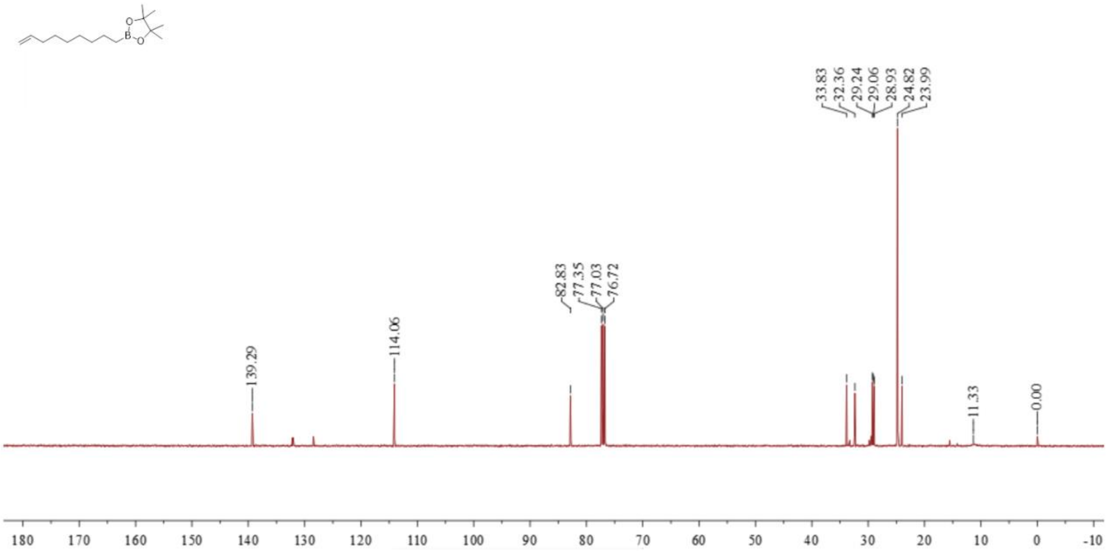

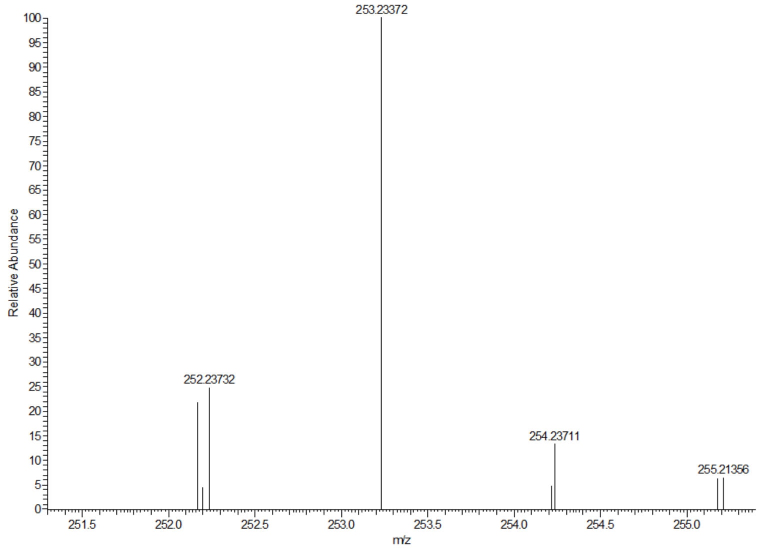

Supplementary Figure 7. ^{1}H, ^{13}C NMR and HRMS of 4,4,5,5-tetramethyl-2-(non-8-en-1-yl)-1,3,2-dioxaborolane

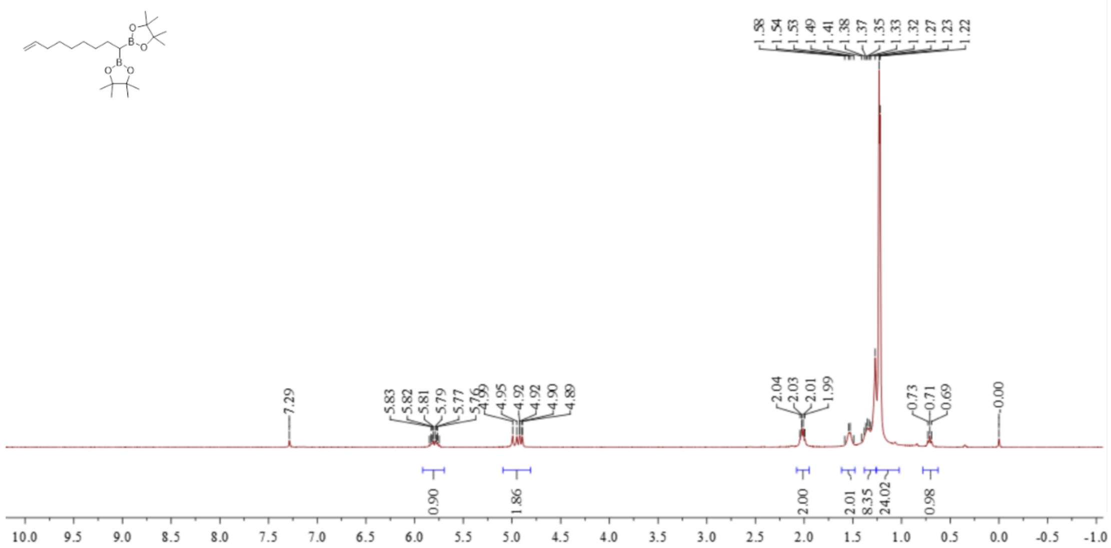

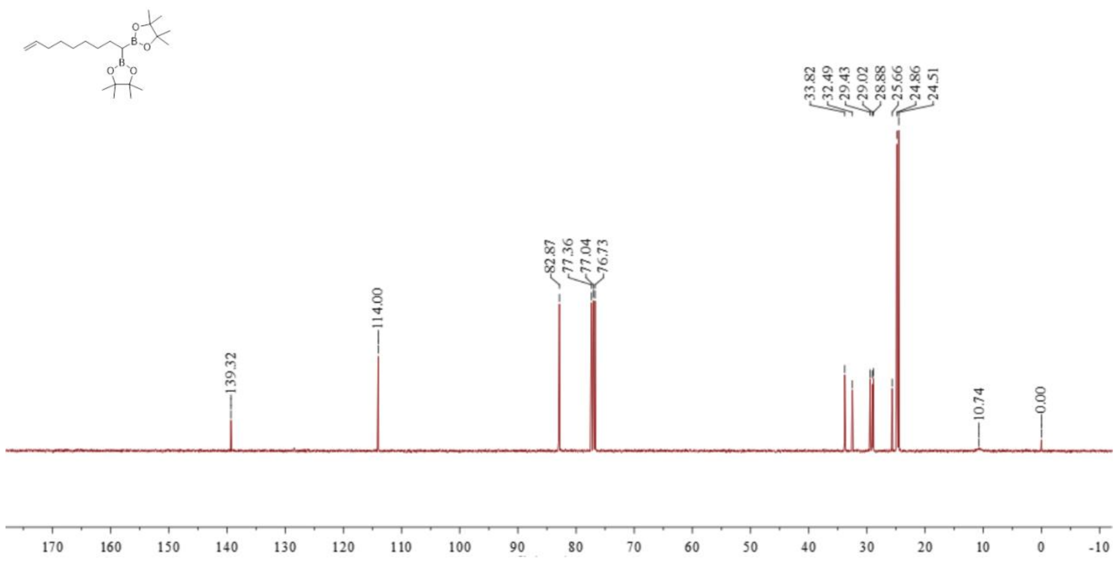

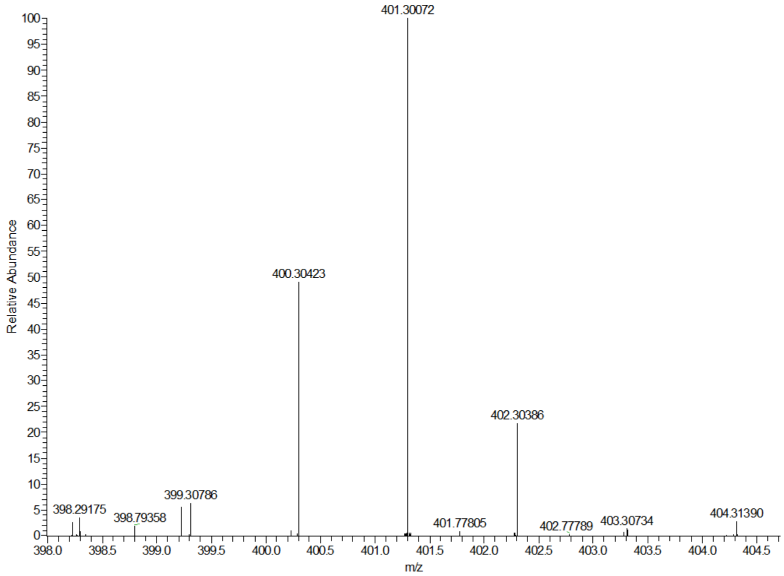

Supplementary Figure 8. ^{1}H, ^{13}C NMR and HRMS of 2,2'-(non-8-ene-1,1-diyl)bis(4,4,5,5-tetramethyl-1,3,2-dioxaborolane)

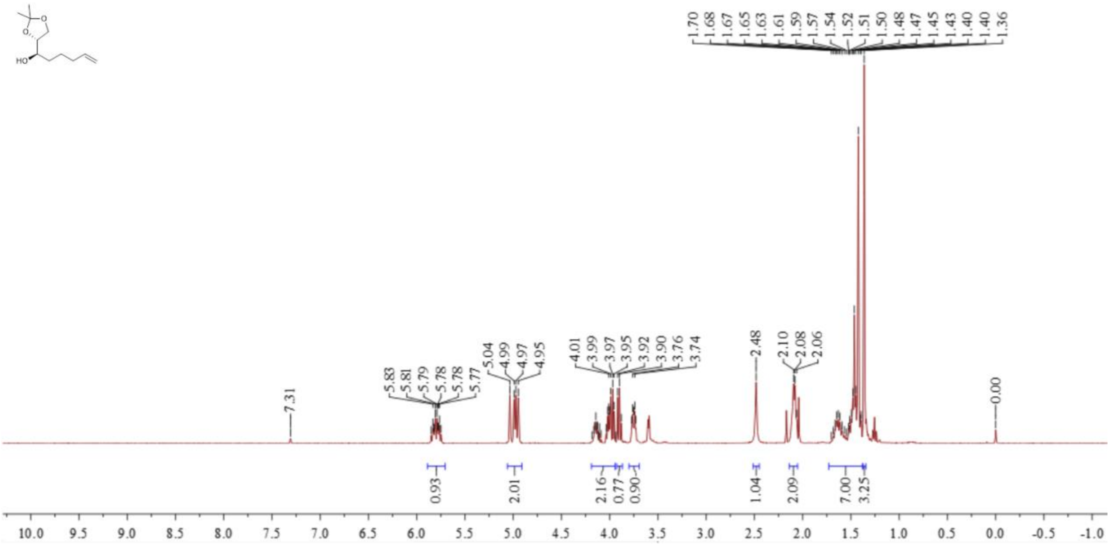

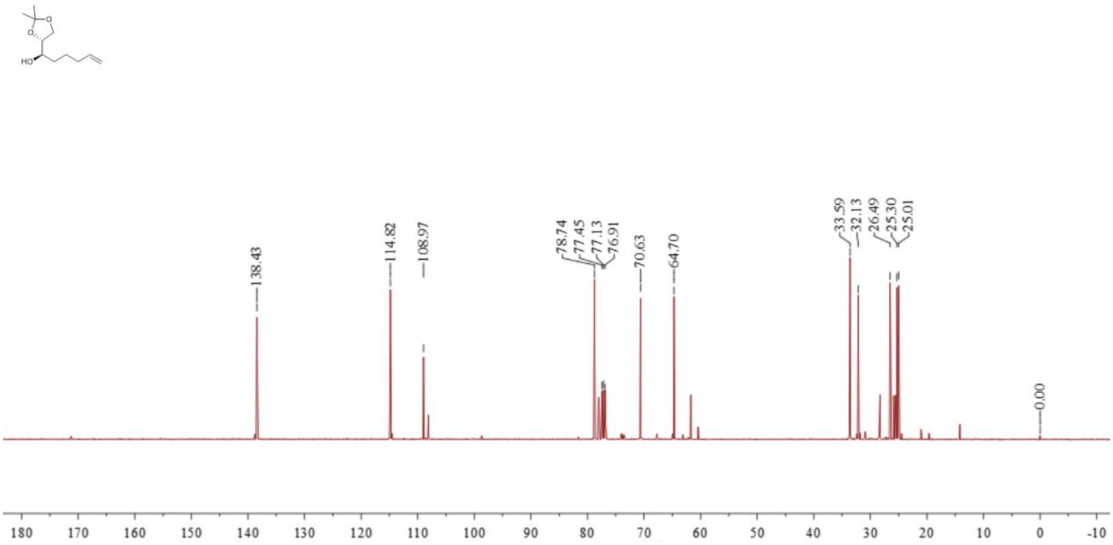

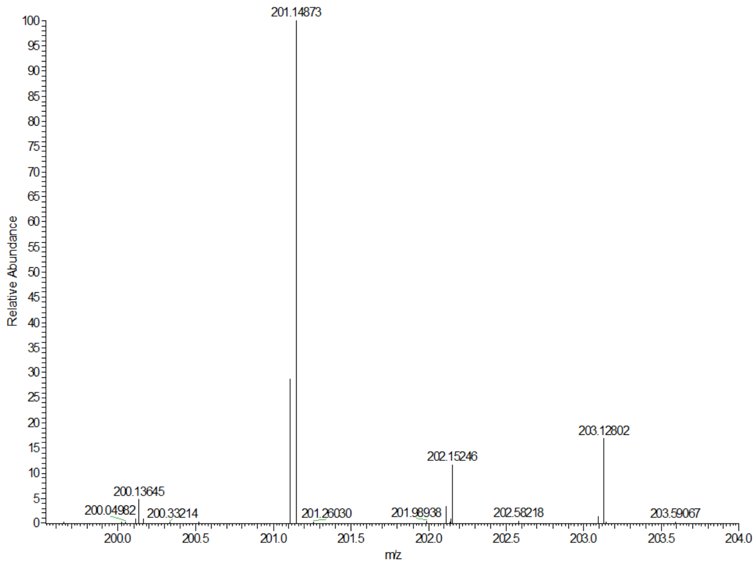

Supplementary Figure 9. ^{1}H, ^{13}C NMR and HRMS of (*R*)-1-((*R*)-2,2-dimethyl-1,3-dioxolan-4-yl)hex-5-en-1-ol

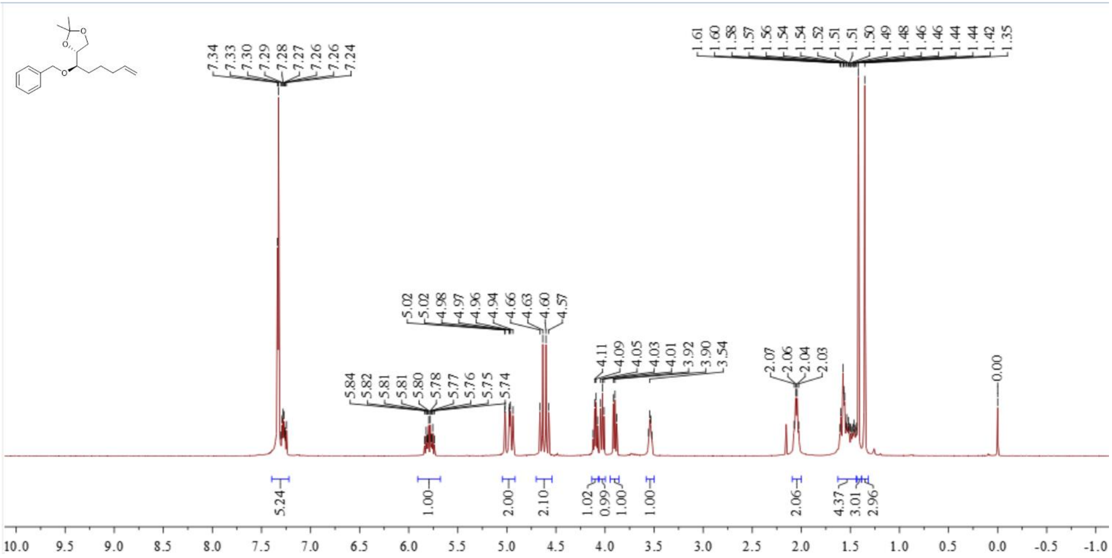

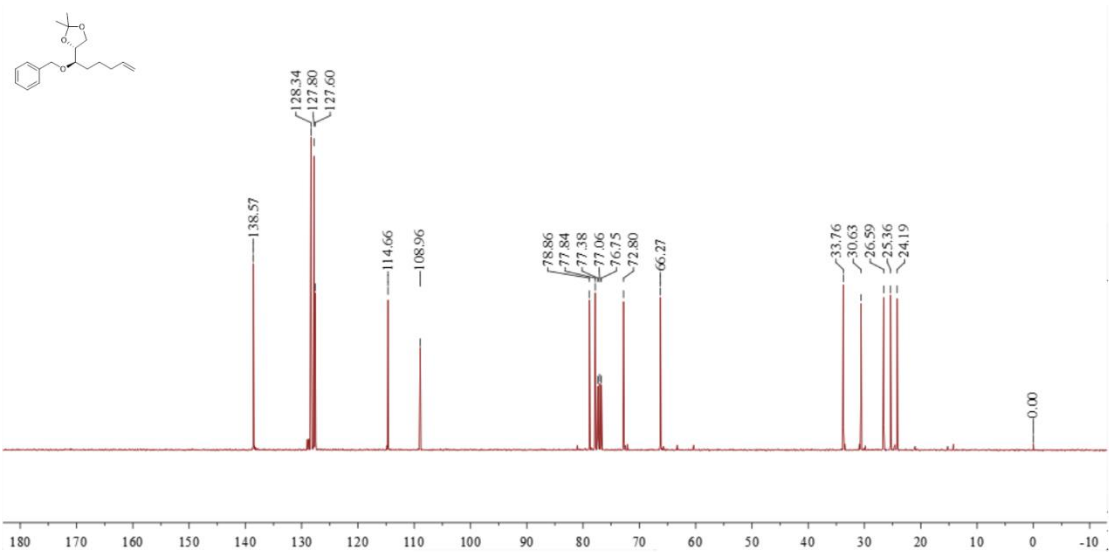

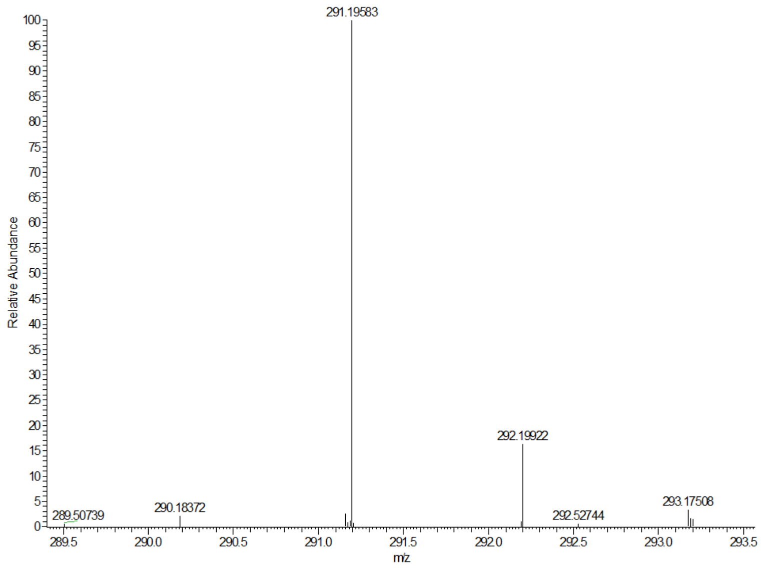

Supplementary Figure 10. ^{1}H, ^{13}C NMR and HRMS of (*R*)-4-((*R*)-1-(benzyloxy)hex-5-en-1-yl)-2,2-dimethyl-1,3-dioxolane

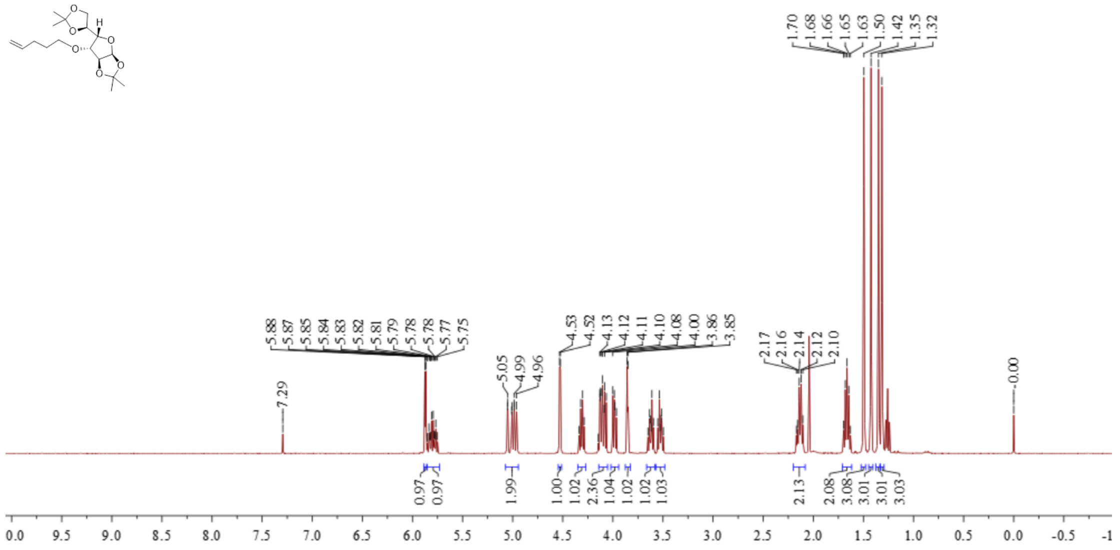

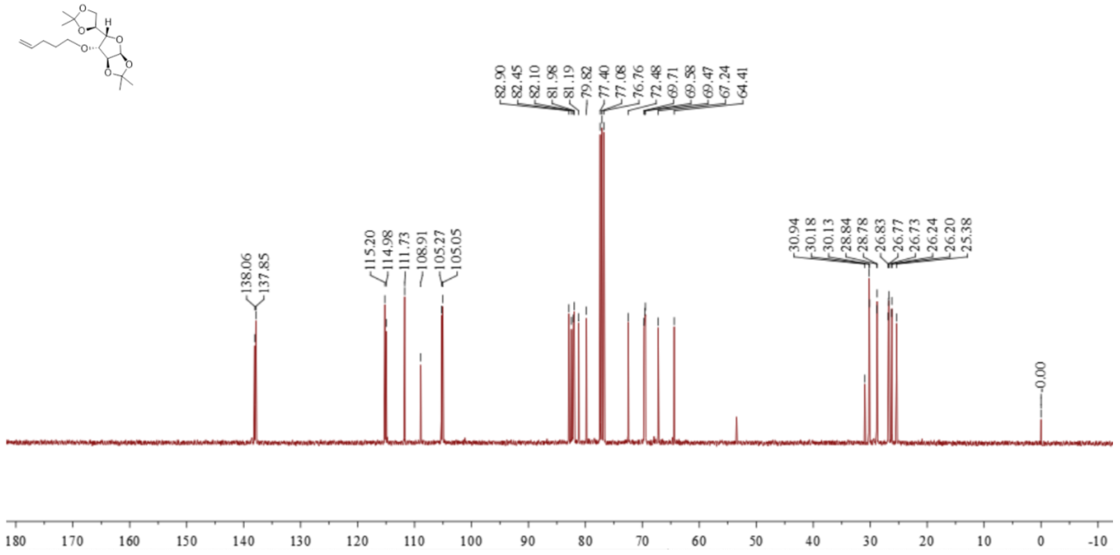

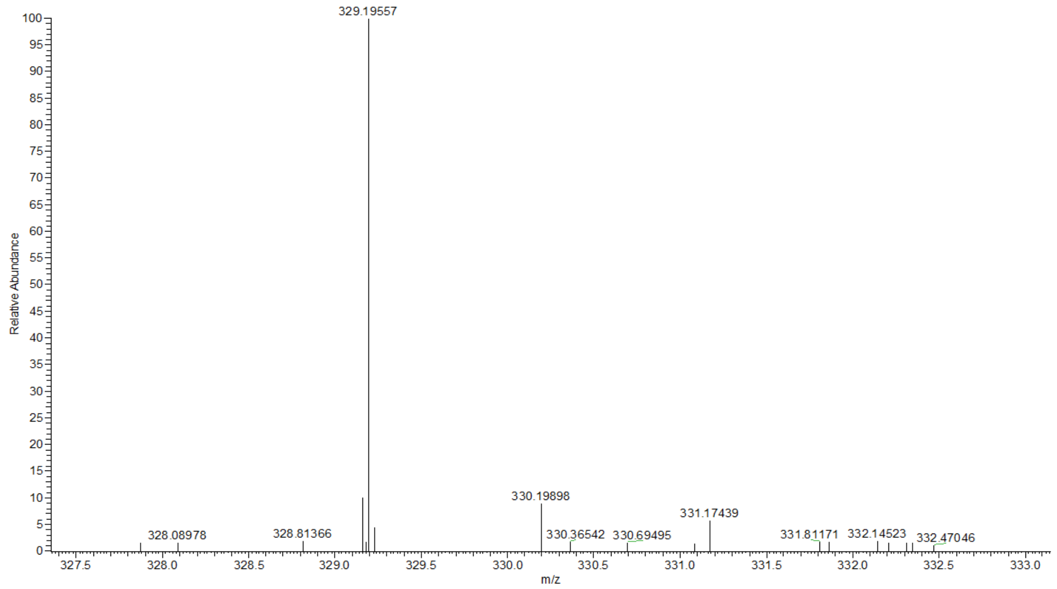

Supplementary Figure 11. ^{1}H, ^{13}C NMR and HRMS of compound 38

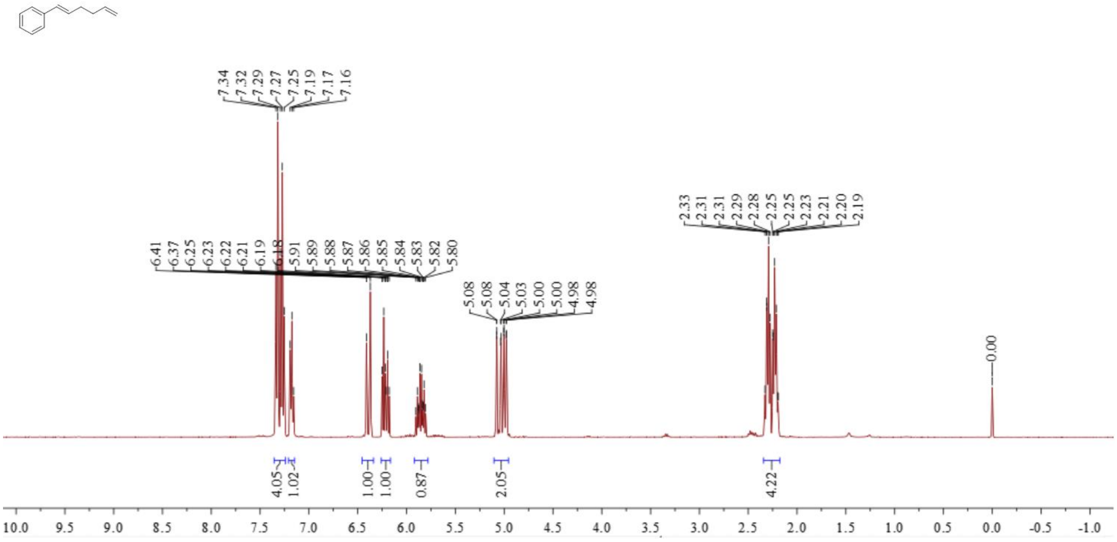

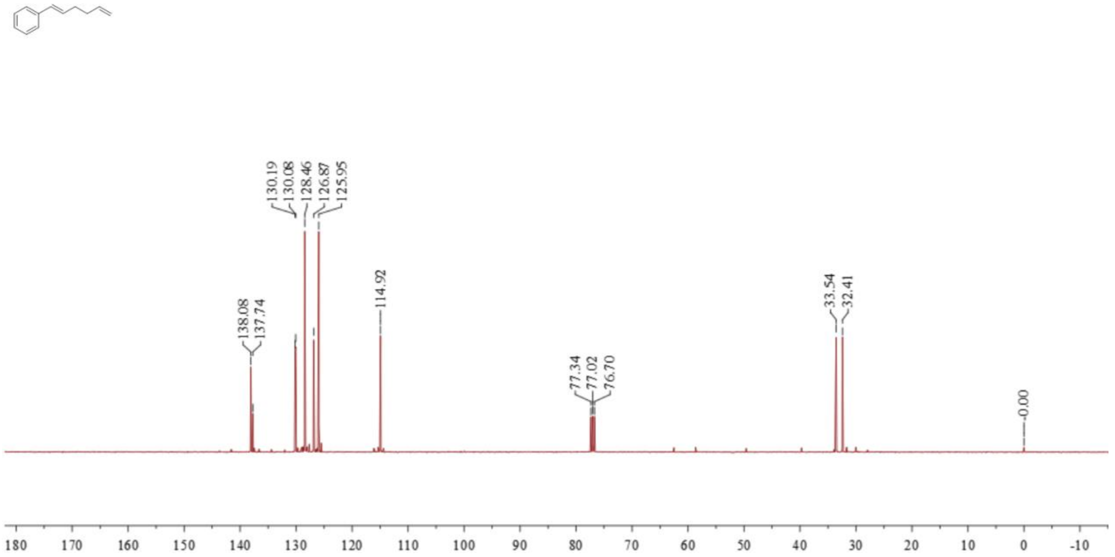

Supplementary Figure 12. ^{1}H and ^{13}C NMR of (*E*)-hexa-1,5-dien-1-ylbenzene

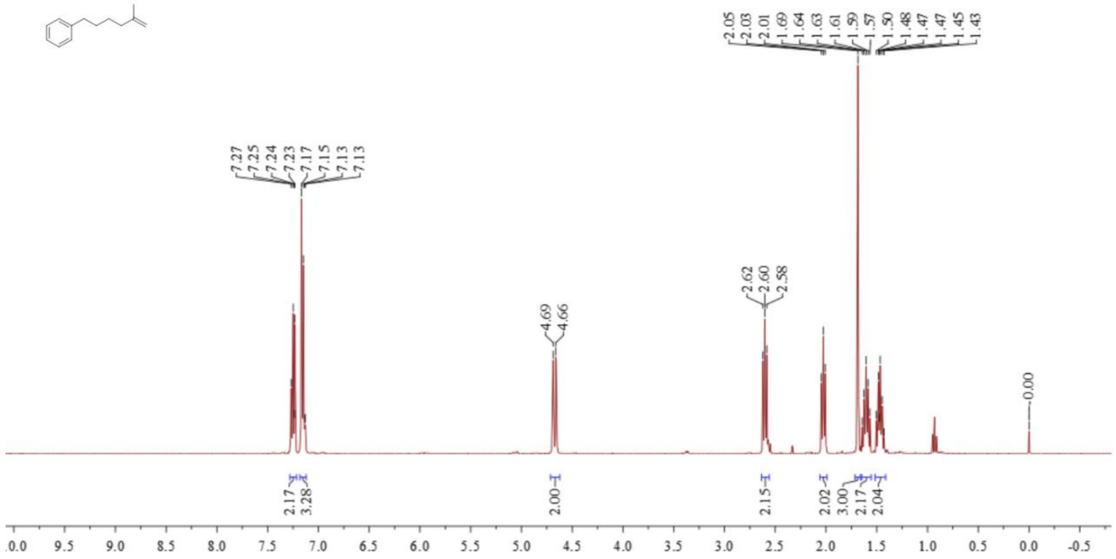

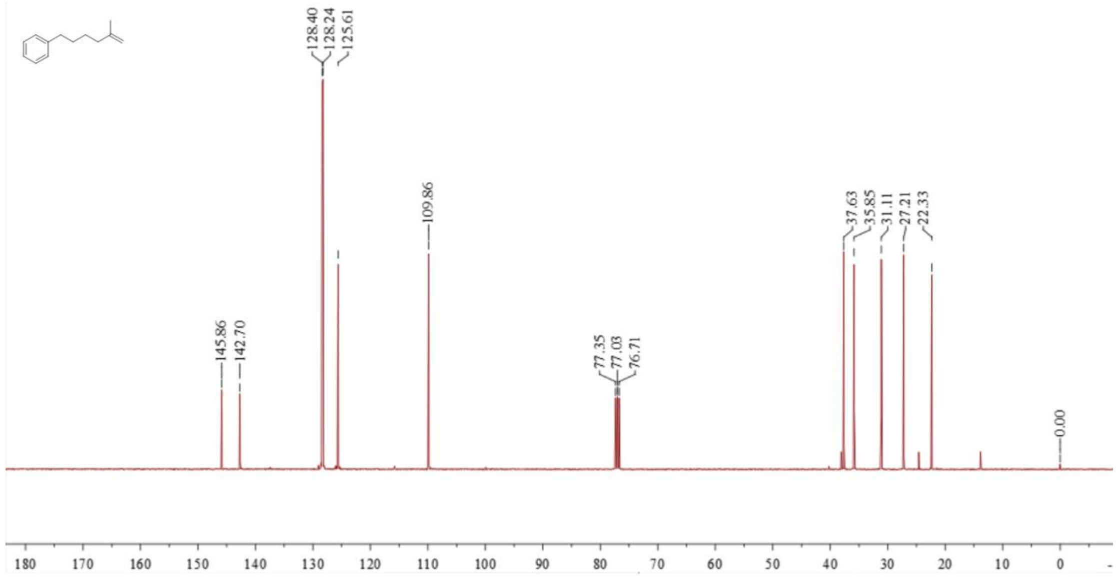

Supplementary Figure 13. ^{1}**H and** ^{13}**C NMR of compound 25**

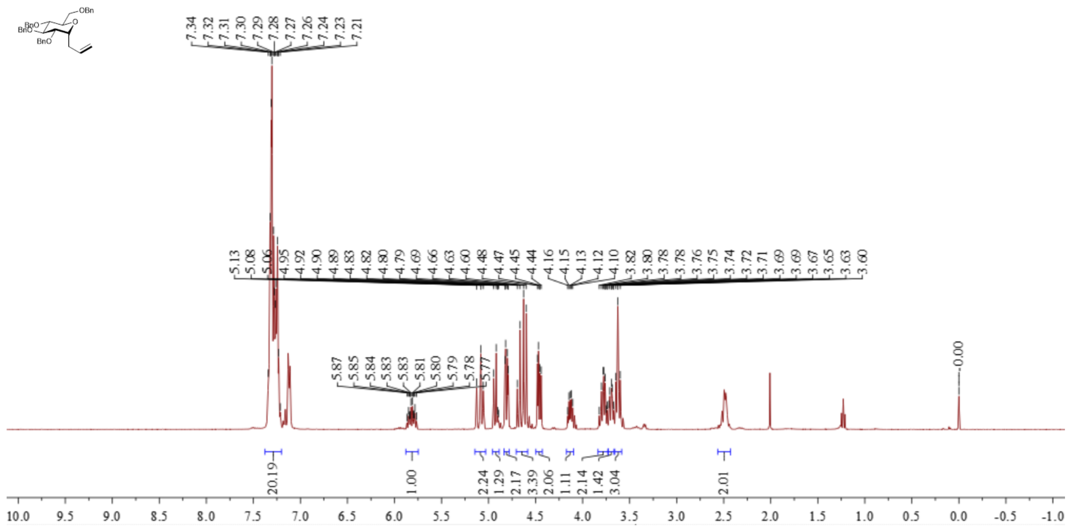

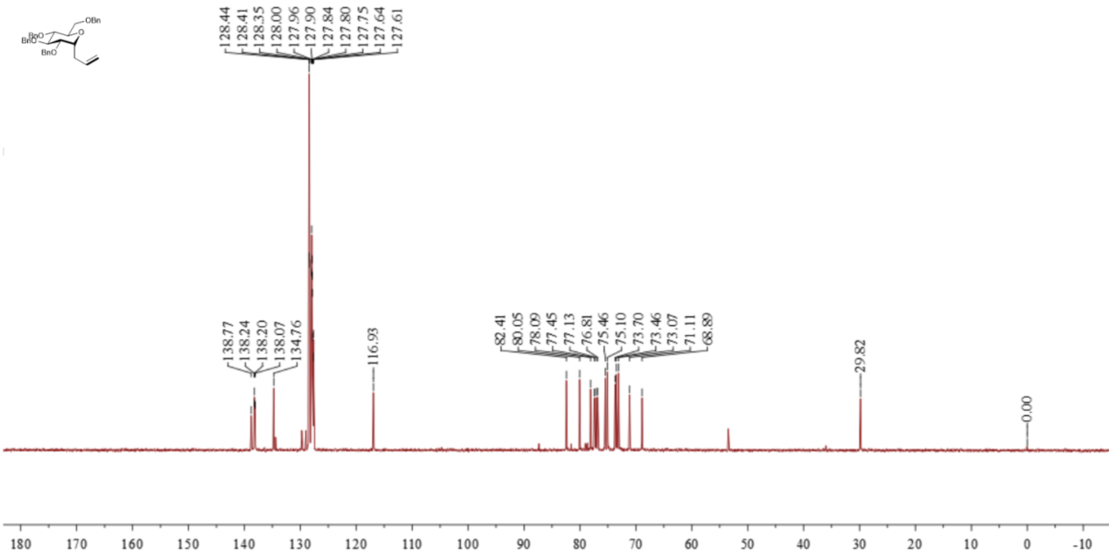

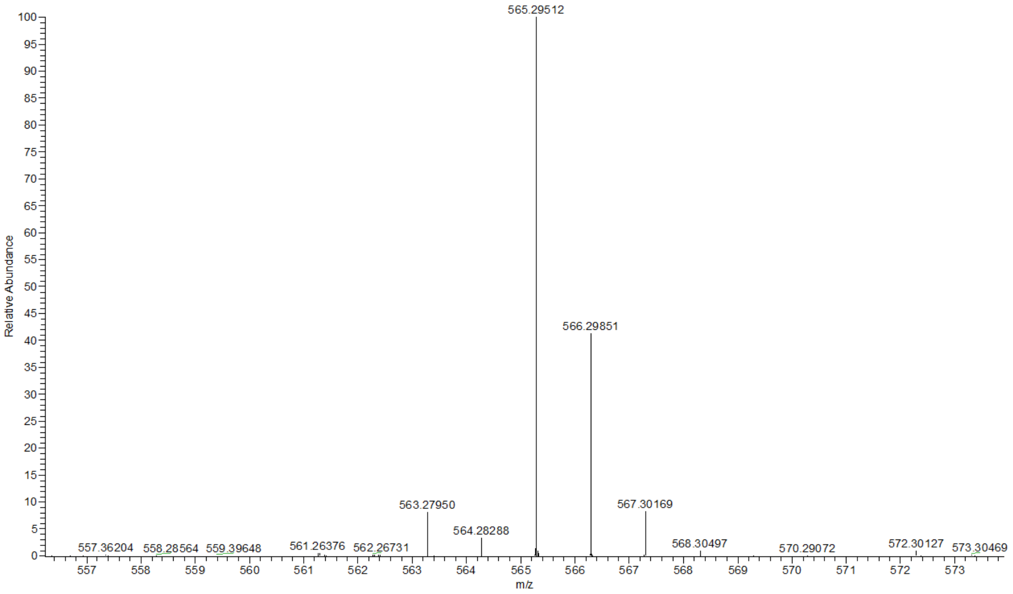

Supplementary Figure 14. ^{1}H, ^{13}C NMR and HRMS of compound 35

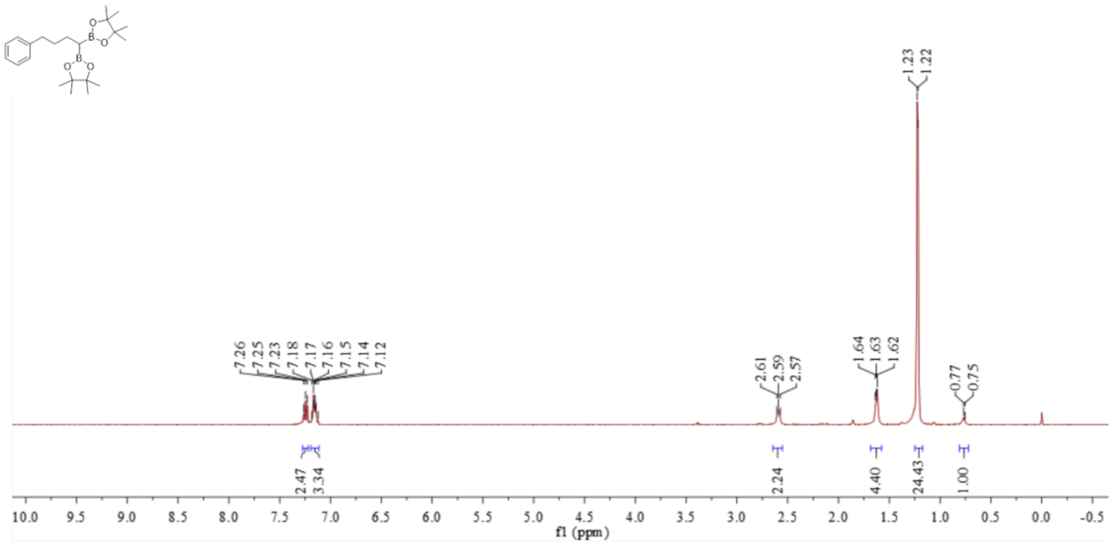

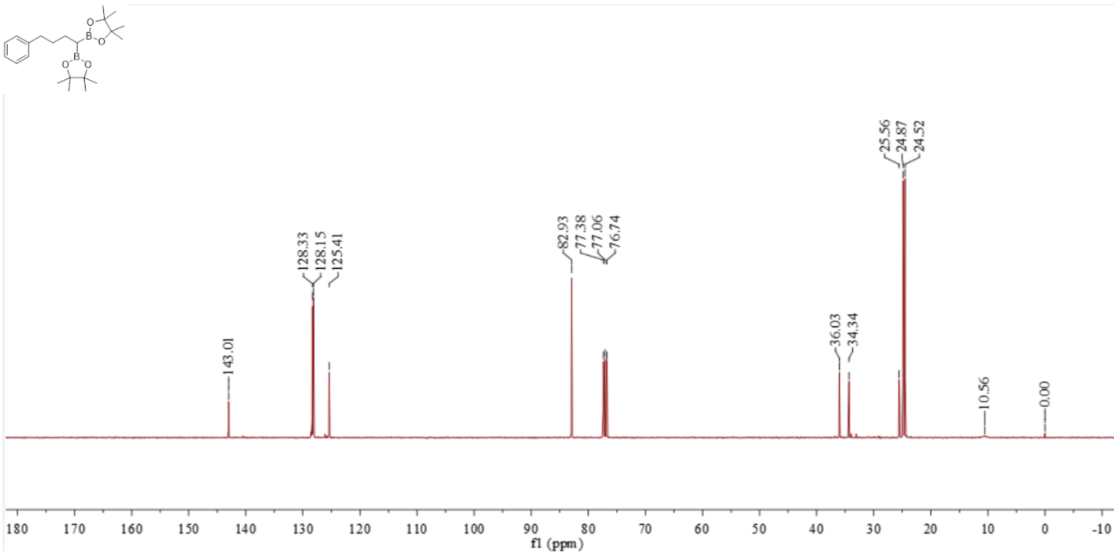

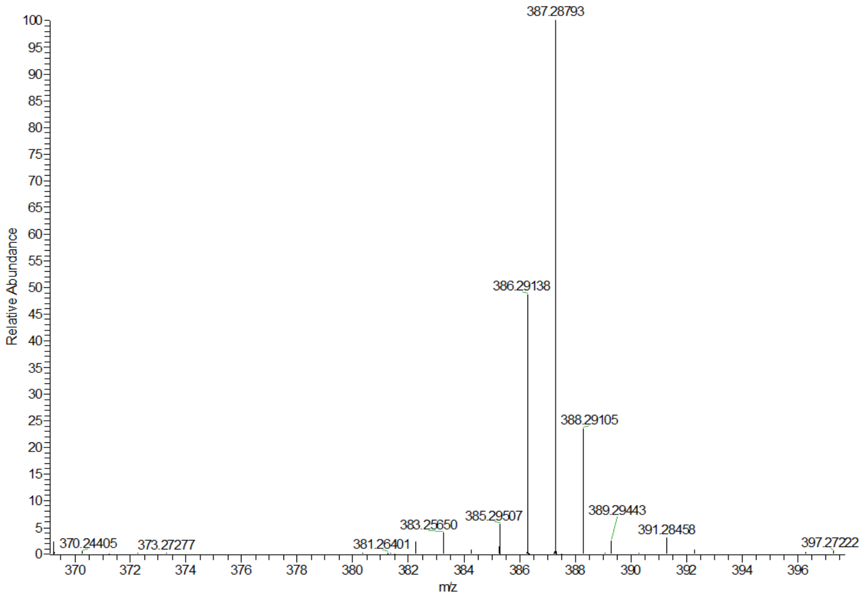

Supplementary Figure 15. ^{1}**H,** ^{13}**C NMR and HRMS of compound 2**

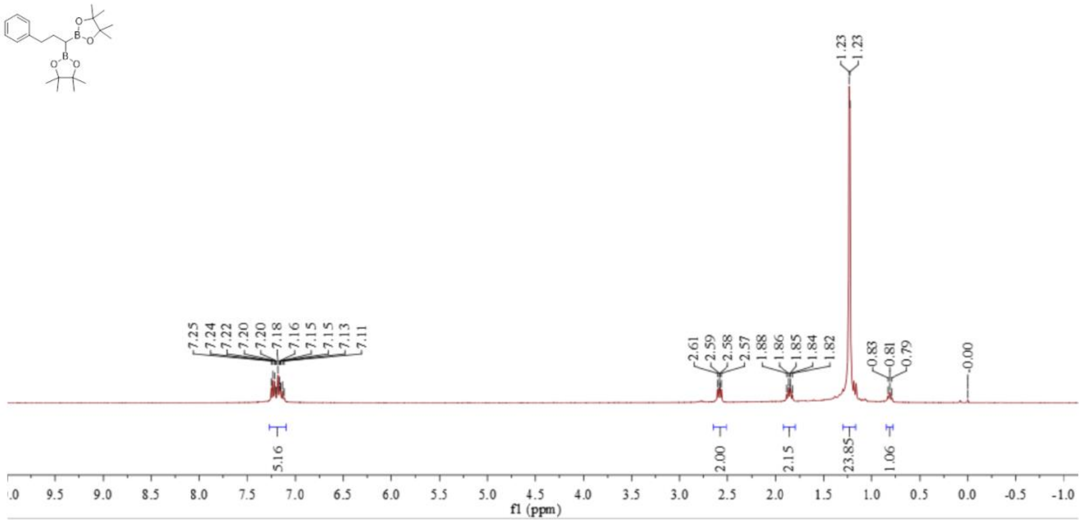

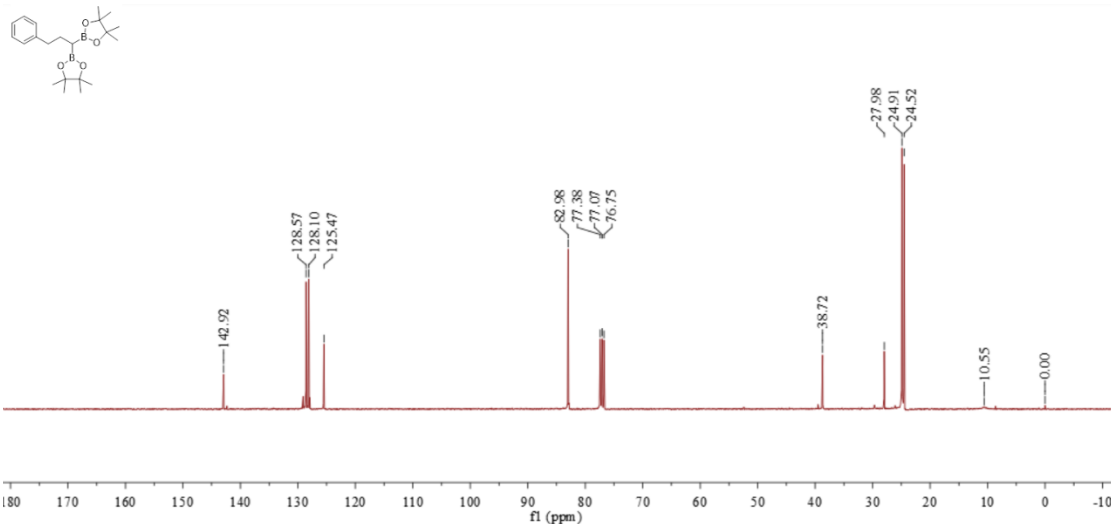

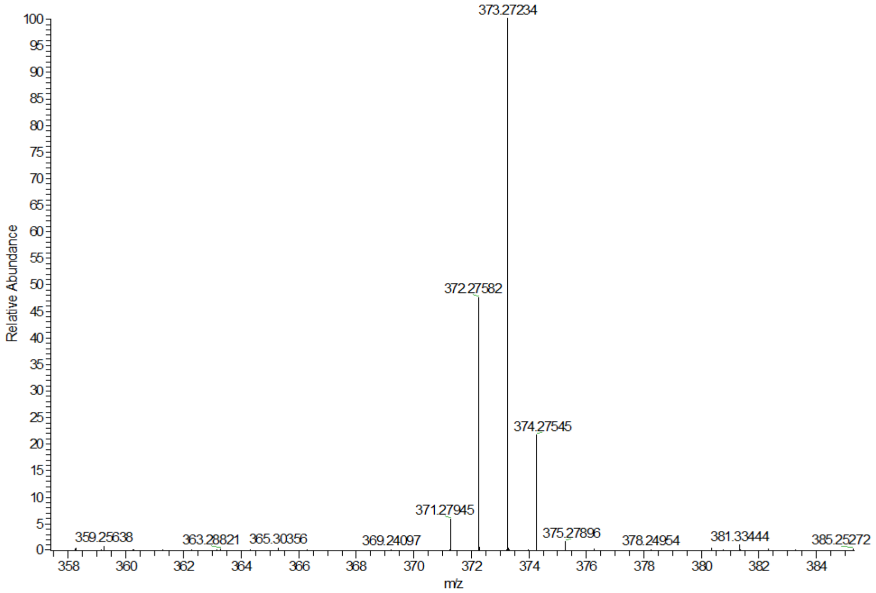

Supplementary Figure 16. ^{1}H, ^{13}C NMR and HRMS of compound 3

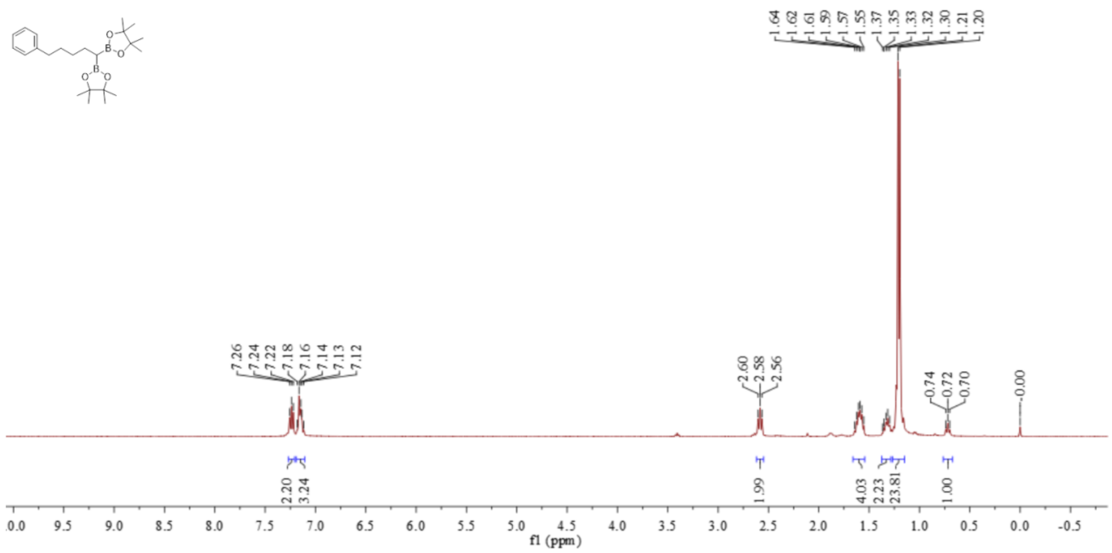

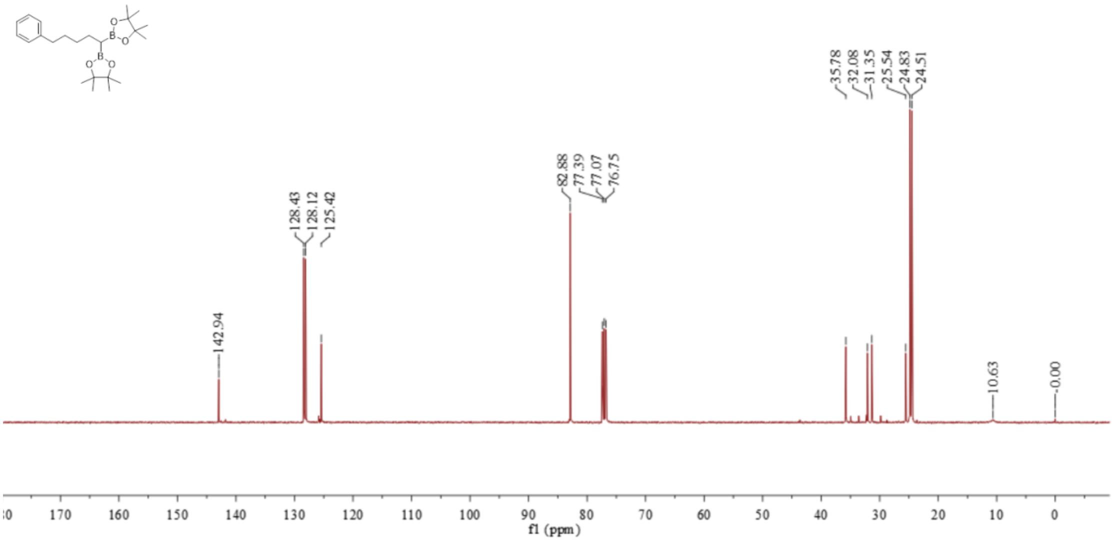

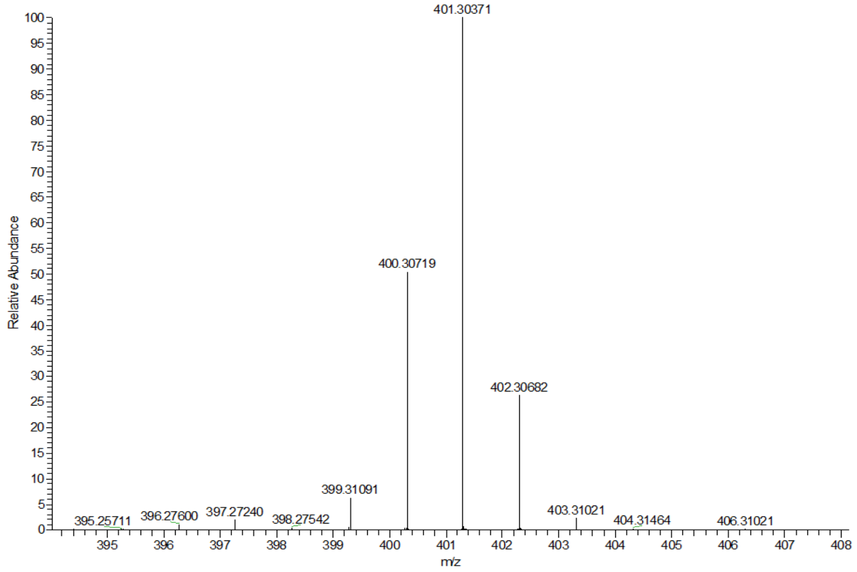

Supplementary Figure 17. ^{1}H, ^{13}C NMR and HRMS of compound 4

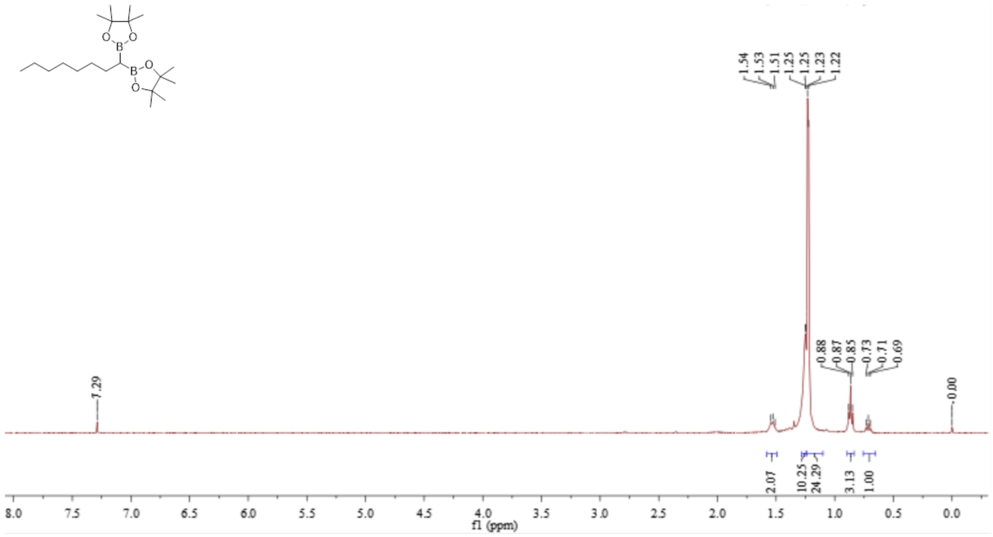

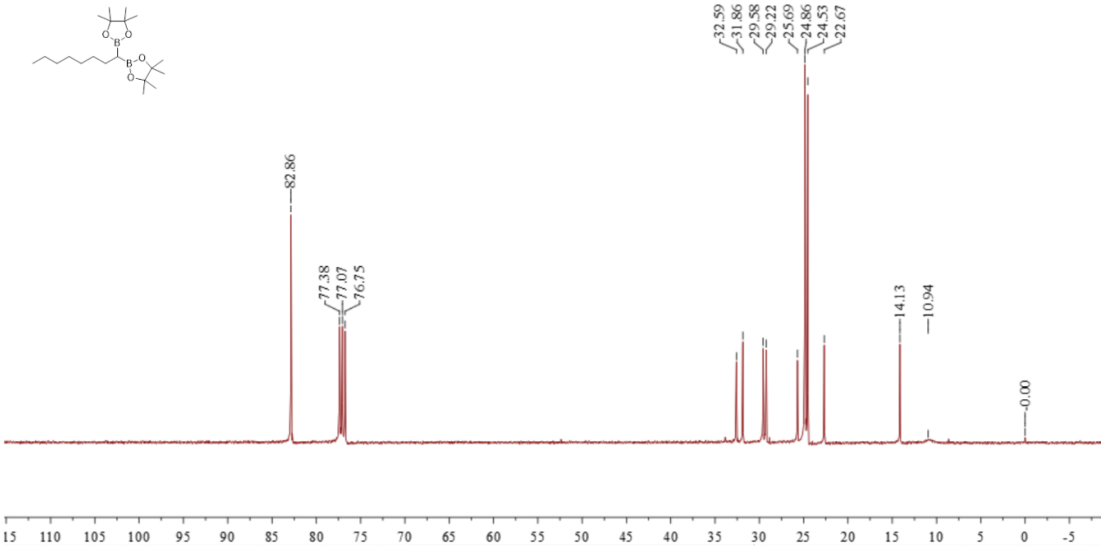

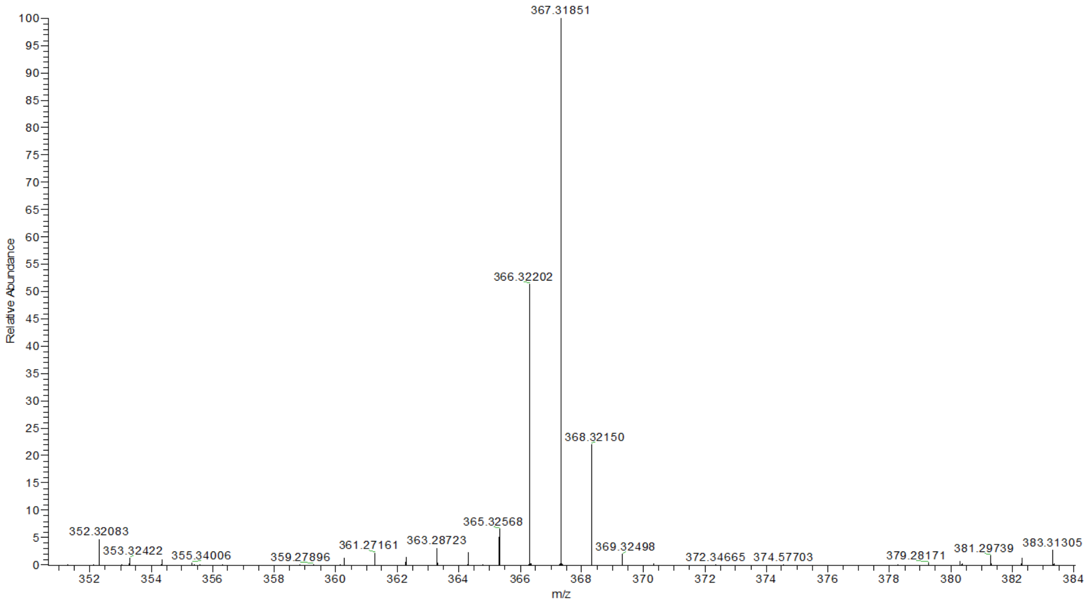

Supplementary Figure 18. ^{1}H, ^{13}C NMR and HRMS of compound 5

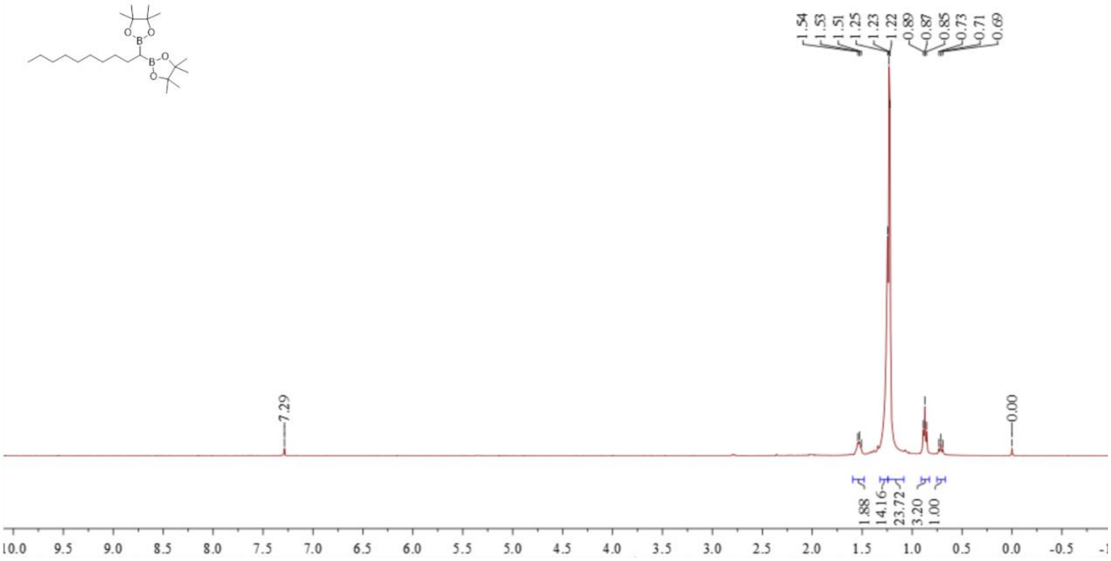

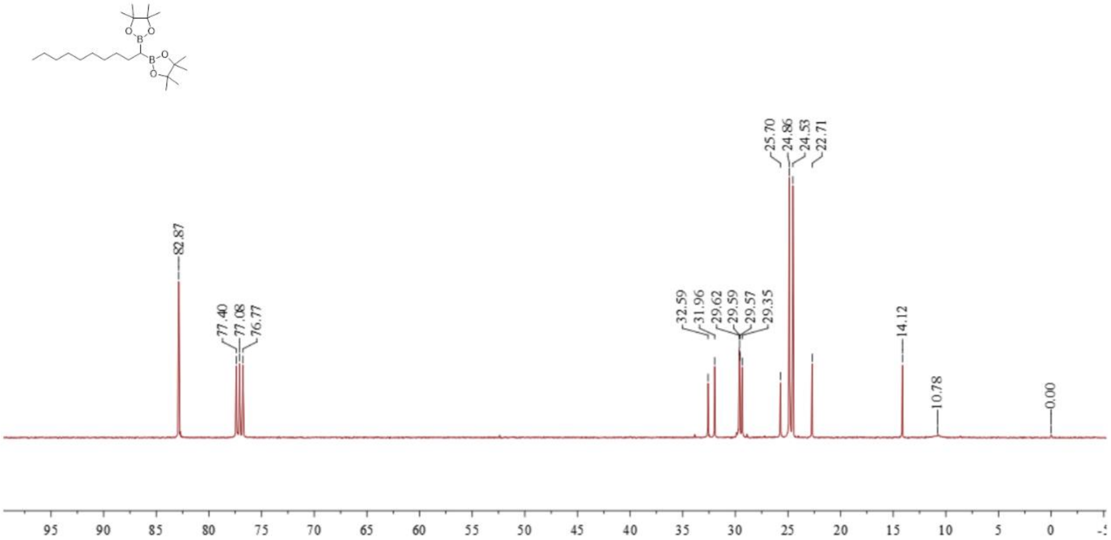

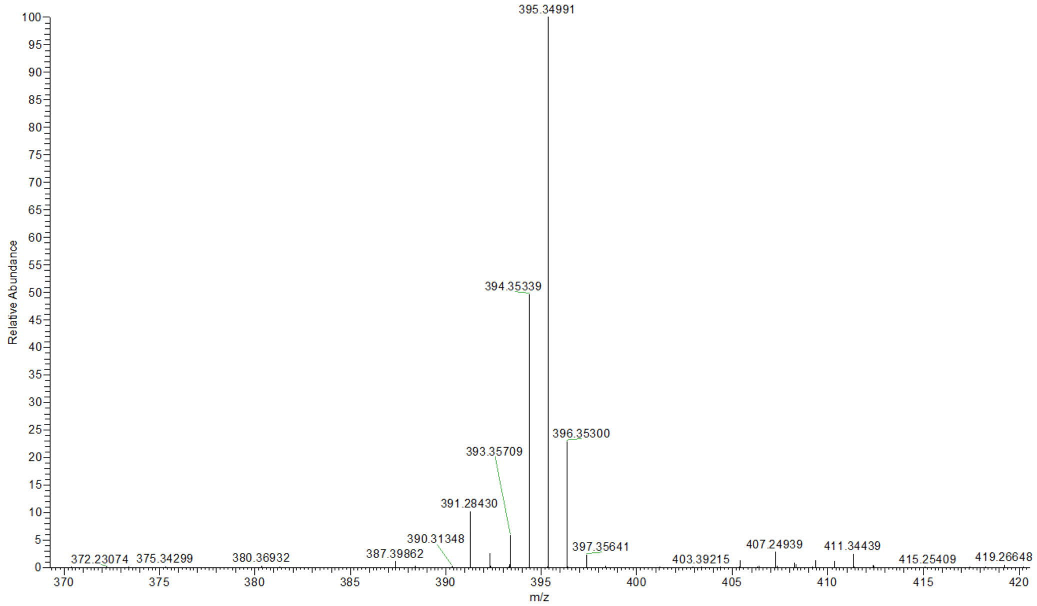

Supplementary Figure 19. **^{1}H, ^{13}C NMR and HRMS of compound 6**

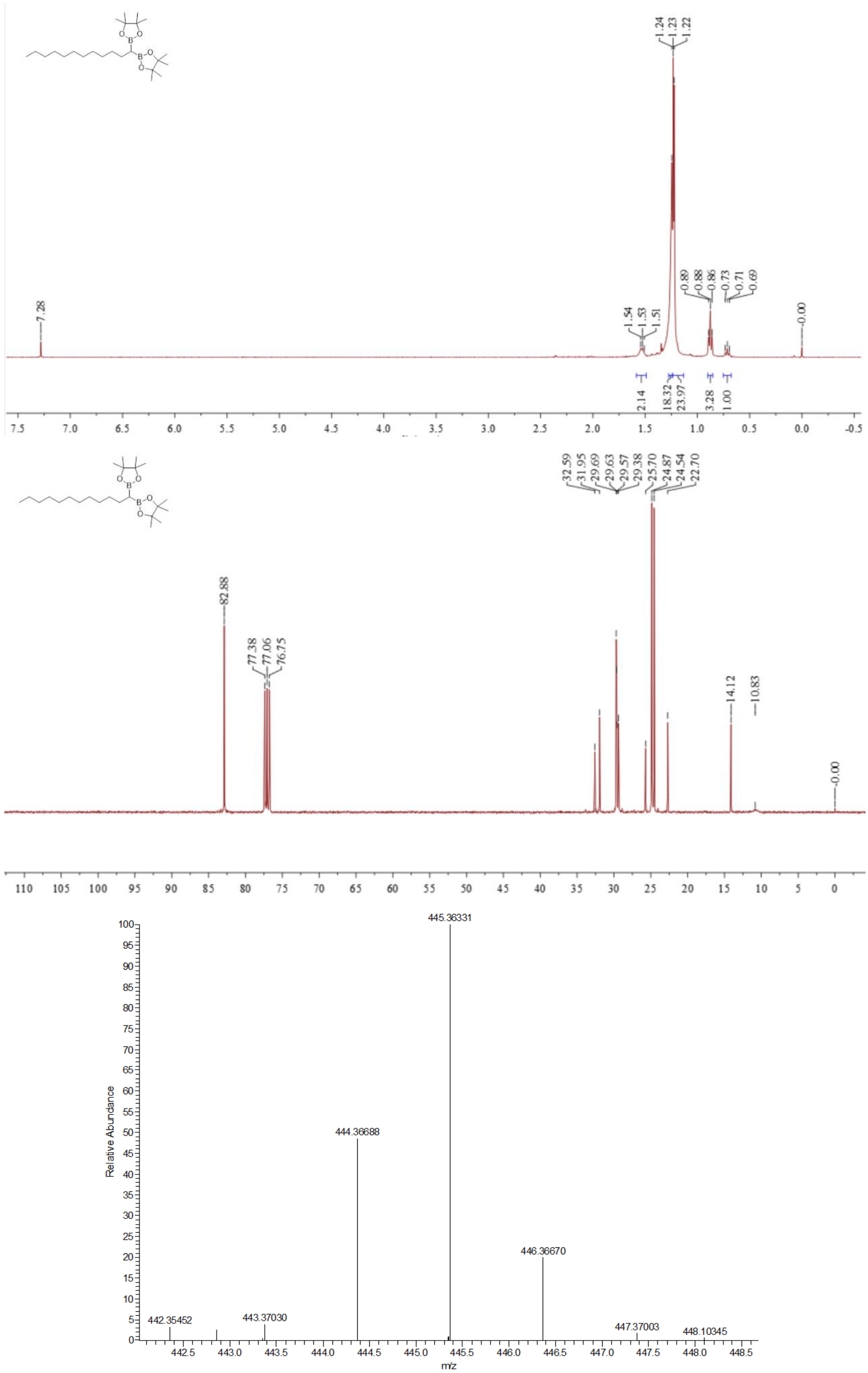

Supplementary Figure 20. ^{1}H, ^{13}C NMR and HRMS of compound 7

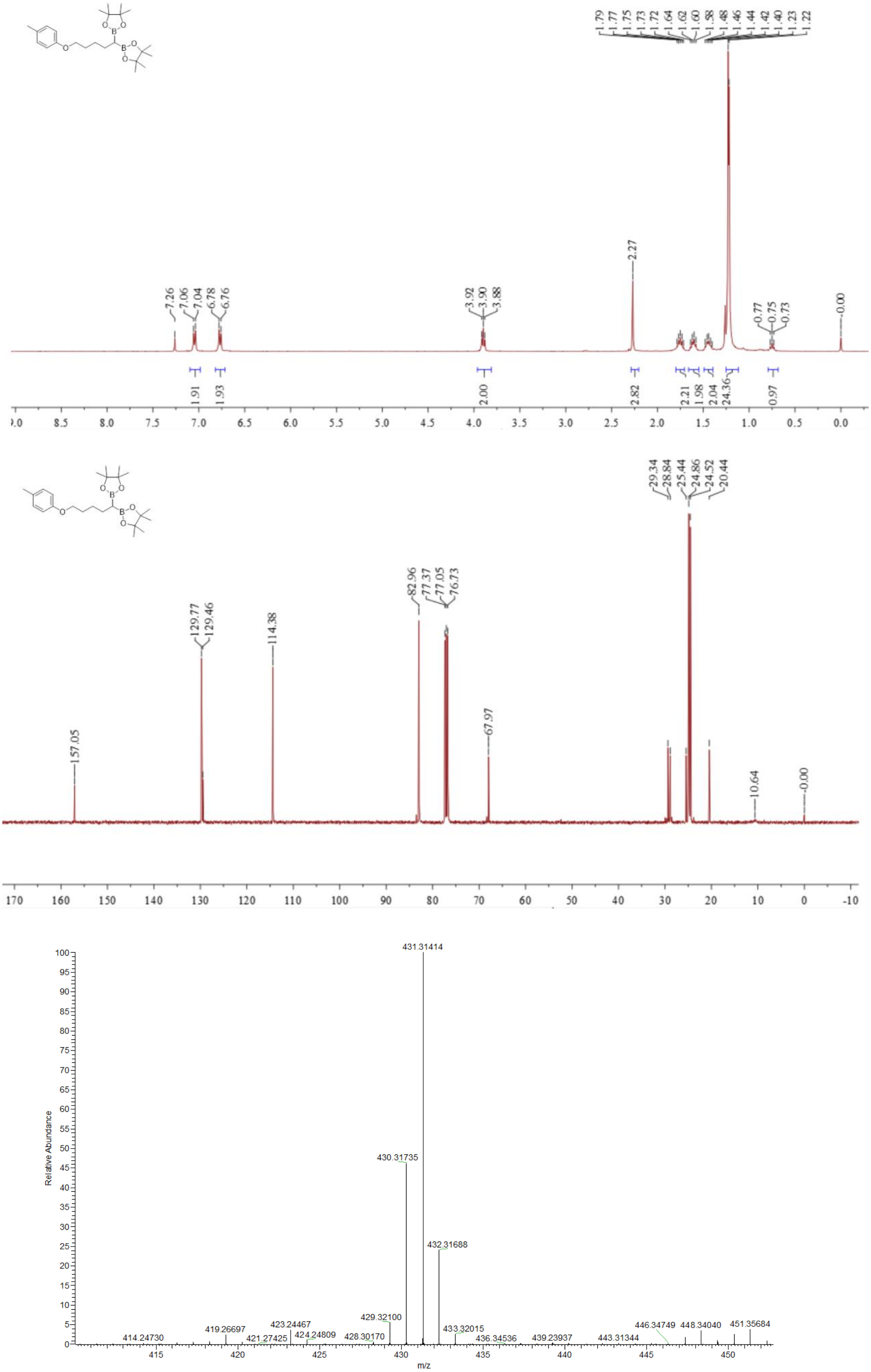

Supplementary Figure 21. ^{1}**H,** ^{13}**C NMR and HRMS of compound 8**

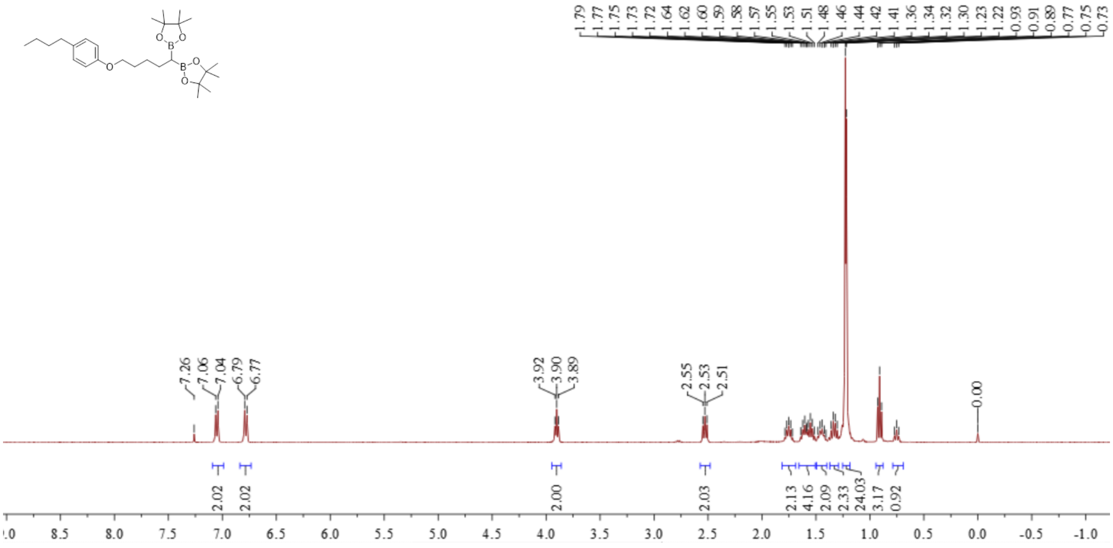

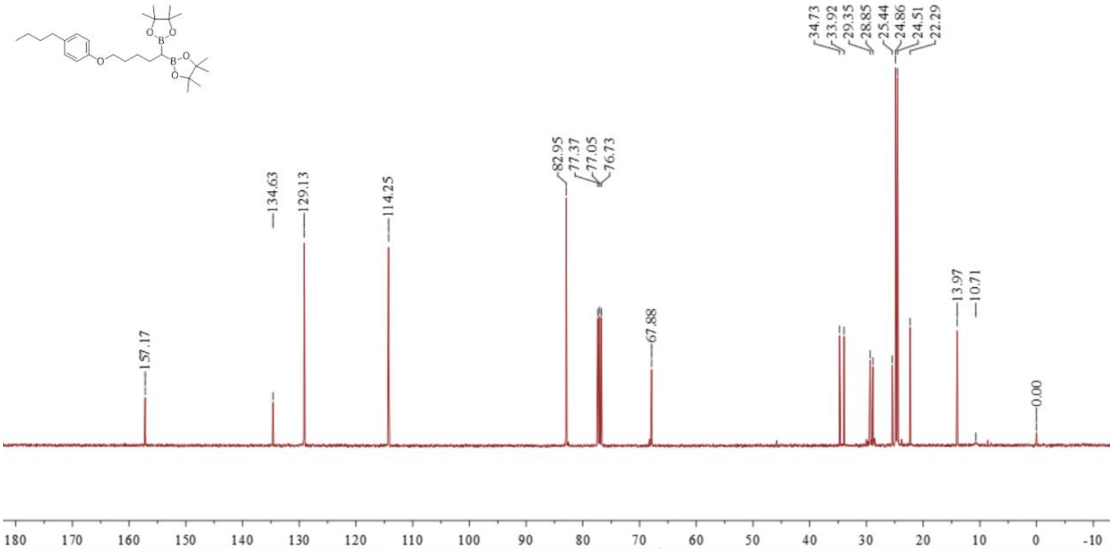

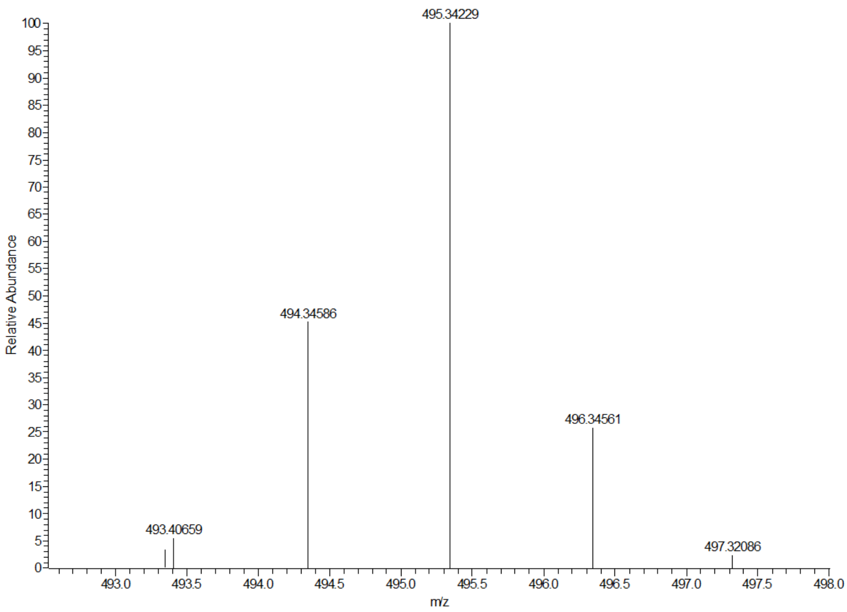

Supplementary Figure 22. ^{1}**H,** ^{13}**C NMR and HRMS of compound 9**

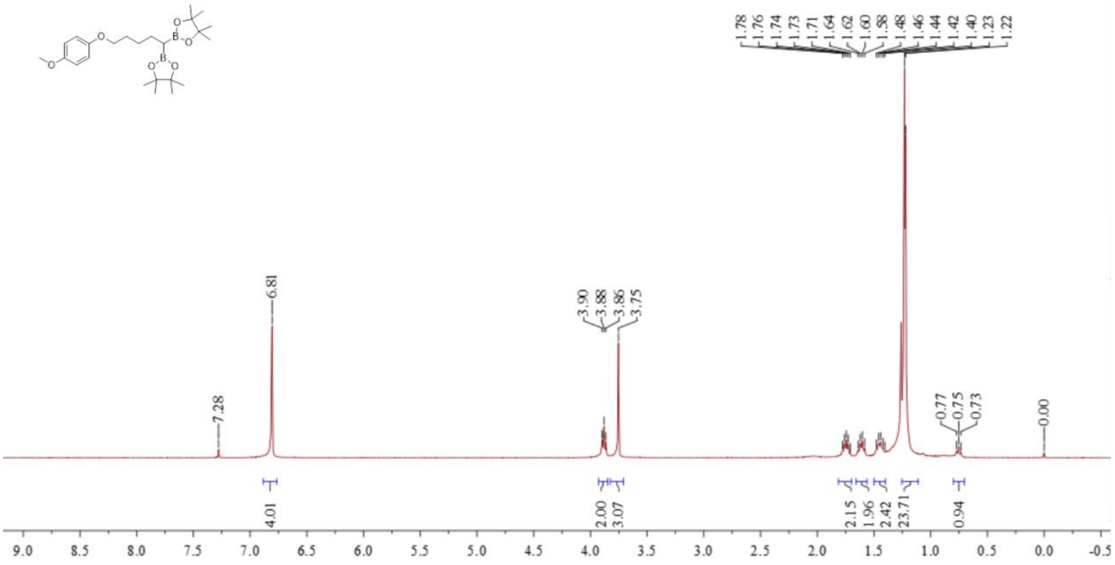

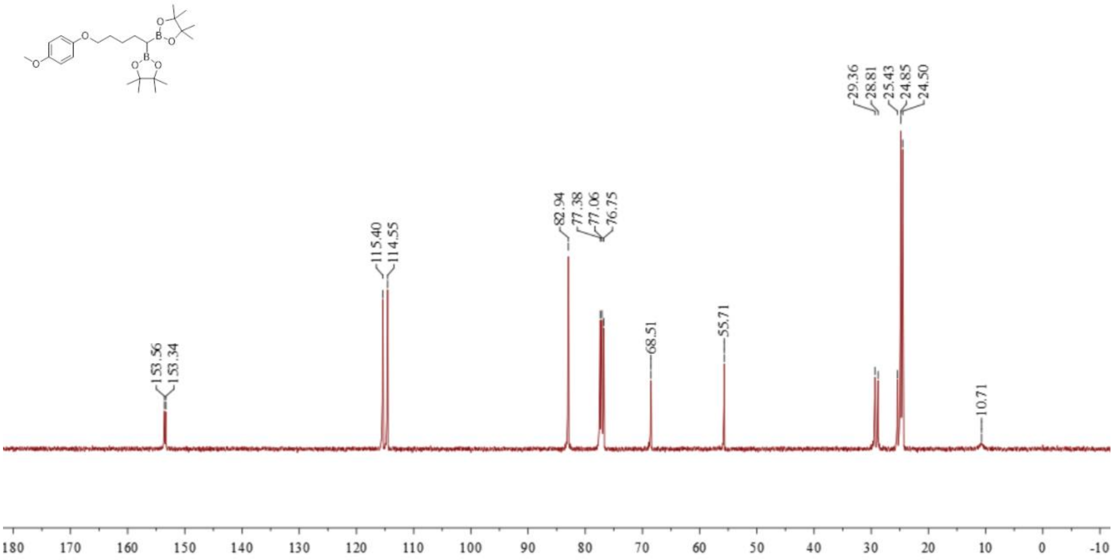

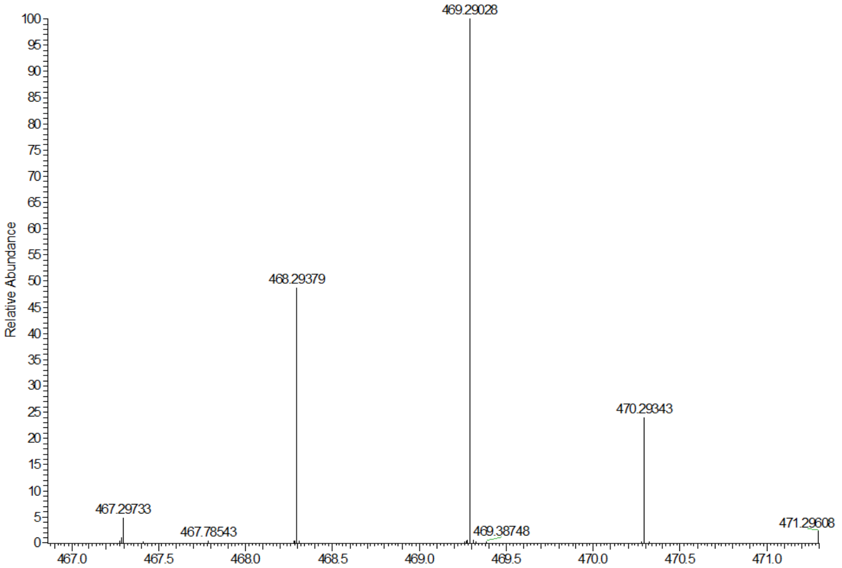

Supplementary Figure 23. ^{1}H, ^{13}C NMR and HRMS of compound 10

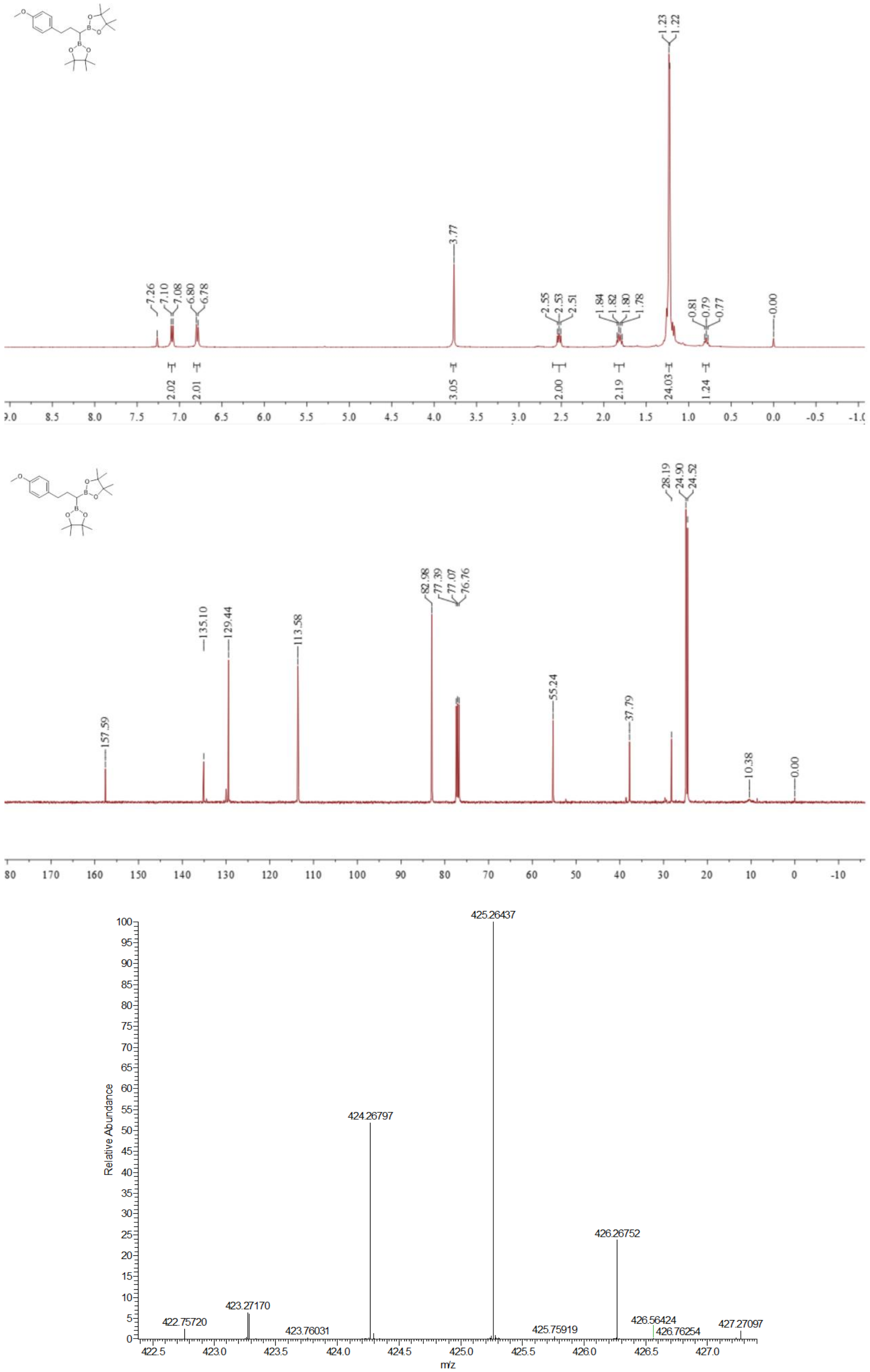

Supplementary Figure 24. ^{1}H, ^{13}C NMR and HRMS of compound 11

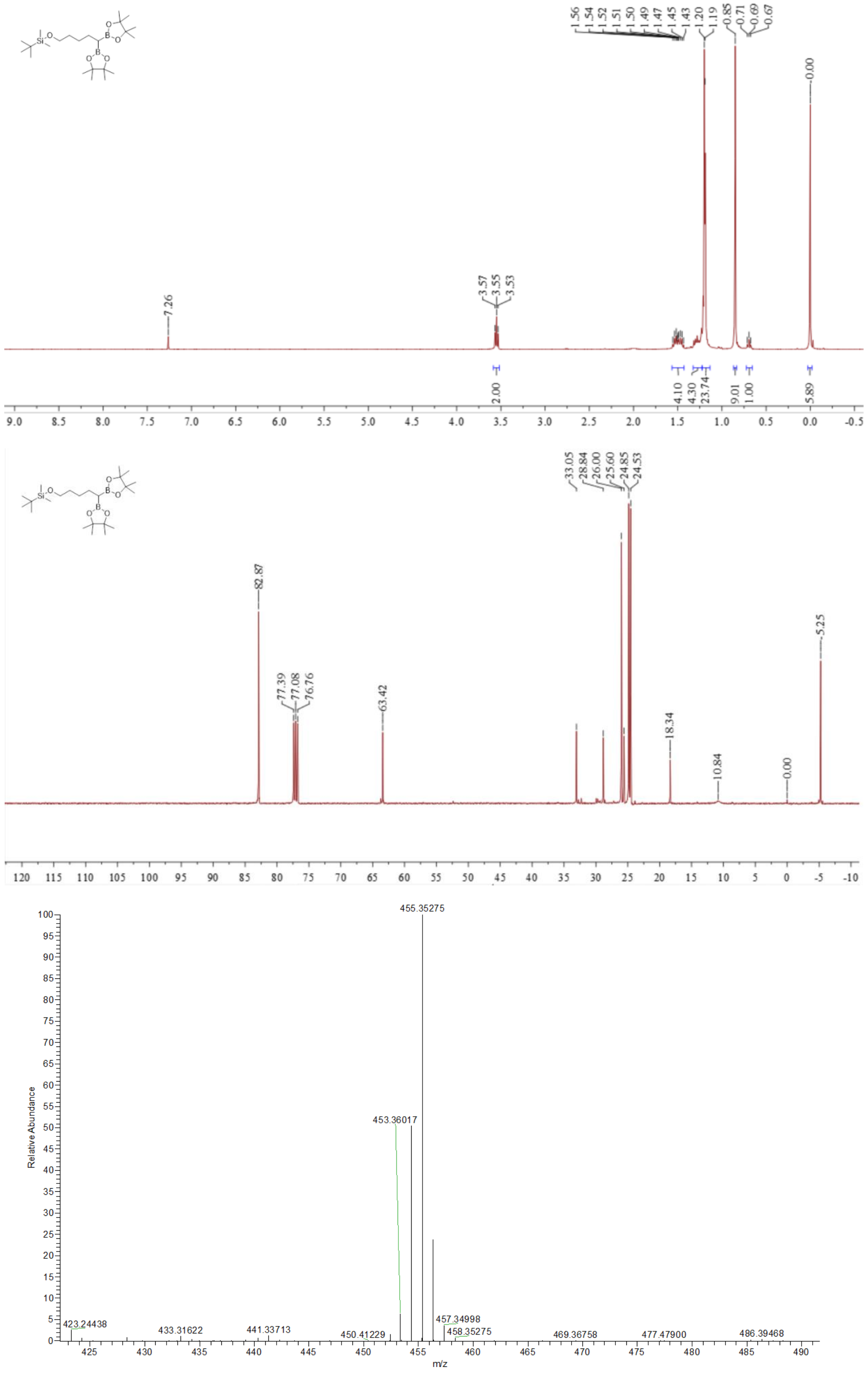

Supplementary Figure 25. ^{1}H, ^{13}C **NMR and HRMS of compound 12**

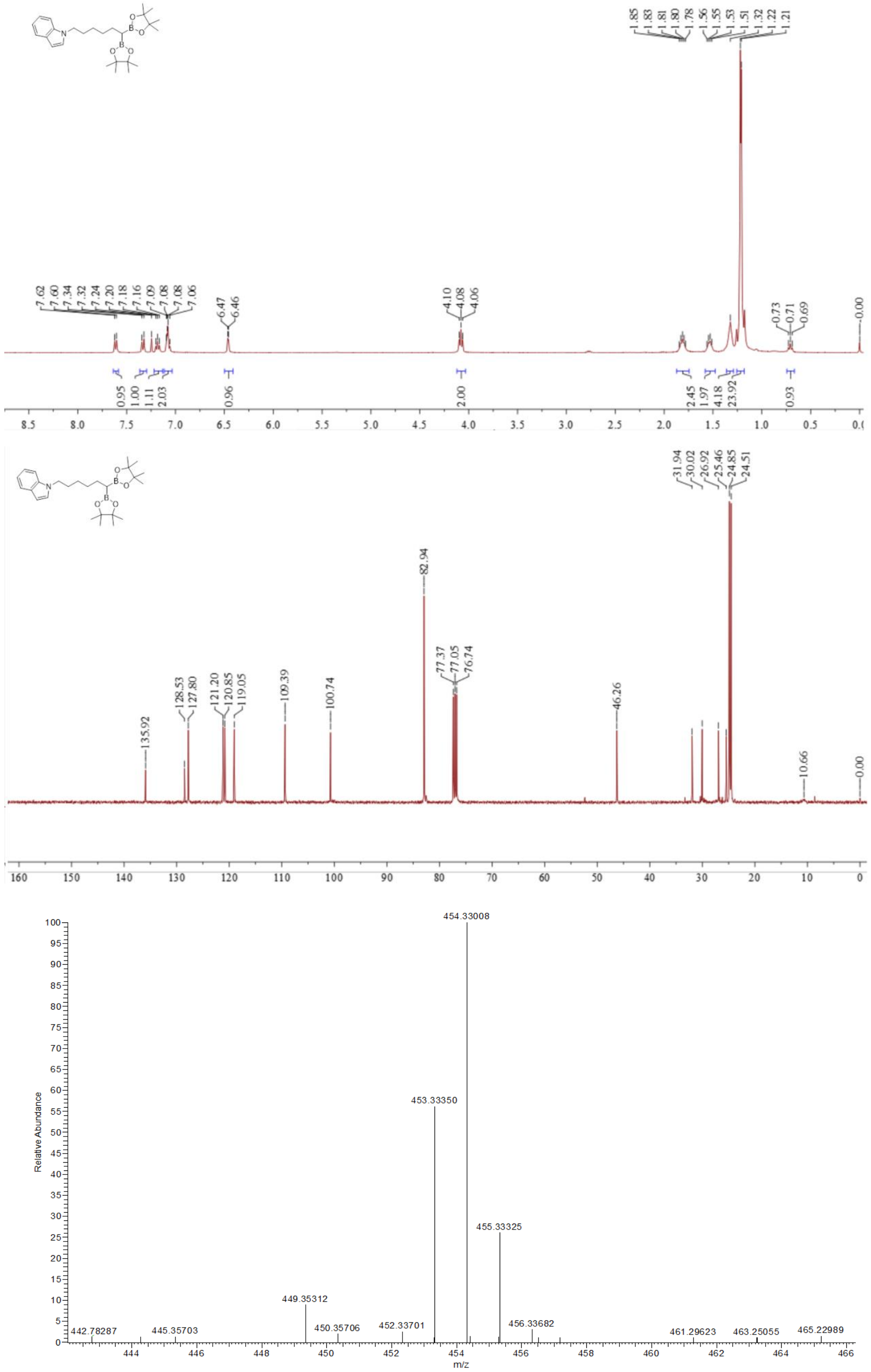

Supplementary Figure 26. ^{1}H, ^{13}C NMR and HRMS of compound 13

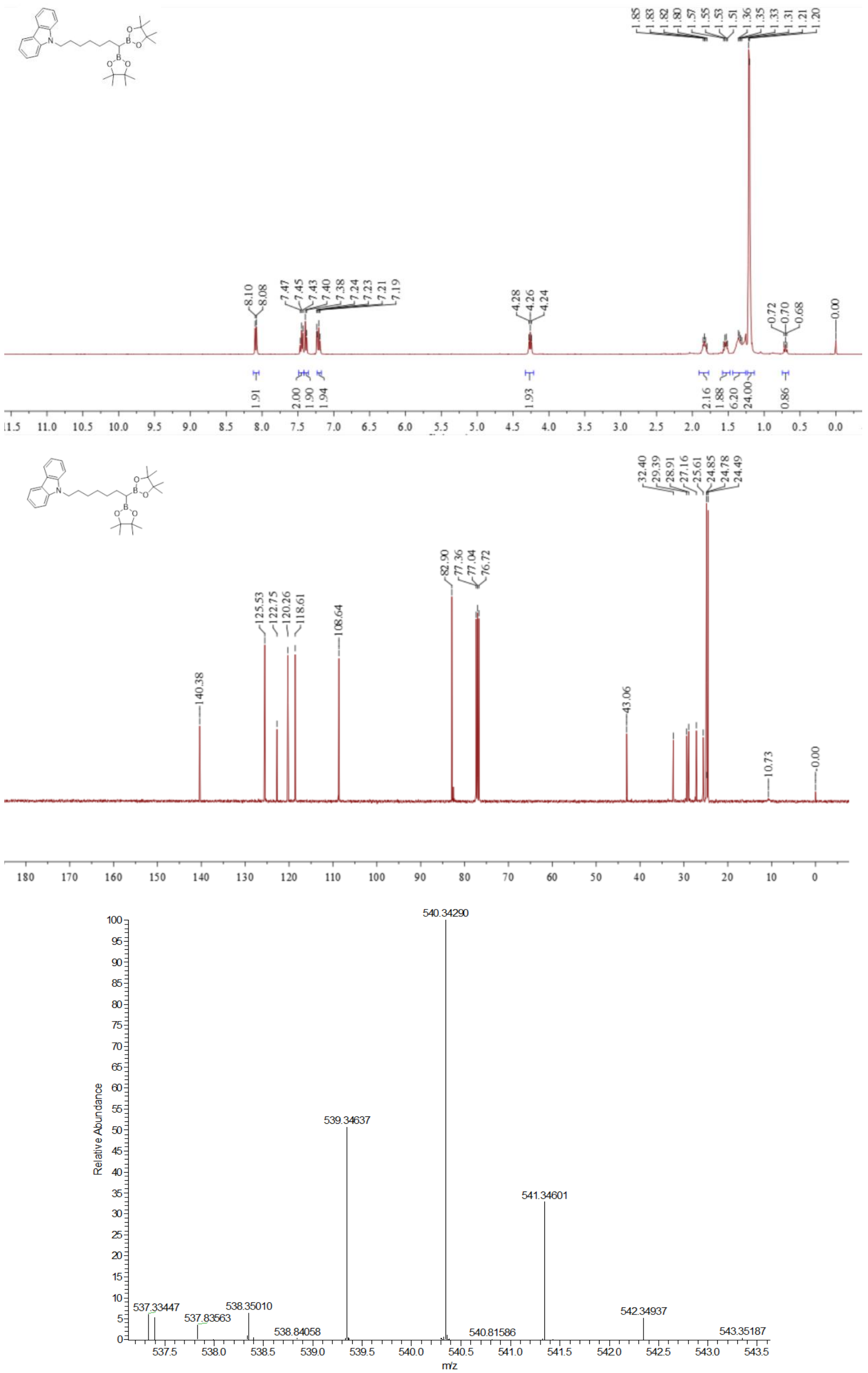

Supplementary Figure 27. ^{1}**H,** ^{13}**C NMR and HRMS of compound 14**

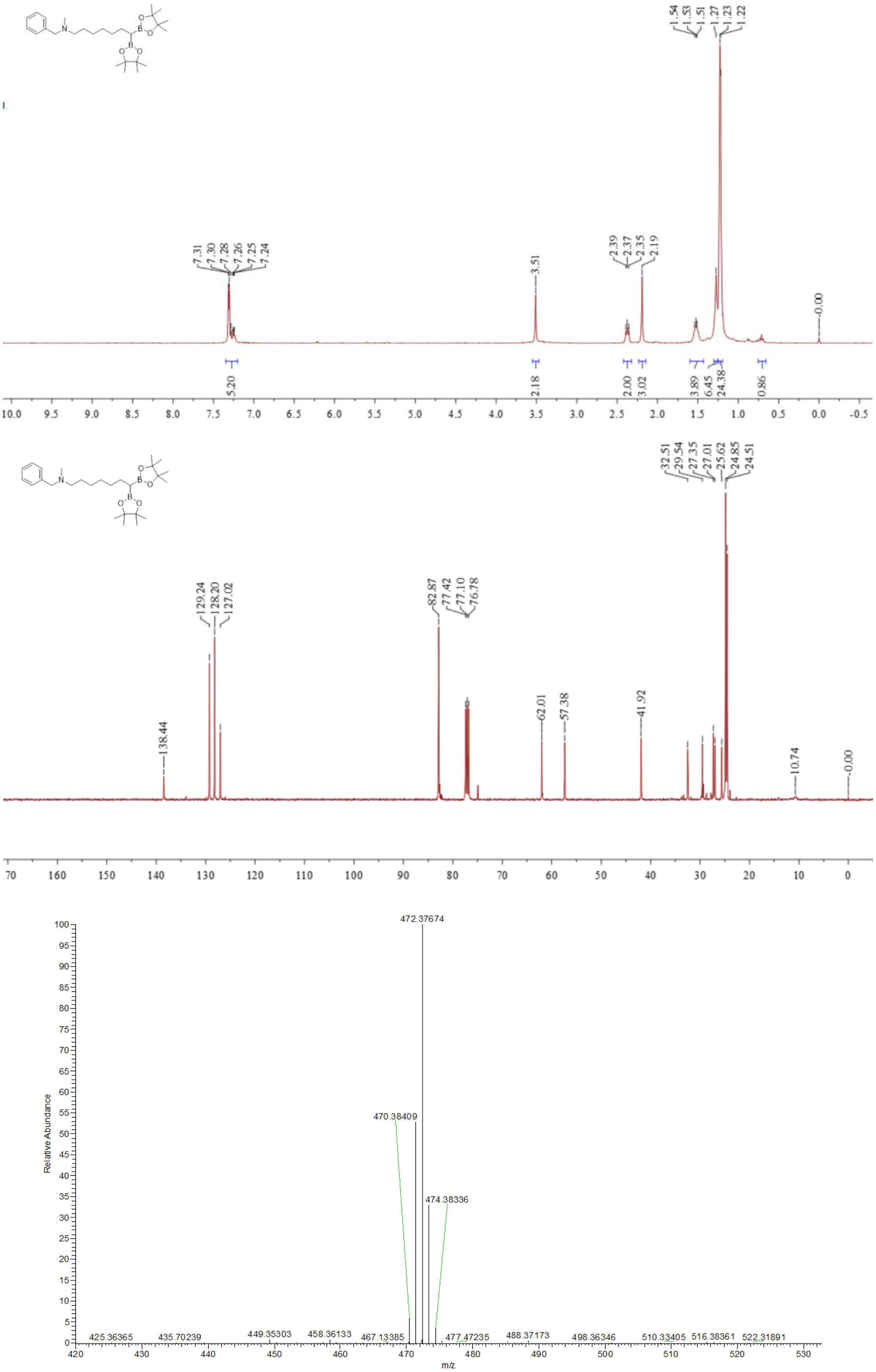

Supplementary Figure 28. ^{1}**H,** ^{13}**C NMR and HRMS of compound 15**

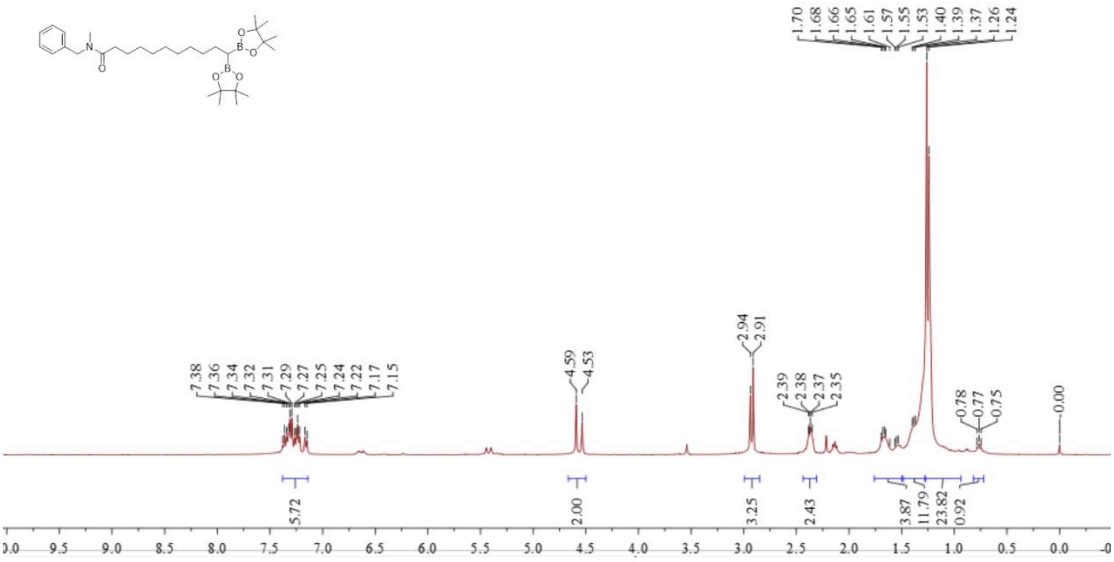

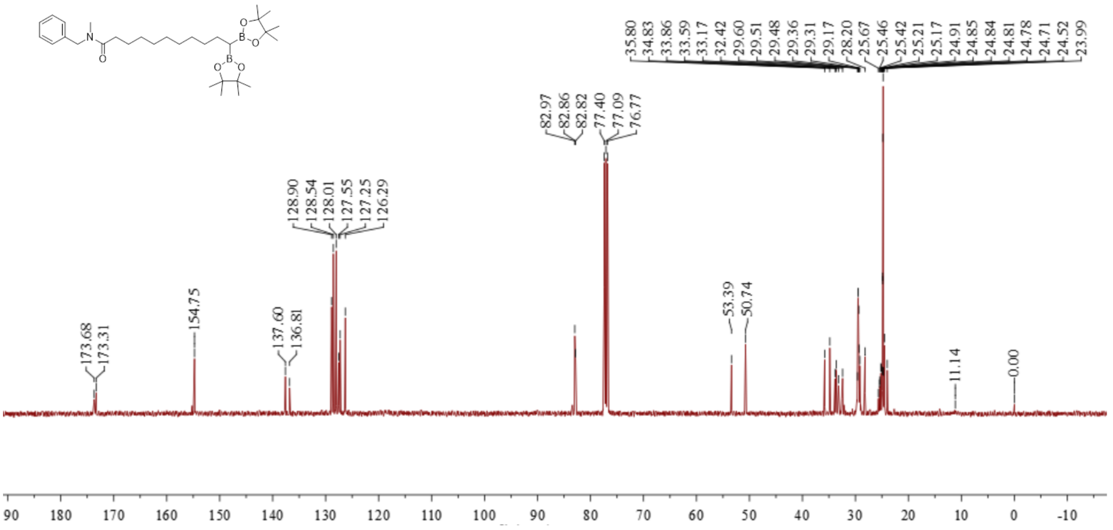

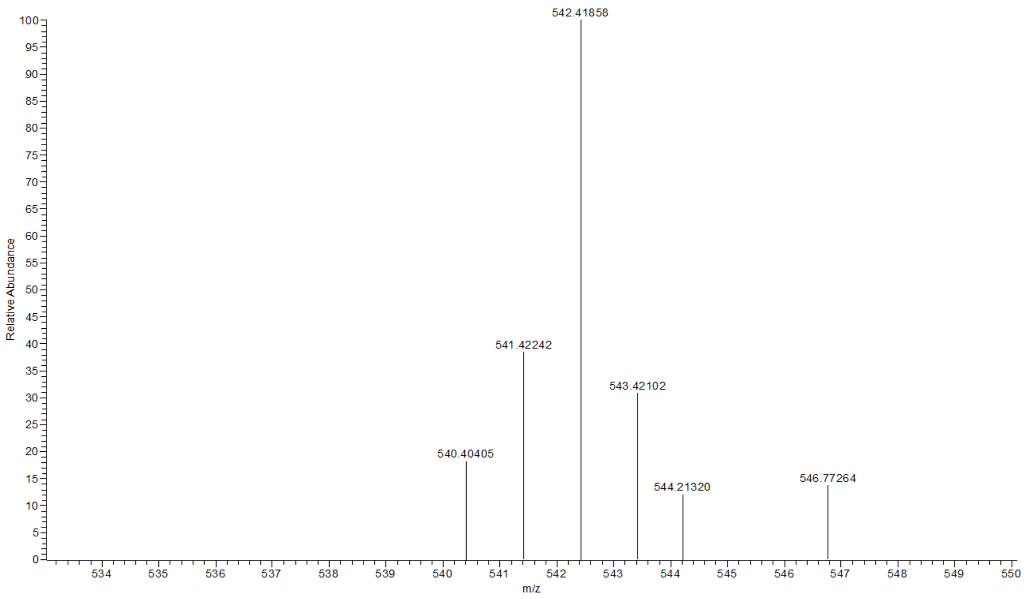

Supplementary Figure 29. ^{1}**H,** ^{13}**C NMR and HRMS of compound 16**

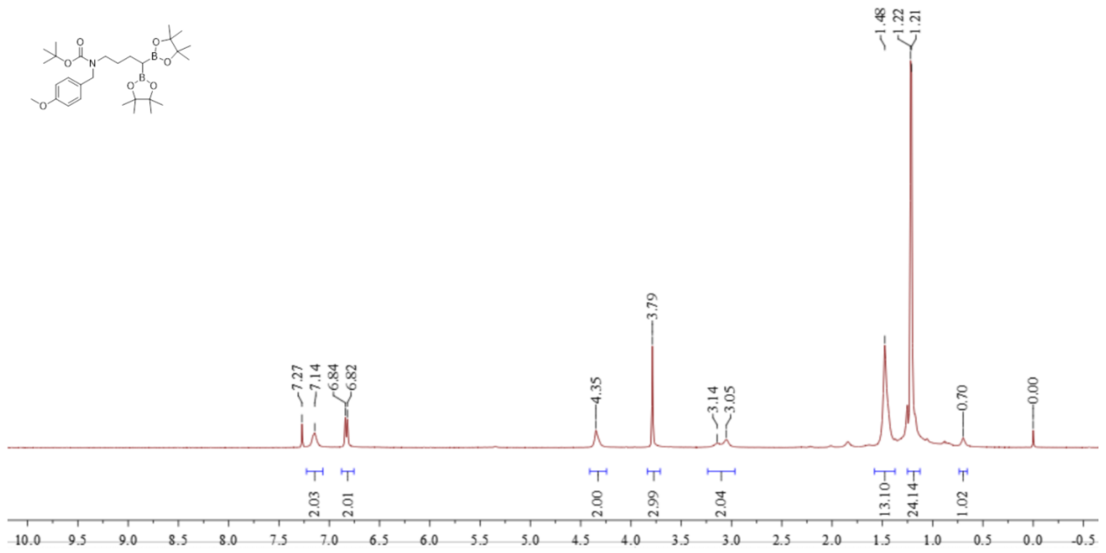

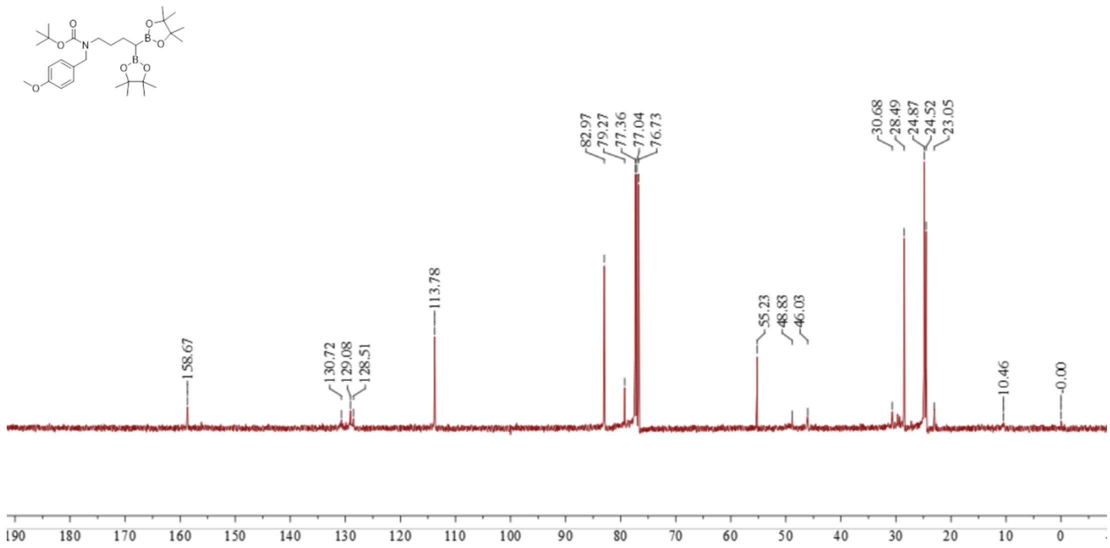

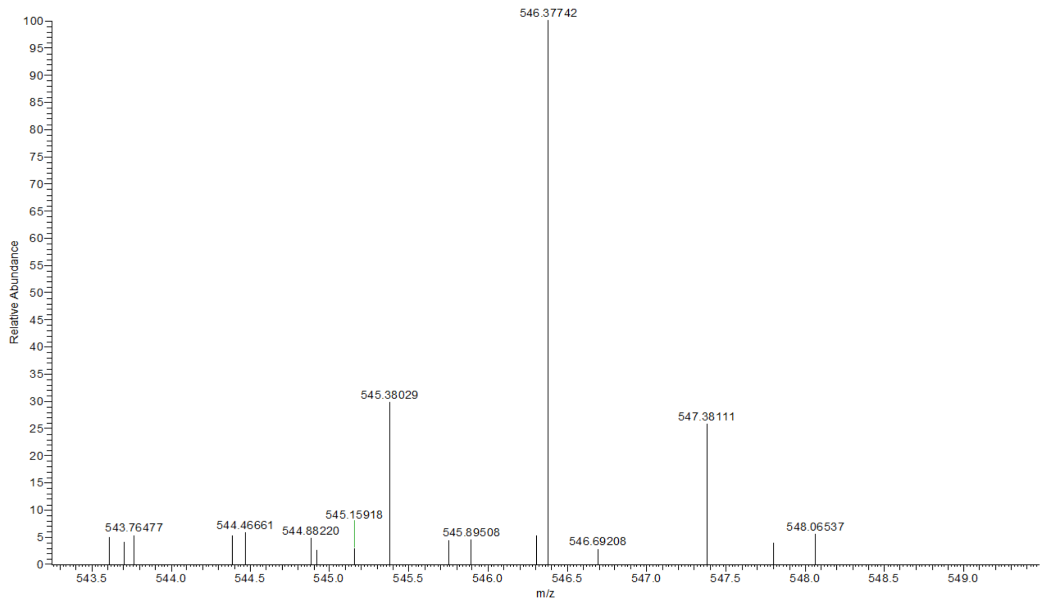

Supplementary Figure 30. **^{1}H, ^{13}C NMR and HRMS of compound 17**

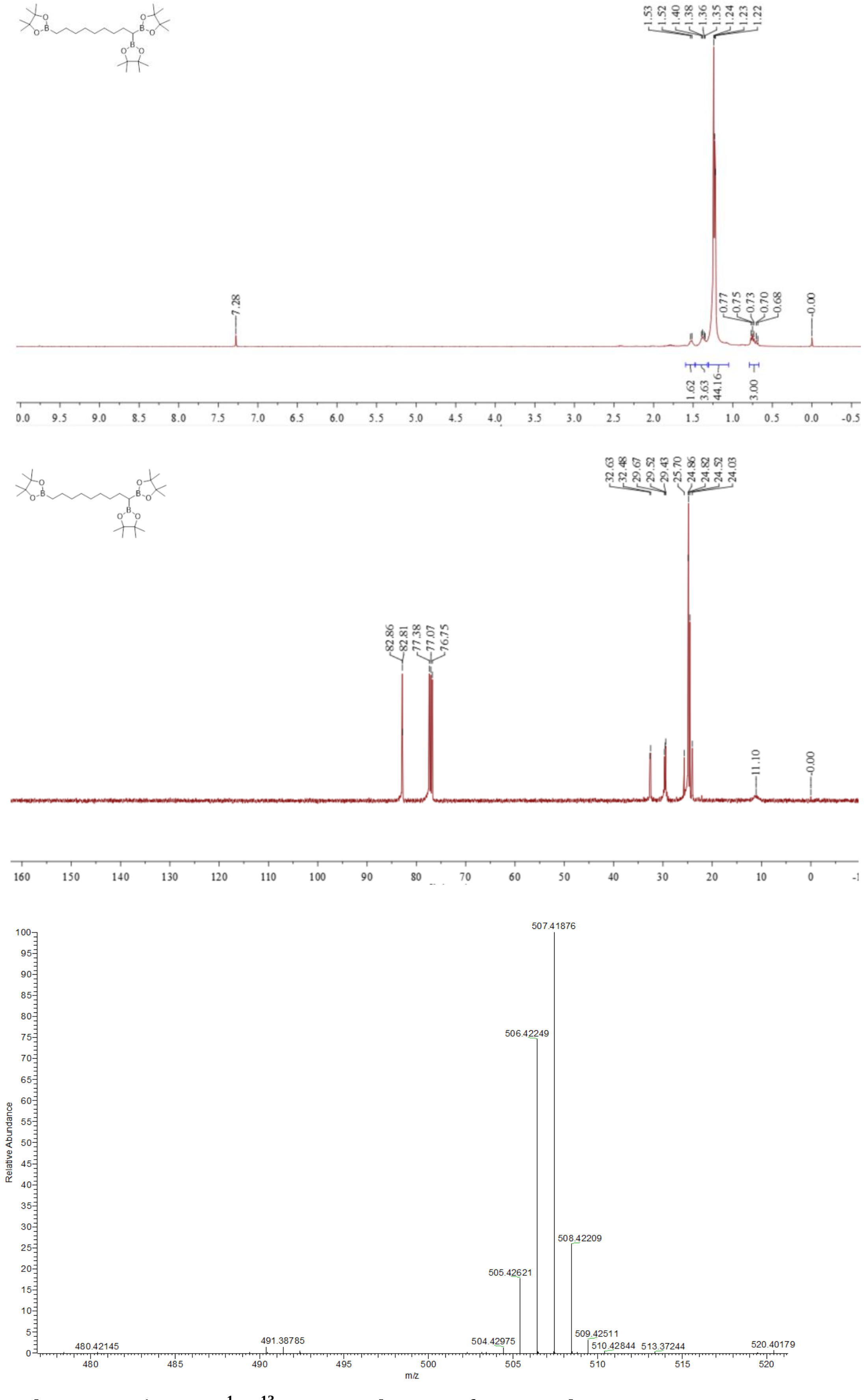

Supplementary Figure 31. ^{1}**H,** ^{13}**C NMR and HRMS of compound 18**

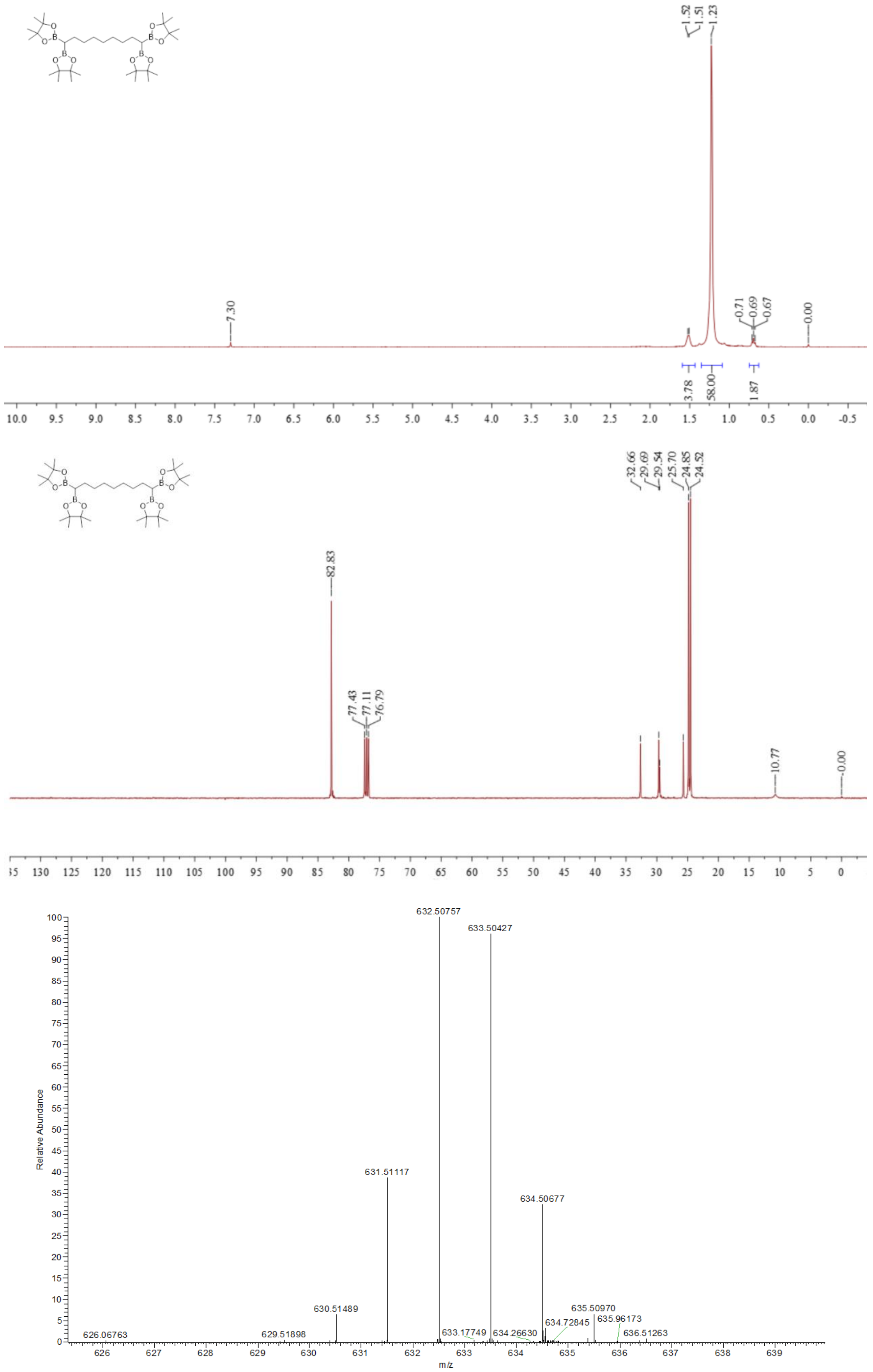

Supplementary Figure 32. ^{1}**H,** ^{13}**C NMR and HRMS of compound 19**

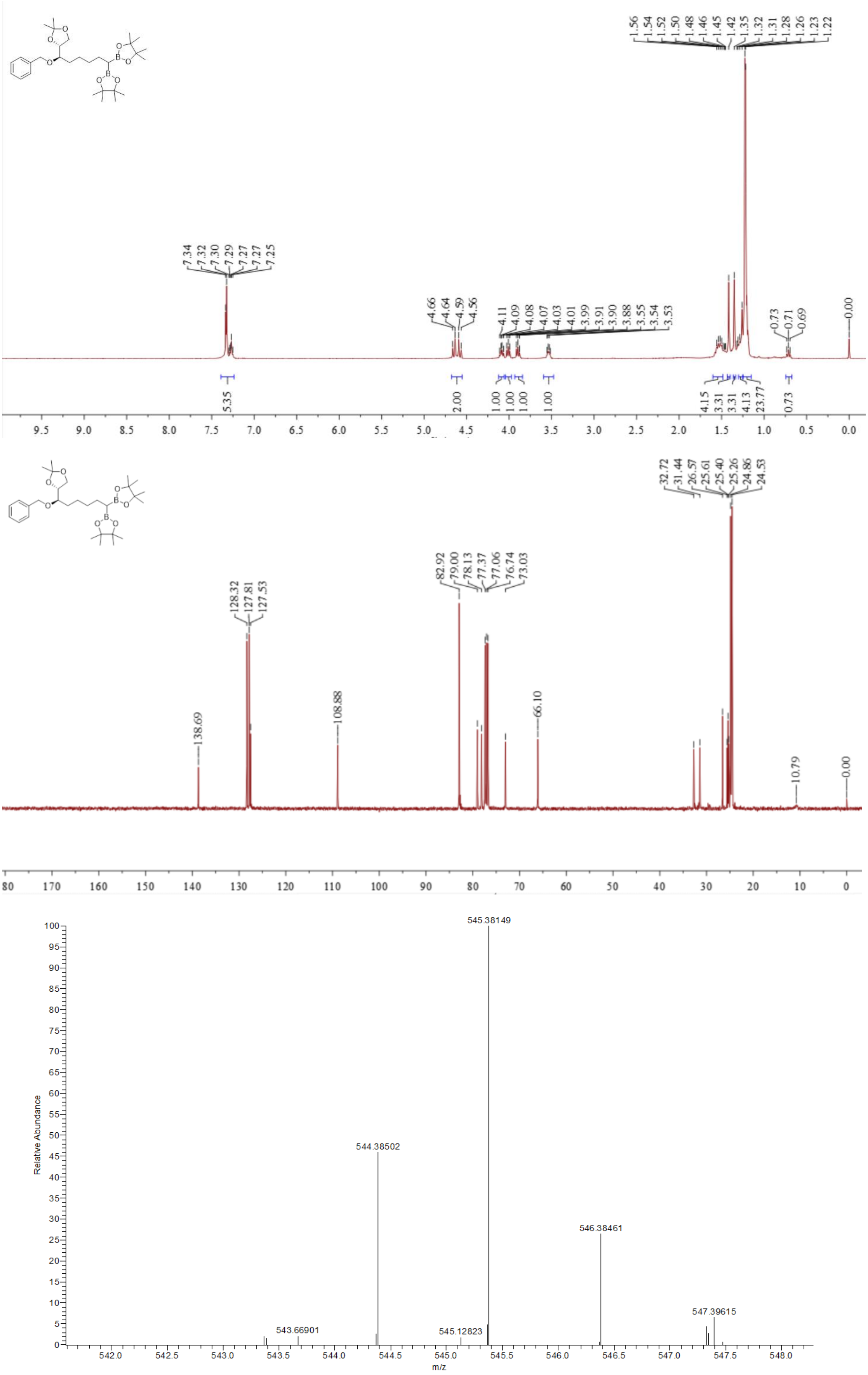

Supplementary Figure 33. ^{1}**H,** ^{13}**C NMR and HRMS of compound 20**

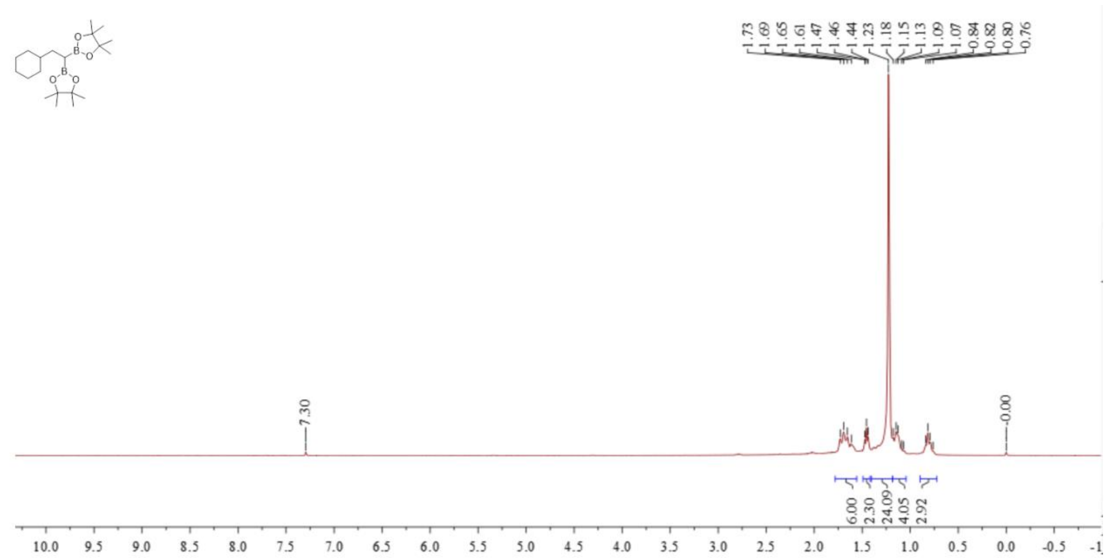

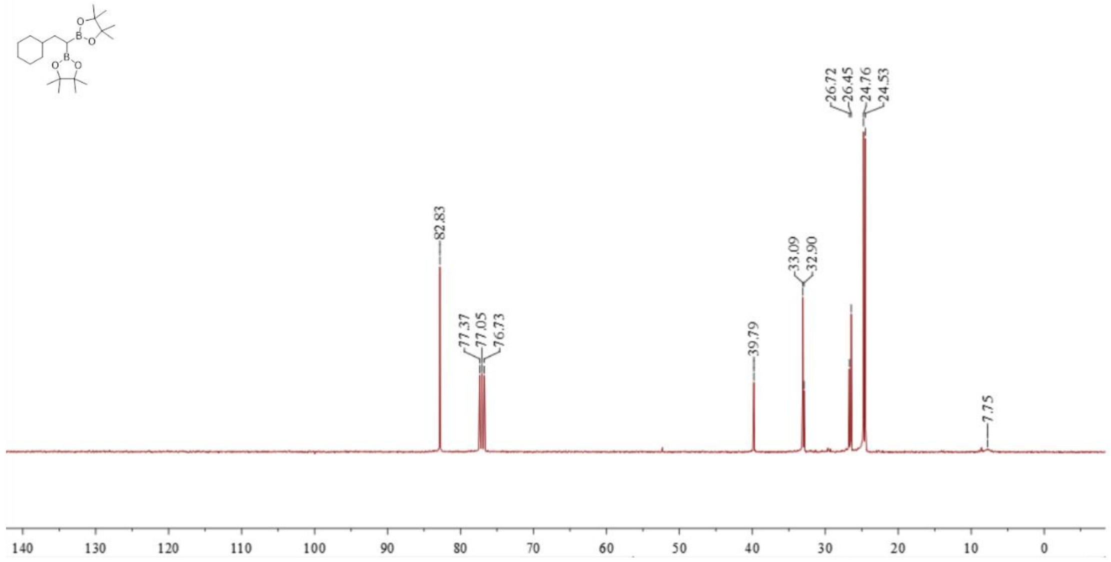

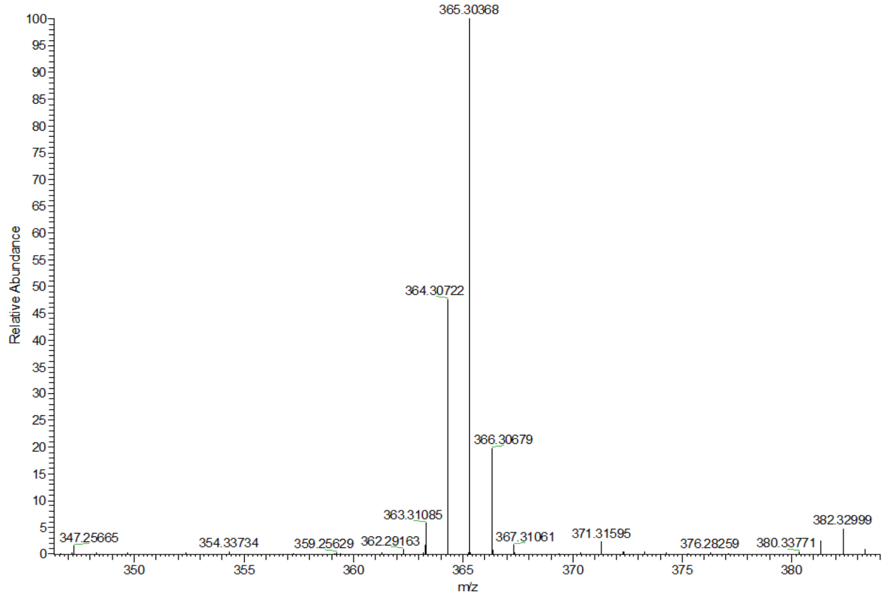

Supplementary Figure 34. **^{1}H, ^{13}C NMR and HRMS of compound 21**

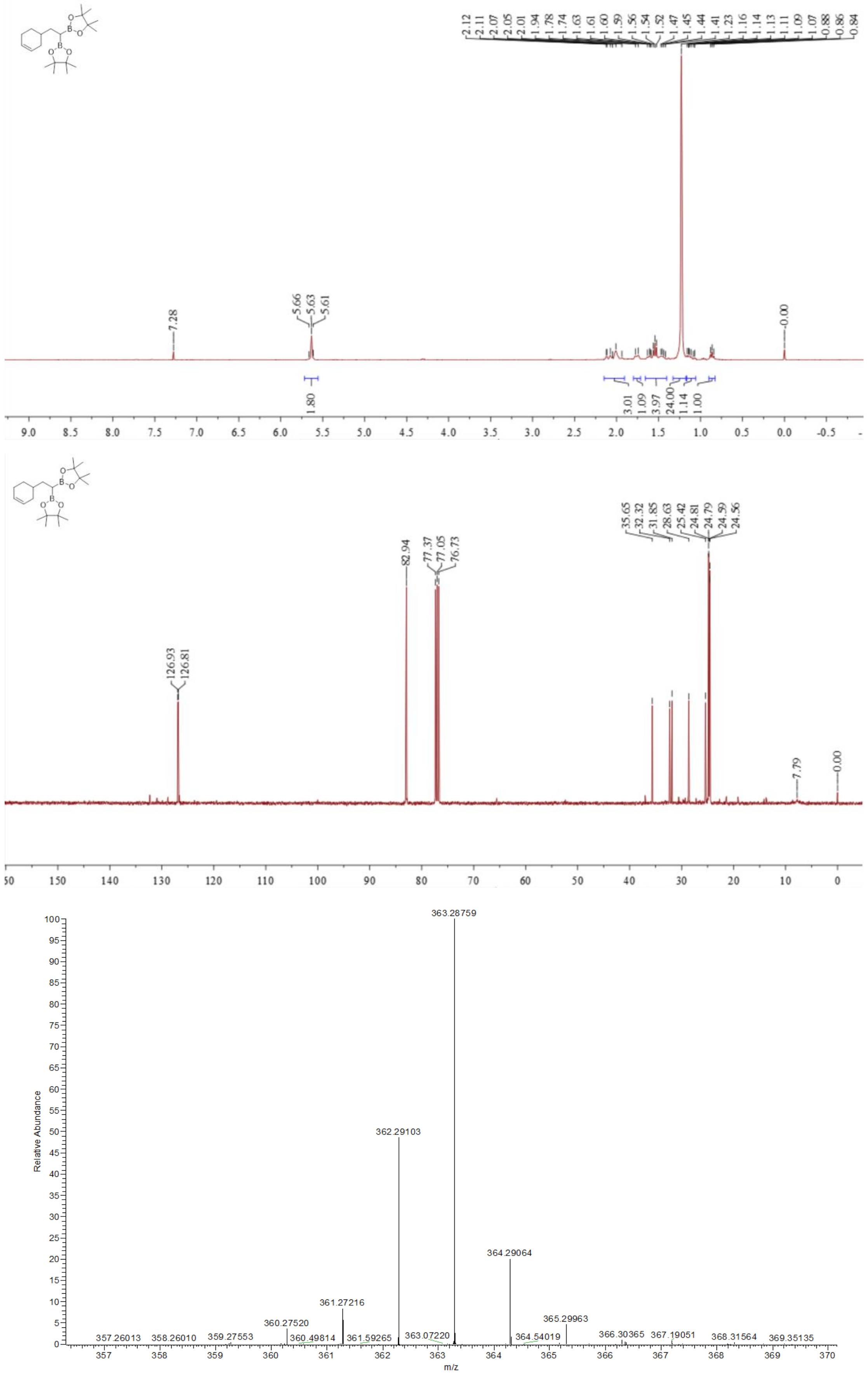

Supplementary Figure 35. ^{1}H, ^{13}C NMR and HRMS of compound 22

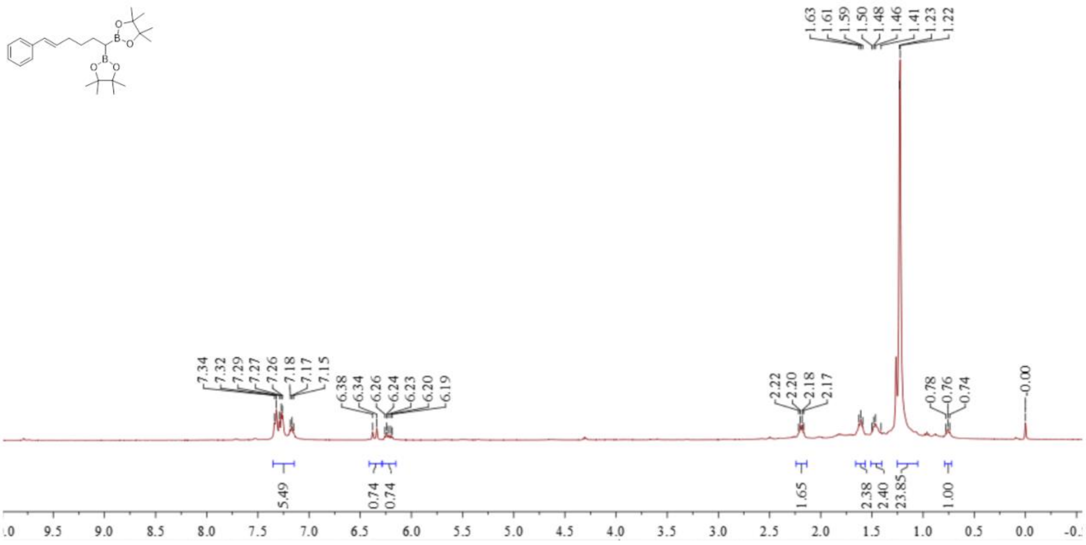

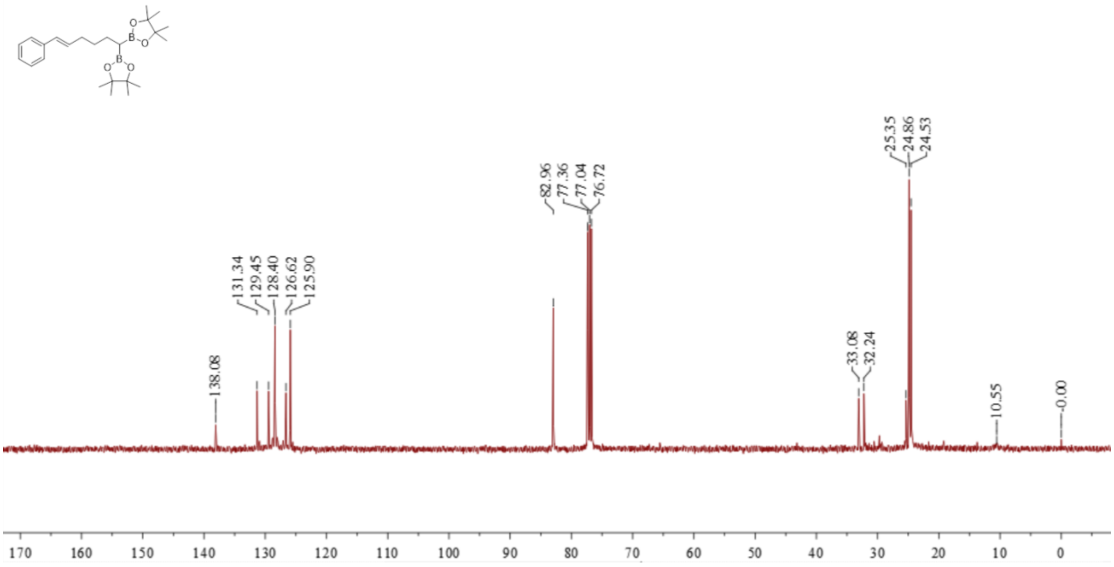

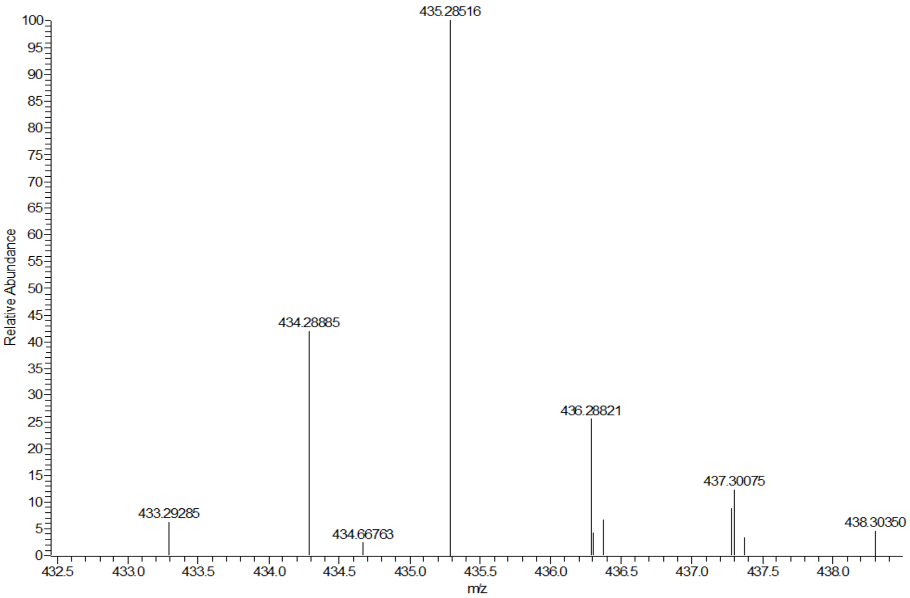

Supplementary Figure 36. ^{1}H, ^{13}C NMR and HRMS of compound 23

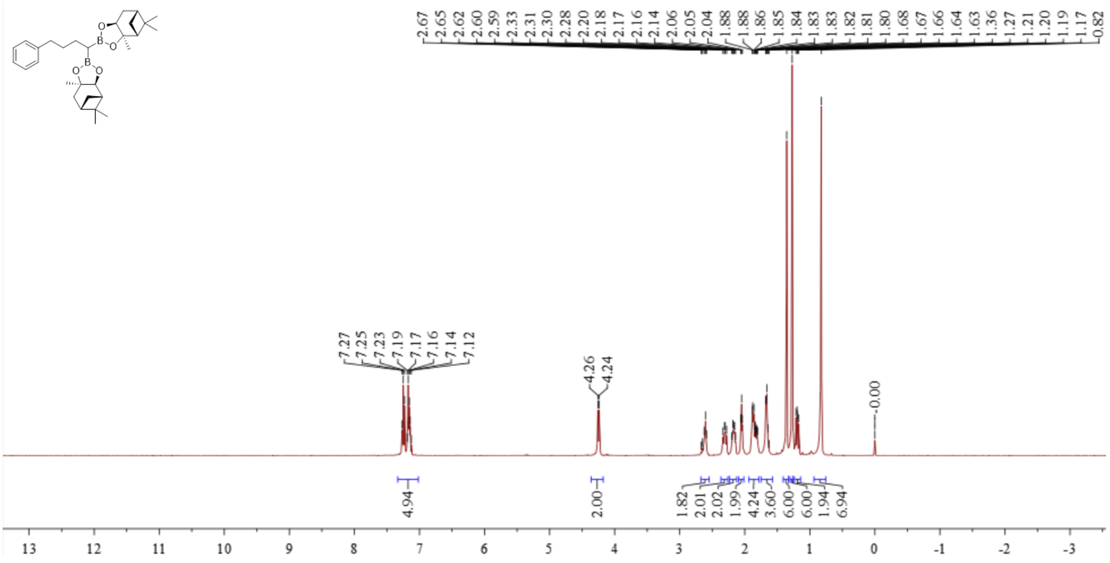

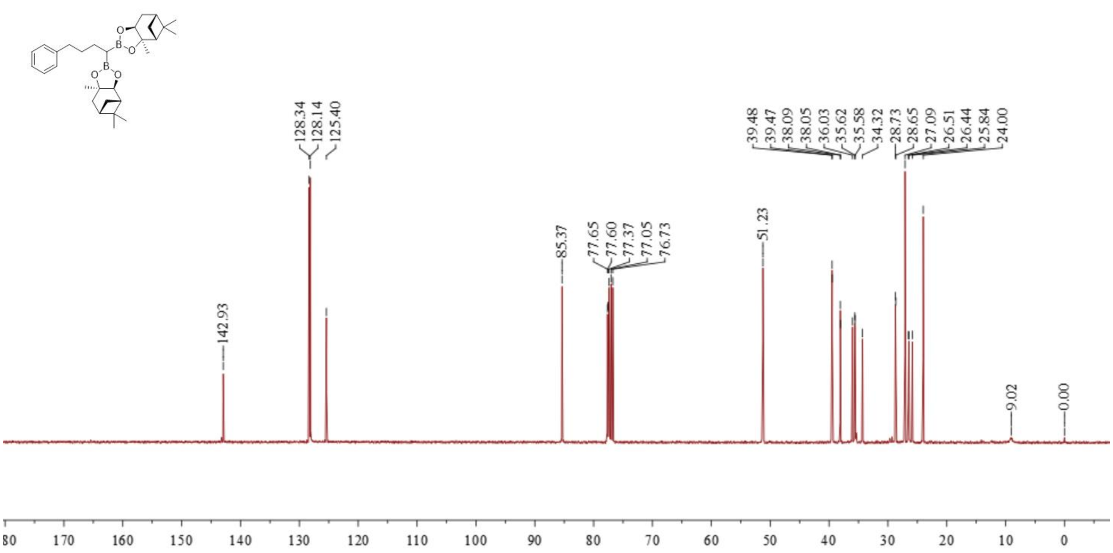

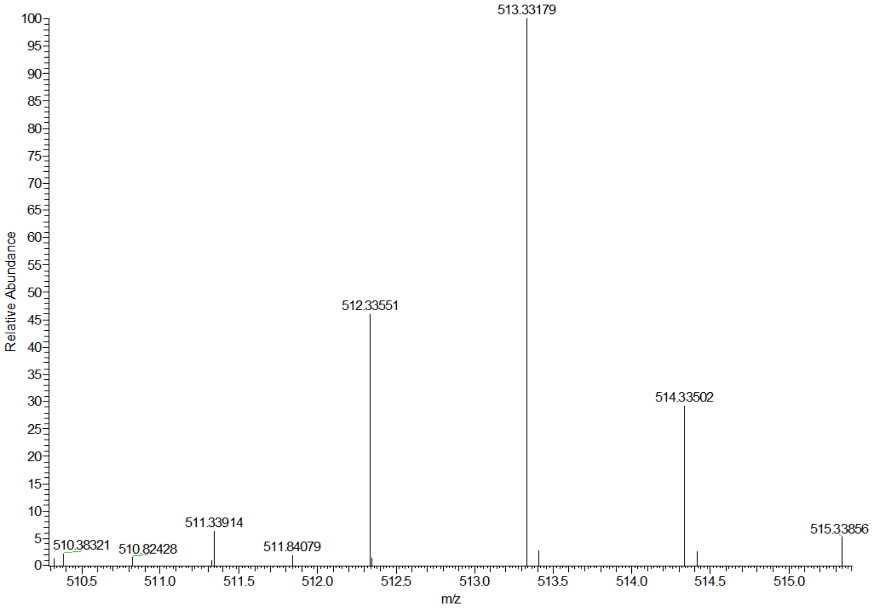

Supplementary Figure 37. ^{1}H, ^{13}C NMR and HRMS of compound 24

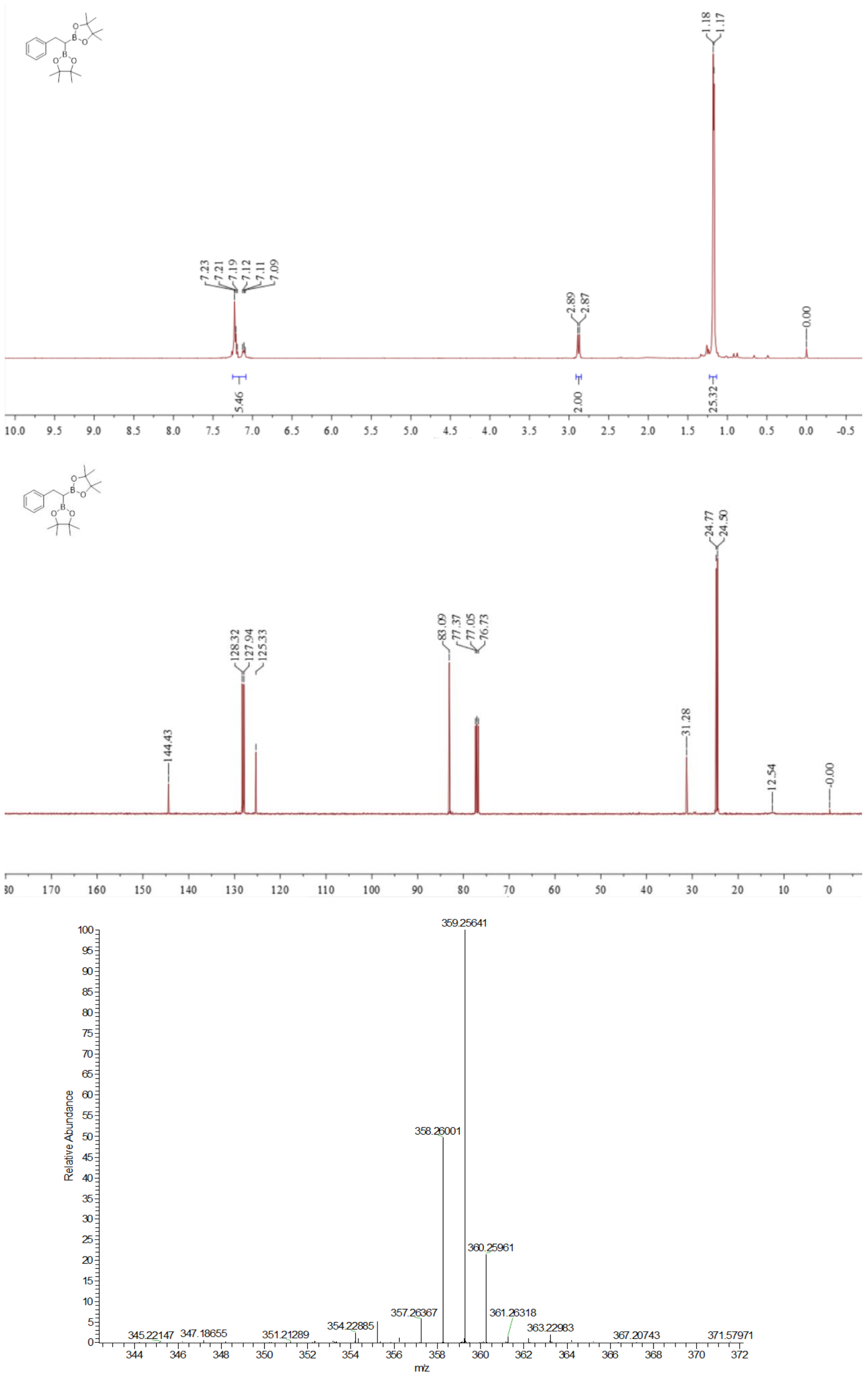

Supplementary Figure 38. ^{1}**H,** ^{13}**C NMR and HRMS of compound 26**

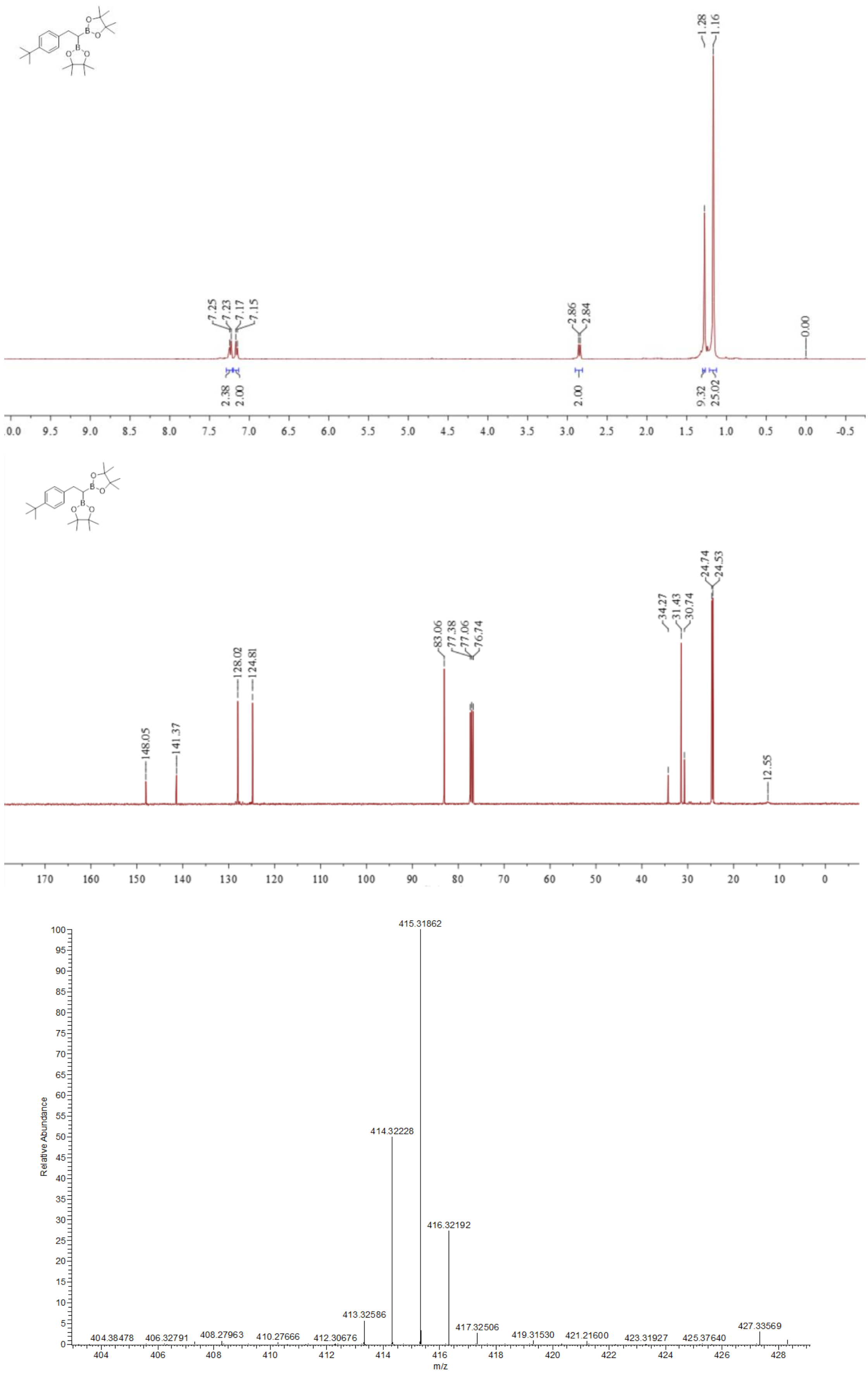

Supplementary Figure 39. ^{1}H, ^{13}C NMR and HRMS of compound 27

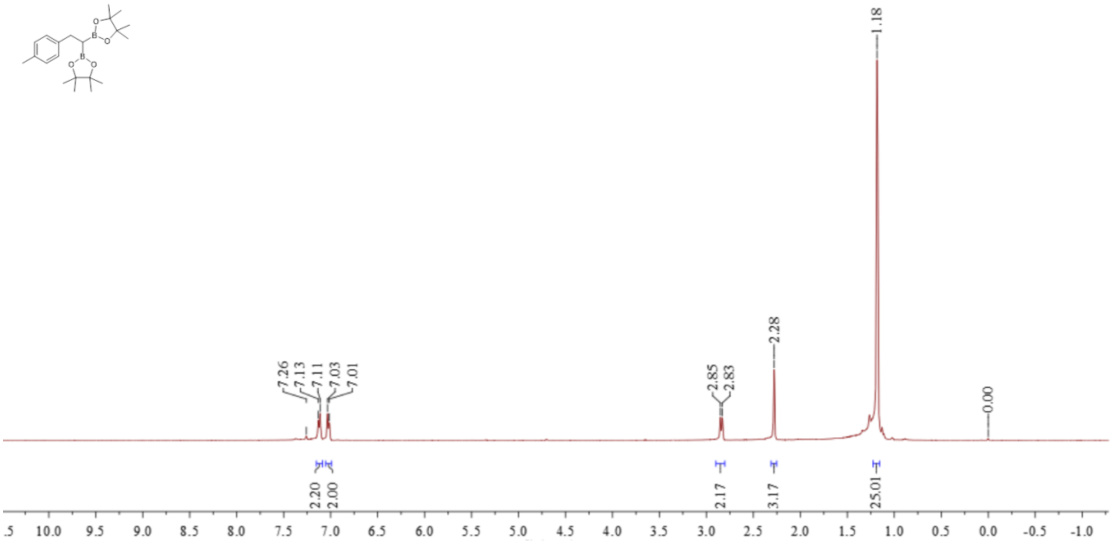

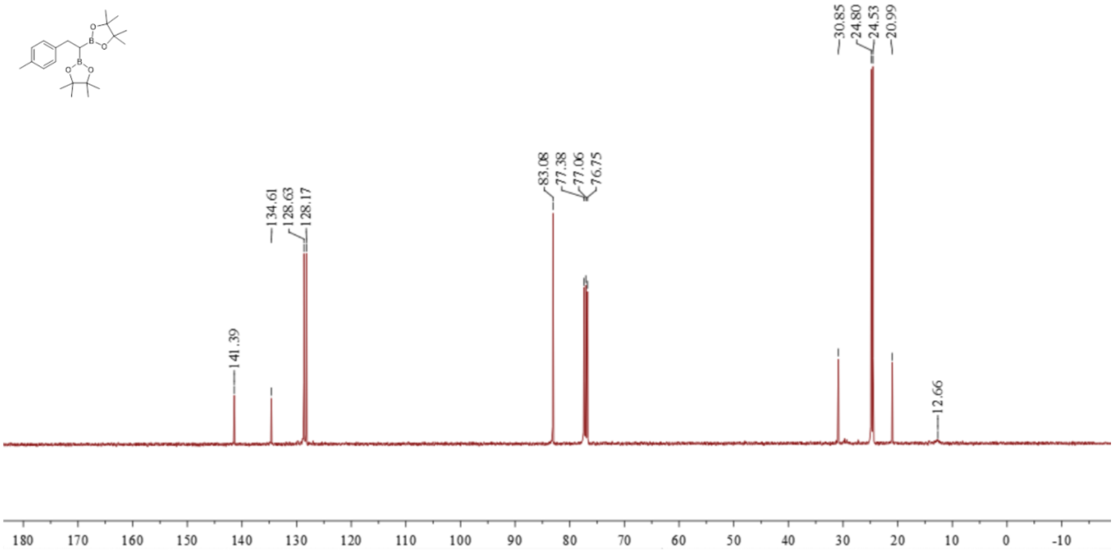

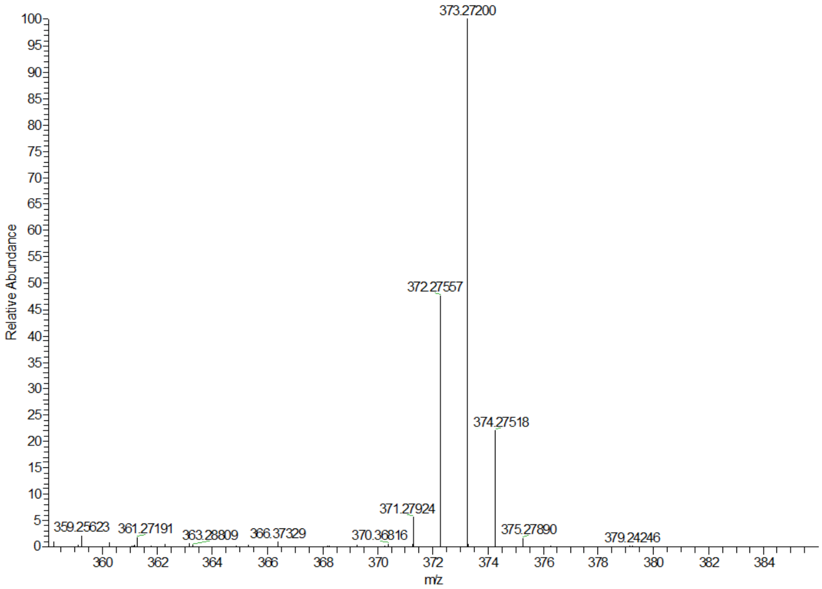

Supplementary Figure 40. ^{1}H, ^{13}C NMR and HRMS of compound 28

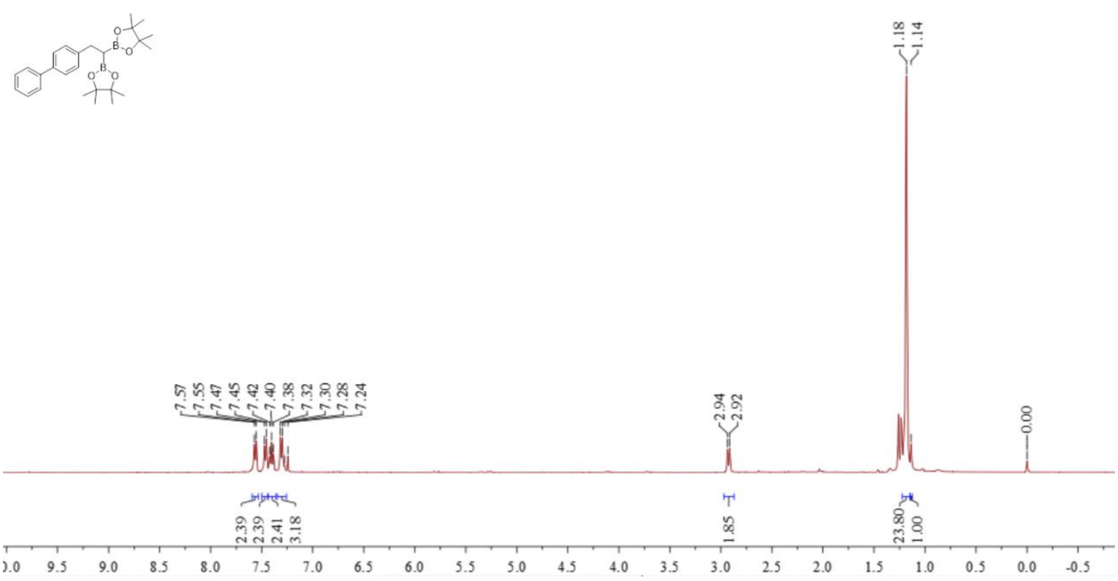

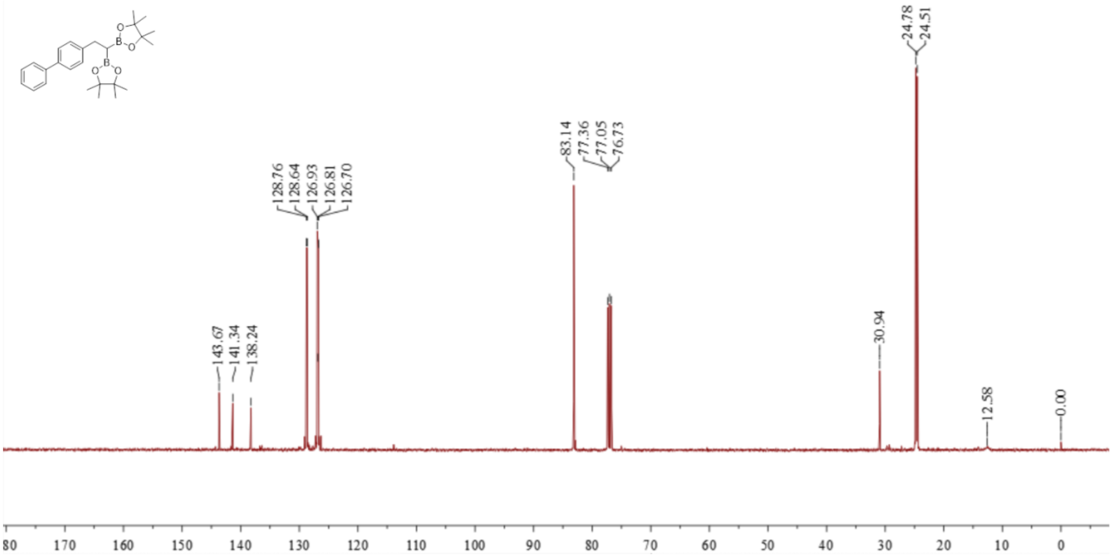

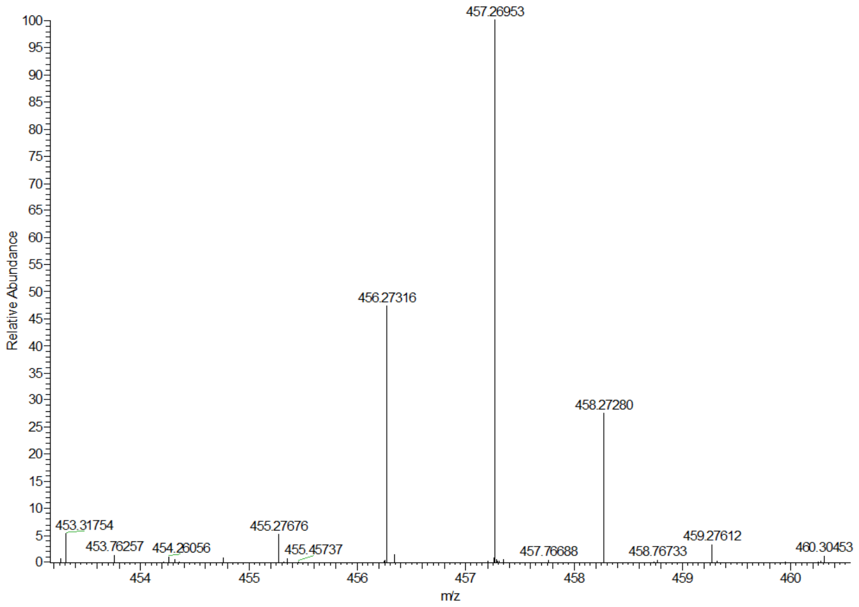

Supplementary Figure 41. ^{1}**H,** ^{13}**C NMR and HRMS of compound 29**

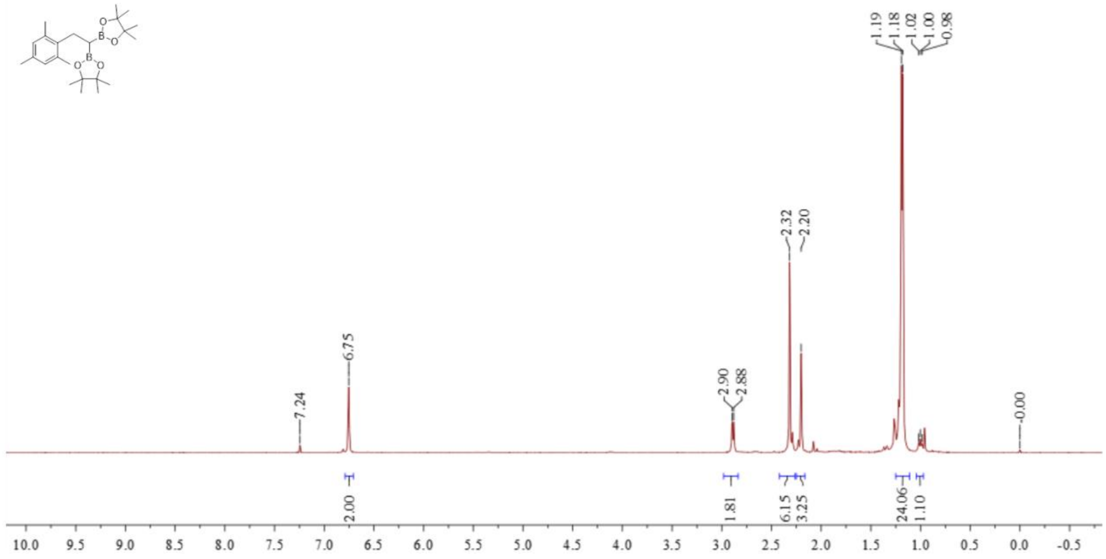

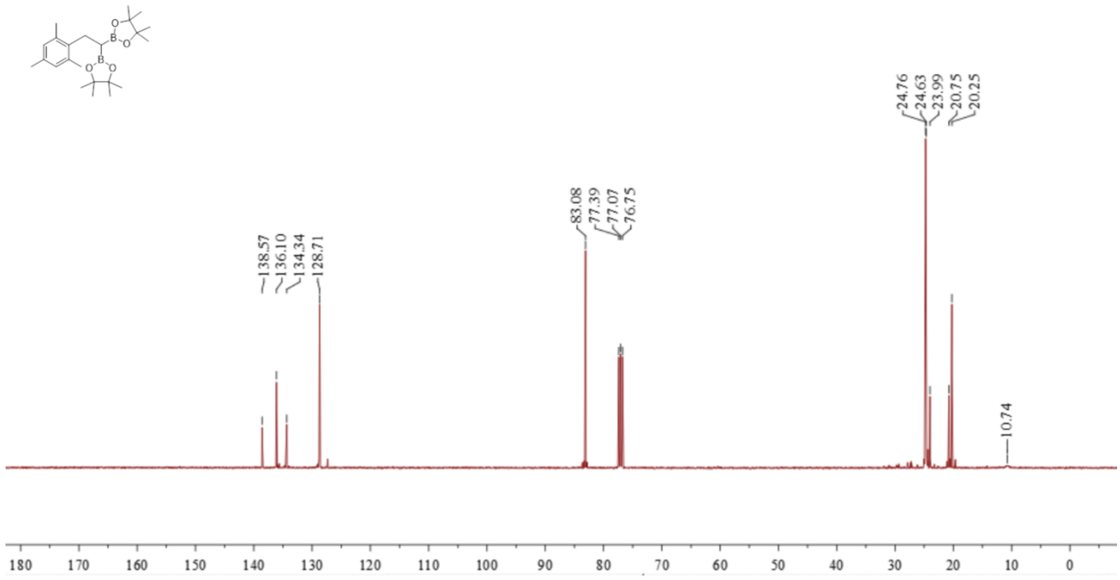

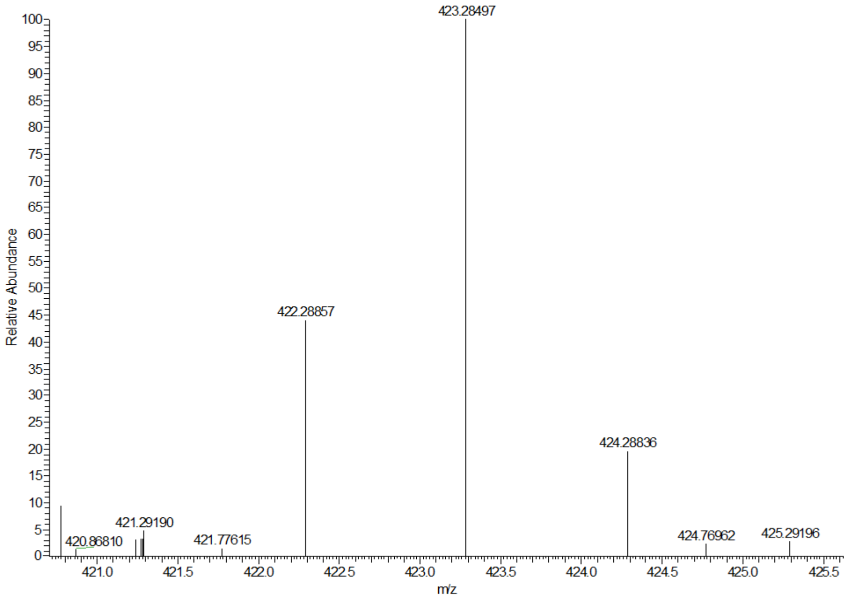

Supplementary Figure 42. ^{1}H, ^{13}C NMR and HRMS of compound 30

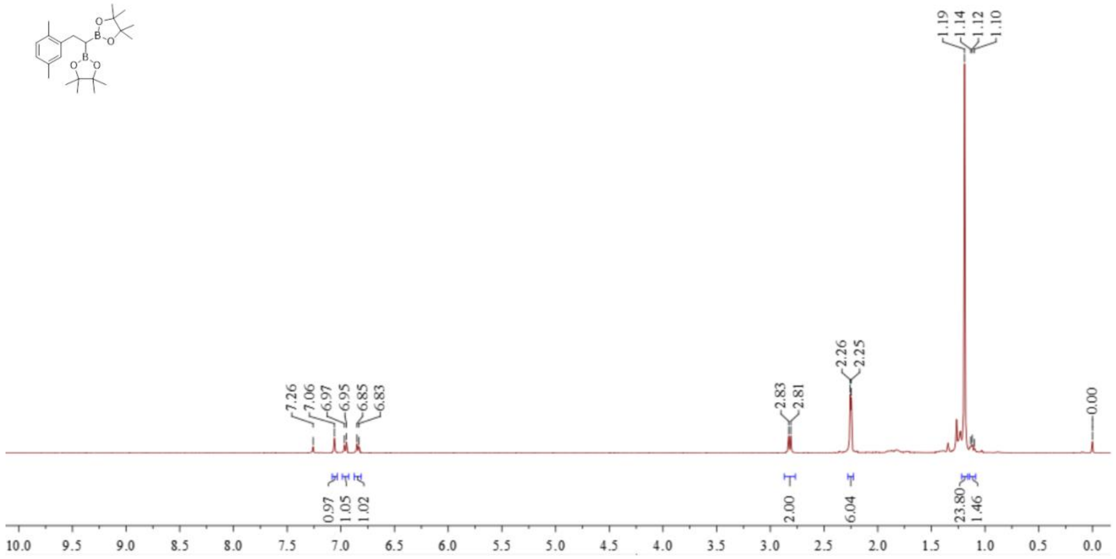

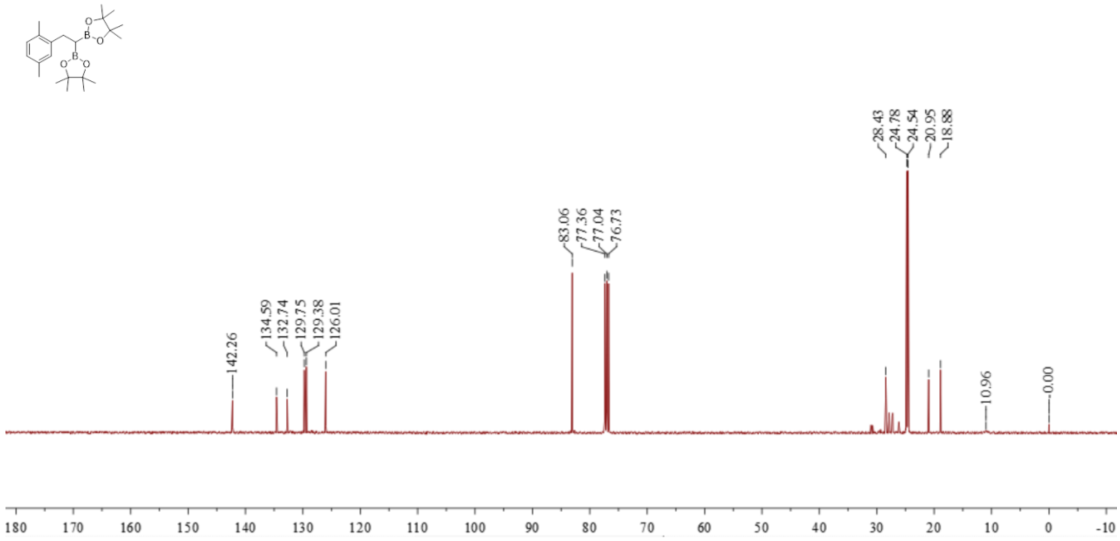

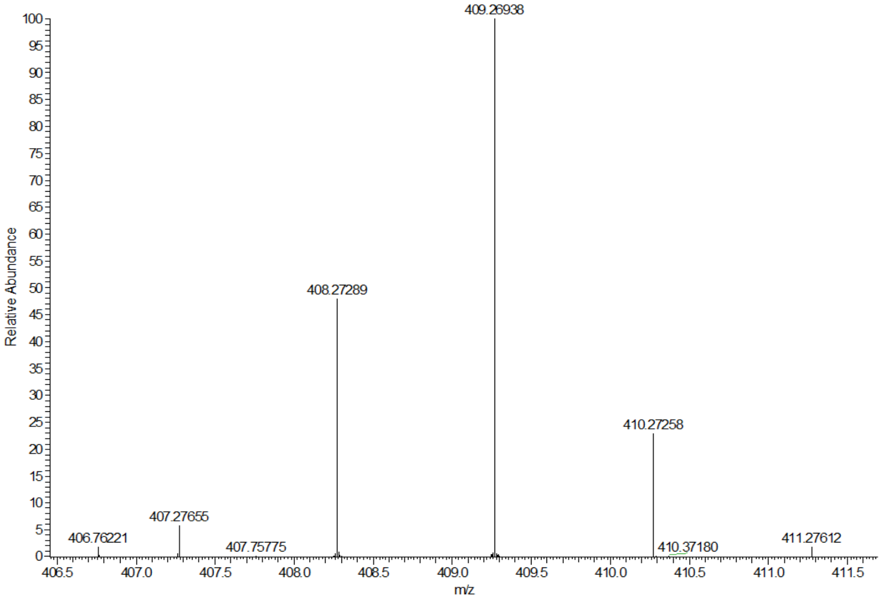

Supplementary Figure 43. ^{1}H, ^{13}C NMR and HRMS of compound 31

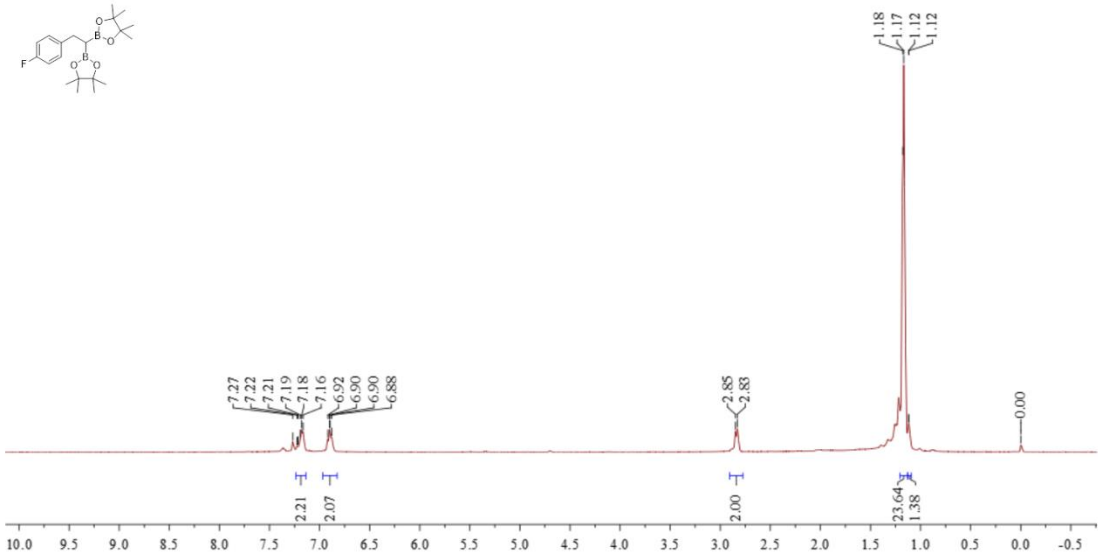
7.27 7.22 7.21 7.19 7.18 7.16 6.92 6.90 6.90 6.88
2.85 2.83
1.18 1.17 1.12 1.12
0.00
2.21 2.07 2.00 23.64 1.38

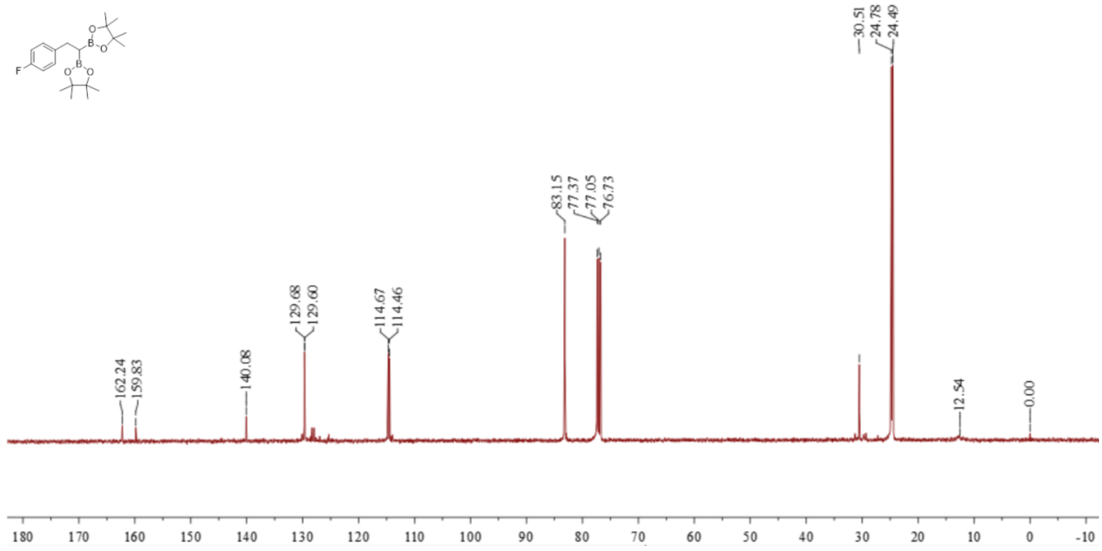
162.24 159.83 140.08 129.68 129.60 114.67 114.46
83.15 77.37 77.05 76.73
30.51 24.78 24.49 12.54 0.00

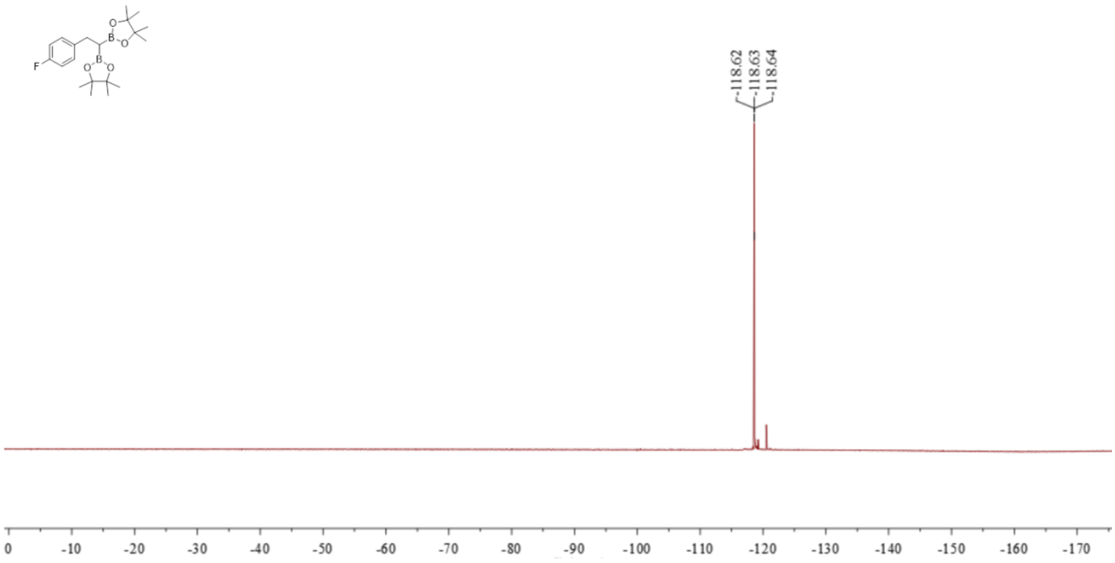
-118.62 -118.63 -118.64

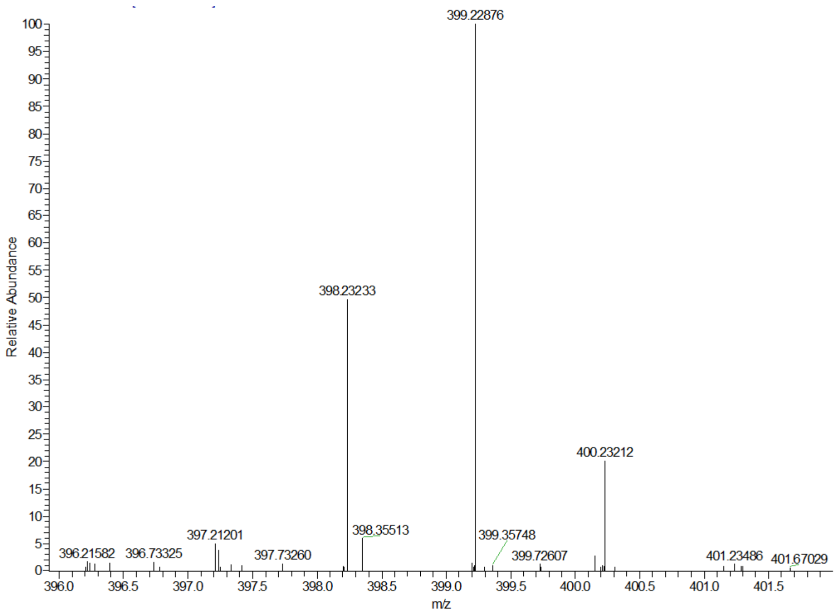

Supplementary Figure 44. ^{1}H, ^{19}F, ^{13}C NMR and HRMS of compound 32

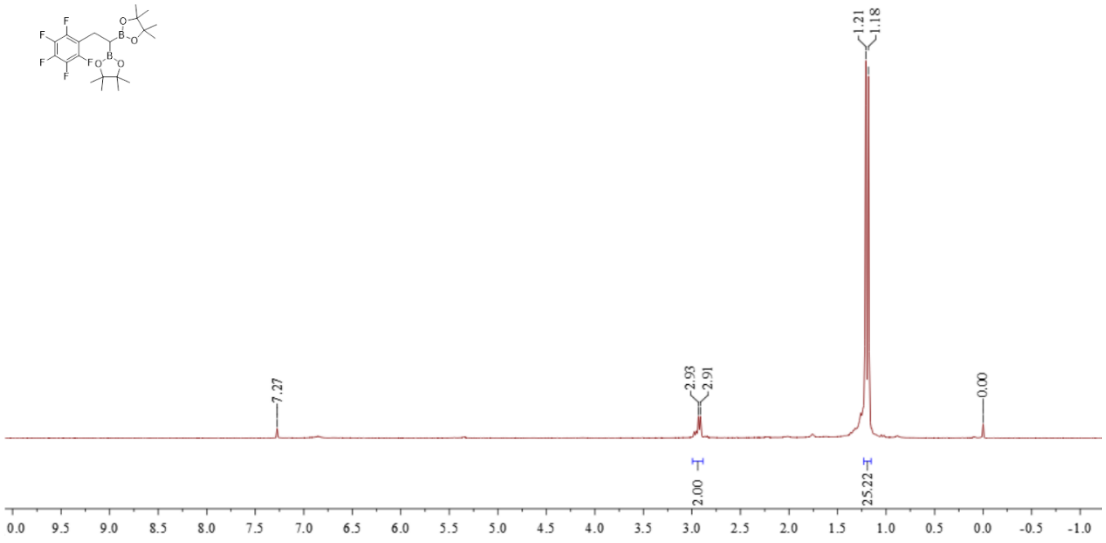

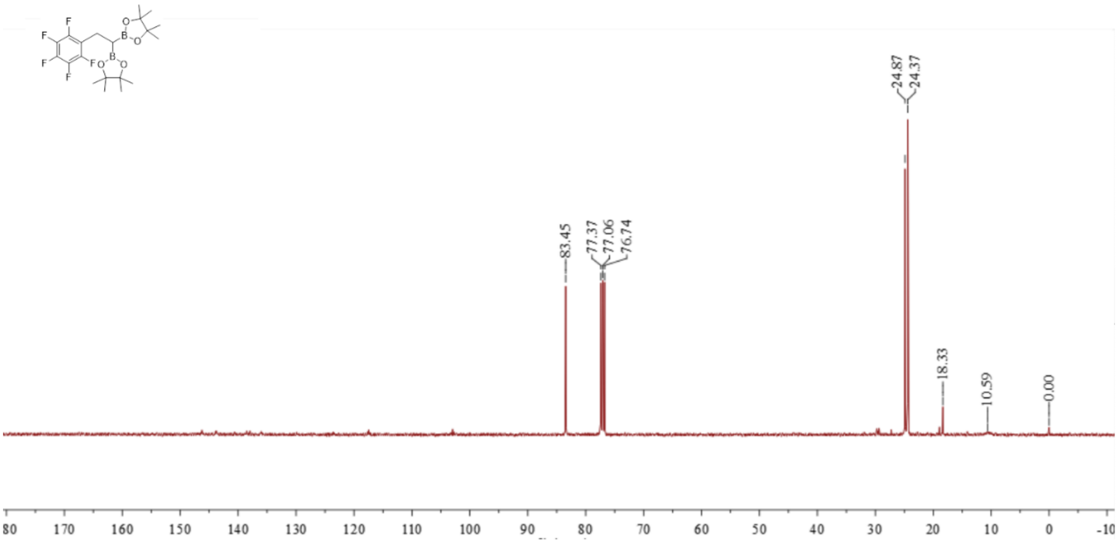

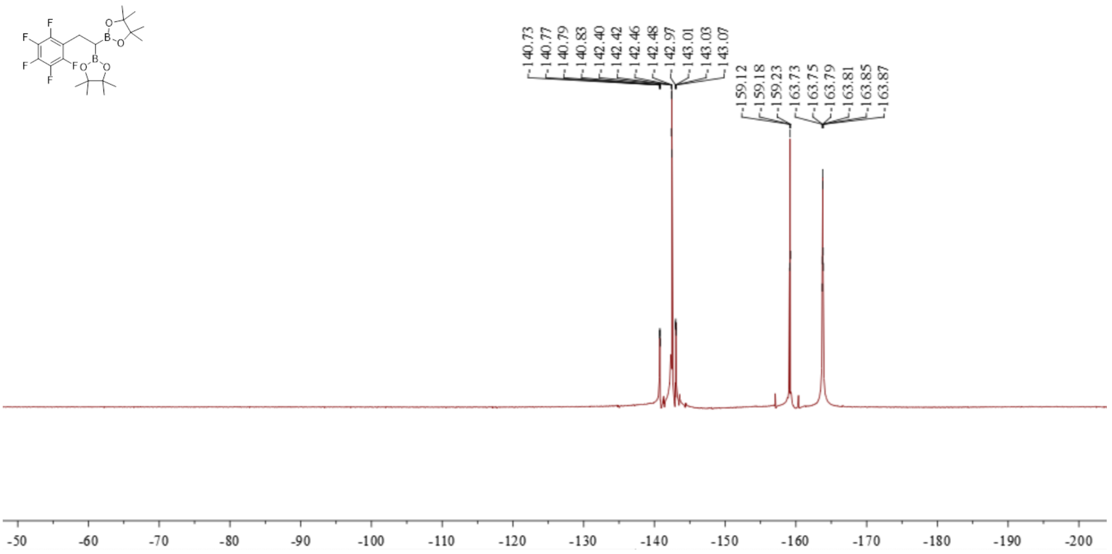

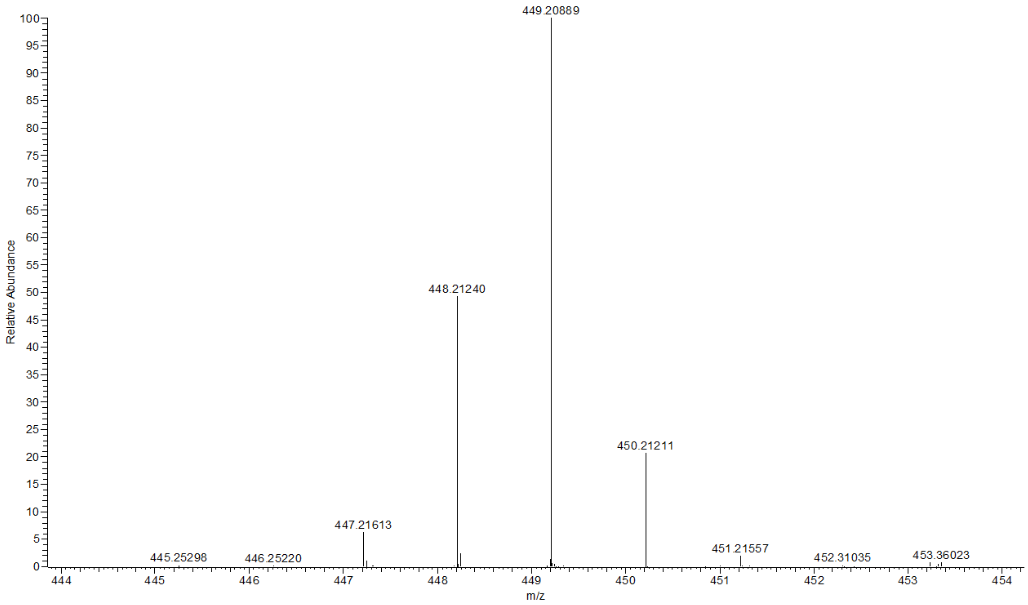

Supplementary Figure 45. ^{1}H, ^{19}F, ^{13}C NMR and HRMS of compound 33

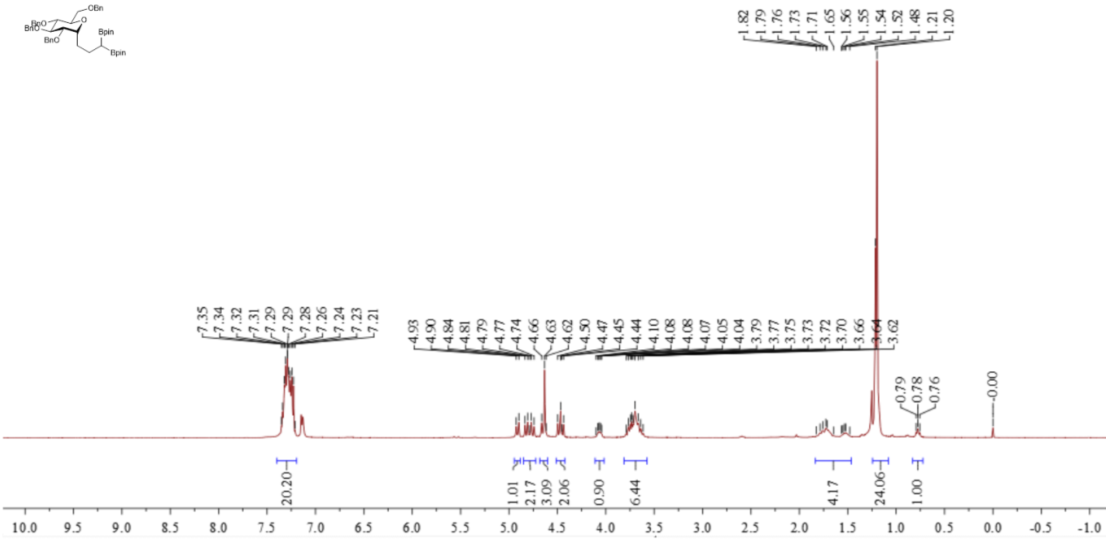

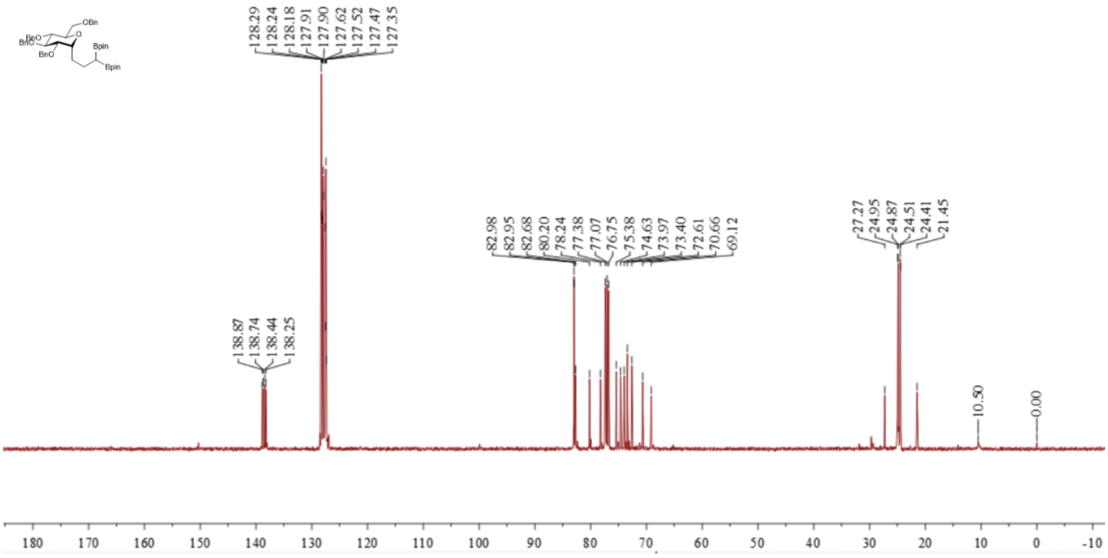

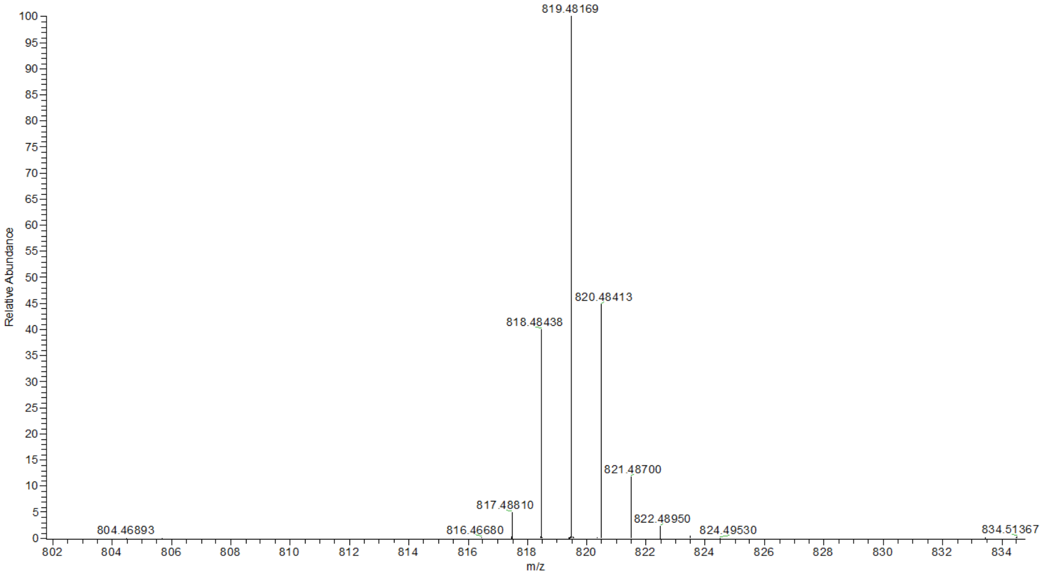

Supplementary Figure 46. **^{1}H, ^{13}C NMR and HRMS of compound 36**

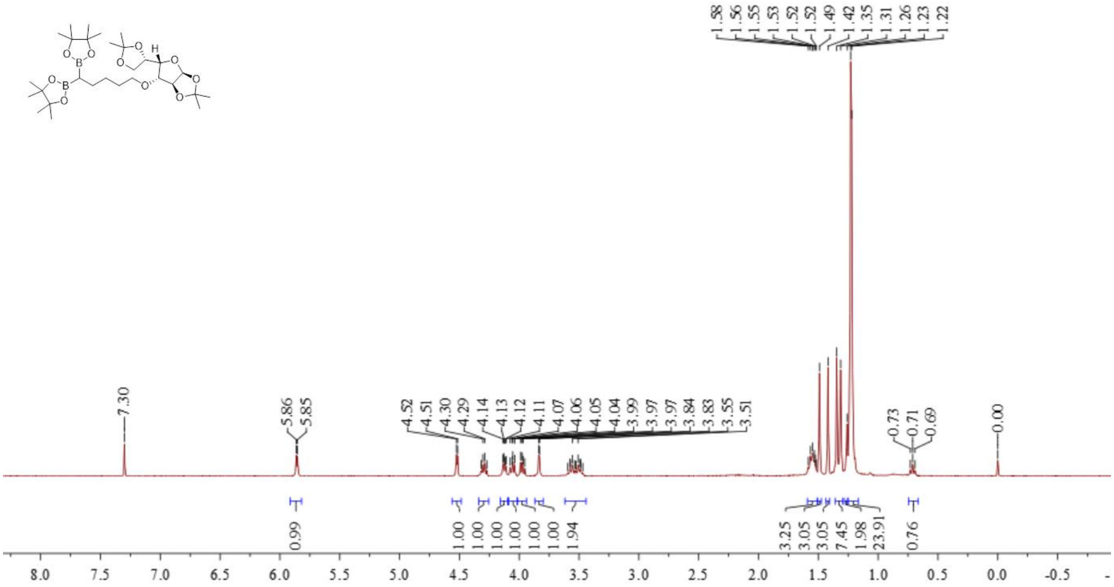

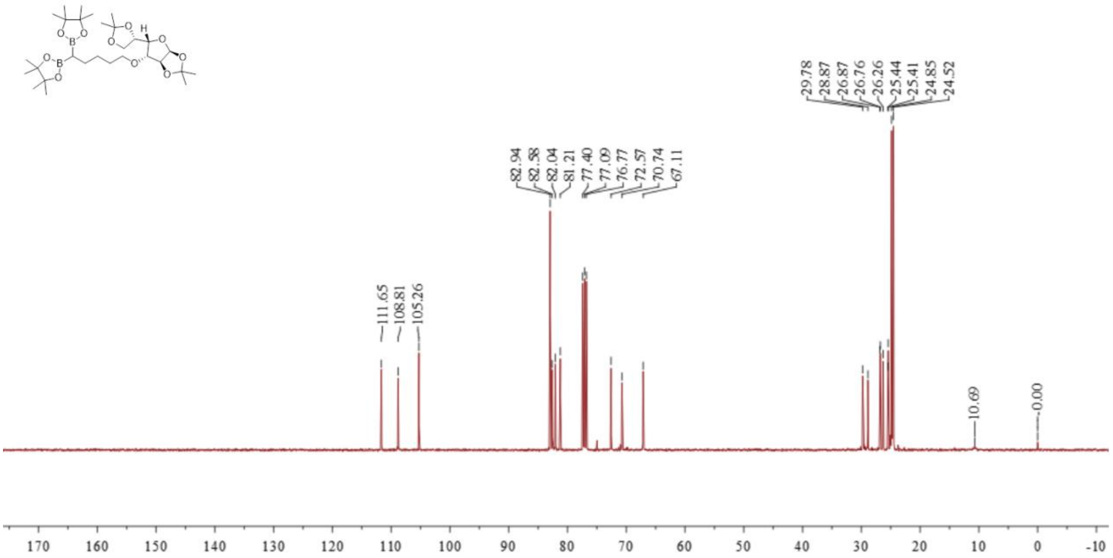

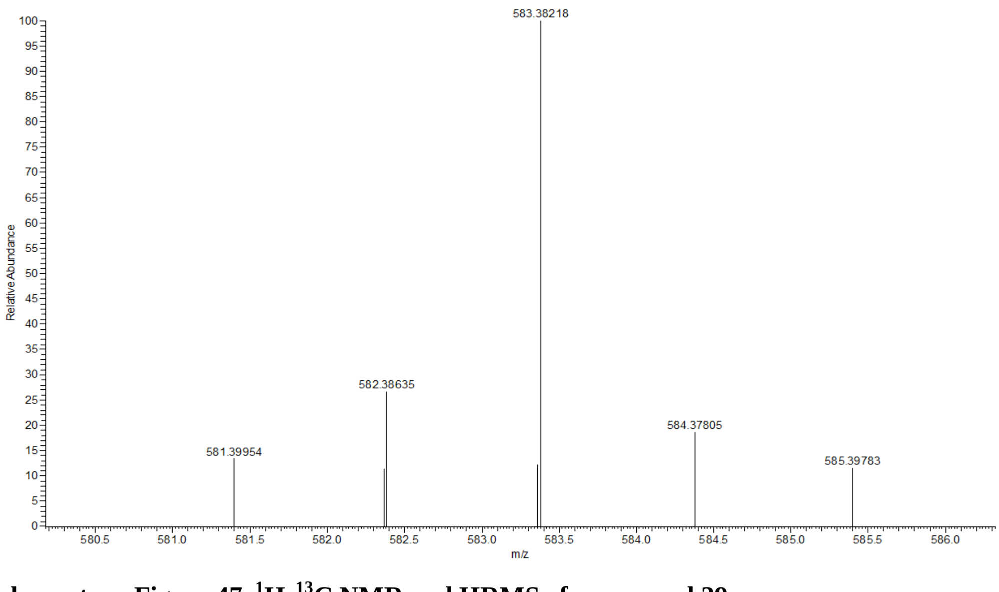

Supplementary Figure 47. ^{1}H, ^{13}C NMR and HRMS of compound 39

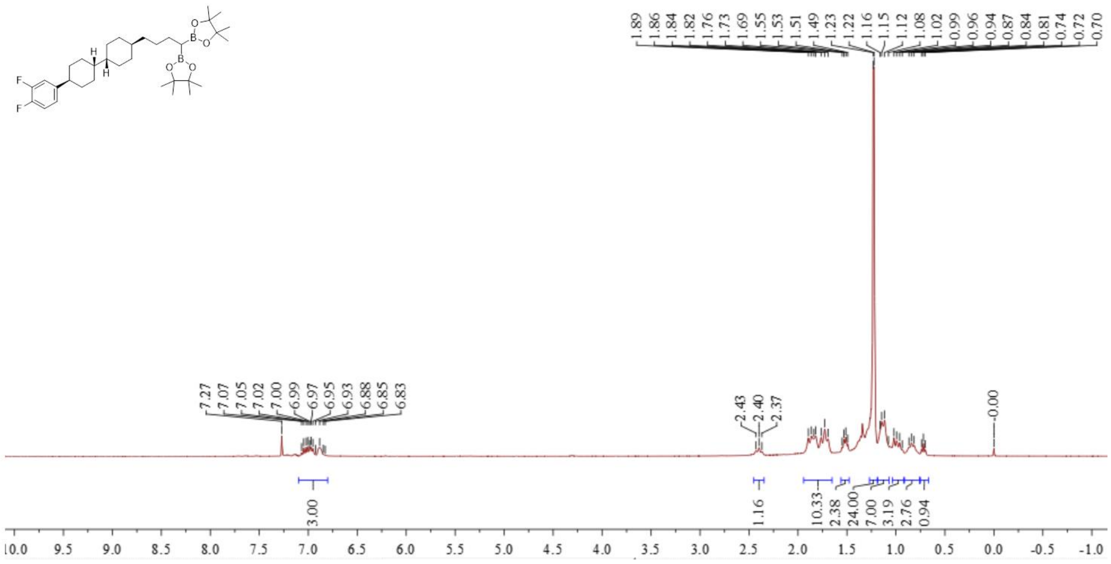

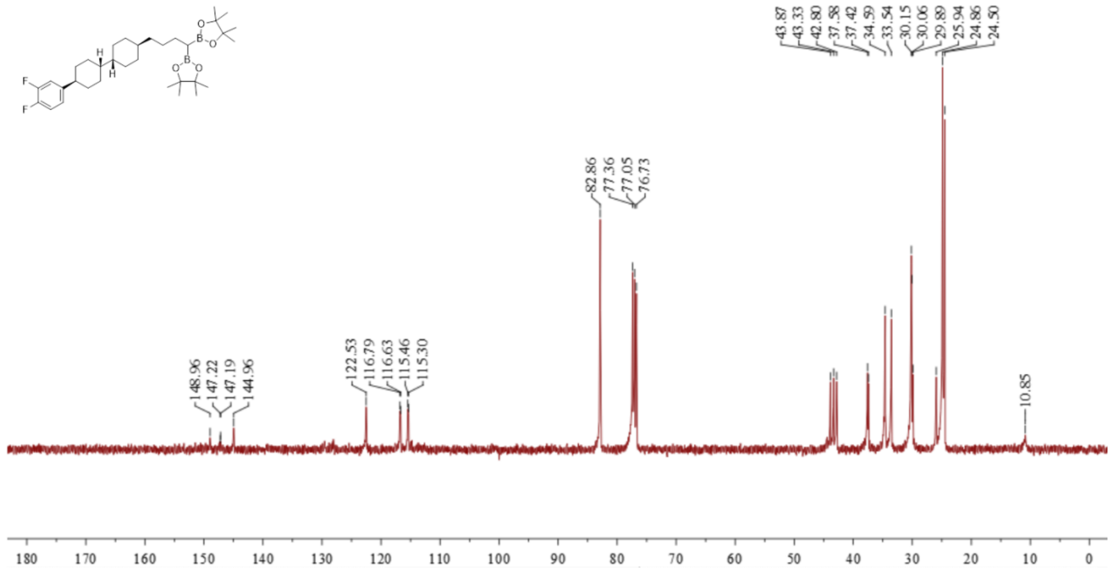

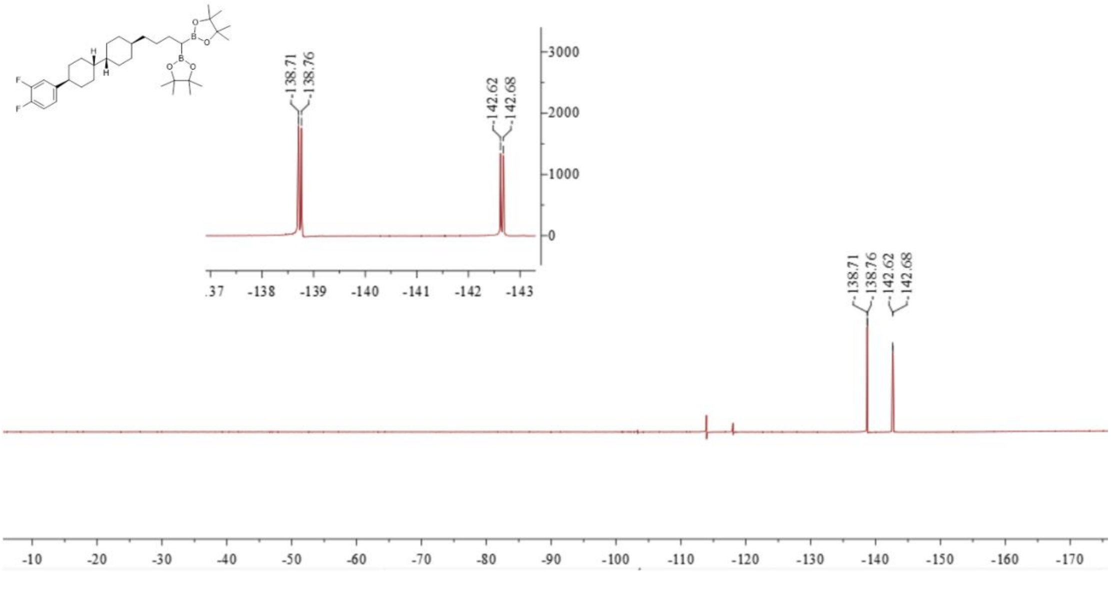

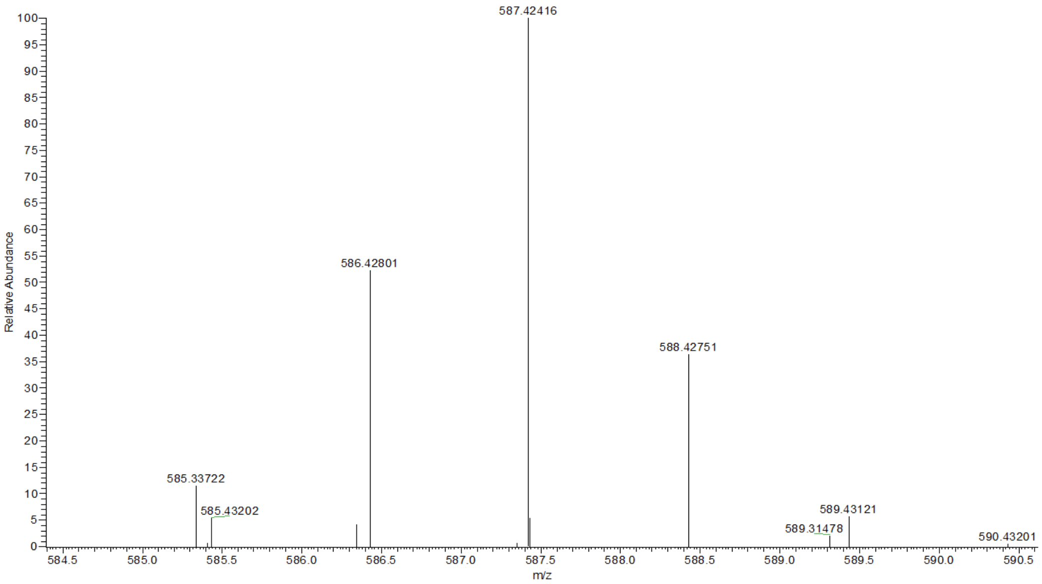

Supplementary Figure 48. ^{1}H, ^{13}C, ^{19}F NMR and HRMS of compound 41

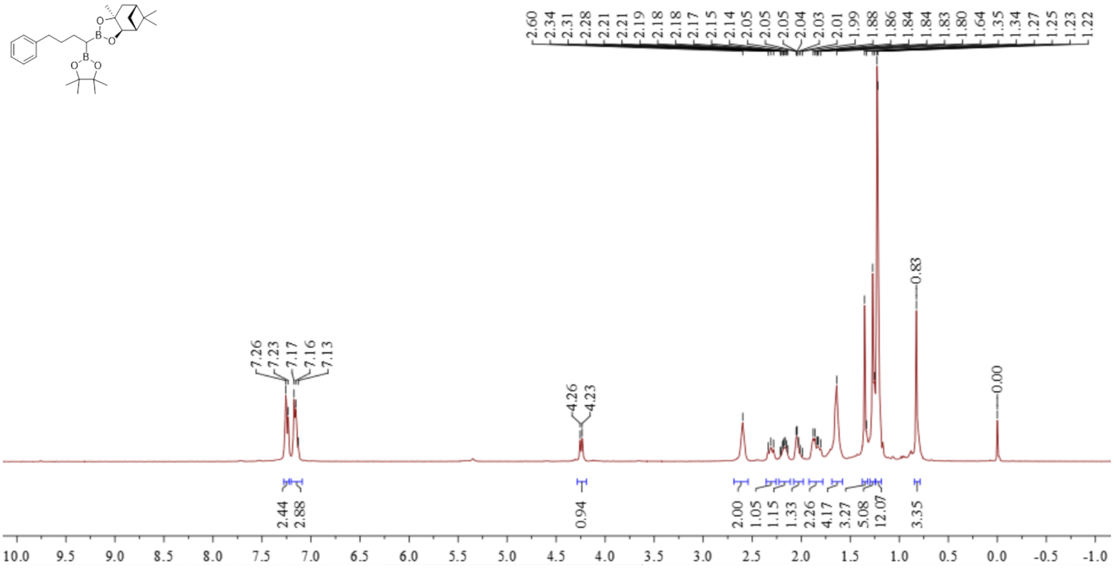

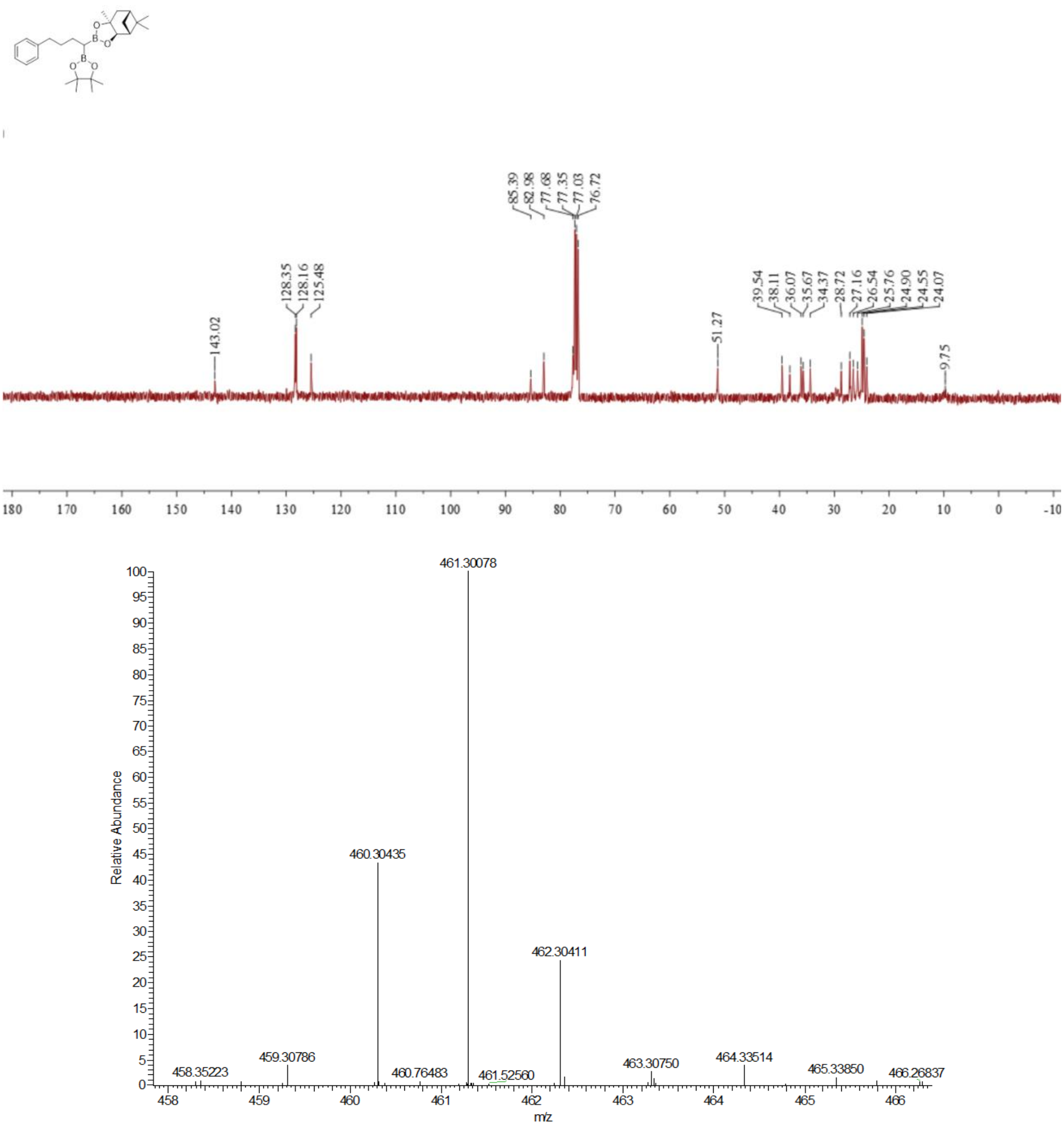

Supplementary Figure 49. ^{1}H, ^{13}C NMR and HRMS of compound 48

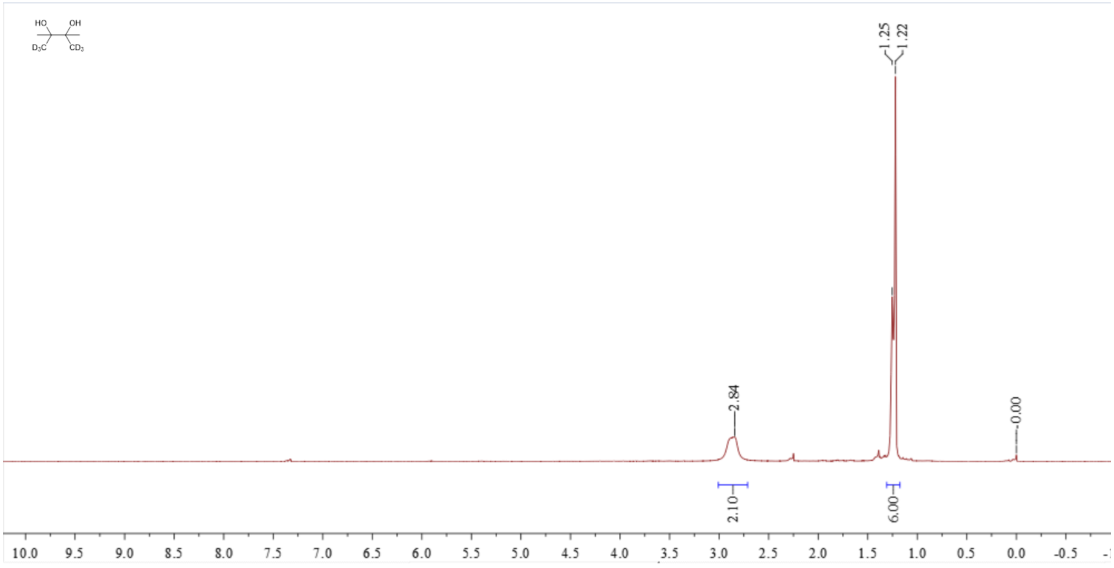

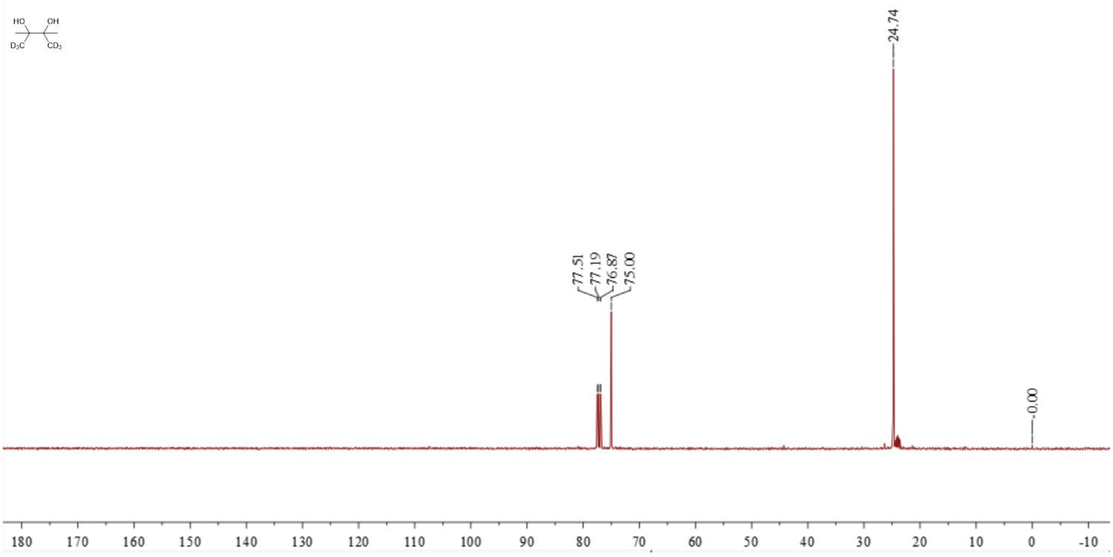

Supplementary Figure 50. ^{1}H and ^{13}C NMR of 2,3-dimethylbutane-1,1,1,4,4,4-*d*$_6$-2,3-diol

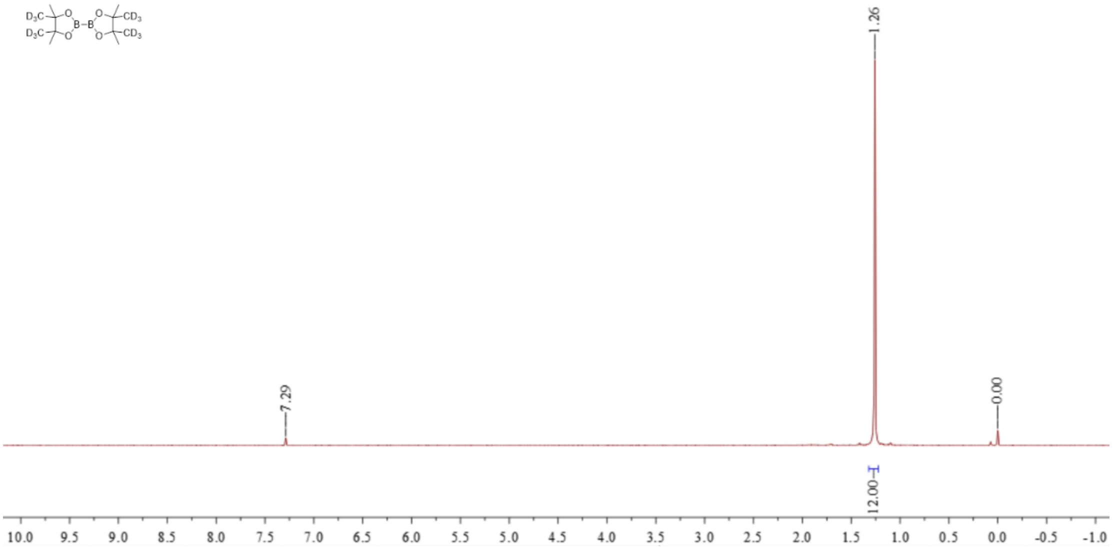

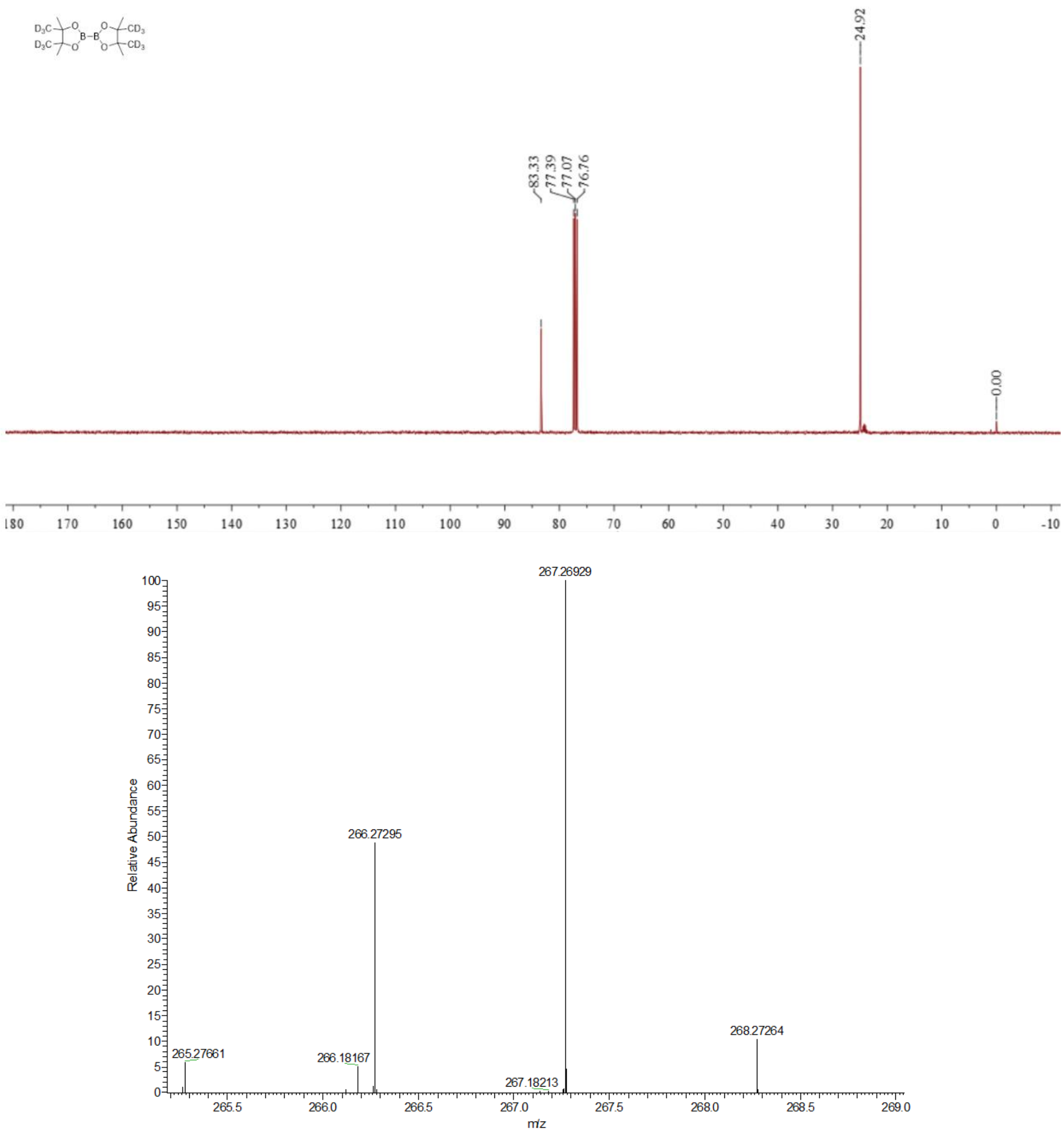

Supplementary Figure 51. ^{1}H, ^{13}C **NMR and HRMS of 4,4',5,5'-tetramethyl-4,4',5,5'-tetrakis(methyl-*d*$_3$)-2,2'-bi(1,3,2-dioxaborolane) (B$_2$pin$_2$-*d*$_{12}$)**

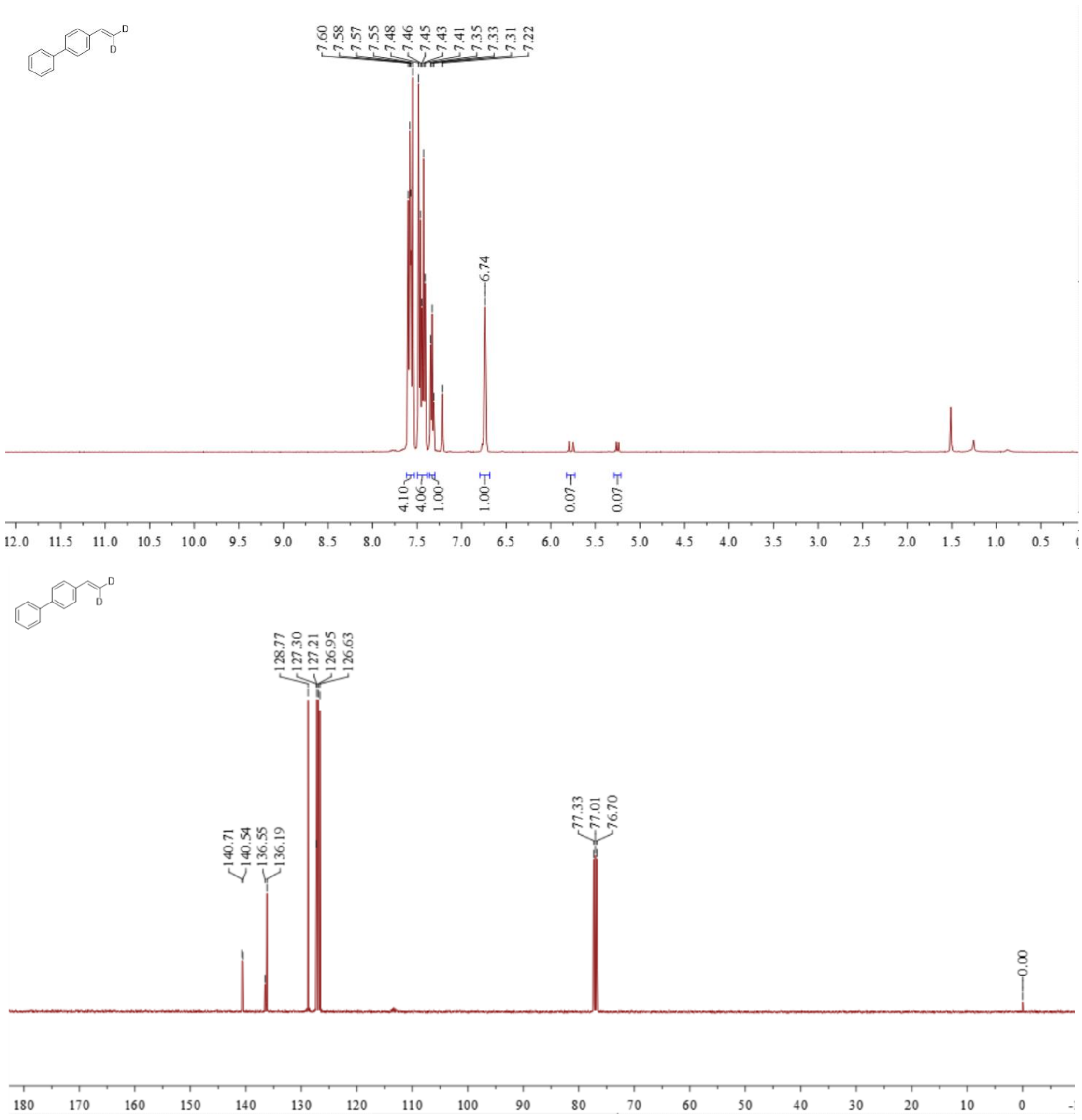

Supplementary Figure 52. ^{1}**H and** ^{13}**C NMR of compound 29a-*d*$_2$**

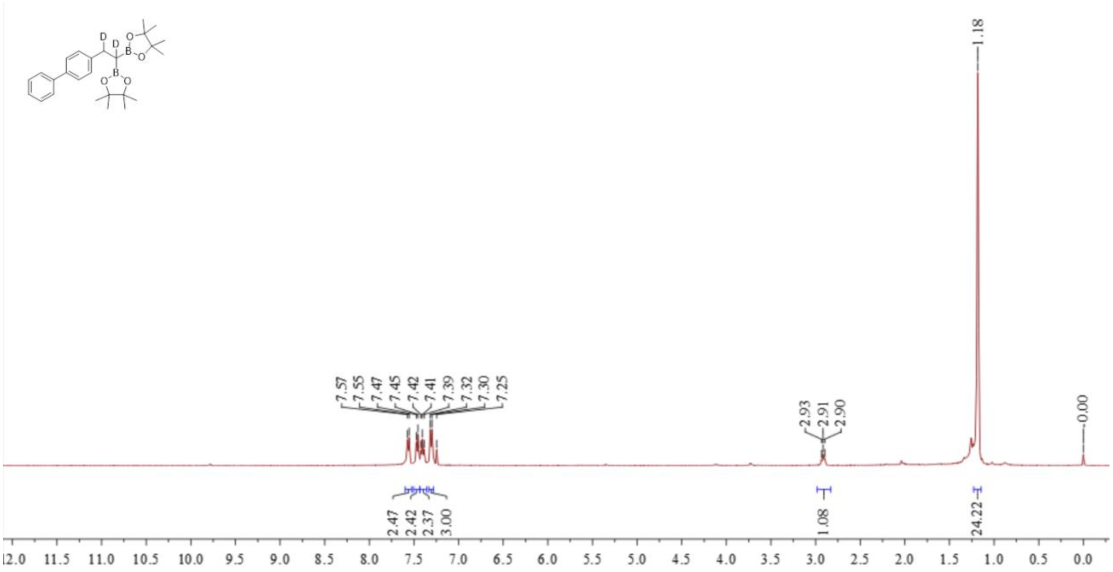

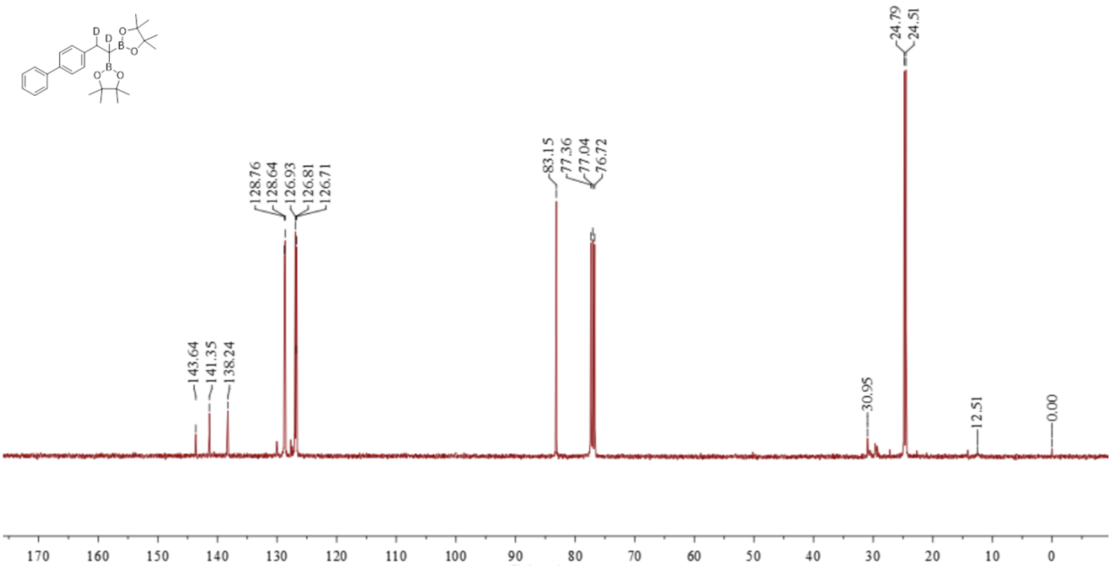

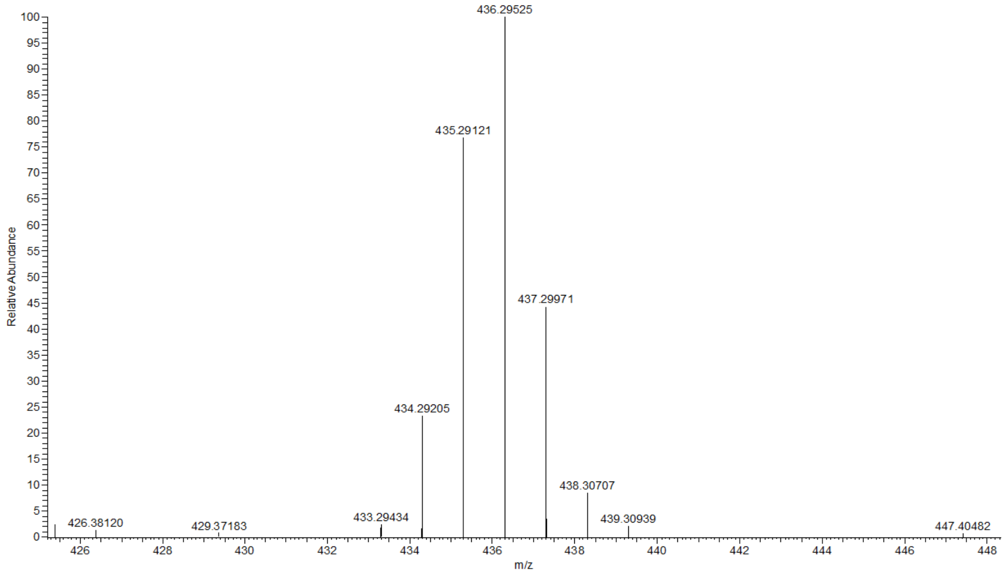

Supplementary Figure 53. ^{1}**H,** ^{13}**C NMR and HRMS of compound 29-d_2**

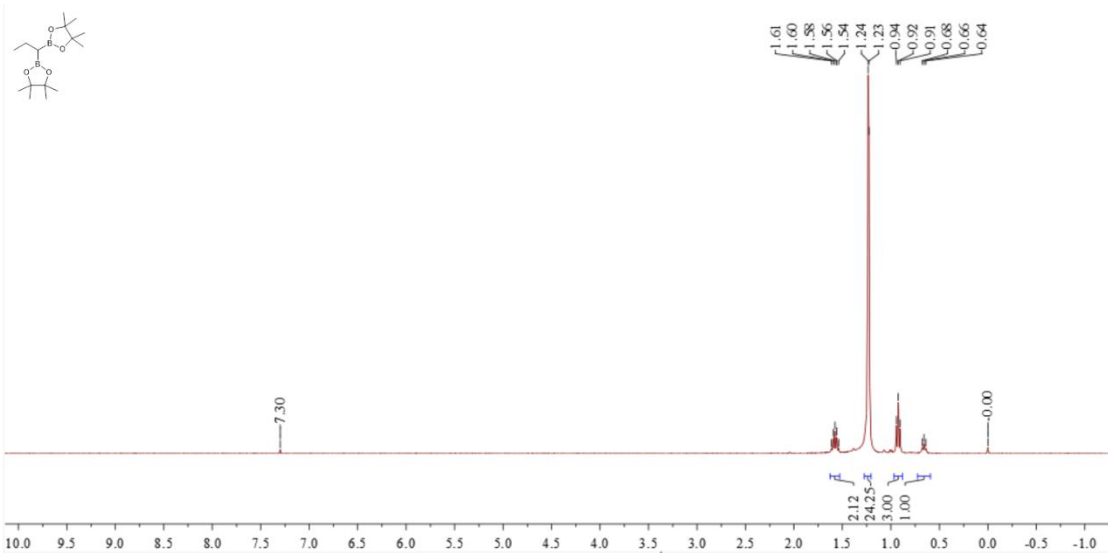

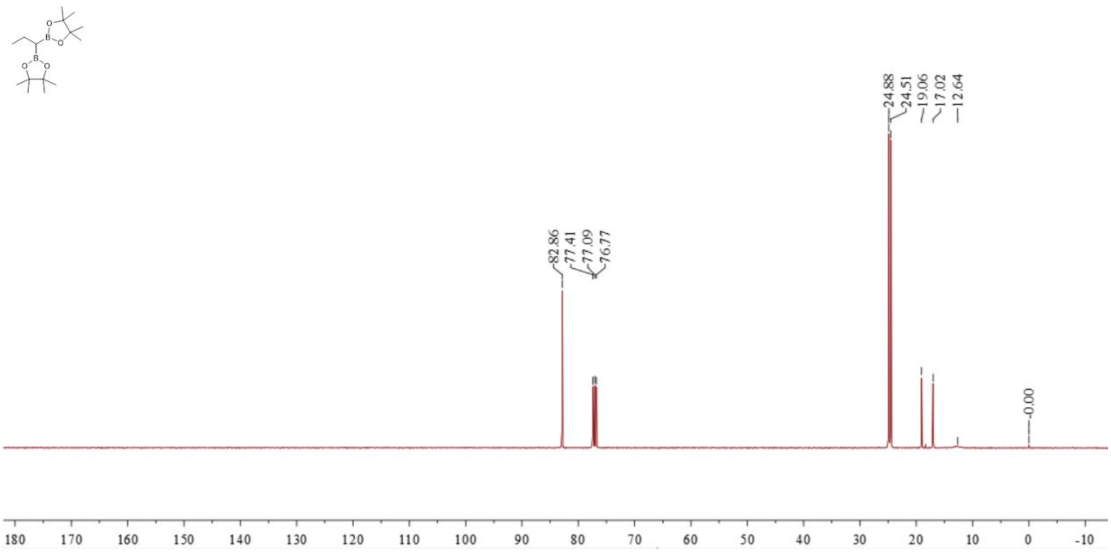

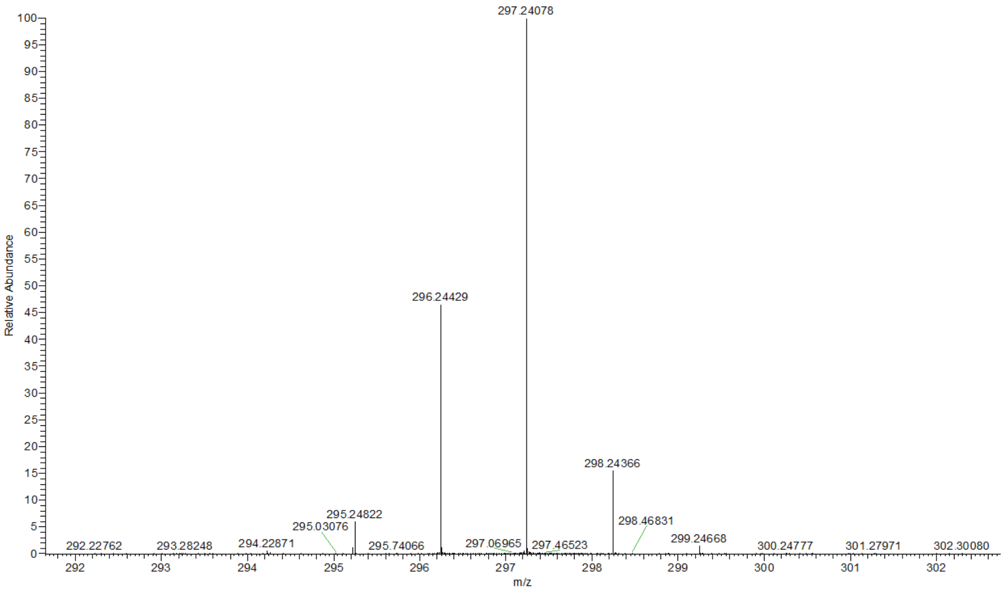

Supplementary Figure 54. ^{1}H, ^{13}C NMR and HRMS of compound 43

Supplementary Methods

Materials

All reactions were carried out in oven-dried Schlenk tubes under an argon atmosphere (purity ≥99.999%). Bis(1,5-cyclooctadiene)nickel(0) (98%, Strem Chemicals) was purchased as a golden yellow solid particles. (9,9-Dimethyl-9*H*-xanthene-4,5-diyl)bis(dicyclohexylphosphane) (Cy-XantPhos) was purchased from Beijing HWRK Chemicals as an off-white powder in 98% purity. Bis(pinacolato)diboron (99%, Alfa-Aesar) was recrystallized from hot petroleum ether. Toluene was purchased from Sinopharm Chemical Reagent Co.,Ltd. 4-Phenyl-1-butene (98%) was purchased from TCI. Anhydrous tetrahydrofuran (99.85%, extra dry, stabilized, acroseal) and triethylamine (99%, TCI) or other trialkylamines (TCI) were used without further purification. The following chemicals were purchased and used as received: LiOMe, $LiOBu^t$, $NaOBu^t$, $KOBu^t$ (99%, J&K), 4-fluoro-pheno and other phenols (J&K), allylbromide, 3-bromo-2-methylpropene, cinnamyl bromide, 4-bromo-1-butene, 5-bromo-1-pentene, 6-bromohex-1-ene, 7-bromo-1-heptene, 8-bromooct-1-ene, 9-bromonon-1-ene, 2,3,4,6-tetra-*O*-benzyl-D-glucopyranose, diethylaminosulfur trifluoride (DAST) (Energy Chemical), oct-1-ene, 1-decene, 1-dodecene, 4-vinyl-1-cyclohexene, 10-undecenoic acid, 4-(but-3-enyl)-4'-(3,4-difluorophenyl)bi(cyclohexane), pentafluorostyrene, vinylmesitylene, 4-phenyl-1-butyne, bis(cyclopentadienyl)zirconium chloride hydride (Schwartz's Reagent), 1,2:5,6-bis-*O*-(1-methylethylidene)-*a*-D-glucofuranose (TCI), allyltrimethylsilane, *R*-glyceraldehyde-acetonide (J&K), bis[(+)-pinanediolato]diboron (BePharm. Ltd.), *tert*-butyldimethylsilyl chloride, indole, 9*H*-carbazole, *N*-methylbenzylamine, vinylarenes, PPh_3, PCy_3, PCy_2But, PCy_2Ph, tricyclopentylphosphine and 2-(dicyclohexylphosphino)-2',4',6'-triisopropylbiphenyl (X-Phos) (Alfa Aesar), 2-(dicyclohexylphosphino)-3,6-dimethoxy-2'-4'-6'-tri-i-propyl-1,1'-biphenyl (BrettPhos), bis(dicyclohexylphosphino)alkanes, dimethylbisdiphenylphosphinoxanthene (XantPhos), 2,2'-(di-*o*-tolylphosphino)diphenylether (DPEPhos), IMes·HCl, ICy (NHC ligands) and 1,1'-bis(dicyclohexylphosphino)ferrocene (Beijing HWRK Chemicals). Anhydrous DMF, DMA, 1,4-dioxane, 1,3,5-trimethylbenzene and methyl *tert*-butyl ether were stored over 4Å molecular sieves under an argon atmosphere in a septum-capped bottle. All other reagents and solvents mentioned in this text were purchased from commercial sources and used without purification.

Analytical Methods

^1H NMR, ^{13}C NMR and ^{19}F NMR spectra were recorded either on Bruker Avance 400 or Varian Mercury 400 spectrometer at ambient temperature in $CDCl_3$ unless otherwise noted. Data for ^1H NMR are reported as follows: chemical shift (δ ppm), multiplicity, integration, and coupling constant (Hz). Data for ^{13}C NMR are reported in terms of chemical shift (δ ppm), multiplicity, and coupling constant (Hz). Gas chromatographic (GC) analysis was acquired on a Shimadzu GC-2014 Series GC System equipped with a flame-ionization detector. GC-MS analysis was performed either on Thermo Scientific AS 3000 Series GC-MS System or Agilent 6890N gas chromatograph coupled to an Agilent 5973 inert mass selective detector. Infrared (IR) spectra were recorded on a Bruker alpha spectrophotometer. Bands are characterized as broad (br), strong (s), medium (m), and weak (w) (ν_{max} cm^{-1}). Optical rotations were measured on a Perkin-Elmer 343 Polarimeter with a sodium lamp at λ = 589 nm. HRMS analysis was performed on Bruker Autoflex MALDI-TOF MS system. Thin-layer chromatography was performed with silica gel 60 F_{254} plates eluting with solvents indicated, visualized by a 254 nm UV lamp and stained by potassium permanganate ($KMnO_4$). Flash chromatography was performed using silica 60 (230-400 mesh).

Preparation of aryloxy-derived terminal alkenes

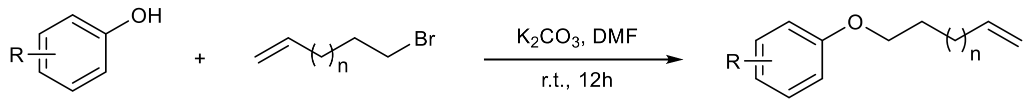

General procedure A: A mixture of phenol (20 mmol) and K_2CO_3 (3.3 g, 24 mmol) in DMF (100 mL) was stirred for 1 hour at room temperature, and bromides (32 mmol) was added. After stirring overnight, the mixture was poured into water (200 mL) and extracted with CH_2Cl_2. The organic phase was washed with 5% NaOH (3 × 100 mL) and water (2 × 100 mL), dried over Na_2SO_4, followed by usual work-up and purified on silica gel column chromatography to afford the desired compounds.

Preparation of alkoxy-derived terminal alkenes

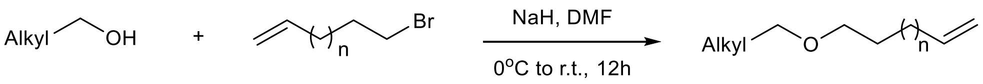

General procedure B: To a solution of alcohols (20 mmol) in DMF (100 mL), sodium hydride powder (22 mmol, 60% dispersion in mineral oil) was added at 0°C. The solution was kept at this temperature for 1h and then bromides (30.0 mmol) were added slowly. Stirring at r.t. overnight, the reaction was quenched by glacial water and then extracted with CH_2Cl_2. The organic phase was dried over Na_2SO_4, followed by usual work-up and purified on silica gel column chromatography to afford the desired compounds.

Preparation of *N,N*-disubstituted terminal alkenes

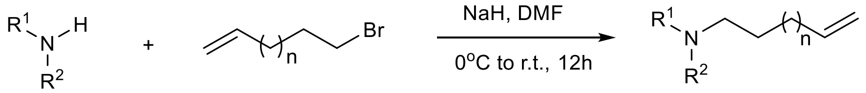

General procedure C: To a solution of amine (indole or 9*H*-carbazole) (10 mmol) in DMF (50 mL), sodium hydride powder (12 mmol, 60% dispersion in mineral oil) was added at 0°C. The solution was kept at this temperature for 1h and then bromides (15 mmol) were added slowly. Stirring overnight at r.t., the reaction was quenched by glacial water and then extracted with CH_2Cl_2. The organic phase was dried over Na_2SO_4, followed by usual work-up and purified on silica gel column chromatography to afford the desired compounds.

It is worth noting that some substrates and organoboron compounds formed in this study are not very UV active, but they could be well stained in $KMnO_4$ aqueous solution.

General Procedures D: for 1,1-diboration terminal aliphatic alkenes described in Table 2

In a glove box, to a 10 mL Schlenk tube equipped with a stir bar were added B_2pin_2 (0.4 mmol, 101.6 mg), 5% $Ni(COD)_2$ (2.8 mg), 5% Cy-XantPhos (6.0 mg) and LiOMe (0.2 mmol, 7.6 mg). Sealed with a teflon cap, the tube was removed from the glove box. The vessel was then evacuated and filled with argon (three cycles). 0.55 mL PhMe/THF (v/v = 10:1), NEt_3 (0.5 equiv., 14 μL) and aliphatic alkene (if the alkene was solid, it can be added to the tube at first). Capped with the teflon cap, the reaction mixture was allowed to stir at room temperature for seconds, then transferred into a 130°C oil bath. After 1 hour stirring, the reaction completed and was cooled to room temperature. The reaction mixture was then diluted with EtOAc, filtered through a short pad of silica gel with copious washings

(EtOAc), concentrated, and purified by column chromatography to afford the desired 1,1-diboration product.

General Procedures E: for 1,1-diboration of vinylarenes described in Table 3

In a glove box, to a 10 mL Schlenk tube equipped with a stir bar were added B_2pin_2 (0.4 mmol, 101.6 mg), 5% $Ni(COD)_2$ (2.8 mg), 10% PCy_3 (5.6 mg) and LiOMe (0.2 mmol, 7.6 mg). Sealed with a teflon cap, the tube was removed from the glove box. The vessel was then evacuated and filled with argon (three cycles). 0.55 mL PhMe/THF (v/v = 10:1), NEt_3 (0.5 equiv., 14 μL) and vinylarene (if the vinylarene was solid, it can be added to the tube at first). Capped with the teflon cap, the reaction mixture was allowed to stir at room temperature for seconds, then transferred into a 130°C oil bath. After 1 hour stirring, the reaction completed and was cooled to room temperature. The reaction mixture was then diluted with EtOAc, filtered through a short pad of silica gel with copious washings (EtOAc), concentrated, and purified by column chromatography to afford the desired 1,1-diboration product.

General Procedure for 1,1-diboration of lower alkenes (propylene and ethylene)

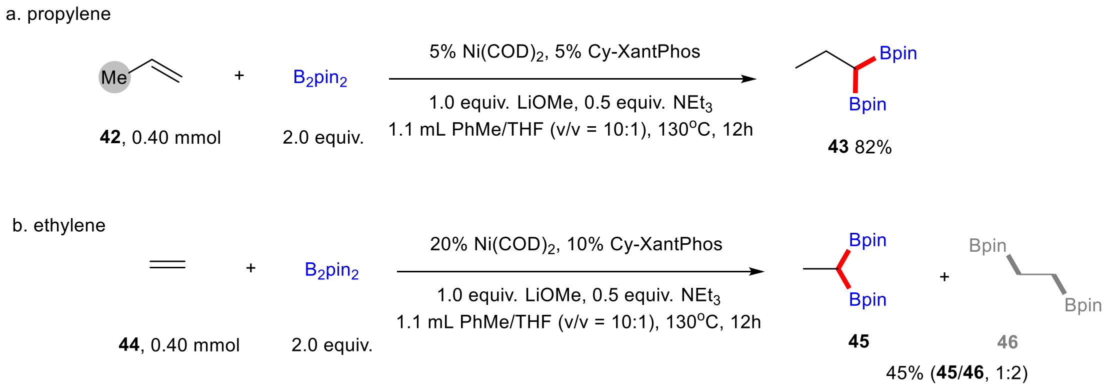

General Procedure F: In a glove box, to a 10 mL Schlenk tube equipped with a stir bar were added B_2pin_2 (2.0 equiv.), $Ni(COD)_2$, Cy-XantPhos and LiOMe (1.0 equiv.). Sealed with a teflon cap, the tube was removed from the glove box. *Then the tube was evacuated under vacuum and lower alkenes was fully charged (three cycles), followed by NEt_3 (0.5 equiv.) and 1.1 mL PhMe/THF (v/v = 10:1).* Capped with the teflon cap, the reaction mixture was allowed to stir at r.t. for seconds, then transferred into a 130°C oil bath. After 12 hours stirring, the reaction completed and was cooled to room temperature. The reaction mixture was then diluted with EtOAc, filtered through a short pad of silica gel with copious washings (EtOAc), concentrated, and purified by column chromatography to afford the desired diboration product (7-10% EtOAc in petroleum ether, stained by $KMnO_4$ in water).

For propylene 42: 5% $Ni(COD)_2$ and 5% Cy-XantPhos was used. **43** was obtained as a colorless oil in 82% yield (97 mg).

For ethylene 44: 20% $Ni(COD)_2$ and 10% Cy-XantPhos was used. **45** and **46** was obtained as a colorless oil in 45% combined yield (50.8 mg)with poor selectivity (Ratio of **45**/**46** was 1:2). Despite our best effort, the selectivity was only slightly improved to 1:2. We also conducted the diboration reaction of **44** in high-pressure reactor using 1.5MPa of ethylene, but only 1,2-diboration product **46** was detected (The diboration optimization results of **44** are listed as below, see the following equations and figures).

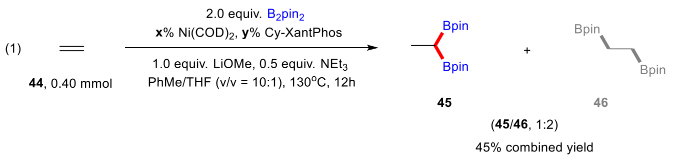

entry	x/%	y/%	43/%	45/%	45/46
1	0	0	0	n.d.	< 1:100
2	10	0	0	n.d.	< 1:100
3	5	5	0	n.d.	< 1:100
4	10	10	0	n.d.	< 1:100
5	15	15	0	n.d.	< 1:100
6	20	20	0	n.d.	< 1:100
7	5	2.5	trace	n.d.	< 1:100
8	10	5	42% combined yield		1:2.5
**9**	**20**	**10**	**15**	**30**	**1:2.0**

n.m. = not determined

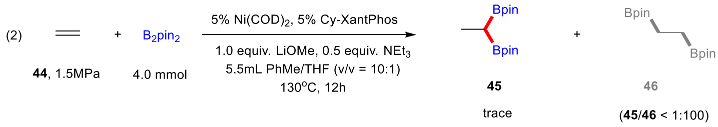

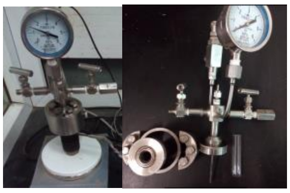

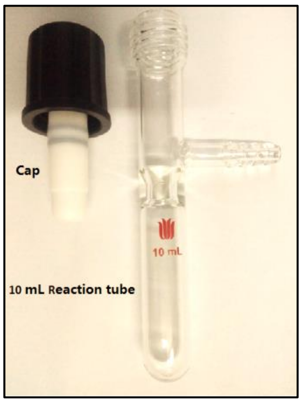

Supplementary Figure 55. 1,1-Diboration Schlenk reaction tube (10 mL)

NMR and **GC-MS spectra** of 2,2'-(ethane-1,1-diyl)bis(4,4,5,5-tetramethyl-1,3,2-dioxaborolane) (**45**) and 1,2-bis(4,4,5,5-tetramethyl-1,3,2-dioxaborolan-2-yl)ethane (**46**)

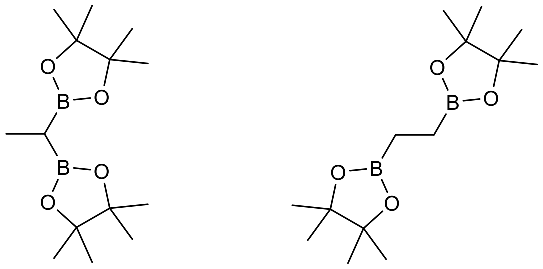

45 **46**

^{1}**H NMR** (400 MHz, $CDCl_3$) δ 1.24 (s, 72H), 1.04 (d, J = 7.3 Hz, 3H), 0.84 (s, 8H), 0.72 (dd, J = 14.4, 7.2 Hz, 1H). ^{13}**C NMR** (101 MHz, $CDCl_3$) δ 82.90, 82.82, 25.03, 24.84, 24.82, 24.73, 24.55, 9.07, 4.45.

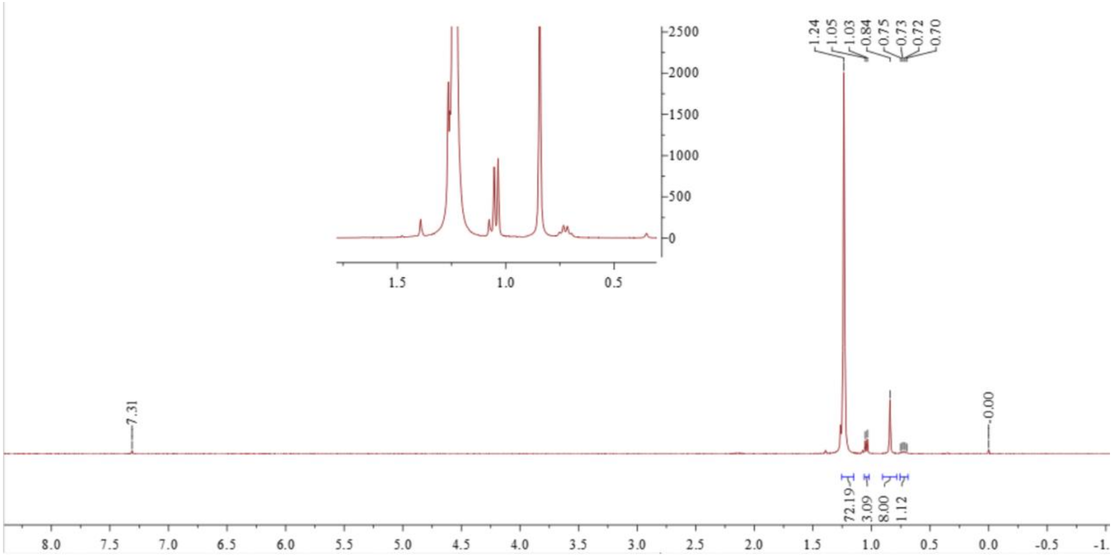

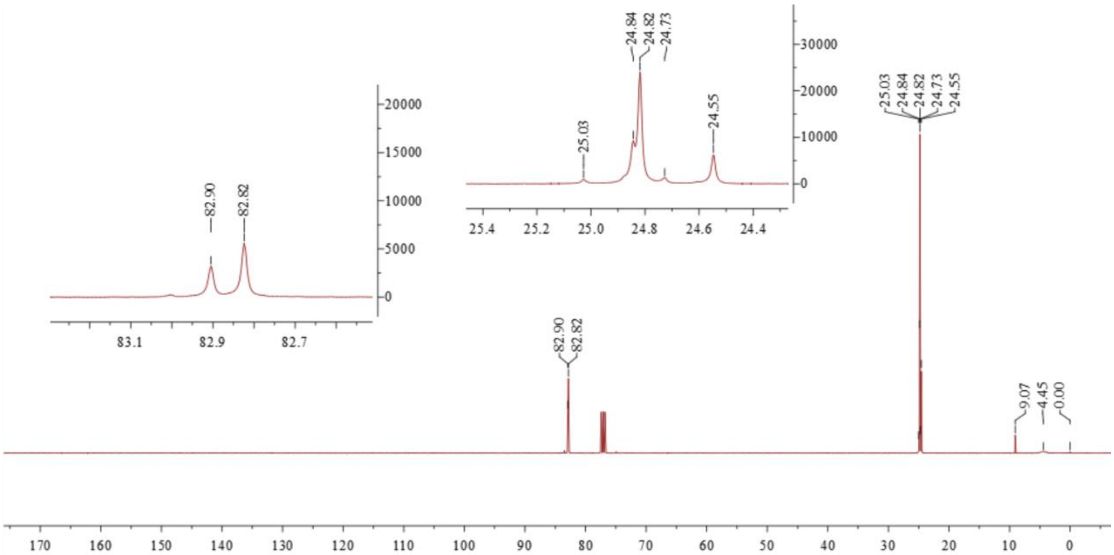

Supplementary Figure 56. ^{1}H and ^{13}C NMR of compounds 45 and 46

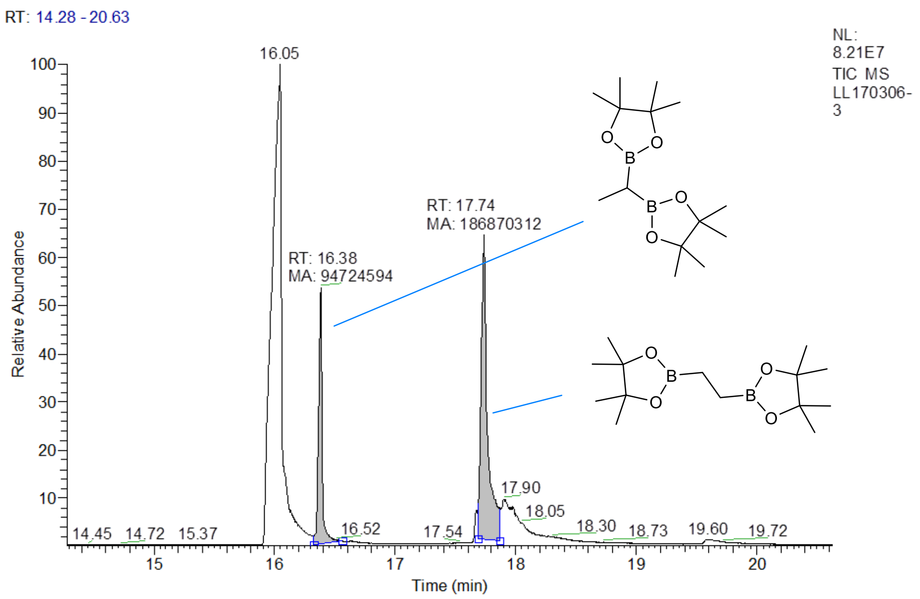
RT: 14.28 - 20.63
NL: 8.21E7 TIC MS LL170306-3
16.05
RT: 16.38
MA: 94724594
RT: 17.74
MA: 186870312
17.90
18.05
14.45
14.72
15.37
16.52
17.54
18.30
18.73
19.60
19.72
Relative Abundance
Time (min)

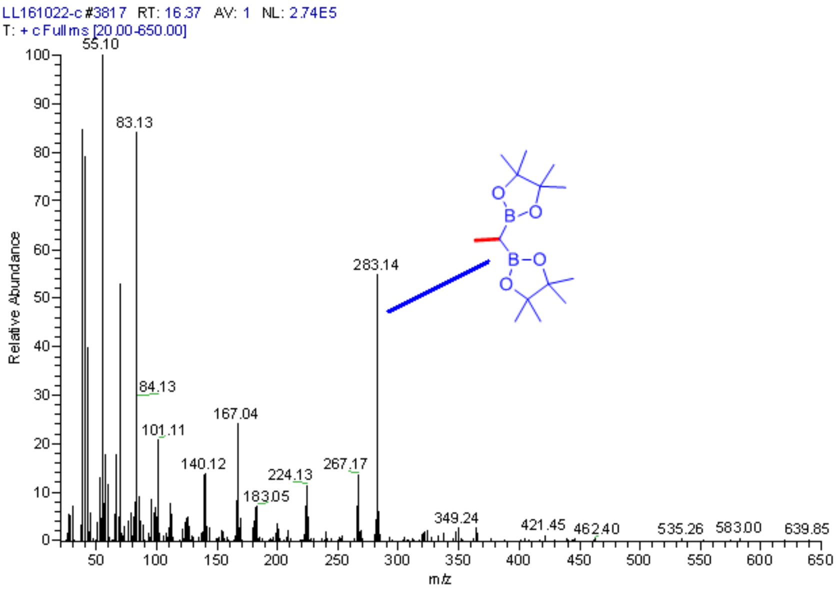
LL161022-c #3817 RT: 16.37 AV: 1 NL: 2.74E5
T: + c Full ms [20.00-650.00]
55.10
83.13
283.14
84.13
167.04
101.11
140.12
224.13
267.17
183.05
349.24
421.45
462.40
535.26
583.00
639.85
Relative Abundance
m/z

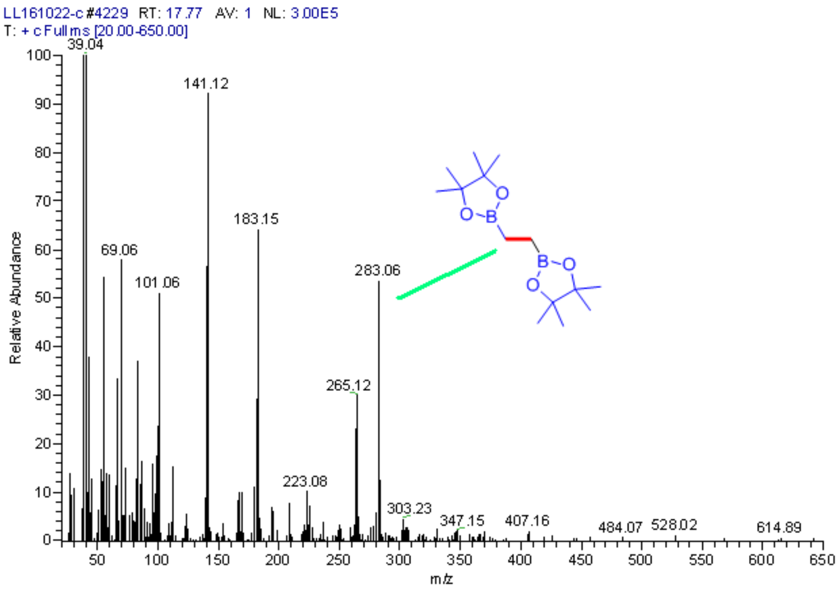

Supplementary Figure 57. GC-MS of compounds 45 and 46

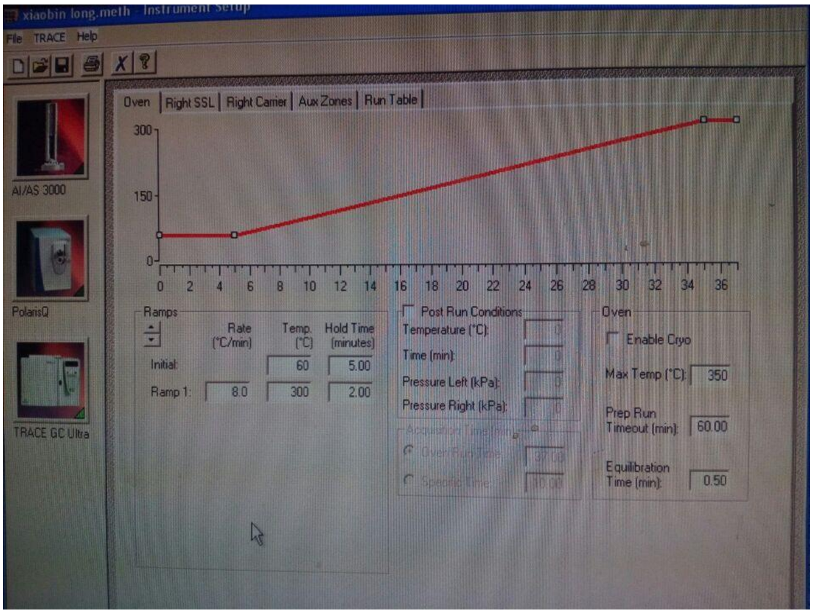

Supplementary Figure 58. GC-MS analysis method of compounds 45 and 46

Gram-scale synthesis of 1,1-diboron compound 6

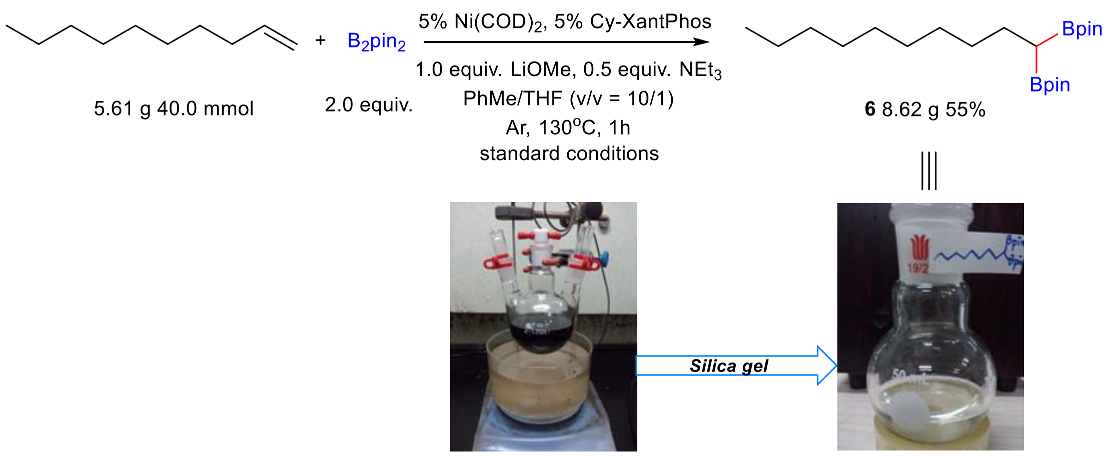

In a glove box, a mixture of 5% $Ni(COD)_2$ (550.1 mg, 2.0 mmol), 5% Cy-XantPhos (1.21 g, 2.0 mmol), LiOMe (1.52 g, 40.0 mmol, 1.0 equiv.) and B_2pin_2 (20.3 g, 80.0 mmol, 2.0 equiv.) were added to a three-necked round bottom flask equipped with a stir bar. The flask was evacuated and filled with argon for three cycles. 110 mL PhMe/THF (v/v = 10:1), NEt_3 (2.78 mL, 0.5 equiv.) and 1-decene (7.97 mL, 40.0 mmol) were added respectively under a positive flow of argon. The reaction mixture was stirred at r.t. for seconds and then transferred to a pre-heated 130°C oil bath. After 1 hour stirring, the reaction mixture was cooled to r.t. and then diluted with EtOAc, filtered through a short pad of silica gel with copious washings by EtOAc, concentrated, and purified by silica gel (7-10% EtOAc in petroleum ether, stained by $KMnO_4$ in water) to afford the desired 1,1-diboron product **6** as a thick oil (8.62 g, 55% yield).

Gram-scale synthesis of 1,1-diboron compound 13

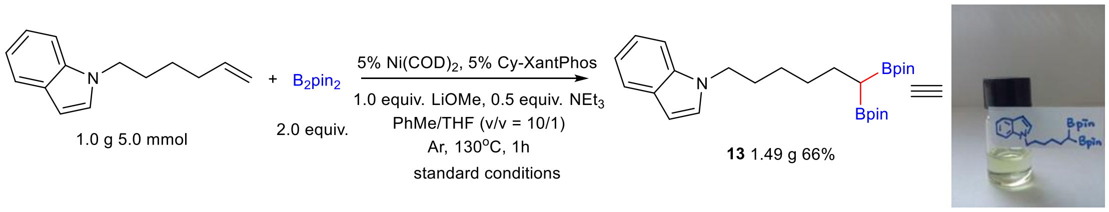

In a glove box, a mixture of 5% $Ni(COD)_2$ (68.8 mg, 0.25 mmol), 5% Cy-XantPhos (151.2 mg, 0.25 mmol), LiOMe (190.0 g, 5.0 mmol, 1.0 equiv.) and B_2pin_2 (2.54 g, 10.0 mmol, 2.0 equiv.) were added to a 25 mL Schlenk tube equipped with a stir bar. The tube was evacuated and filled with argon for three cycles. PhMe (12.5 mL)/THF (1.25 mL) (v/v = 10:1), NEt_3 (348 μL, 0.5 equiv.) and 1-(hex-5-en-1-yl)-1*H*-indole (1.0 g, 5.0 mmol) were added respectively under a positive flow of argon. The reaction mixture was stirred at r.t. for seconds and then transferred to a pre-heated 130°C oil bath. After 1 hour stirring, the reaction mixture was cooled to r.t. and then diluted with EtOAc, filtered through a short pad of silica gel with copious washings by EtOAc, concentrated, and purified by silica gel (7-10% EtOAc in petroleum ether) to afford the desired 1,1-diboration product **13** as a thick oil (1.49 g, 66% yield).

Supplementary Tables

Supplementary Table 1. Solvent optimization

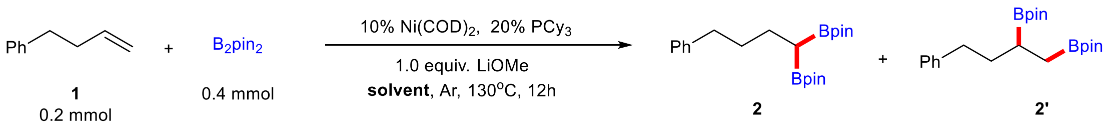

Entry	Solvents	Yield/%	
		**2**	**2'**
1	dioxane	3	trace
2	DMF	trace	77
3	*t*-BuOMe	37	trace
4	PhMe	**38**	trace
5	mesitylene	37	trace
6	2-methyl-2-butanol	8	4

Supplementary Table 2. Ligands optimization

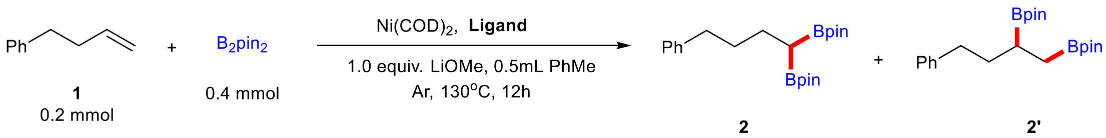

Entry	Ligands	Yield/%	
		**2**	**2'**
1	PCy_3	39	trace
2	1,10-phenanthroline	0	22
3	4,4',4''-tri-*tert*-butyl-2,2':6'2''-terpyridine	trace	55
4	DMAP	trace	trace
5	XPhos	trace	trace
6	RuPhos	trace	trace
7	DPEPhos	trace	trace
8	CPhos	trace	5
9	TriPhos	trace	34
10	Dppe	trace	24
11	dppp	trace	19
12	Cy-XantPhos	**62**	4

Supplementary Table 3. Additives optimization

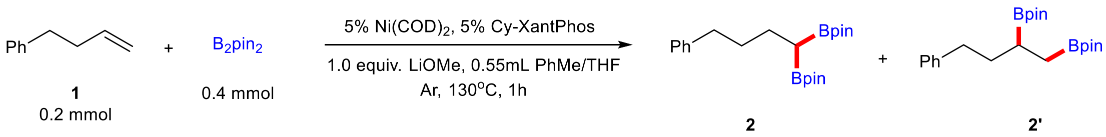

Entry	Additives (1.0 equiv.)	Yield/%	
		**2**	**2'**
1	NEt_3	66	trace
2	*N*-methylpiperidine	62	6
3	*N*-ethylpiperidine	trace	13

4	$MeNEt_2$	59	trace
5	Me_2N^nBu	23	32
6	N^nBu_3	61	6
7	N^iPr_3	60	5
8	NEt_3 (0.5 equiv.)	**78**	trace
9	TBAB	trace	trace
10	4Å MS (15.0 mg)	trace	trace

Supplementary Table 4. Nickel catalysts optimization

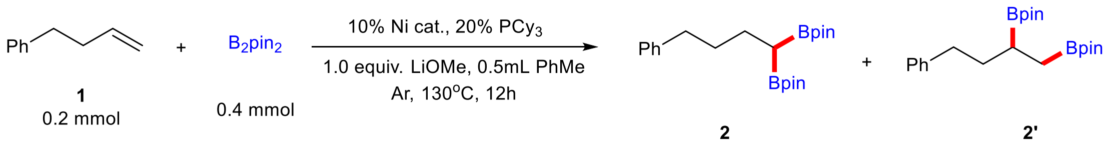

Entry	Ni catalyst (10%)	Yield/%	
		**2**	**2'**
1	$NiBr_2{\cdot}dme$	5	6
2	$NiCl_2{\cdot}dme$	5	13
2	$NiCl_2$	10	15
3	$NiCl_2(PCy_3)_2$	12	12
4	$Ni(PPh_3)_4$	0	33
5	$Ni((PhO)_3P)_4$	0	38

Supplementary Discussions

Cross-over experiment of B_2pin_2 and B_2pai_2

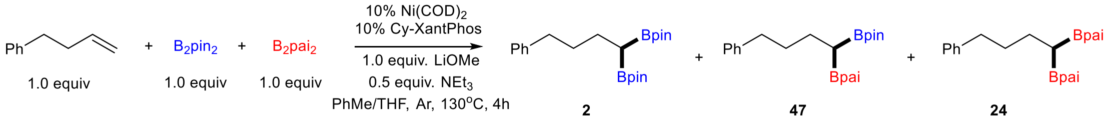

(GC peak area ratio of **2**/**47**/**24**, 1 : 2.2 : 1.1)

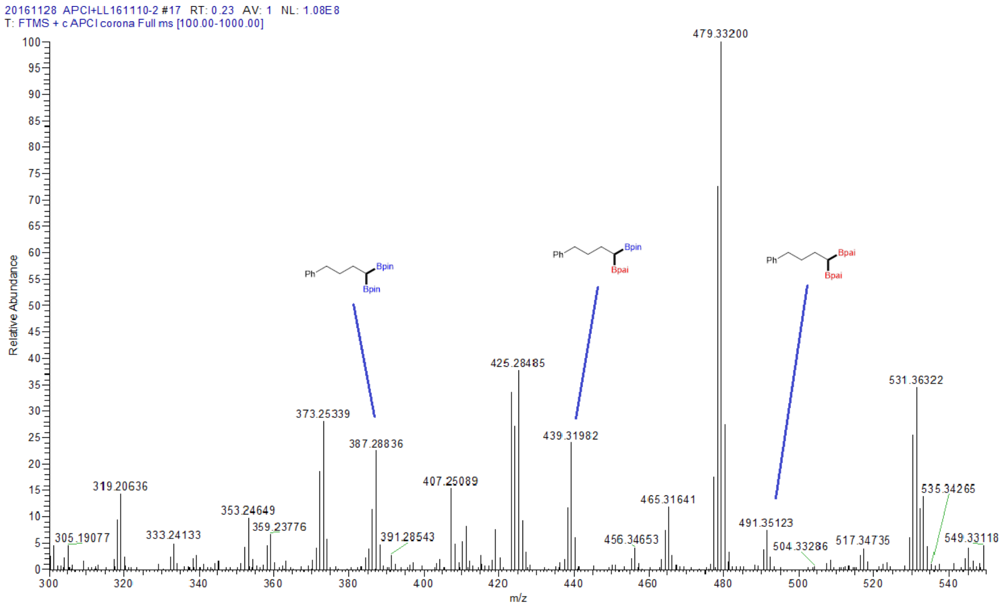

Supplementary Figure 59. HRMS of cross-over experiment result of B_2pin_2 and B_2pai_2

SHIMADZU LabSolutions 分析报告 ——→ Analysis Report

〈样品信息〉

样品名	: LL161110-2c		
样品ID	:		
数据文件名	: LL161110-2c.gcd		
方法文件名	: LL161117-4-(260-20-280).gcm		
批处理文件名	: LL161110-2.gcb		
样品瓶号	: 7	样品类型	: 未知
进样体积	: 1 uL		
分析日期	: 2016/11/17 2:46:48	分析者	: System Administrator
处理日期	: 2016/11/17 3:06:10	处理者	: System Administrator

〈色谱图〉

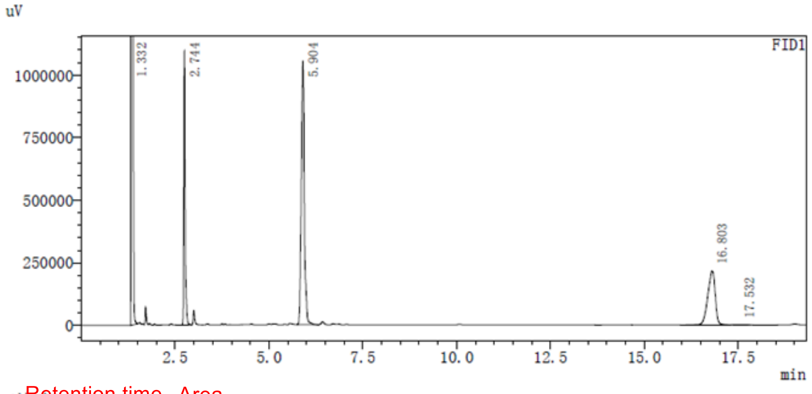

〈峰表〉 Retention time Area

FID1

No. ←

峰号	保留时间	面积	高度	浓度	浓度单位	标记	化合物名
1	1.332	345990848	215507593	96.719			
2	2.744	2727923	1087238	0.763			
3	5.904	5982736	1051214	1.672			
4	16.803	3015772	216744	0.843		S	
5	17.532	10595	956	0.003		T	
总计		357727874	217863745				

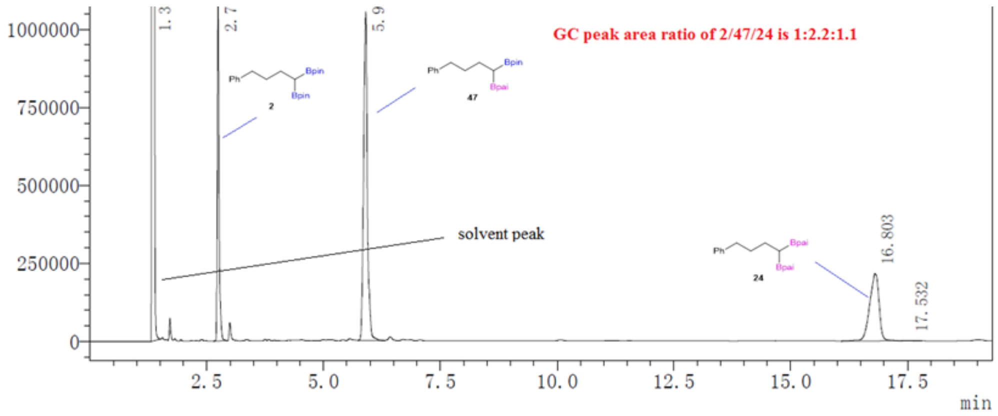

〈峰表〉 Retention time Area

FID1

No.

峰号	保留时间	面积	高度	浓度	浓度单位	标记	化合物名
1	1.332	345990848	215507593	96.719			
2	2.744	2727923	1087238	0.763			
3	5.904	5982736	1051214	1.672			
4	16.803	3015772	216744	0.843		S	
5	17.532	10595	956	0.003		T	
总计		357727874	217863745				

Supplementary Figure 60. GC-MS analysis report of compounds 2, 47 and 24

Cross-over experiment of B_2pin_2 and B_2pin_2-d_{12}

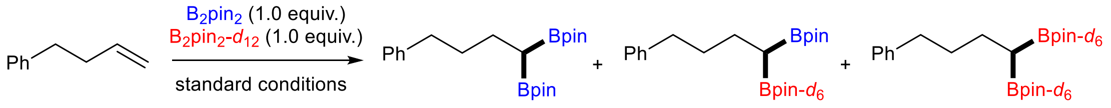

1 **2** **2-d_6** **2-d_{12}**

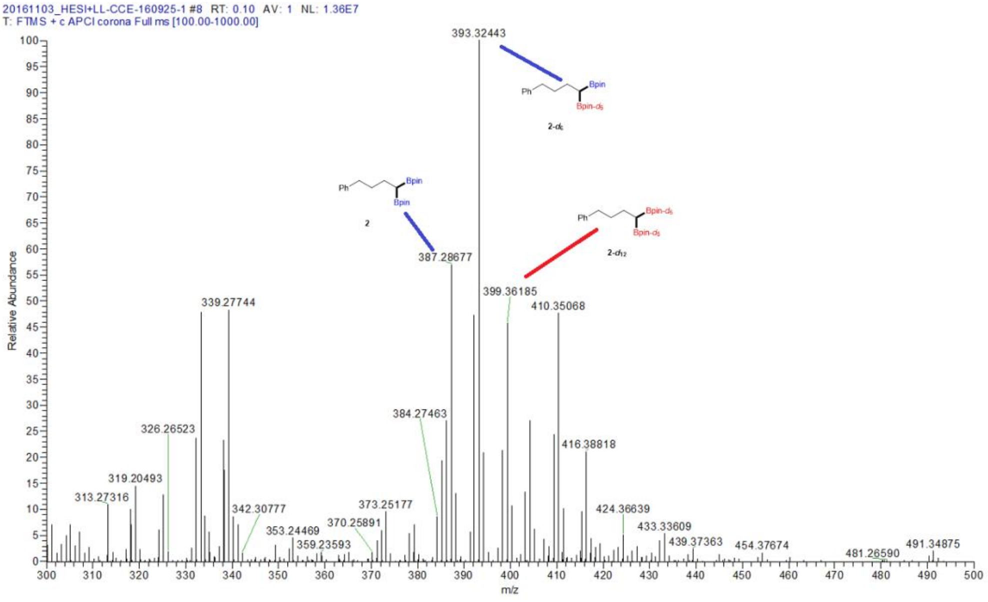

Supplementary Figure 61. HRMS of cross-over experiment result of B_2pin_2 and B_2pin_2-d_{12}

Deuterium labeling experiment

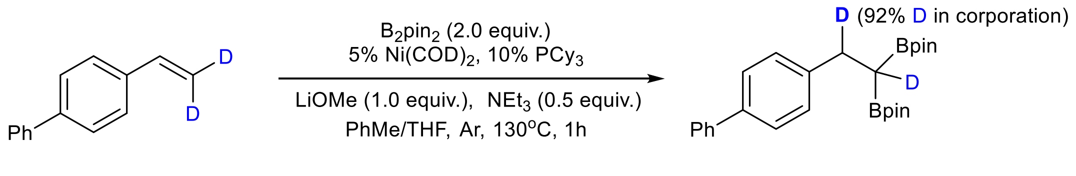

29a-*d*$_2$ 93% D in corporation

29-*d*$_2$ 40%

In a glove box, to a 10 mL Schlenk tube equipped with a stir bar were added B_2pin_2 (0.4 mmol, 101.6 mg), **29a-*d*$_2$** (0.2 mmol, 36.4 mg), 5% $Ni(COD)_2$ (2.8 mg), 10% PCy_3 (5.6 mg) and LiOMe (0.2 mmol, 7.6 mg). Sealed with a cap, the tube was removed from the glove box. The vessel was then evacuated and filled with argon (three cycles) and followed by addition of 0.55 mL PhMe/THF (v/v = 10:1), NEt_3 (0.5 equiv., 14 μL). Capped with the cap, the reaction mixture was allowed to stir at room temperature for seconds, and then transferred into a 130°C oil bath. After 1 hour stirring, the reaction completed and was cooled to room temperature. The reaction mixture was then diluted with EtOAc, filtered through a short pad of silica gel with copious washings (EtOAc), concentrated, and purified by column chromatography (7-10% EtOAc in petroleum ether) to afford **29-*d*$_2$** as a colorless solid (40%, 35 mg). One of benzylic hydrogen atoms in **29-*d*$_2$** was 92% deuterated (see Fig. 53).

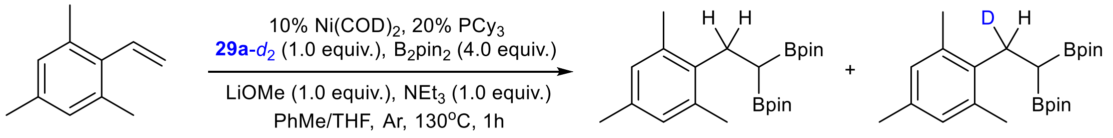

0.2 mmol

30

30-*d*$_1$

44% (**30** : **30-*d*$_1$** = 2.3 : 1)

In a glove box, to a 10 mL Schlenk tube equipped with a stir bar were added B_2pin_2 (0.8 mmol, 203.2 mg), **29a-*d*$_2$** (0.2 mmol, 36.4 mg), 10% $Ni(COD)_2$ (5.5 mg), 20% PCy_3 (11.2 mg) and LiOMe (0.4 mmol, 15.2 mg). Sealed with a cap, the tube was removed from the glove box. The vessel was then evacuated and filled with argon (three cycles) and followed by addition of 0.55 mL PhMe/THF (v/v = 10:1), NEt_3 (0.5 equiv., 28 μL) and vinylmesitylene (0.2 mmol, 29.2 mg). Capped with the cap, the reaction mixture was allowed to stir at room temperature for seconds, and then transferred into a 130°C oil bath. After 1 hour stirring, the reaction completed and was cooled to room temperature. The reaction mixture was then diluted with EtOAc, filtered through a short pad of silica gel with copious washings (EtOAc), concentrated, and purified by silica gel (7-10% EtOAc in petroleum ether) to afford **30** and **30-*d*$_1$** as a thick oil mixture in 44% yield (ratio of **30**/**30-*d*$_1$** is 2.3/1) (see Fig. 62).

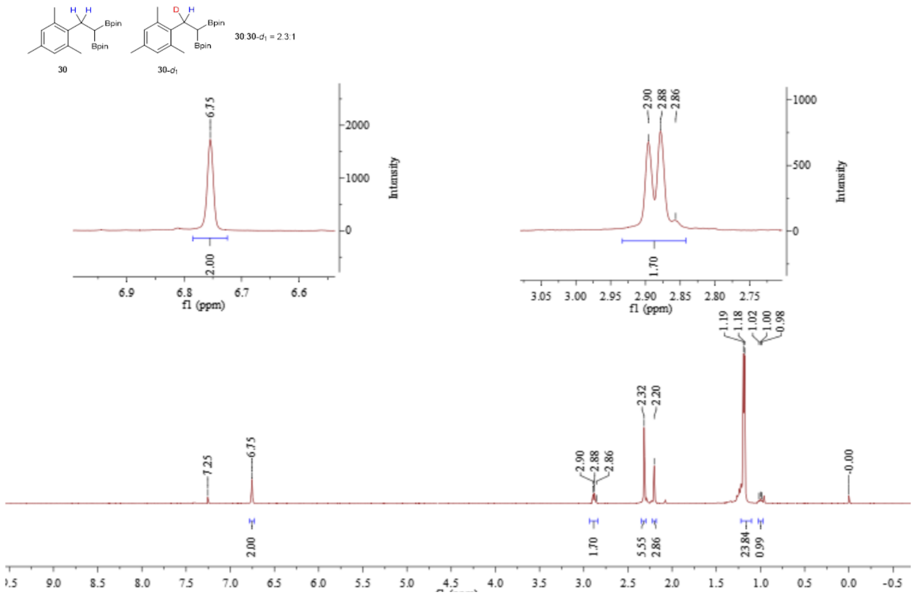

Supplementary Figure 62. **^{1}H NMR of 30 and 30-d_1**

Proposed catalytic cycle

Based on the mechanism experiments we conducted, a relatively probable catalytic cycle was proposed (shown as follows). The transmetalation of Bpin motif *via* boron-ate complex to nickel complex would give a nickel boryl species **A** (path a). Insertion of aliphatic alkene **1** into the Ni-B bond in **A** generates **B** (path b), which undergoes β-hydride elimination to give species **C** (path c). According to the ratio of **30** to **30-d_1**, we reason that 1-alkenyl boron is not entirely free and is more inclined to coordinate with nickel hydride species forming **C** in path c. Followed by reaction of **C** with boron-ate complex, and then the insertion proceeds (path d). Two possible insertion patterns generate complexes **D** or **E** respectively, which then reductively eliminates to furnish the product **2** and regenerates the catalyst (path e and e').

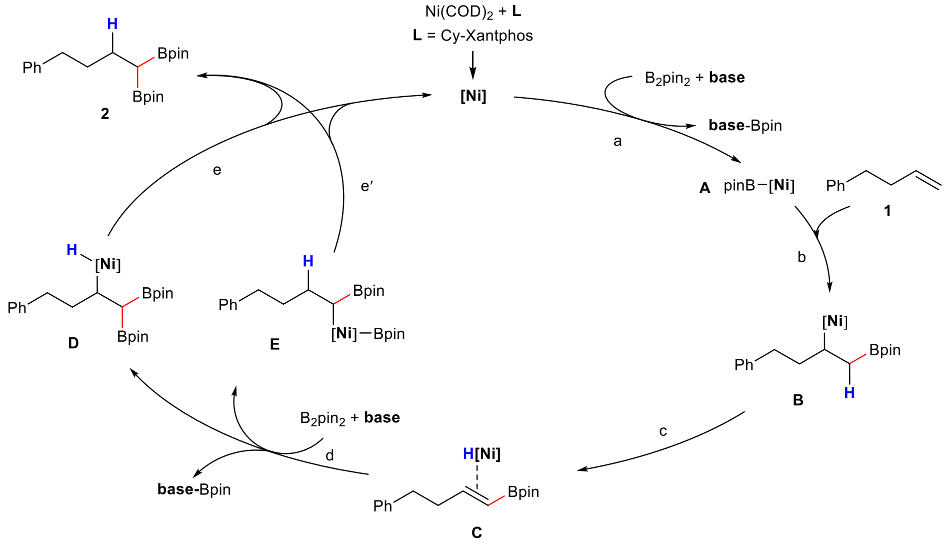

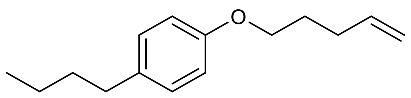

butyl-4-(pent-4-en-1-yloxy)benzene

Prepared according to the general procedure A using 4-butylphenol (3.0 g, 20.0 mmol), K_2CO_3 (3.3 g, 24 mmol) and 5-bromo-1-pentene (3.8 mL, 32 mmol) in DMF (100 mL). The crude reaction mixture was poured into water (500 mL) and extracted with EtOAc (70 mL × 3). The organic phase was combined, washed with 5% NaOH (3 × 100 mL), dried over Na_2SO_4, concentrated and purified on silica gel (5% EtOAc/hexanes) to afford the product as a colorless oil (3.8 g, 87% yield). **^{1}H NMR** (400 MHz, $CDCl_3$) δ 7.06 (d, *J* = 8.5 Hz, 2H), 6.80 (d, *J* = 8.6 Hz, 2H), 5.84 (m, 1H), 5.02 (m, 2H), 3.92 (t, *J* = 6.4 Hz, 2H), 2.67 – 2.49 (m, 2H), 2.22 (m, 2H), 1.95 – 1.75 (m, 2H), 1.55 (m, 2H), 1.33 (m, 2H), 0.91 (t, *J* = 7.3 Hz, 3H). **^{13}C NMR** (101 MHz, $CDCl_3$) δ 157.08, 137.92, 134.88, 129.21, 115.09, 114.28, 67.15, 34.75, 33.94, 30.17, 28.53, 22.32, 13.98. **IR**(neat): 2956 (m), 2929 (s), 2859 (m), 1607 (m), 1512 (s), 1471 (w), 1243 (s), 1174 (m), 915 (m), 823 (m) cm^{-1}. **HRMS** Calcd for $C_{15}H_{23}O$ $[M+H]^+$ 219.1743; Found: 219.1744.

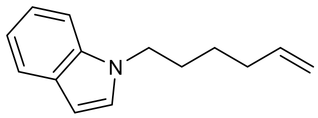

1-(hex-5-en-1-yl)-1*H*-indole

Prepared according to the general procedure C using indole (1.16 g, 10.0 mmol), NaH (480 mg, 12 mmol, 60% dispersion in mineral oil) and 6-bromo-1-hexene (2.0 mL, 15 mmol) in DMF (50 mL). The reaction was quenched by glacial water and then extracted with CH_2Cl_2 (50 mL × 3). The organic phase was combined and dried over Na_2SO_4, followed by the usual work-up and purified on silica gel (5-8% EtOAc in petroleum ether) to afford the product as a clear oil (1.3 g, 65% yield). **^{1}H NMR** (400 MHz, $CDCl_3$) δ 7.61 (d, *J* = 7.9 Hz, 1H), 7.29 (d, *J* = 8.2 Hz, 1H), 7.18 (t, *J* = 7.6 Hz, 1H), 7.08 (t, *J* = 7.4 Hz, 1H), 7.02 (d, *J* = 2.9 Hz, 1H), 6.46 (d, *J* = 2.8 Hz, 1H), 5.72 (m, 1H), 4.95 (t, *J* = 13.6 Hz, 2H), 4.03 (t, *J* = 7.1 Hz, 2H), 2.02 (q, *J* = 7.1 Hz, 2H), 1.83 – 1.71 (m, 2H), 1.43 – 1.29 (m, 2H). **^{13}C NMR** (101 MHz, $CDCl_3$) δ 138.16, 135.88, 128.53, 127.69, 121.27, 120.89, 119.13, 114.89, 109.31, 100.86, 46.13, 33.24, 29.58, 26.14. **IR**(neat): 3055 (m), 2994 (s), 1644 (m), 1511 (m), 1463 (m), 924 (s), 762 (s) cm^{-1}. **HRMS** Calcd for $C_{14}H_{18}N$ $[M+H]^+$ 200.1434; Found: 200.1432.

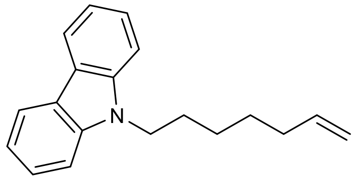

9-(hept-6-en-1-yl)-9*H*-carbazole

Prepared according to the general procedure C using carbazole (1.67 g, 10.0 mmol), NaH (480 mg, 12 mmol, 60% dispersion in mineral oil) and 7-bromo-1-heptene (2.28 mL, 15.0 mmol) in DMF (50 mL). The reaction was quenched by glacial water and then extracted with CH_2Cl_2 (50 mL × 2). The organic phase was combined and dried over Na_2SO_4, followed by the usual work-up and purified on silica gel (7-10% EtOAc in petroleum ether) to afford the product as a colorless thick oil (1.6 g, 61% yield). **^{1}H NMR** (400 MHz, $CDCl_3$) δ 8.08 (d, *J* = 7.7 Hz, 2H), 7.43 (t, *J* = 7.6 Hz, 2H), 7.35 (d, *J* = 8.2 Hz, 2H),

7.19 (dd, *J* = 16.2, 9.2 Hz, 2H), 5.81 – 5.66 (m, 1H), 4.93 (t, *J* = 14.3 Hz, 2H), 4.23 (t, *J* = 7.3 Hz, 2H), 2.00 (t, *J* = 12.2 Hz, 2H), 1.83 (dd, *J* = 13.9, 6.9 Hz, 2H), 1.38 (m, 4H). **^{13}C NMR** (101 MHz, $CDCl_3$) δ 140.34, 138.57, 125.52, 122.77, 120.29, 118.67, 114.50, 108.57, 42.88, 33.54, 28.72, 28.59, 26.67. **IR**(neat): 3053 (m), 2928 (s), 2854 (m), 1640 (w), 1627 (w), 1597 (m), 1463 (s), 1452 (s), 1382 (w), 1347 (m), 1326 (s), 1253 (w), 1230 (m), 1153 (m), 1121 (w), 996 (w), 910 (m), 749 (s), 722 (s) cm^{-1}. **HRMS** Calcd for $C_{19}H_{22}N$ $[M+H]^+$ 264.1747; Found: 264.1748.

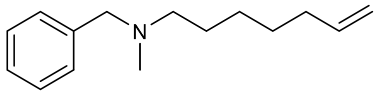

***N*-benzyl-*N*-methylhept-6-en-1-amine**

Prepared according to the general procedure C using benzyl methylamine (1.21 g, 10.0 mmol), NaH (480 mg, 12 mmol, 60% dispersion in mineral oil) and 7-bromo-1-heptene (2.28 mL, 15 mmol) in DMF (50 mL). The reaction was quenched by glacial water and then extracted with CH_2Cl_2 (50 mL × 2). The organic phase was combined and dried over Na_2SO_4, filtrated, concentrated *in vacuo* and purified on silica gel (10% EtOAc in petroleum ether) to afford the product as a colorless oil (1.35 g, 62% yield). **^{1}H NMR** (400 MHz, $CDCl_3$) δ 7.76 (d, *J* = 8.0 Hz, 2H), 7.35 – 7.28 (m, 5H), 6.56 (d, *J* = 15.8 Hz, 1H), 6.14 – 6.03 (m, 1H), 5.84 (m, 1H), 5.23 (t, *J* = 14.8 Hz, 2H), 4.17 (s, 2H), 3.98 (d, *J* = 6.8 Hz, 2H), 3.91 (s, 2H), 3.86 (d, *J* = 5.6 Hz, 2H), 2.42 (s, 3H). **^{13}C NMR** (101 MHz, $CDCl_3$) δ 143.54, 136.12, 136.10, 134.80, 133.84, 129.51, 128.64, 128.09, 127.84, 126.55, 123.03, 117.80, 81.68, 79.10, 70.44, 57.11, 48.78, 36.28, 21.56. **IR**(neat): 2930 (s), 2856 (m), 2787 (m), 1637 (w), 1453 (m), 907 (m), 735 (m), 698 (m) cm^{-1}. **HRMS** Calcd for $C_{15}H_{24}N$ $[M+H]^+$ 218.1903; Found: 218.1904.

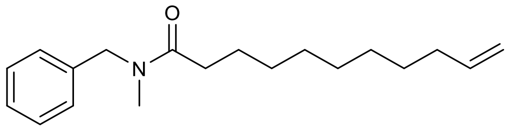

***N*-benzyl-*N*-methylundec-10-enamide**

This substrate was prepared from undecenoic acid (3.3 mL, 16.1 mmol) and pivaloyl chloride (2.0 mL, 16.2 mmol) in the presence of triethylamine (5.8 mL, 48 mmol) in THF (40 mL) followed by the reaction with benzylmethylamine (2.4 mL, 18 mmol). Purified by silica gel (15/100/1, EtOAc/petroleum ether/triethylamine) to afford the desired enamide as a colorless oil (3.19 g, 82% yield). *Spectroscopic data of this enamide were obtained as a mixture of two rotational isomers*. **^{1}H NMR** (400 MHz, $CDCl_3$) δ 7.41 – 7.19 (m, 4H), 7.15 (d, *J* = 7.4 Hz, 1H), 5.89 – 5.72 (m, 1H), 4.95 (dd, *J* = 25.7, 13.6 Hz, 2H), 4.56 (d, *J* = 23.8 Hz, 2H), 2.92 (d, *J* = 12.3 Hz, 3H), 2.36 (t, *J* = 7.5 Hz, 2H), 2.03 (p, *J* = 6.5 Hz, 2H), 1.75 – 1.59 (m, 2H), 1.44 – 1.19 (m, 10H). **^{13}C NMR** (101 MHz, $CDCl_3$) δ 173.60, 173.23, 139.12, 137.58, 136.79, 128.87, 128.52, 127.98, 127.52, 127.23, 126.26, 114.14, 53.33, 50.70, 34.79, 33.83, 33.78, 33.53, 33.10, 29.46, 29.41, 29.35, 29.30, 29.07, 29.05, 28.89, 28.88, 25.40, 25.16. **IR**(neat): 2926 (s), 2854 (m), 1650 (s), 1491 (w), 1453 (m), 1401 (m), 909 (m), 727 (m), 699 (m) cm^{-1}. **HRMS** Calcd for $C_{19}H_{30}NO$ $[M+H]^+$ 288.2322; Found: 288.2323.

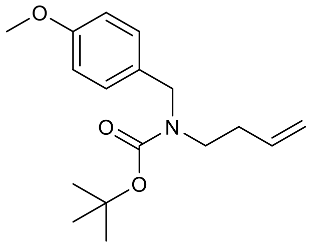

***tert*-butyl but-3-en-1-yl(4-methoxybenzyl)carbamate**

This substrate was prepared as follows, to a round-bottom flask with a magnetic stir bar were added 4-bromo-1-butene (2.7 g, 20 mmol), *p*-methoxybenzylamine (12.4 g, 5.0 equiv.) and ethanol (200 mL). The mixture was stirred at 80°C for 6 h and then concentrated in *vacuo* to remove most of the solvent. The concentrate was washed with water, extracted with EtOAc (100 mL × 3), dried over Na_2SO_4, filtrated, concentrated *in vacuo* and purified by silica gel (EtOAc/petroleum ether/triethylamine, 10/100/1) to afford *N*-(*p*-methoxybenzyl)but-3-en-1-amine as a colorless oil (3.7 g, 97% yield). To a round-bottom flask with a magnetic stir bar charged with the obtained secondary amine (2.9 g, 15.0 mmol), Boc_2O (4.9 g, 22.5 mmol) and THF (100 mL), the reaction mixture was stirred overnight at 50°C. Followed by the normal work-up, purified by silica gel (10-15% EtOAc in petroleum ether) to afford the desired product as a colorless thick oil (3.37g, 77% yield). **^{1}H NMR** (400 MHz, $CDCl_3$) δ 7.16 (s, 2H), 6.85 (d, *J* = 8.3 Hz, 2H), 5.83 – 5.65 (m, 1H), 5.00 (t, *J* = 13.3 Hz, 2H), 4.37 (d, *J* = 9.2 Hz, 2H), 3.77 (s, 3H), 3.32 – 3.09 (m, 2H), 2.22 (s, 2H), 1.48 (s, 9H). **^{13}C NMR** (101 MHz, $CDCl_3$) δ 158.80, 155.91, 155.51, 135.54, 130.55, 129.07, 128.47, 116.42, 113.83, 79.50, 55.18, 50.05, 49.41, 45.89, 32.88, 32.40, 28.44. **IR**(neat): 3006 (w), 2976 (m), 2932 (m), 2838 (w), 1693 (s), 1607 (m), 1513 (s), 1464 (m), 1411 (m), 1366 (m), 1302 (w), 1248 (s), 1170 (s), 1143 (m), 1032 (m), 915 (w), 879 (w) cm^{-1}. **HRMS** Calcd for $C_{17}H_{26}NO_3$ $[M+H]^+$ 292.1907; Found: 292.1908.

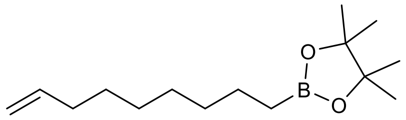

4,4,5,5-tetramethyl-2-(non-8-en-1-yl)-1,3,2-dioxaborolane

This substrate was prepared as follows, in air, CuI (48 mg, 0.25 mmol), PPh_3 (86 mg, 0.33 mmol), LiOMe (380 mg, 5.0 mmol) and bis(pinacolato)diboron (973 mg, 3.8 mmol) were added to a Schlenk tube equipped with a stir bar. The vessel was evacuated and filled with argon (three cycles). DMF (5 mL), the 9-bromonon-1-ene (512 mg, 2.5 mmol) were added under an argon atmosphere. The resulting reaction mixture was stirred vigorously at 25 ℃ for 18 h. The reaction mixture was then diluted with EtOAc, filtered through silica gel with copious washings by EtOAc, concentrated, and purified by silica gel (6-9% EtOAc in petroleum ether, stained by $KMnO_4$ in water) to afford the desired product as a colorless oil (472 mg, 75% yield). **^{1}H NMR** (400 MHz, $CDCl_3$) δ 5.96 – 5.67 (m, 1H), 4.95 (m, 2H), 2.03 (m, 2H), 1.39 (m, 4H), 1.28 (m, 6H), 1.27 – 1.20 (s, 12H), 0.77 (t, *J* = 8.0, 2H). **^{13}C NMR** (101 MHz, $CDCl_3$) δ 139.29, 114.06, 82.83, 33.83, 32.36, 29.24, 29.06, 28.93, 24.82, 23.99, 11.33. **IR**(neat): 2978 (m), 2938 (s), 2855 (m), 1637 (w), 1463 (w), 1378 (s), 1318 (m), 1146 (s), 967 (w) cm^{-1}. **HRMS** Calcd for $C_{15}H_{30}BO_2$ $[M+H]^+$ 253.2333; Found: 253.2337.

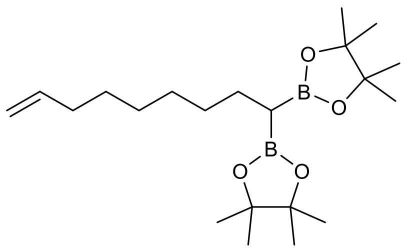

2,2'-(non-8-ene-1,1-diyl)bis(4,4,5,5-tetramethyl-1,3,2-dioxaborolane)

This substrate was prepared as follows, in air, bis(4,4,5,5-tetramethyl-1,3,2-dioxaborolan-2-yl)methane (1.34 g, 5.0 mmol) was added to a Schlenk tube equipped with a stir bar. The vessel was evacuated and filled with argon (three cycles). THF (20.0 mL) and LDA (6.0 mmol, Acros) were added *via* syringe at 0°C. The reaction mixture was stirred for 5 min, and then 9-bromonon-1-ene (1.03 g, 5.0 mmol) was added in dropwise at this temperature. The reaction mixture was allowed to warm to room temperature and stirred for 2 h. The reaction mixture was then diluted with Et_2O and quenched by the NH_4Cl (aq). The organic layer was dried over Na_2SO_4 (s), filtered and concentrated cautiously in *vacuo*. The crude oil was purified by silica gel (7-10% EtOAc in petroleum ether, stained by $KMnO_4$ in water) to afford the desired product as a thick oil (2.74 g, 69% yield). **^{1}H NMR** (400 MHz, $CDCl_3$) δ 5.80 (m, 1H), 5.08 – 4.82 (m, 2H), 2.02 (dd, *J* = 13.6, 6.8 Hz, 2H), 1.62 – 1.48 (m, 2H), 1.45 – 1.25 (m, 8H), 1.23 (d, *J* = 3.6 Hz, 24H), 0.71 (t, *J* = 7.8 Hz, 1H). **^{13}C NMR** (101 MHz, $CDCl_3$) δ 139.32, 114.00, 82.87, 33.82, 32.49, 29.43, 29.02, 28.88, 25.66, 24.86, 24.51, 10.74. **IR**(neat): 2978 (s), 2927 (s), 2856 (m), 1465 (w), 1370 (s), 1315 (s), 1268 (m), 1214 (w), 1142 (s), 970 (m), 905 (w), 850 (m) cm^{-1}. **HRMS** Calcd for $C_{21}H_{40}B_2O_4Na$ $[M+Na]^+$ 401.3005; Found: 401.3007.

Synthesis of (*R*)-4-((*R*)-1-(benzyloxy)hex-5-en-1-yl)-2,2-dimethyl-1,3-dioxolane

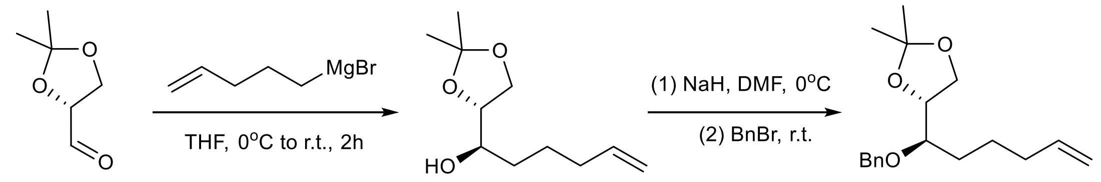

To a stirred solution of (*R*)-2,2-dimethyl-1,3-dioxolane-4-carbaldehyde (1.97 g, 15.0 mmol) in dry THF (20.0 mL) was added a solution of 4-pentenylmagnesium bromide prepared from magnesium powder (360.0 mg, 15.0 mmol) and 4-pentenyl bromide (1.18 g, 10.0 mmol) in dry THF (20.0 mL) at 0 ℃. After being stirred for 2h at room temperature, the mixture was cooled in an ice-bath, quenched by the addition of saturated NH_4C1 aqueous solution, and extracted with EtOAc. The organic phase was washed with brine, dried over Na_2SO_4, and evaporated. The crude product was purified by silica gel (20-25% EtOAc in petroleum ether, stained by $KMnO_4$ in water) to afford (*R*)-1-((*R*)-2,2-dimethyl-1,3-dioxolan-4-yl)hex-5-en-1-ol as a clear oil (840 mg, 42% yield) (*Larger polarity compared with its diastereoisomer generated in this reaction*). The obtained secondary alcohol (800 mg, 4.0 mmol) was dissolved in DMF (20.0 mL) and NaH (4.0 mmol, 60% dispersion in mineral oil) was added to the DMF solution. After stirring at room temperature for 0.5h, benzyl bromide (0.6 mL, 5.0 mmol) was added in dropwise and stirred for overnight. The reaction was quenched by saturated NH_4Cl solution, and extracted with EtOAc (20.0 mL × 3). The organic phase was then combined and washed with brine, dried over Na_2SO_4 and concentrated. Purified by silica gel (5-7% EtOAc in petroleum ether). (*R*)-4-((*R*)-1-(benzyloxy)hex-5-en-1-yl)-2,2-dimethyl-1,3-dioxolane was isolated as a clear oil (1.05 g, 90% yield).

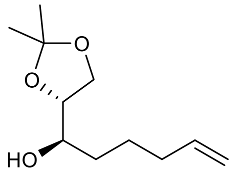

(*R*)-1-((*R*)-2,2-dimethyl-1,3-dioxolan-4-yl)hex-5-en-1-ol

^{1}H NMR (400 MHz, $CDCl_3$) δ 5.80 (m, 1H), 4.99 (dd, *J* = 21.5, 13.7 Hz, 2H), 4.20 – 3.93 (m, 2H), 3.90 (t, *J* = 7.3 Hz, 1H), 3.79 – 3.72 (m, 1H), 2.48 (s, 1H), 2.15 – 2.05 (m, 2H), 1.71 – 1.38 (m, 7H), 1.36 (s, 3H). **^{13}C NMR** (101 MHz, $CDCl_3$) δ 138.43, 114.82, 108.97, 78.74, 70.63, 64.70, 33.59, 32.13, 26.49, 25.30, 25.01. **IR**(neat): 3443 (br), 2986 (m), 2936 (s), 1639 (w), 1457 (w), 1371 (m), 1250 (m), 1216 (m), 1156 (w), 1068 (s), 912 (m), 855 (m) cm^{-1}. **HRMS** Calcd for $C_{11}H_{21}O_3$ [M+H]$^+$ 201.1485; Found: 201.1487. $[\alpha]_D^{20}$ = +11.13 (*c* = 1.0675, $CHCl_3$, *l* = 100 mm).

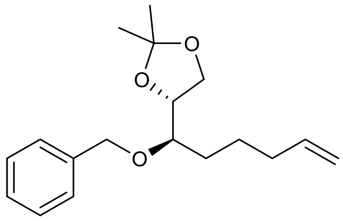

(*R*)-4-((*R*)-1-(benzyloxy)hex-5-en-1-yl)-2,2-dimethyl-1,3-dioxolane

^{1}H NMR (400 MHz, $CDCl_3$) δ 7.37 – 7.23 (m, 5H), 5.79 (m, 1H), 5.06 – 4.92 (m, 1H), 4.62 (q, *J* = 11.4 Hz, 2H), 4.10 (dd, *J* = 12.1, 6.3 Hz, 1H), 4.05 – 4.00 (m, 1H), 3.93 – 3.87 (m, 1H), 3.54 (dd, *J* = 9.2, 5.8 Hz, 1H), 2.05 (dd, *J* = 12.4, 6.0 Hz, 2H), 1.65 – 1.43 (m, 3H), 1.42 (s, 3H), 1.35 (s, 3H). **^{13}C NMR** (101 MHz, CDCl3) δ 138.57, 128.34, 127.80, 127.60, 114.66, 108.96, 78.86, 77.84, 72.80, 66.27, 33.76, 30.63, 26.59, 25.36, 24.19. (Signal for one carbon could not be located). **IR**(neat): 2935 (s), 2861 (m), 1721 (w), 1637 (w), 1455 (m), 1210 (w), 1074 (s), 912 (m), 737 (m), 698 (s) cm^{-1}. **HRMS** Calcd for $C_{18}H_{27}O_3$ [M+H]$^+$ 291.1955; Found: 291.1958. $[\alpha]_D^{20}$ = +6.63 (*c* = 1.000, $CHCl_3$, *l* = 100 mm).

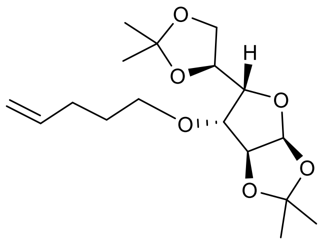

(3*aS*,5*S*,6*R*,6*aS*)-5-((*S*)-2,2-dimethyl-1,3-dioxolan-4-yl)-2,2-dimethyl-6-(pent-4-en-1-yloxy)tetrahydrofuro[2,3-d][1,3]dioxole (38)

Prepared according to the general procedure B using diacetone-D-glucose (5.2 g, 20.0 mmol), NaH (880 mg, 22.0 mmol, 60% dispersion in mineral oil) and 5-bromo-1-pentene (3.55 mL, 30.0 mmol) in DMF (100 mL). The reaction was quenched by glacial water and then extracted with CH_2Cl_2 (50 mL × 3). The organic phase was combined and dried over Na_2SO_4, followed by the usual work-up and purified on silica gel (10-15% EtOAc in petroleum, stained by $KMnO_4$ in water) to afford the product as a thick oil (4.8 g, 73% yield). **^{1}H NMR** (400 MHz, $CDCl_3$) δ 5.87 (d, *J* = 3.7 Hz, 1H), 5.80 (m, 1H), 5.08 – 4.94 (m, 2H), 4.53 (d, *J* = 3.7 Hz, 1H), 4.31 (dd, *J* = 13.6, 6.1 Hz, 1H), 4.17 – 4.05 (m, 2H), 3.98

(dd, J = 8.5, 5.9 Hz, 1H), 3.85 (d, J = 3.1 Hz, 1H), 3.62 (dt, J = 9.3, 6.3 Hz, 1H), 3.52 (dt, J = 9.3, 6.3 Hz, 1H), 2.18 – 2.09 (m, 2H), 1.73 – 1.61 (m, 2H), 1.50 (s, 3H), 1.42 (s, 3H), 1.35 (s, 3H), 1.32 (s, 3H). **^{13}C NMR** (101 MHz, $CDCl_3$) δ 138.06, 137.85, 115.20, 114.98, 111.73, 108.91, 105.27, 105.05, 82.90, 82.45, 82.10, 81.98, 81.19, 79.82, 72.48, 69.71, 69.58, 69.47, 67.24, 64.41, 30.94, 30.18, 30.13, 28.84, 28.78, 26.83, 26.77, 26.73, 26.24, 26.20, 25.38. **IR**(neat): 2983 (m), 2936 (s), 2870 (m), 1639 (w), 1447 (w), 1373 (s), 1255 (m), 1217 (m), 1165 (m), 1124 (m), 1081 (s), 1025 (s), 912 (m), 847 (w), 639 (w) cm^{-1}. **HRMS** Calcd for $C_{17}H_{29}O_6$ $[M+H]^+$ 329.1959; Found: 329.1956. $[\alpha]_D^{20}$ = -17.13 (c = 0.7683, $CHCl_3$, l = 100 mm).

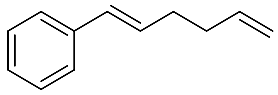

(*E*)-hexa-1,5-dien-1-ylbenzene

To a stirred solution of cinnamyl bromide (2.0 g, 11.5 mmol) in THF (50 mL) was added slowly allylmagnesium bromide (15.0 mL, 1.0 M in ether) and the reaction mixture was stirred at room temperature for 2h. The reaction was quenched with saturated NH_4Cl solution. The mixture was extracted with EtOAc (30 mL × 2). The combined organic layers were dried over Na_2SO_4 and concentrated. Purification of the crude oil by silica gel (petroleum ether), the corresponding product was obtained as a clear oil (1.33 g, 73 % yield). **^{1}H NMR** (400 MHz, $CDCl_3$) δ 7.38 – 7.23 (m, 4H), 7.17 (t, J = 7.2 Hz, 1H), 6.39 (d, J = 15.9 Hz, 1H), 6.21 (dt, J = 15.8, 6.6 Hz, 1H), 5.85 (m, 1H), 5.02 (m, 2H), 2.35 – 2.17 (m, 4H). **^{13}C NMR** (101 MHz, $CDCl_3$) δ 138.08, 137.74, 130.19, 130.08, 128.46, 126.87, 125.95, 114.92, 33.54, 32.41. **IR**(neat): 3080 (m), 3026 (s), 2978 (m), 2925 (s), 2844 (w), 1641 (m), 1597 (w), 1495 (m), 1448 (m), 965 (s), 912 (s), 741 (s), 692 (s) cm^{-1}.

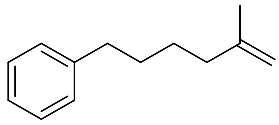

(5-methylhex-5-en-1-yl)benzene (25)

(3-bromopropyl)benzene (2.0 g, 10.0 mmol) and anhydrous THF (30.0 mL) were placed in a 50 mL flask under argon. (2-methylallyl)magnesium bromide (1.1 equiv., 11.0 mmol) in THF was added dropwise at 0 °C for 0.5h . Stirring at 0 ℃ for another 2h, the reaction mixture was then quenched with saturated NH_4Cl solution. The mixture was extracted with EtOAc (30 mL × 2). The combined organic layers were dried over Na_2SO_4 and concentrated. Purification of the crude oil by silica gel (petroleum ether), the corresponding product **25** was obtained as a clear oil (1.24 g, 71 % yield). **^{1}H NMR** (400 MHz, $CDCl_3$) δ 7.25 (dd, J = 9.7, 5.4 Hz, 2H), 7.14 (dd, J = 10.0, 4.5 Hz, 3H), 4.67 (d, J = 10.8 Hz, 2H), 2.64 – 2.58 (m, 2H), 2.03 (t, J = 7.6 Hz, 2H), 1.69 (s, 3H), 1.65 – 1.55 (m, 2H), 1.47 (dt, J = 15.4, 7.5 Hz, 2H). **^{13}C NMR** (101 MHz, $CDCl_3$) δ 145.86, 142.70, 128.40, 128.24, 125.61, 109.86, 37.63, 35.85, 31.11, 27.21, 22.33. **IR**(neat): 3027 (w), 2933 (s), 2857 (m), 1599 (w), 1495 (w), 1453 (m), 1370 (w), 887 (m), 745 (m), 698 (s) cm^{-1}.

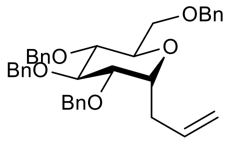

(2*R*,3*S*,4*R*,5*R*,6*R*)-2-allyl-3,4,5-tris(benzyloxy)-6-((benzyloxy)methyl)tetrahydro-2*H*-pyran (35)

This substrate was prepared as follows, tetrabenzyl-D-glucose (**34**, 2.7 g, 5.0 mmol) and anhydrous THF (40.0 mL) were placed in a 100 mL flask under argon. DAST (5.5 mmol, 0.73 mL) was added at -30°C. The reaction was then allowed to stir at room temperature for 1h. The flask was cooled to -30°C and methanol (0.5 ml) was added to quench the reaction. Followed by a usual work-up, afforded (2*R*,3*R*,4*S*,5*R*)-3,4,5-tris(benzyloxy)-2-((benzyloxy)methyl)-6-fluorotetrahydro-2*H*-pyran in 99% yield. The mixture of fluorides was dissovled in DCM (40.0 mL) at 0 °C and allytrimethylsilane (2.0 equiv.) and $BF_3{\cdot}Et_2O$ (0.2 equiv.) were added. After 2h, TLC indicated complete reaction. Followed by the normal work-up, **35** was obtained as a white solid (2.2 g, 78% yield). ^{1}**H NMR** (400 MHz, $CDCl_3$) δ 7.36 – 7.20 (m, 20H), 5.82 (m, 1H), 5.09 (t, *J* = 14.0 Hz, 2H), 4.93 (d, *J* = 11.0 Hz, 1H), 4.81 (dd, *J* = 10.8, 3.0 Hz, 2H), 4.65 (dd, *J* = 26.7, 11.6 Hz, 3H), 4.46 (dd, *J* = 11.4, 5.1 Hz, 2H), 4.13 (dt, *J* = 11.3, 5.8 Hz, 1H), 3.83 – 3.75 (m, 2H), 3.70 (dt, *J* = 9.1, 4.7 Hz, 1H), 3.62 (t, *J* = 9.2 Hz, 3H), 2.57 – 2.41 (m, 2H). ^{13}**C NMR** (101 MHz, $CDCl_3$) δ 138.77, 138.24, 138.20, 138.07, 134.76, 128.44, 128.41, 128.35, 128.00, 127.96, 127.92, 127.90, 127.84, 127.80, 127.75, 127.64, 127.61, 116.93, 82.41, 80.05, 78.09, 75.46, 75.10, 73.70, 73.46, 73.07, 71.11, 68.89, 29.82. **IR**(neat): 3034 (w), 2911 (m), 2866 (m), 1495 (m), 1454 (s), 1360 (m), 1208 (w), 1092 (s), 1028 (m), 909 (w), 736 (s), 697 (s) cm^{-1}. **HRMS** Calcd for $C_{37}H_{41}O_5$ $[M+H]^+$ 565.2949; Found: 565.2951. $[\alpha]_D^{20}$ = +30.48 (*c* = 1.1750, $CHCl_3$, *l* = 100 mm).

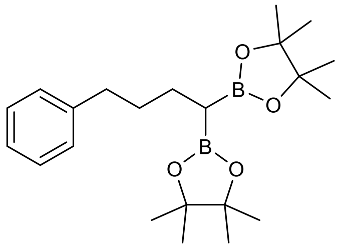

2,2'-(4-phenylbutane-1,1-diyl)bis(4,4,5,5-tetramethyl-1,3,2-dioxaborolane) (2)

Prepared according to the general procedure D using 4-phenyl-1-butene (30.0 μL, 0.2 mmol) and B_2pin_2 (0.4 mmol, 101.6 mg) with 5% $Ni(COD)_2$ (2.8 mg), 5% Cy-XantPhos (6.0 mg) and LiOMe (0.2 mmol, 7.6 mg). The crude reaction mixture was purified on silica gel (7-10% EtOAc/petroleum ether) to afford the product as a viscous oil (45.6 mg, 59% yield). ^{1}**H NMR** (400 MHz, $CDCl_3$) δ 7.25 (t, *J* = 7.6 Hz, 2H), 7.19 – 7.11 (m, 3H), 2.59 (t, *J* = 6.7 Hz, 2H), 1.68 – 1.57 (m, 4H), 1.22 (d, *J* = 3.7 Hz, 24H), 0.76 (t, *J* = 6.2 Hz, 1H). ^{13}**C NMR** (101 MHz, $CDCl_3$) δ 143.01, 128.33, 128.15, 125.41, 82.93, 36.03, 34.34, 25.56, 24.87, 24.52, 10.56. **IR**(neat): 2979 (s), 2929 (m), 2853 (w), 1457 (m), 1366 (s), 1308 (s), 1270 (m), 1213 (m), 1136 (s), 971 (s), 849 (m), 735 (s), 698 (s), 669 (m) cm^{-1}. **HRMS** Calcd for $C_{22}H_{37}B_2O_4$ $[M+H]^+$ 387.2872; Found: 387.2879.

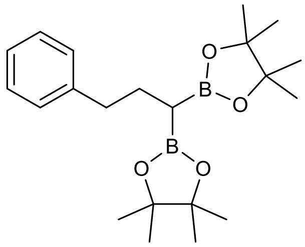

2,2'-(3-phenylpropane-1,1-diyl)bis(4,4,5,5-tetramethyl-1,3,2-dioxaborolane) (3)

Prepared according to the general procedure D using allylbenzene (26.5 μL, 0.2 mmol) and B_2pin_2 (0.4 mmol, 101.6 mg) with 5% $Ni(COD)_2$ (2.8 mg), 5% Cy-XantPhos (6.0 mg) and LiOMe (0.2 mmol, 7.6 mg). The crude reaction mixture was purified on silica gel (7-10% EtOAc/petroleum ether) to afford the product as a white solid (44.6 mg, 60% yield). **^{1}H NMR** (400 MHz, $CDCl_3$) δ 7.27 – 7.09 (m, 5H), 2.59 (dd, *J* = 9.1, 6.9 Hz, 2H), 1.92 – 1.80 (m, 2H), 1.23 (d, *J* = 3.4 Hz, 24H), 0.81 (t, *J* = 8.0 Hz, 1H). **^{13}C NMR** (101 MHz, $CDCl_3$) δ 142.92, 128.57, 128.10, 125.47, 82.98, 77.38, 77.07, 76.75, 38.72, 27.98, 24.91, 24.52, 10.55. **IR**(neat): 2978 (s), 2931 (m), 1455 (m), 1370 (s), 1315 (s), 1264 (m), 1216 (m), 1140 (s), 969 (s), 850 (s), 749 (m), 700 (s) cm^{-1}. **HRMS** Calcd for $C_{21}H_{35}B_2O_4$ $[M+H]^+$ 373.2716; Found: 373.2723.

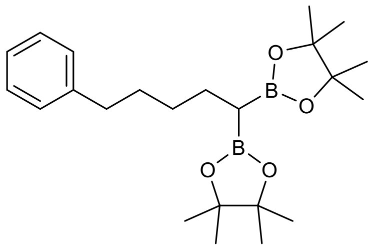

2,2'-(5-phenylpentane-1,1-diyl)bis(4,4,5,5-tetramethyl-1,3,2-dioxaborolane) (4)

Prepared according to the general procedure D using allylbenzene (29.2 mg, 0.2 mmol) and B_2pin_2 (0.4 mmol, 101.6 mg) with 5% $Ni(COD)_2$ (2.8 mg), 5% Cy-XantPhos (6.0 mg) and LiOMe (0.2 mmol, 7.6 mg). The crude reaction mixture was purified on silica gel (7-10% EtOAc/petroleum ether) to afford the product as a viscous oil (49.6 mg, 62% yield). **^{1}H NMR** (400 MHz, $CDCl_3$) δ 7.26 – 7.22 (m, 2H), 7.18 – 7.12 (m, 3H), 2.58 (t, *J* = 7.6 Hz, 2H), 1.60 (m, 2H), 1.37 – 1.30 (m, 2H), 1.20 (d, *J* = 6.5 Hz, 24H), 0.72 (t, *J* = 7.8 Hz, 1H). **^{13}C NMR** (101 MHz, $CDCl_3$) δ 142.94, 128.43, 128.12, 125.42, 82.88, 35.78, 32.08, 31.35, 25.54, 24.83, 24.51, 10.63. **IR**(neat): 2982 (s), 2930 (s), 2858 (m), 1455 (m), 1370 (s), 1315 (s), 1268 (s), 1215 (m), 1141 (s), 970 (s), 850 (s), 747 (m), 699 (s) cm^{-1}. **HRMS** Calcd for $C_{23}H_{39}B_2O_4$ $[M+H]^+$ 401.3029; Found: 401.3037.

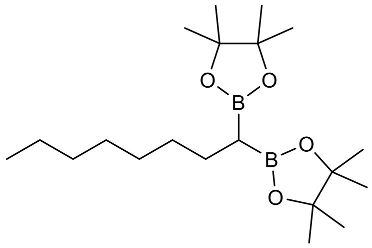

2,2'-(octane-1,1-diyl)bis(4,4,5,5-tetramethyl-1,3,2-dioxaborolane) (5)

Prepared according to the general procedure D using 1-octene (31.4 μL, 0.2 mmol) and B_2pin_2 (0.4 mmol, 101.6 mg) with 5% $Ni(COD)_2$ (2.8 mg), 5% Cy-XantPhos (6.0 mg) and LiOMe (0.2 mmol, 7.6 mg). The crude reaction mixture was purified on silica gel (7-10% EtOAc/petroleum ether, stained by $KMnO_4$ in water) to afford the product as a viscous oil (41.7 mg, 57% yield). **^{1}H NMR** (400 MHz, $CDCl_3$) δ 1.54 – 1.51 (m, 2H), 1.25 (m, 10H), 1.23 (d, *J* = 3.7 Hz, 24H), 0.86 (t, *J* = 6.8 Hz, 3H), 0.71 (t, *J* = 7.8 Hz, 1H). **^{13}C NMR** (101 MHz, $CDCl_3$) δ 82.86, 32.59, 31.86, 29.58, 29.22, 25.69, 24.86, 24.53, 22.67, 14.13, 10.94. **IR**(neat): 2980 (s), 2926 (s), 2856 (m), 1467 (m), 1370 (s), 1314 (s), 1268 (m), 1215 (m), 1143 (s), 970 (s), 850 (s), 670 (w), 579 (w) cm^{-1}. **HRMS** Calcd for $C_{20}H_{41}B_2O_4$ $[M+H]^+$ 367.3185; Found: 367.3185.

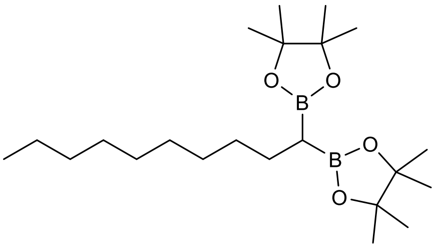

2,2'-(decane-1,1-diyl)bis(4,4,5,5-tetramethyl-1,3,2-dioxaborolane) (6)

Prepared according to the general procedure D using 1-*n*-decene (37.8 µL, 0.2 mmol) and B_2pin_2 (0.4 mmol, 101.6 mg) with 5% $Ni(COD)_2$ (2.8 mg), 5% Cy-XantPhos (6.0 mg) and LiOMe (0.2 mmol, 7.6 mg). The crude reaction mixture was purified on silica gel (7-10% EtOAc/petroleum ether, stained by $KMnO_4$ in water) to afford the product as a viscous oil (49.7 mg, 63% yield). **^{1}H NMR** (400 MHz, $CDCl_3$) δ 1.54 – 1.51 (m, 2H), 1.25 (m, 14H), 1.23 (d, J = 3.7 Hz, 24H), 0.87 (t, J = 6.7 Hz, 3H), 0.71 (t, J = 7.8 Hz, 1H). **^{13}C NMR** (101 MHz, $CDCl_3$) δ 82.87, 32.59, 31.96, 29.62, 29.59, 29.57, 29.35, 25.70, 24.86, 24.53, 22.71, 14.12, 10.78. **IR**(neat): 2978 (s), 2926 (s), 2855 (m), 1467 (m), 1370 (s), 1314 (s), 1268 (m), 1215 (m), 1142 (s), 970 (s), 850 (s), 671 (w) cm^{-1}. **HRMS** Calcd for $C_{22}H_{45}B_2O_4$ $[M+H]^+$ 395.3498; Found: 395.3499.

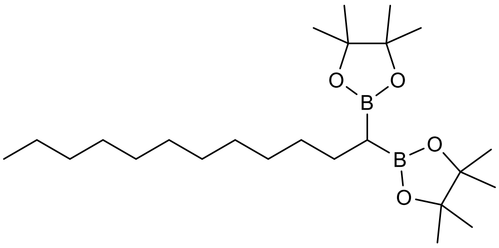

2,2'-(dodecane-1,1-diyl)bis(4,4,5,5-tetramethyl-1,3,2-dioxaborolane) (7)

Prepared according to the general procedure D using dodecylene (44.4 µL, 0.2 mmol) and B_2pin_2 (0.4 mmol, 101.6 mg) with 5% $Ni(COD)_2$ (2.8 mg), 5% Cy-XantPhos (6.0 mg) and LiOMe (0.2 mmol, 7.6 mg). The crude reaction mixture was purified on silica gel (7-10% EtOAc/petroleum ether, stained by $KMnO_4$ in water) to afford the product as a viscous oil (49.8 mg, 59% yield). **^{1}H NMR** (400 MHz, $CDCl_3$) δ 1.54 – 1.51 (m, 2H), 1.24 (m, 18H), 1.23 (d, J = 3.7 Hz, 24H), 0.88 (t, J = 6.9 Hz, 3H), 0.71 (t, J = 7.9 Hz, 1H). **^{13}C NMR** (101 MHz, $CDCl_3$) δ 82.88, 32.59, 31.95, 29.69, 29.63, 29.57, 29.38, 25.70, 24.87, 24.54, 22.70, 14.12, 10.83. (Signals for two carbons could not be located). **IR**(neat): 2978 (s), 2928 (s), 2854 (s), 1467 (m), 1370 (s), 1314 (s), 1268 (m), 1216 (m), 1142 (s), 970 (m), 850 (m), 673 (w) cm^{-1}. **HRMS** Calcd for $C_{24}H_{48}B_2O_4Na$ $[M+Na]^+$ 445.3631; Found: 445.3633.

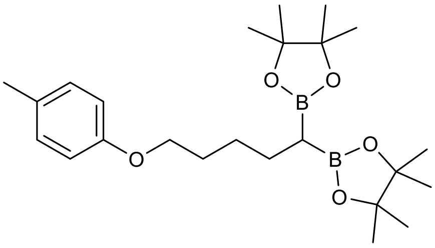

2,2'-(5-(*p*-tolyloxy)pentane-1,1-diyl)bis(4,4,5,5-tetramethyl-1,3,2-dioxaborolane) (8)

Prepared according to the general procedure D using 1-methyl-4-(pent-4-en-1-yloxy)benzene (35.3 mg, 0.2 mmol) and B_2pin_2 (0.4 mmol, 101.6 mg) with 5% $Ni(COD)_2$ (2.8 mg), 5% Cy-XantPhos (6.0 mg)

and LiOMe (0.2 mmol, 7.6 mg). The crude reaction mixture was purified on silica gel (7-10% EtOAc/petroleum ether) to afford the product as a viscous oil (61.9 mg, 72% yield). **^{1}H NMR** (400 MHz, $CDCl_3$) δ 7.05 (d, *J* = 8.2 Hz, 2H), 6.77 (d, *J* = 8.3 Hz, 2H), 3.90 (t, *J* = 6.5 Hz, 2H), 2.27 (s, 3H), 1.79 – 1.72 (m, 2H), 1.64 – 1.58 (dd, *J* = 15.5, 7.8 Hz, 2H), 1.48-1.40 (dt, *J* = 15.2, 7.7 Hz, 2H), 1.23 (d, *J* = 3.8 Hz, 24H), 0.75 (t, *J* = 7.8 Hz, 1H). **^{13}C NMR** (101 MHz, $CDCl_3$) δ 157.05, 129.77, 129.46, 114.38, 82.96, 67.97, 29.34, 28.84, 25.44, 24.86, 24.52, 20.44, 10.64. **IR**(neat): 2978 (s), 2931 (m), 2864 (w), 1613 (w), 1512 (s), 1469 (w), 1370 (s), 1315 (s), 1266 (m), 1246 (s), 1216 (w), 1141 (s), 970 (m), 850 (m), 817 (w) cm^{-1}. **HRMS** Calcd for $C_{24}H_{41}B_2O_5$ $[M+H]^+$ 431.3135; Found: 431.3141.

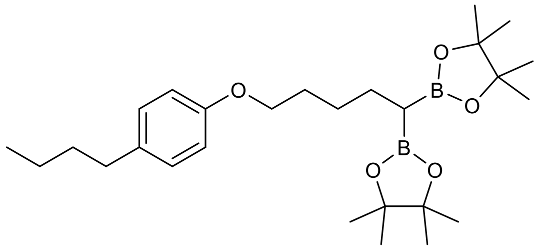

2,2'-(5-(4-butylphenoxy)pentane-1,1-diyl)bis(4,4,5,5-tetramethyl-1,3,2-dioxaborolane) (9)

Prepared according to the general procedure D using 1-butyl-4-(pent-4-en-1-yloxy)benzene (43.7 mg, 0.2 mmol) and B_2pin_2 (0.4 mmol, 101.6 mg) with 5% $Ni(COD)_2$ (2.8 mg), 5% Cy-XantPhos (6.0 mg) and LiOMe (0.2 mmol, 7.6 mg). The crude reaction mixture was purified on silica gel (7-10% EtOAc/petroleum ether) to afford the product as a viscous oil (61.4 mg, 65% yield). **^{1}H NMR (**400 MHz, $CDCl_3$) δ 7.05 (d, *J* = 8.4 Hz, 2H), 6.78 (d, *J* = 8.5 Hz, 2H), 3.90 (t, *J* = 6.6 Hz, 2H), 2.53 (t, *J* = 7.7 Hz, 2H), 1.79 – 1.72 (m, 2H), 1.58 (m, 4H), 1.48 – 1.41 (m, 2H), 1.33 (m, 2H), 1.22 (d, *J* = 4.0 Hz, 24H), 0.91 (t, *J* = 7.3 Hz, 3H), 0.75 (t, *J* = 7.8 Hz, 1H). **^{13}C NMR** (101 MHz, $CDCl_3$) δ 157.17, 134.63, 129.13, 114.25, 82.95, 67.88, 34.73, 33.92, 29.35, 28.85, 25.44, 24.86, 24.51, 22.29, 13.97, 10.71. **IR**(neat): 2977 (s), 2930 (s), 2860 (m), 1613 (w), 1512 (s), 1468 (m), 1371 (s), 1316 (s), 1266 (m), 1245 (s), 1216 (w), 1142 (s), 970 (m), 850 (m), 829 (w) cm^{-1}. **HRMS** Calcd for $C_{27}H_{46}B_2O_5Na$ $[M+Na]^+$ 495.3424; Found: 495.3423.

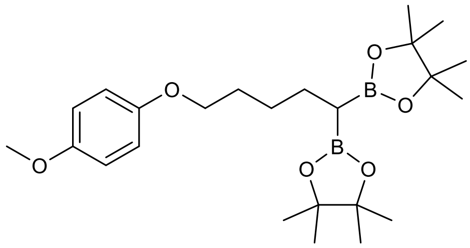

2,2'-(5-(4-methoxyphenoxy)pentane-1,1-diyl)bis(4,4,5,5-tetramethyl-1,3,2-dioxaborolane) (10)

Prepared according to the general procedure D using 1-methoxy-4-(pent-4-en-1-yloxy)benzene (38.5 mg, 0.2 mmol) and B_2pin_2 (0.4 mmol, 101.6 mg) with 5% $Ni(COD)_2$ (2.8 mg), 5% Cy-XantPhos (6.0 mg) and LiOMe (0.2 mmol, 7.6 mg). The crude reaction mixture was purified on silica gel (7-10% EtOAc/petroleum ether) to afford the product as a viscous oil (63.3 mg, 71% yield). **^{1}H NMR** (400 MHz, $CDCl_3$) δ 6.81 (s, 4H), 3.88 (t, *J* = 6.5 Hz, 2H), 3.75 (s, 3H), 1.83 – 1.69 (m, 2H), 1.66 – 1.58 (m, 2H), 1.48 – 1.40 (m, 2H), 1.23 (d, *J* = 3.9 Hz, 24H), 0.75 (t, *J* = 7.8 Hz, 1H). **^{13}C NMR** (101 MHz, $CDCl_3$) δ 153.56, 153.34, 115.40, 114.55, 82.94, 68.51, 55.71, 29.36, 28.81, 25.43, 24.85, 24.50, 10.71. **IR**(neat): 2978 (s), 2933 (m), 2866 (w), 1508 (s), 1468 (w), 1370 (s), 1315 (s), 1266 (m), 1232 (s), 1141 (s), 1041 (m), 970 (m), 850 (m), 825 (m) cm^{-1}. **HRMS** Calcd for $C_{24}H_{40}B_2O_6Na$ $[M+Na]^+$

469.2903; Found: 469.2903.

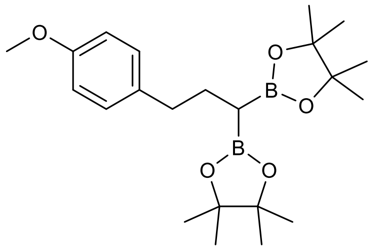

2,2'-(3-(4-methoxyphenoxy)propane-1,1-diyl)bis(4,4,5,5-tetramethyl-1,3,2-dioxaborolane) (11)

Prepared according to the general procedure D using 1-(allyloxy)-4-methoxybenzene (32.8 mg, 0.2 mmol) and B_2pin_2 (0.4 mmol, 101.6 mg) with 5% $Ni(COD)_2$ (2.8 mg), 5% Cy-XantPhos (6.0 mg) and LiOMe (0.2 mmol, 7.6 mg). The crude reaction mixture was purified on silica gel (7-10% EtOAc/petroleum ether) to afford the product as a viscous oil (53.5 mg, 72% yield). **^{1}H NMR** (400 MHz, $CDCl_3$) δ 7.09 (d, *J* = 8.4 Hz, 2H), 6.79 (d, *J* = 8.4 Hz, 2H), 3.77 (s, 3H), 2.55 – 2.51 (m, 2H), 1.84 – 1.78 (m, 2H), 1.23 (d, *J* = 3.4 Hz, 24H), 0.79 (t, *J* = 7.9 Hz, 1H). **^{13}C NMR** (101 MHz, $CDCl_3$) δ 157.59, 135.10, 129.44, 113.58, 82.98, 55.24, 37.79, 28.19, 24.90, 24.52, 10.38. **IR**(neat): 2976 (s), 2924 (s), 1611 (w), 1512 (m), 1456 (w), 1361 (m), 1316 (s), 1264 (m), 1245 (m), 1138 (s), 1032 (m), 970 (m), 851 (m), 815 (m) cm^{-1}. **HRMS** Calcd for $C_{22}H_{36}B_2O_5Na$ $[M+Na]^+$ 425.2641; Found: 425.2644.

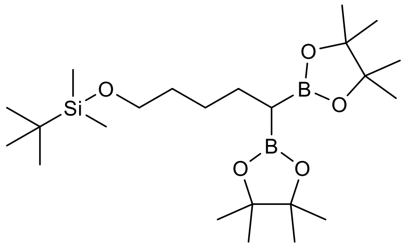

((5,5-bis(4,4,5,5-tetramethyl-1,3,2-dioxaborolan-2-yl)pentyl)oxy)(*tert*-butyl)dimethylsilane (12)

Prepared according to the general procedure D using *tert*-butyldimethyl(pent-4-en-1-yloxy)silane (40.0 mg, 0.2 mmol) and B_2pin_2 (0.4 mmol, 101.6 mg) with 5% $Ni(COD)_2$ (2.8 mg), 5% Cy-XantPhos (6.0 mg) and LiOMe (0.2 mmol, 7.6 mg). The crude product was then purified on silica gel (7-10% EtOAc/petroleum ether, stained by $KMnO_4$ in water) to afford **12** as a viscous oil (58.2 mg, 64% yield). **^{1}H NMR** (400 MHz, $CDCl_3$) δ 3.55 (t, *J* = 6.6 Hz, 2H), 1.56 – 1.43 (m, 4H), 1.32 – 1.23 (m, 4H), 1.19 (d, *J* = 3.5 Hz, 24H), 0.85 (s, 9H), 0.69 (t, *J* = 7.8 Hz, 1H), 0.00 (s, 6H). **^{13}C NMR** (101 MHz, $CDCl_3$) δ 82.87, 63.42, 33.05, 28.84, 26.00, 25.60, 24.85, 24.53, 18.34, 10.84, -5.25. **IR**(neat): 2930 (s), 2858 (s), 1471 (m), 1314 (s), 1265 (s), 1215 (m), 1142 (s), 1102 (m), 1006 (w), 970 (m), 837 (s), 776 (m), 669 (m), 579 (w) cm^{-1}. **HRMS** Calcd for $C_{23}H_{49}B_2O_5Si$ $[M+H]^+$ 455.3530; Found: 455.3528.

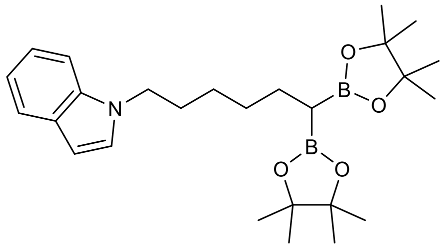

1-(6,6-bis(4,4,5,5-tetramethyl-1,3,2-dioxaborolan-2-yl)hexyl)-1*H*-indole (13)

Prepared according to the general procedure D using 1-(hex-5-en-1-yl)-1*H*-indole (39.9 mg, 0.2 mmol) and B_2pin_2 (0.4 mmol, 101.6 mg) with 5% $Ni(COD)_2$ (2.8 mg), 5% Cy-XantPhos (6.0 mg) and LiOMe (0.2 mmol, 7.6 mg). The crude reaction mixture was purified on silica gel (12-15% EtOAc/petroleum ether) to afford the product as a white solid (63.4 mg, 70% yield). **^{1}H NMR** (400 MHz, $CDCl_3$) δ 7.61 (d, *J* = 7.8 Hz, 1H), 7.33 (d, *J* = 8.3 Hz, 1H), 7.18 (t, *J* = 7.6 Hz, 1H), 7.08 (m, 2H), 6.46 (d, *J* = 2.8 Hz, 1H), 4.08 (t, *J* = 7.2 Hz, 2H), 1.81 (m, 2H), 1.54 (m, 2H), 1.32 (m, 4H), 1.22 (d, *J* = 3.9 Hz, 24H), 0.71 (t, *J* = 7.7 Hz, 1H). **^{13}C NMR** (101 MHz, $CDCl_3$) δ 135.92, 128.53, 127.80, 121.20, 120.85, 119.05, 109.39, 100.74, 82.94, 46.26, 31.94, 30.02, 26.92, 25.46, 24.85, 24.51, 10.66. **IR**(neat): 2977 (m), 2930 (m), 1464 (m), 1370 (s), 1315 (s), 1268 (w), 1140 (s), 965 (m), 849 (m), 740 (m) cm^{-1}. **HRMS** Calcd for $C_{26}H_{42}B_2NO_4$ $[M+H]^+$ 454.3294; Found: 454.3301.

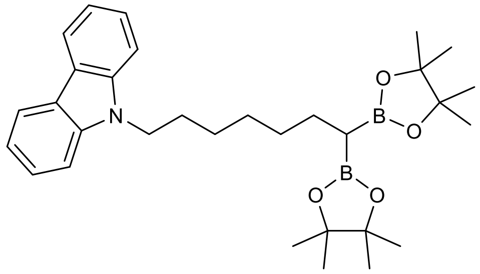

9-(7,7-bis(4,4,5,5-tetramethyl-1,3,2-dioxaborolan-2-yl)heptyl)-9*H*-carbazole (14)

Prepared according to the general procedure D using 9-(hept-6-en-1-yl)-9*H*-carbazole (52.7 mg, 0.2 mmol) and B_2pin_2 (0.4 mmol, 101.6 mg) with 5% $Ni(COD)_2$ (2.8 mg), 5% Cy-XantPhos (6.0 mg) and LiOMe (0.2 mmol, 7.6 mg). The crude reaction mixture was purified on silica gel (12-15% EtOAc/petroleum ether) to afford the product as a white solid (49.5 mg, 48% yield). **^{1}H NMR** (400 MHz, $CDCl_3$) δ 8.09 (d, *J* = 7.7 Hz, 2H), 7.45 (t, *J* = 7.5 Hz, 3H), 7.39 (d, *J* = 8.1 Hz, 2H), 7.21 (t, *J* = 7.4 Hz, 2H), 4.26 (t, *J* = 7.3 Hz, 2H), 1.82 (m, 2H), 1.54 (m, 2H), 1.34 (m, 6H), 1.21 (d, *J* = 5.1 Hz, 24H), 0.70 (t, *J* = 7.8 Hz, 1H). **^{13}C NMR** (101 MHz, $CDCl_3$) δ 140.38, 125.53, 122.75, 120.26, 118.61, 108.64, 82.90, 43.06, 32.40, 32.40, 29.39, 28.91, 27.16, 25.61, 24.85, 24.49, 10.73. **IR**(neat): 2977 (m), 2929 (m), 2856 (w), 1597 (w), 1485 (m), 1464 (m), 1453 (m), 1370 (s), 1348 (s), 1325 (s), 1267 (w), 1248 (w), 1140 (s), 969 (m), 850 (m), 750 (s), 723 (m) cm^{-1}. **HRMS** Calcd for $C_{31}H_{45}B_2NO_4Na$ $[M+Na]^+$ 540.3427; Found: 540.3429.

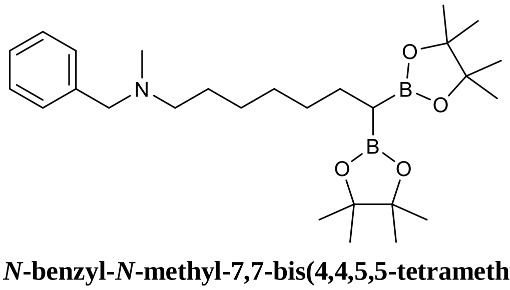

***N*-benzyl-*N*-methyl-7,7-bis(4,4,5,5-tetramethyl-1,3,2-dioxaborolan-2-yl)heptan-1-amine (15)**

Prepared according to the general procedure D using *N*-benzyl-*N*-methylhept-6-en-1-amine (43.5 mg, 0.2 mmol) and B_2pin_2 (0.4 mmol, 101.6 mg) with 5% $Ni(COD)_2$ (2.8 mg), 5% Cy-XantPhos (6.0 mg) and LiOMe (0.2 mmol, 7.6 mg). The crude reaction mixture was purified on silica gel (20% EtOAc in petroleum ether) to afford the product as a thick oil (63.4 mg, 67% yield). **^{1}H NMR** (400 MHz, $CDCl_3$) δ 7.31 – 7.24 (m, 5H), 3.51 (s, 2H), 2.53 – 2.30 (m, 2H), 2.19 (s, 3H), 1.53 (m, 4H), 1.27 (m, 6H), 1.22 (d, *J* = 3.6 Hz, 24H), 0.71 (t, *J* = 7.8 Hz, 1H). **^{13}C NMR** (101 MHz, $CDCl_3$) δ 138.44, 129.24, 128.20,

127.02, 82.87, 62.01, 57.38, 41.92, 32.51, 29.54, 27.35, 27.01, 25.62, 24.85, 24.51, 10.74. **IR**(neat): 2977 (s), 2929 (s), 2856 (m), 2788 (w), 1456 (m), 1370 (s), 1316 (s), 1268 (m), 1215 (w), 1142 (s), 970 (m), 850 (m), 741 (w), 695 (w), 673 (w) cm^{-1}. **HRMS** Calcd for $C_{27}H_{48}B_2NO_4$ $[M+H]^+$ 472.3764; Found: 472.3767.

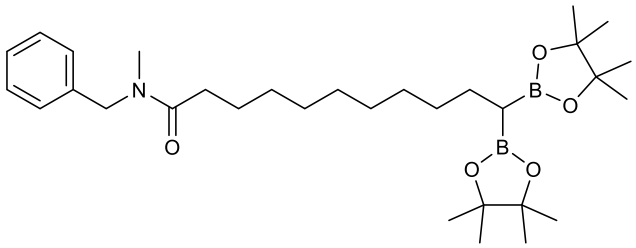

***N*-benzyl-*N*-methyl-11,11-bis(4,4,5,5-tetramethyl-1,3,2-dioxaborolan-2-yl)undecanamide (16)**

Prepared according to the general procedure D using *N*-benzyl-*N*-methylundec-10-enamide (57.5 mg, 0.2 mmol) and B_2pin_2 (0.4 mmol, 101.6 mg) with 5% $Ni(COD)_2$ (2.8 mg), 5% Cy-XantPhos (6.0 mg) and LiOMe (0.2 mmol, 7.6 mg). The crude reaction mixture was purified on silica gel (20% EtOAc in petroleum ether) to afford the product as a thick oil (60.9 mg, 56% yield). (mixture of two rotational isomers, the ratio of major/minor is 3/2). **^{1}H NMR** (400 MHz, $CDCl_3$) δ 7.27 (m, 5H), 4.56 (d, *J* = 22.8 Hz, 2H), 2.92 (d, *J* = 10.6 Hz, 3H), 2.37 (m, 2H), 1.62 (m, 4H), 1.48 – 1.28 (m, 12H), 1.25 (d, *J* = 8.3 Hz, 2H), 0.79 – 0.73 (m, 1H). **^{13}C NMR** (101 MHz, $CDCl_3$) δ 173.68, 173.31, 154.75, 137.60, 136.81, 128.90, 128.54, 128.01, 127.55, 127.25, 126.29, 82.97 (s), 82.84 (d, *J* = 4.2 Hz), 53.39, 50.74, 35.80, 34.83, 33.86, 33.59, 33.17, 32.42, 30.19 – 28.67 (m), 28.20, 25.67, 25.44 (d, *J* = 4.0 Hz), 25.19 (d, *J* = 3.4 Hz), 24.99 – 24.69 (m), 24.52, 23.99. **IR**(neat): 2977 (m), 2926 (s), 2854 (m), 1650 (s), 1453 (m), 1399 (m), 1362 (s), 1320 (s), 1267 (w), 1145 (s), 973 (w), 849 (w), 731 (w), 699 (w) cm^{-1}. **HRMS** Calcd for $C_{31}H_{54}B_2NO_5$ $[M+H]^+$ 542.4183; Found: 542.4186.

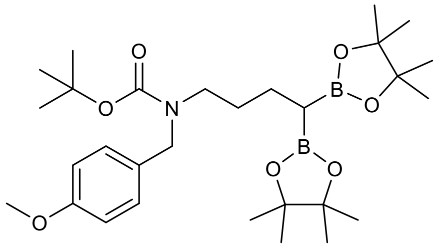

***tert*-butyl(4,4-bis(4,4,5,5-tetramethyl-1,3,2-dioxaborolan-2-yl)butyl)(4-methoxybenzyl)carbamate (17)**

Prepared according to the general procedure D using *N*-Boc-*N*-(4-methoxybenzyl)but-3-en-1-amine (58.3 mg, 0.2 mmol) and B_2pin_2 (0.4 mmol, 101.6 mg) with 5% $Ni(COD)_2$ (2.8 mg), 5% Cy-XantPhos (6.0 mg) and LiOMe (0.2 mmol, 7.6 mg). The crude reaction mixture was purified on silica gel (20% EtOAc in petroleum ether) to afford the product as a white solid (39.3 mg, 36% yield). **^{1}H NMR** (400 MHz, $CDCl_3$) δ 7.14 (s, 2H), 6.83 (d, *J* = 8.3 Hz, 2H), 4.35 (s, 2H), 3.79 (s, 3H), 3.10 (d, *J* = 36.2 Hz, 2H), 1.48 (s, 13H), 1.21 (d, *J* = 4.1 Hz, 24H), 0.70 (s, 1H). **^{13}C NMR** (101 MHz, $CDCl_3$) δ 158.67, 130.72, 129.08, 128.51, 113.78, 82.97, 79.27, 55.23, 48.83, 46.03, 30.68, 28.49, 24.87, 24.52, 23.05, 10.46. **IR**(neat): 2977 (s), 2930 (m), 1693 (s), 1611 (w), 1513 (s), 1464 (m), 1413 (m), 1366 (s), 1314 (s), 1248 (s), 1142 (s), 1036 (m), 969 (m), 851 (s) cm^{-1}. **HRMS** Calcd for $C_{29}H_{50}B_2NO_7$ $[M+H]^+$ 546.3768; Found: 546.3774.

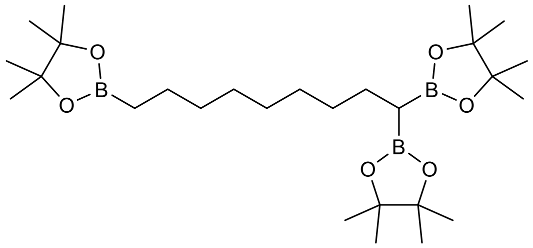

2,2',2''-(nonane-1,1,9-triyl)tris(4,4,5,5-tetramethyl-1,3,2-dioxaborolane) (18)

Prepared according to the general procedure D using 4,4,5,5-tetramethyl-2-(non-8-en-1-yl)-1,3,2-dioxaborolane (50.5 mg, 0.2 mmol) and B_2pin_2 (0.4 mmol, 101.6 mg) with 5% $Ni(COD)_2$ (2.8 mg), 5% Cy-XantPhos (6.0 mg) and LiOMe (0.2 mmol, 7.6 mg). The crude reaction mixture was purified on silica gel (7-10% EtOAc in petroleum ether, stained by $KMnO_4$ in water) to afford the product as a white solid (25.1 mg, 25% yield). **^{1}H NMR** (400 MHz, $CDCl_3$) δ 1.52 (m, 2H), 1.37 (m, 4H), 1.29 – 1.17 (m, 44H), 0.77 – 0.68 (m, 3H). **^{13}C NMR** (101 MHz, $CDCl_3$) δ 82.86, 82.81, 32.63, 32.48, 29.67, 29.52, 29.43, 25.70, 24.86, 24.82, 24.52, 24.03, 11.10 (Signal for one carbon could not be located). **IR**(neat): 2978 (s), 2926 (s), 2855 (m), 1469 (w), 1371 (s), 1317 (s), 1270 (m), 1215 (w), 1145 (s), 970 (m), 849 (m), 669 (w) cm^{-1}. **HRMS** Calcd for $C_{27}H_{54}B_3O_6$ $[M+H]^+$ 507.4194; Found: 507.4188.

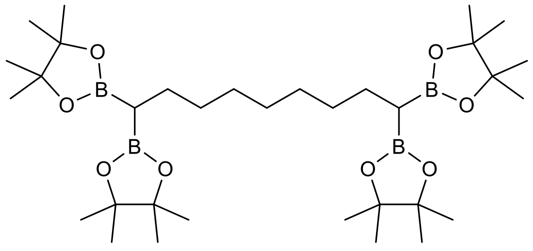

1,1,9,9-tetrakis(4,4,5,5-tetramethyl-1,3,2-dioxaborolan-2-yl)nonane (19)

Prepared according to the general procedure D using 2,2'-(non-8-ene-1,1-diyl)bis(4,4,5,5-tetramethyl-1,3,2-dioxaborolane) (75.6 mg, 0.2 mmol) and B_2pin_2 (0.4 mmol, 101.6 mg) with 5% $Ni(COD)_2$ (2.8 mg), 5% Cy-XantPhos (6.0 mg) and LiOMe (0.2 mmol, 7.6 mg). The crude reaction mixture was purified on silica gel (9-12% EtOAc in petroleum ether, stained by $KMnO_4$ in water) to afford the product as a white solid (57.1 mg, 45% yield). **^{1}H NMR** (400 MHz, $CDCl_3$) δ 1.52 (d, J = 5.7 Hz, 4H), 1.23 (s, 58H), 0.69 (t, J = 7.5 Hz, 2H). **^{13}C NMR** (101 MHz, $CDCl_3$) δ 82.83, 32.66, 29.69, 29.54, 25.70, 24.85, 24.52, 10.77. **IR**(neat): 2978 (s), 2927 (s), 2855 (m), 1467 (m), 1313 (s), 1269 (m), 1216 (m), 1142 (s), 1005 (w), 970 (s), 850 (s), 670 (w), 579 (w) cm^{-1}. **HRMS** Calcd for $C_{33}H_{65}B_4O_8$ $[M+H]^+$ 633.5046; Found: 633.5043.

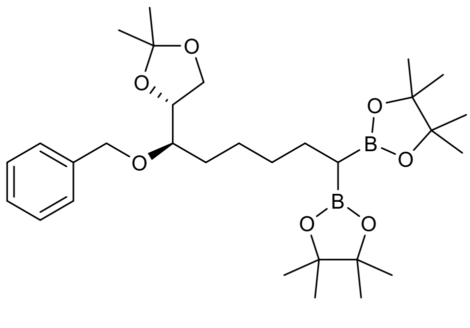

2,2'-((*R*)-6-(benzyloxy)-6-((*R*)-2,2-dimethyl-1,3-dioxolan-4-yl)hexane-1,1-diyl)bis(4,4,5,5-tetramet

hyl-1,3,2-dioxaborolane) (20)

Prepared according to the general procedure D using (*R*)-4-((*R*)-1-(benzyloxy)hex-5-en-1-yl)-2,2-dimethyl-1,3-dioxolane (58.1 mg, 0.2 mmol) and B_2pin_2 (0.4 mmol, 101.6 mg) with 5% $Ni(COD)_2$ (2.8 mg), 5% Cy-XantPhos (6.0 mg) and LiOMe (0.2 mmol, 7.6 mg). The crude reaction mixture was purified on silica gel (9-12% EtOAc in petroleum ether) to afford the product as a thick oil (85.2 mg, 78% yield). **^{1}H NMR** (400 MHz, $CDCl_3$) δ 7.34 – 7.25 (m, 5H), 4.61 (m, 2H), 4.09 (m, 1H), 4.01 (t, *J* = 7.1 Hz, 1H), 3.90 (t, *J* = 7.4 Hz, 1H), 3.53 (m, 1H), 1.56 – 1.45 (m, 4H), 1.42 (s, 3H), 1.35 (s, 3H), 1.31 – 1.26 (m, 4H), 1.22 (d, *J* = 3.3 Hz, 24H), 0.71 (t, *J* = 7.9 Hz, 1H). **^{13}C NMR** (101 MHz, $CDCl_3$) δ 138.69, 128.32, 127.81, 127.53, 108.88, 82.92, 79.00, 78.13, 73.03, 66.10, 32.72, 31.44, 26.57, 25.61, 25.40, 25.26, 24.86, 24.53, 10.79. **IR**(neat): 2978 (s), 2930 (s), 2858 (m), 1456 (m), 1371 (s), 1317 (s), 1267 (m), 1215 (w), 1141 (s), 1028 (w), 970 (m), 850 (m), 735 (w), 697 (w) cm^{-1}. **HRMS** Calcd for $C_{30}H_{51}B_2O_7$ $[M+H]^+$ 545.3815; Found: 545.3815. $[\alpha]_D^{20}$ = +5.05 (*c* = 0.9743, $CHCl_3$, *l* = 100 mm).

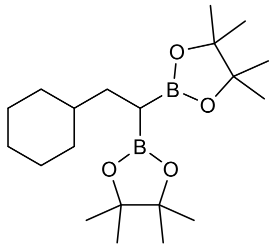

2,2'-(2-cyclohexylethane-1,1-diyl)bis(4,4,5,5-tetramethyl-1,3,2-dioxaborolane) (21)

Prepared according to the general procedure D using vinylcyclohexane (27.4 μL, 0.2 mmol) and B_2pin_2 (0.4 mmol, 101.6 mg) with 5% $Ni(COD)_2$ (2.8 mg), 5% Cy-XantPhos (6.0 mg) and LiOMe (0.2 mmol, 7.6 mg). The crude reaction mixture was purified on silica gel (7-10% EtOAc in petroleum ether, stained by $KMnO_4$ in water) to afford the product as a thick oil (45.0 mg, 62% yield). **^{1}H NMR** (400 MHz, CDCl3) δ 1.67 (m, 6H), 1.46 (t, *J* = 7.4 Hz, 3H), 1.23 (s, 24H), 1.11 (d, *J* = 31.4 Hz, 2H), 0.80 (m, 3H). **^{13}C NMR** (101 MHz, $CDCl_3$) δ 82.83, 39.79, 33.09, 32.90, 26.72, 26.45, 24.76, 24.53, 7.75. **IR**(neat): 2978 (s), 2923 (s), 2851 (s), 1449 (m), 1370 (s), 1313 (s), 1267 (m), 1216 (w), 1142 (s), 970 (s), 851 (s) cm^{-1}. **HRMS** Calcd for $C_{20}H_{39}B_2O_4$ $[M+H]^+$ 365.3029; Found: 365.3037.

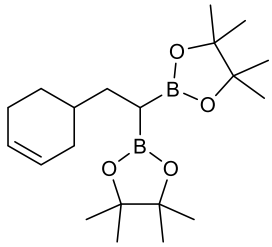

2,2'-(2-(cyclohex-3-en-1-yl)ethane-1,1-diyl)bis(4,4,5,5-tetramethyl-1,3,2-dioxaborolane) (22)

Prepared according to the general procedure D using 4-vinyl-1-cyclohexene (26.0 μL, 0.2 mmol) and B_2pin_2 (0.4 mmol, 101.6 mg) with 5% $Ni(COD)_2$ (2.8 mg), 5% Cy-XantPhos (6.0 mg) and LiOMe (0.2 mmol, 7.6 mg). The crude reaction mixture was purified on silica gel (7-10% EtOAc in petroleum ether, stained by $KMnO_4$ in water) to afford the product as a thick oil (31.8 mg, 44% yield). **^{1}H NMR** (400 MHz, $CDCl_3$) δ 5.66 – 5.61 (m, 2H), 2.05 (m, 2H), 1.76 (d, *J* = 14.1 Hz, 2H), 1.65 – 1.40 (m, 4H), 1.23 (s, 24H), 1.30 – 1.04 (m, 1H), 0.86 (t, *J* = 7.9 Hz, 1H). **^{13}C NMR** (101 MHz, $CDCl_3$) δ 126.93, 126.81, 82.94, 35.65, 32.32, 31.85, 28.6, 25.42, 24.80 (d, *J* = 2.1 Hz), 24.58 (d, *J* = 3.3 Hz), 7.79. **IR**(neat): 2978 (s), 2925 (s), 1449 (m), 1370 (s), 1315 (s), 1262 (m), 1240 (w), 1215 (m), 1141 (s), 970 (s), 851

(s), 657 (m) cm^{-1}. **HRMS** Calcd for $C_{20}H_{37}B_2O_4$ [M+H]$^+$ 363.2872; Found: 363.2876.

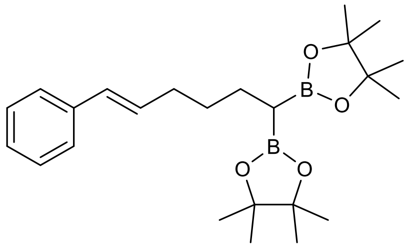

(*E*)-2,2'-(6-phenylhex-5-ene-1,1-diyl)bis(4,4,5,5-tetramethyl-1,3,2-dioxaborolane) (23)

Prepared according to the general procedure D using (*E*)-hexa-1,5-dien-1-ylbenzene (31.6 mg, 0.2 mmol) and B_2pin_2 (0.4 mmol, 101.6 mg) with 5% Ni(COD)$_2$ (2.8 mg), 5% Cy-XantPhos (6.0 mg) and LiOMe (0.2 mmol, 7.6 mg). The crude reaction mixture was purified on silica gel (7-10% EtOAc in petroleum ether) to afford the product as a white solid (40.2 mg, 49% yield). **^{1}H NMR** (400 MHz, $CDCl_3$) δ 7.25 (m, 5H), 6.36 (d, *J* = 15.9 Hz, 1H), 6.26 – 6.19 (m, 1H), 2.19 (m, 2H), 1.63 – 1.59 (m, 2H), 1.46 (m, 2H), 1.23 (d, *J* = 2.6 Hz, 24H), 0.78 – 0.74 (m, 1H). **^{13}C NMR** (101 MHz, $CDCl_3$) δ 138.08, 131.34, 129.45, 128.40, 126.62, 125.90, 82.96, 33.08, 32.24, 25.35, 24.86, 24.53, 10.55. **IR**(neat): 2978 (s), 2930 (s), 2858 (w), 1721 (w), 1451 (m), 1371 (s), 1318 (s), 1268 (s), 1215 (w), 1141 (s), 969 (m), 850 (m), 703 (m) cm^{-1}. **HRMS** Calcd for $C_{24}H_{38}B_2O_4Na$ [M+Na]$^+$ 435.2848; Found: 435.2852.

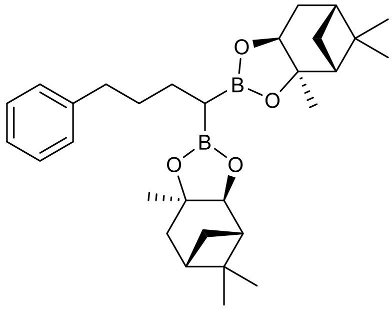

(3*aR*,4*R*,6*R*,7*aS*)-3a,5,5-trimethyl-2-(4-phenyl-1-((3*aS*,4*S*,6*S*,7*aR*)-5,5,7a-trimethylhexahydro-4,6-methanobenzo[d][1,3,2]dioxaborol-2-yl)butyl)hexahydro-4,6-methanobenzo[d][1,3,2]dioxaborole (24)

Prepared according to the general procedure D using 4-phenyl-1-butene (30.0 μL, 0.2 mmol) and B_2pai_2 (0.4 mmol, 143.2 mg) with 10% Ni(COD)$_2$ (5.5 mg), 10% Cy-XantPhos (12.1 mg) and LiOMe (0.2 mmol, 7.6 mg) in 1.1 mL PhMe/THF (v/v = 10:1). The crude reaction mixture was purified on silica gel (7-10% EtOAc in petroleum ether) to afford the product as a white solid (55.0 mg, 56% yield). **^{1}H NMR** (400 MHz, $CDCl_3$) δ 7.27 – 7.12 (m, 5H), 4.25 (d, *J* = 8.6 Hz, 2H), 2.63 (m, 2H), 2.31 (m, 2H), 2.20 – 2.14 (m, 2H), 2.05 (t, *J* = 4.7 Hz, 2H), 1.88 – 1.80 (m, 4H), 1.73 – 1.60 (m, 4H), 1.36 (s, 6H), 1.27 (s, 6H), 1.19 (m, 2H), 0.82 (s, 7H). **^{13}C NMR** (101 MHz, $CDCl_3$) δ 142.93, 128.34, 128.14, 125.40, 85.37, 77.65, 77.60, 51.23, 39.48, 39.47, 38.09, 38.05, 36.03, 35.62, 35.58, 34.32, 28.73, 28.65, 27.09, 26.51, 26.44, 25.84, 24.00, 9.02. **IR**(neat): 2985 (m), 2928 (s), 2869 (m), 1452 (m), 1375 (s), 1340 (m), 1308 (w), 1278 (m), 1235 (m), 1122 (m), 1078 (m), 1030 (s), 751 (w), 697 (w) cm^{-1}. $[\alpha]_D^{20}$ = +17.56 (*c* = 1.1267, $CHCl_3$, *l* = 100 mm). **HRMS** Calcd for $C_{30}H_{44}B_2O_4Na$ [M+Na]$^+$ 513.3318; Found: 513.3318.

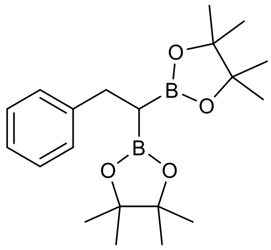

2,2'-(2-phenylethane-1,1-diyl)bis(4,4,5,5-tetramethyl-1,3,2-dioxaborolane) (26)

Prepared according to the general procedure E using styrene (23.0 μL, 0.2 mmol) and B_2pin_2 (0.4 mmol, 101.6 mg) with 5% $Ni(COD)_2$ (2.8 mg), 10% PCy_3 (5.6 mg) at 140°C for 12h. The crude reaction mixture was purified on silica gel (7-10% EtOAc in petroleum ether) to afford the product as a thick oil (32.5 mg, 45% yield). **^{1}H NMR** (400 MHz, $CDCl_3$) δ 7.23 – 7.09 (m, 5H), 2.88 (d, *J* = 8.4 Hz, 2H), 1.17 (d, *J* = 4.2 Hz, 25H). **^{13}C NMR** (101 MHz, $CDCl_3$) δ 144.43, 128.32, 127.94, 125.33, 83.09, 31.28, 24.77, 24.50, 12.54. **IR**(neat): 3026 (w), 2978 (s), 2930 (m), 2858 (w), 1454 (m), 1371 (s), 1321 (s), 1269 (m), 1241 (w), 1140 (s), 972 (s), 852 (s), 699 (m) cm^{-1}. **HRMS** Calcd for $C_{20}H_{33}B_2O_4$ $[M+H]^+$ 359.2559; Found: 359.2564.

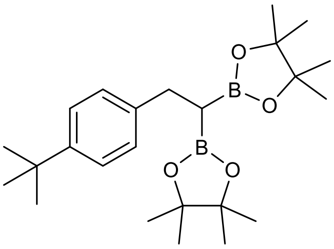

2,2'-(2-(4-(*tert*-butyl)phenyl)ethane-1,1-diyl)bis(4,4,5,5-tetramethyl-1,3,2-dioxaborolane) (27)

Prepared according to the general procedure E using 1-(*tert*-butyl)-4-vinylbenzene (36.6.0 μL, 0.2 mmol) and B_2pin_2 (0.4 mmol, 101.6 mg) with 5% $Ni(COD)_2$ (2.8 mg), 10% PCy_3 (5.6 mg) for 12h. The crude reaction mixture was purified on silica gel (7-10% EtOAc in petroleum ether) to afford the product as a thick oil (43.1 mg, 52% yield). **^{1}H NMR** (400 MHz, $CDCl_3$) δ 7.24 (d, *J* = 7.9 Hz, 2H), 7.16 (d, *J* = 8.0 Hz, 2H), 2.85 (d, *J* = 8.4 Hz, 2H), 1.28 (s, 9H), 1.16 (s, 25H). **^{13}C NMR** (101 MHz, $CDCl_3$) δ 148.05, 141.37, 128.02, 124.81, 83.06, 34.27, 31.43, 30.74, 24.74, 24.53, 12.55. **IR**(neat): 2975 (s), 2926 (m), 2866 (w), 1509 (w), 1464 (m), 1323 (s), 1267 (m), 1237 (m), 1213 (m), 1138 (s), 1109 (w), 1076 (w), 970 (s), 855 (m), 839 (s), 669 (w), 566 (m) cm^{-1}. **HRMS** Calcd for $C_{24}H_{41}B_2O_4$ $[M+H]^+$ 415.3185; Found: 415.3186.

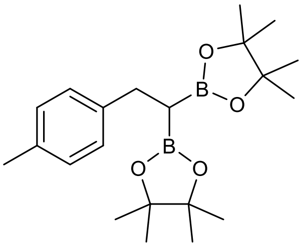

2,2'-(2-(*p*-tolyl)ethane-1,1-diyl)bis(4,4,5,5-tetramethyl-1,3,2-dioxaborolane) (28)

Prepared according to the general procedure E using 1-methyl-4-vinylbenzene (26.4 μL, 0.2 mmol) and B_2pin_2 (0.4 mmol, 101.6 mg) with 5% $Ni(COD)_2$ (2.8 mg), 10% PCy_3 (5.6 mg). The crude reaction mixture was purified on silica gel (7-10% EtOAc in petroleum ether) to afford the product as a thick oil (41.7 mg, 56% yield). **^{1}H NMR** (400 MHz, $CDCl_3$) δ 7.12 (d, *J* = 7.6 Hz, 2H), 7.02 (d, *J* = 7.6 Hz, 2H),

2.84 (d, J = 8.2 Hz, 2H), 2.28 (s, 3H), 1.18 (s, 25H). **^{13}C NMR** (101 MHz, $CDCl_3$) δ 141.39, 134.61, 128.63, 128.17, 83.08, 30.85, 24.80, 24.53, 20.99, 12.66. **IR**(neat): 2978 (s), 2928 (m), 2858 (w), 1515 (m), 1360 (s), 1319 (s), 1269 (m), 1241 (w), 1215 (w), 1140 (s), 972 (s), 853 (s), 841 (m) cm^{-1}. **HRMS** Calcd for $C_{21}H_{35}B_2O_4$ [M+H]$^+$ 373.2716; Found: 373.2720.

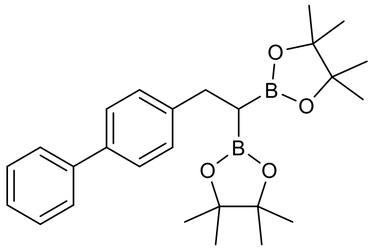

2,2'-(2-([1,1'-biphenyl]-4-yl)ethane-1,1-diyl)bis(4,4,5,5-tetramethyl-1,3,2-dioxaborolane) (29)
Prepared according to the general procedure E using 4-vinyl-1,1'-biphenyl (36 mg, 0.2 mmol) and B_2pin_2 (0.4 mmol, 101.6 mg) with 5% $Ni(COD)_2$ (2.8 mg), 10% PCy_3 (5.6 mg) for 12h. The crude reaction mixture was purified on silica gel (7-10% EtOAc in petroleum ether) to afford the product as a white solid (68.6mg, 71% yield). **^{1}H NMR** (400 MHz, $CDCl_3$) δ 7.56 (d, J = 7.7 Hz, 2H), 7.46 (d, J = 7.2 Hz, 2H), 7.40 (t, J = 7.5 Hz, 2H), 7.30 (t, J = 7.8 Hz, 3H), 2.93 (d, J = 8.3 Hz, 2H), 1.18 (s, 24H), 1.14 (s, 1H). **^{13}C NMR** (101 MHz, $CDCl_3$) δ 143.67, 141.34, 138.24, 128.76, 128.64, 126.93, 126.81, 126.70, 83.14, 30.94, 24.78, 24.51, 12.58. **IR**(neat): 2978 (s), 2929 (m), 1487 (m), 1371 (s), 1320 (s), 1268 (m), 1139 (s), 971 (m), 844 (m), 763 (m), 697 (m) cm^{-1}. **HRMS** Calcd for $C_{26}H_{36}B_2O_4Na$ [M+Na]$^+$ 457.2692; Found: 457.2695.

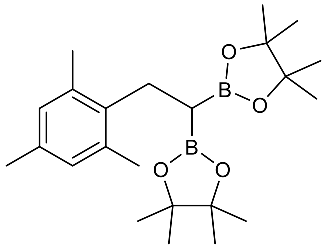

2,2'-(2-mesitylethane-1,1-diyl)bis(4,4,5,5-tetramethyl-1,3,2-dioxaborolane) (30)
Prepared according to the general procedure E using 1,3,5-trimethyl-2-vinylbenzene (32.2 μL, 0.2 mmol) and B_2pin_2 (0.4 mmol, 101.6 mg) with 5% $Ni(COD)_2$ (2.8 mg), 10% PCy_3 (5.6 mg) for 12h. The crude reaction mixture was purified on silica gel (7-10% EtOAc in petroleum ether) to afford the product as a thick oil (52.7 mg, 66% yield). **^{1}H NMR** (400 MHz, $CDCl_3$) δ 6.75 (s, 2H), 2.89 (d, J = 6.9 Hz, 2H), 2.32 (s, 6H), 2.20 (s, 3H), 1.19 (d, J = 5.3 Hz, 24H), 1.02 – 0.98 (m, 1H). **^{13}C NMR** (101 MHz, $CDCl_3$) δ 138.57, 136.10, 134.34, 128.71, 83.08, 24.76, 24.63, 23.99, 20.75, 20.25, 10.74. **IR**(neat): 2978 (s), 2928 (m), 1481 (m), 1467 (m), 1355 (s), 1314 (s), 1268 (m), 1140 (s), 971 (m), 850 (s) cm^{-1}. **HRMS** Calcd for $C_{23}H_{38}B_2O_4Na$ [M+Na]$^+$ 423.2848; Found: 423.2850.

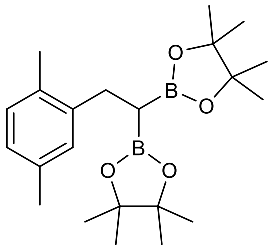

2,2'-(2-(2,5-dimethylphenyl)ethane-1,1-diyl)bis(4,4,5,5-tetramethyl-1,3,2-dioxaborolane) (31)
Prepared according to the general procedure E using 1,4-dimethyl-2-vinylbenzene (29.3 μL, 0.2 mmol) and B_2pin_2 (0.4 mmol, 101.6 mg) with 5% $Ni(COD)_2$ (2.8 mg), 10% PCy_3 (5.6 mg) for 12h. The crude reaction mixture was purified on silica gel (7-10% EtOAc in petroleum ether) to afford the product as a thick oil (31.7 mg, 41% yield). **^{1}H NMR** (400 MHz, $CDCl_3$) δ 7.06 (s, 1H), 6.96 (d, *J* = 7.5 Hz, 1H), 6.84 (d, *J* = 7.6 Hz, 1H), 2.82 (d, *J* = 8.0 Hz, 2H), 2.80 (t, *J* = 11.2 Hz, 2H), 2.25 (d, *J* = 4.2 Hz, 6H), 1.19 (s, 24H), 1.12 (t, *J* = 7.4 Hz, 2H). **^{13}C NMR** (101 MHz, $CDCl_3$) δ 142.26, 134.59, 132.74, 129.75, 129.38, 126.01, 83.06, 28.43, 24.78, 24.54, 20.95, 18.88, 10.96. **IR**(neat): 2978 (s), 2929 (m), 1458 (m), 1359 (s), 1317 (s), 1269 (m), 1215 (w), 1141 (s), 971 (m), 851 (s) cm^{-1}. **HRMS** Calcd for $C_{22}H_{36}B_2O_4Na$ $[M+Na]^+$ 409.2692; Found:409.2694.

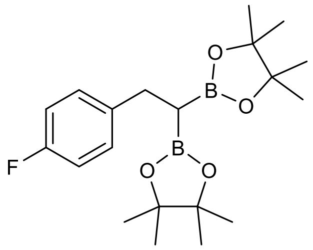

2,2'-(2-(4-fluorophenyl)ethane-1,1-diyl)bis(4,4,5,5-tetramethyl-1,3,2-dioxaborolane) (32)
Prepared according to the general procedure E using 1-fluoro-4-vinylbenzene (23.9 μL, 0.2 mmol) and B_2pin_2 (0.4 mmol, 101.6 mg) with 5% $Ni(COD)_2$ (2.8 mg), 10% PCy_3 (5.6 mg) for 12h. The crude reaction mixture was purified on silica gel (7-10% EtOAc in petroleum ether) to afford the product as a thick oil (25.6 mg, 34% yield). **^{1}H NMR** (400 MHz, $CDCl_3$) δ 7.22 – 7.16 (m, 2H), 6.90 (m, 2H), 2.84 (d, *J* = 7.5 Hz, 2H), 1.17 (d, *J* = 3.7 Hz, 24H), 1.12 (d, *J* = 1.5 Hz, 1H). **^{13}C NMR** (101 MHz, $CDCl_3$) δ 162.24, 159.83, 140.08, 129.68, 129.60, 114.67, 114.46, 83.15, 30.51, 24.78, 24.49, 12.54. **^{19}F NMR** (376 MHz, $CDCl_3$) δ -118.62, -118.63, -118.64. **IR**(neat): 2979 (s), 2931 (m), 1597 (w), 1510 (s), 1456 (m), 1371 (s), 1319 (s), 1269 (w), 1219 (m), 1140 (s), 972 (m), 854 (m), 843 (m), 679 (w) cm^{-1}. **HRMS** Calcd for $C_{20}H_{31}B_2FO_4Na$ $[M+Na]^+$ 399.2285; Found: 399.2288.

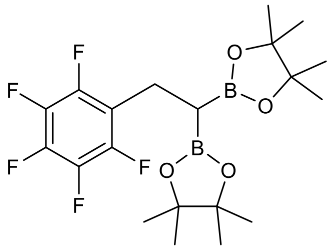

2,2'-(2-(perfluorophenyl)ethane-1,1-diyl)bis(4,4,5,5-tetramethyl-1,3,2-dioxaborolane) (33)
Prepared according to the general procedure E using 1,2,3,4,5-pentafluoro-6-vinylbenzene (27.6 μL, 0.2 mmol) and B_2pin_2 (0.4 mmol, 101.6 mg) with 5% $Ni(COD)_2$ (2.8 mg), 10% PCy_3 (5.6 mg) for 12h. The crude reaction mixture was purified on silica gel (7-10% EtOAc in petroleum ether) to afford the product as a thick oil (35.9 mg, 40% yield). **^{1}H NMR** (400 MHz, $CDCl_3$) δ 2.92 (d, *J* = 8.1 Hz, 2H), 1.19 (d, *J* = 11.4 Hz, 25H). **^{13}C NMR** (101 MHz, $CDCl_3$) δ 83.45, 24.87, 24.37, 18.33, 10.59. **^{19}F NMR** (376 MHz, $CDCl_3$) δ -140.73, -140.77, -140.79, -140.83, -142.40, -142.42, -142.46, -142.48, -142.97, -143.01, -143.03, -143.07, -159.12, -159.18, -159.23, -163.73, -163.75, -163.79, -163.81, -163.85, -163.87. **IR**(neat): 2980 (s), 2930 (w), 1520 (s), 1503 (s), 1372 (s), 1321 (s), 1268 (m), 1245 (w), 1167 (w), 1141 (s), 992 (m), 972 (m), 850 (m) cm^{-1}. **HRMS** Calcd for $C_{20}H_{28}B_2F_5O_4$ $[M+H]^+$ 449.2088;

Found: 449.2089.

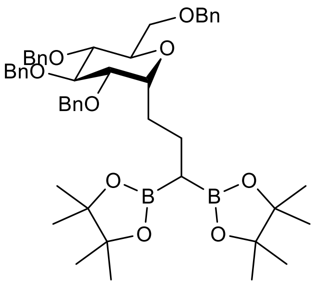

2,2'-(3-((2*R*,3*S*,4*R*,5*R*,6*R*)-3,4,5-tris(benzyloxy)-6-((benzyloxy)methyl)tetrahydro-2*H*-pyran-2-yl)propane-1,1-diyl)bis(4,4,5,5-tetramethyl-1,3,2-dioxaborolane) (36)

Prepared according to the general procedure D using **35** (113.0 mg, 0.2 mmol) and B_2pin_2 (0.4 mmol, 101.6 mg) with 10% Ni(COD)$_2$ (5.5 mg), 10% Cy-XantPhos (12.0 mg) and LiOMe (0.2 mmol, 7.6 mg). The crude reaction mixture was purified on silica gel (13-16% EtOAc in petroleum ether) to afford the product as a thick oil (103.2 mg, 63% yield). **^{1}H NMR** (400 MHz, $CDCl_3$) δ 7.35 – 7.21 (m, 20H), 4.91 (d, *J* = 10.9 Hz, 1H), 4.85 – 4.72 (m, 2H), 4.68 – 4.60 (m, 3H), 4.46 (m, 2H), 4.07 (m, 1H), 3.82 – 3.60 (m, 6H), 1.85 – 1.46 (m, 4H), 1.21 (d, *J* = 6.4 Hz, 1H), 0.78 (t, *J* = 7.4 Hz, 1H). **^{13}C NMR** (101 MHz, $CDCl_3$) δ 138.87, 138.74, 138.44, 138.25, 128.29, 128.24, 128.18, 127.91, 127.90, 127.62, 127.52, 127.47, 127.35, 82.98, 82.95, 82.68, 80.20, 78.24, 75.38, 74.63, 73.97, 73.40, 72.61, 70.66, 69.12, 27.27, 24.95, 24.87, 24.51, 24.41, 21.45, 10.50. **IR**(neat): 3031 (w), 2977 (s), 2928 (s), 2967 (m), 1495 (w), 1454 (m), 1361 (s), 1314 (s), 1268 (w), 1214 (w), 1140 (s), 1092 (s), 1028 (w), 970 (w), 850 (m), 736 (s), 697 (s) cm^{-1}. **HRMS** Calcd for $C_{49}H_{65}B_2O_9$ $[M+H]^+$ 819.4809; Found: 819.4817. $[\alpha]_D^{20}$ = +27.08 (*c* = 0.7933, $CHCl_3$, *l* = 100 mm).

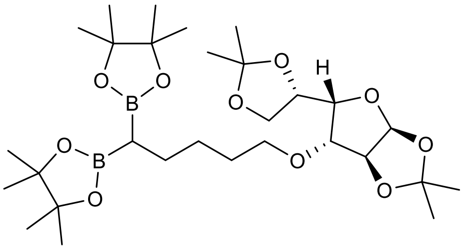

2,2'-(5-(((3*aS*,5*S*,6*R*,6*aS*)-5-((*S*)-2,2-dimethyl-1,3-dioxolan-4-yl)-2,2-dimethyltetrahydrofuro[2,3-d][1,3]dioxol-6-yl)oxy)pentane-1,1-diyl)bis(4,4,5,5-tetramethyl-1,3,2-dioxaborolane) (39)

Prepared according to the general procedure D using **38** (65.7 mg, 0.2 mmol) and B_2pin_2 (0.4 mmol, 101.6 mg) with 5% Ni(COD)$_2$ (2.8 mg), 5% Cy-XantPhos (6.0 mg) and LiOMe (0.2 mmol, 7.6 mg) for 4h. The crude reaction mixture was purified on silica gel (13-16% EtOAc in petroleum ether, stained by $KMnO_4$ in water) to afford the product as a thick oil (80.4 mg, 69% yield). **^{1}H NMR** (400 MHz, $CDCl_3$) δ 5.86 (d, *J* = 3.7 Hz, 1H), 4.52 (d, *J* = 3.6 Hz, 1H), 4.29 (dd, *J* = 13.1, 6.2 Hz, 1H), 4.12 (dd, *J* = 7.3, 3.0 Hz, 1H), 4.06 (dd, *J* = 8.4, 6.3 Hz, 1H), 3.97 (dd, *J* = 8.5, 6.0 Hz, 1H), 3.83 (d, *J* = 3.0 Hz, 1H), 3.53 (m, 2H), 1.60 – 1.51 (m, 3H), 1.49 (s, 3H), 1.42 (s, 3H), 1.33 (d, *J* = 13.5 Hz, 7H), 1.26 (s, 2H), 1.23 (d, *J* = 3.5 Hz, 24H), 0.71 (t, *J* = 7.8 Hz, 1H). **^{13}C NMR** (101 MHz, $CDCl_3$) δ 111.65, 108.81, 105.26, 82.94, 82.58, 82.04, 81.21, 72.57, 70.74, 67.11 , 29.78, 28.87, 26.87, 26.76, 26.26, 25.44, 25.41, 24.85, 24.52, 10.69. **IR**(neat): 2979 (s), 2934 (s), 1458 (m), 1370 (s), 1317 (s), 1265 (m), 1216 (m), 1141 (s), 1083 (s), 1020 (m), 970 (m), 850 (s), 733 (m), 673 (w) cm^{-1}. **HRMS** Calcd for $C_{29}H_{53}B_2O_{10}$ $[M+H]^+$ 583.3819; Found: 583.3822. $[\alpha]_D^{20}$ = -12.37 (*c* = 1.085, $CHCl_3$, *l* = 100 mm).

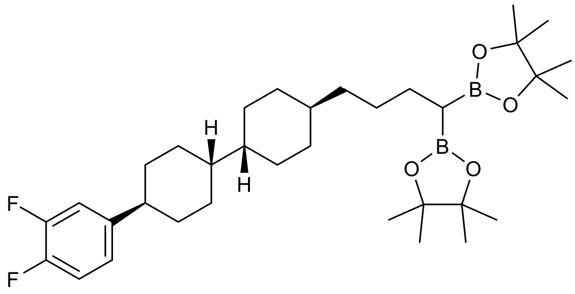

4-{*trans*-4-[*trans*-4-(3,4-difluorophenyl)cyclohexyl]cyclohexyl}butylbis(4,4,5,5-tetramethyl-1,3,2-dioxaborolane) (41)

Prepared according to the general procedure D using **40** (66.5 mg, 0.2 mmol) and B_2pin_2 (0.4 mmol, 101.6 mg) with 5% $Ni(COD)_2$ (2.8 mg), 5% Cy-XantPhos (6.0 mg) and LiOMe (0.2 mmol, 7.6 mg). The crude reaction mixture was purified on silica gel (7-10% EtOAc in petroleum ether) to afford the product as a white solid (55.1 mg, 47% yield). **^{1}H NMR** (400 MHz, $CDCl_3$) δ 7.07 – 6.83 (m, 3H), 2.40 (t, *J* = 12.0 Hz, 1H), 1.89 – 1.82 (m, 10H), 1.52 (m, 2H), 1.23 (d, *J* = 3.8 Hz, 24H), 1.13 (m, 7H), 0.98 (m, 3H), 0.87 – 0.81 (m, 3H), 0.72 (t, *J* = 7.8 Hz, 1H). **^{13}C NMR** (101 MHz, $CDCl_3$) δ 148.96, 147.22, 147.19, 144.96, 122.53, 116.79, 116.63, 115.46, 115.30, 82.86, 43.87, 43.33, 42.80, 37.58, 37.42, 34.59, 33.54, 30.15, 30.06, 29.89, 25.94, 24.86, 24.50, 10.85. **^{19}F NMR** (376 MHz, CDCl3) δ -138.71, -138.76, -142.62, -142.68. **IR**(neat): 2977 (m), 2920 (s), 2849 (m), 1517 (m), 1446 (w), 1369 (m), 1310 (s), 1270 (w), 1214 (w), 1138 (s), 968 (m), 849 (m), 737 (m) cm^{-1}. **HRMS** Calcd for $C_{34}H_{55}B_2F_2O_4$ $[M+H]^+$ 587.4249; Found: 587.4242.

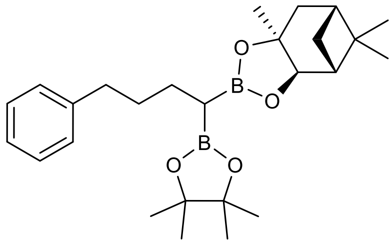

(3*aR*,4*R*,6*R*,7*aS*)-5,5,7a-trimethyl-2-(4-phenyl-1-(4,4,5,5-tetramethyl-1,3,2-dioxaborolan-2-yl)butyl)hexahydro-4,6-methanobenzo[d][1,3,2]dioxaborole (47)

Prepared according to the general procedure D using 4-phenyl-1-butene (30.0 μL, 0.2 mmol), B_2pin_2 (0.2 mmol, 50.8 mg) and B_2pai_2 (71.6 mg, 0.2 mmol) with 10% $Ni(COD)_2$ (5.5 mg), 10% Cy-XantPhos (12.0 mg) and LiOMe (0.2 mmol, 7.6 mg) for 4h. The crude reaction mixture was purified on silica gel (10% EtOAc in petroleum ether) to afford the product as a white solid. **^{1}H NMR** (400 MHz, $CDCl_3$) δ 7.25 (d, *J* = 10.1 Hz, 2H), 7.19 – 7.10 (m, 3H), 4.24 (d, *J* = 8.5 Hz, 1H), 2.60 (s, 2H), 2.37 – 2.25 (m, 1H), 2.18 (m, 1H), 2.09 – 1.96 (m, 1H), 1.93 – 1.77 (m, 2H), 1.64 (s, 4H), 1.34 (d, *J* = 7.5 Hz, 3H), 1.26 (d, *J* = 6.9 Hz, 5H), 1.22 (d, *J* = 2.5 Hz, 12H), 0.83 (s, 3H). **^{13}C NMR** (101 MHz, $CDCl_3$) δ 143.02, 128.35, 128.16, 125.48, 85.39, 82.98, 77.68, 51.27, 39.54, 38.11, 36.07, 35.67, 34.37, 28.72, 27.16, 26.54, 25.76, 24.90, 24.55, 24.07, 9.75. **IR**(neat): 2977 (s), 2929 (s), 2867 (w), 1451 (m), 1370 (s), 1311 (s), 1277 (w), 1235 (m), 1145 (s), 1078 (m), 1030 (s), 967 (w), 851 (m), 743 (m), 695 (m) cm^{-1}. **HRMS** Calcd for $C_{26}H_{40}B_2O_4Na$ $[M+Na]^+$ 461.3005; Found:461.3008.

Synthesis of B_2pin_2-d_{12}

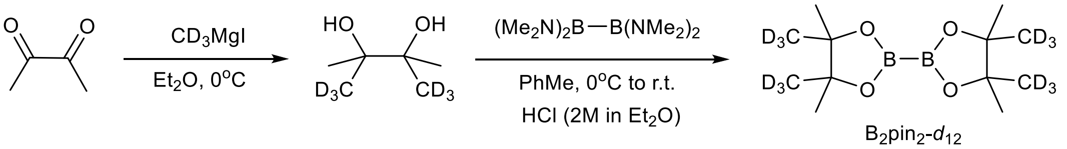

Iodomethane-d_3 (3.0 mL, 48.2 mmol) was added in dropwise to a suspension of Mg powder (2.89 g, 120.5 mmol) in Et_2O (20.0 mL) at 0 ℃ under argon atmosphere. A particle of iodine was added as an initiator to the ether solution and the solution was warmed to r.t. and stirred for 2h. The (methyl-d_3)magnesium iodide ether solution was then cooled to 0 ℃ and a solution of 2-3-butanedione (2.07 g, 24.1 mmol) in Et_2O (10.0 mL) was added in dropwise over 15 min. The mixture was warmed to r.t. and stirred overnight. The mixture was diluted with Et_2O (50.0 mL) and quenched by addition of saturated NH_4Cl solution (50.0 mL) (*Reaction mixture turned into a thick slurry during quenching and needed to be agitated with a glass rod to aid stirring*). The organic layers were separated and the aqueous phase was extracted with Et_2O (50 mL × 2) and the combined organic phase was washed with brine. The organic phase was then dried over Na_2SO_4 and the solvent was carefully removed. The crude oil was purified by silica gel (eluted with EtOAc) to give 2,3-dimethylbutane-1,1,1,4,4,4-d_6-2,3-diol as a colorless oil (1.28 g, 43%). Tetrakis(dimethylamido)diborane (594 mg, 3.0 mmol) and the obtained deuterated alcohol (745 mg, 6.0 mmol) were added to a round-bottom flask equipped with 15.0 mL toluene and a magnetic stir bar. The toluene solution was cooled to 0 ℃ and a solution of HCl in Et_2O (2.0 M, 7.0 mL) was added in dropwise over 20 min. White solids appeared with addition of the acidic ether solution. The solution was then warmed to r.t. for 4h when the addition finished. Followed by filtration and cautious concentration, the crude material was purified by silica gel (10-13% EtOAc in petroleum ether, stained by $KMnO_4$ in water) to afford the desired B_2pin_2-d_{12} as a white solid (495 mg, 62%).

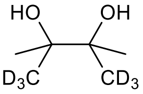

2,3-dimethylbutane-1,1,1,4,4,4-d_6-2,3-diol

^{1}H NMR (400 MHz, $CDCl_3$) δ 2.79 (s, 2H), 1.23 (d, J = 10.3 Hz,6H). **^{13}C NMR** (101 MHz, CDCl3) δ 75.00 (s), 24.74 (s). **IR**(neat): 3440 (br), 2976 (s), 2941 (m), 2230 (m), 1457 (m), 1374 (s), 1141 (s), 1119 (s), 1050 (s), 920 (m) cm^{-1}.

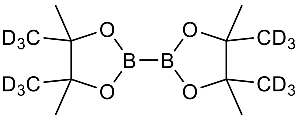

4,4',5,5'-tetramethyl-4,4',5,5'-tetrakis(methyl-d_3)-2,2'-bi(1,3,2-dioxaborolane) (B_2pin_2-d_{12})

^{1}H NMR (400 MHz, $CDCl_3$) δ 1.26 (s, 12H). **^{13}C** NMR (101 MHz, $CDCl_3$) δ 83.33, 24.92. **IR**(neat): 2978 (s), 2936 (m), 2229 (s), 1460 (m), 1380 (m), 1286 (br), 1169 (br), 1100 (s), 1052 (m), 909 (s), 897 (s), 790 (s), 768 (s), 620 (m), 549 (s) cm^{-1}. **HRMS** Calcd for $C_{12}H_{13}D_{12}B_2O_4$ $[M+H]^+$ 267.2687; Found: 267.2693.

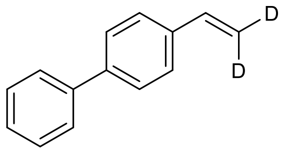

4-(vinyl-2,2-*d*$_2$)-1,1'-biphenyl (29a-*d*$_2$)

This compound was prepared as follows, methyl-*d*$_3$-triphenylphosphonium iodide (98% deuterium) (2.0 g, 5.0 mmol) was dissolved in anhydrous THF (30.0 mL) at -78°C. *n*-Butyllithium (2.5M in hexanes, 1.05 equiv.) was added in dropwise over 30 minutes at -78°C. At this temperature, [1,1'-biphenyl]-4-carbaldehyde (912 mg, 5 mmol) was added dropwise. The reaction was then allowed to warm to room temperature for 1h. Quenched by ice water, the reaction was extracted with EtOAc (30.0 mL × 3). The organic phase was combined, dried over Na_2SO_4, filtrated and concentrated. The deuterated product was purified by silica gel (petroleum ether) and obtained as a white solid (793 mg, 87% yield) containing 93% deuterium isotope according to ^{1}H NMR. **^{1}H NMR** (400 MHz, $CDCl_3$) δ 7.58 (m, 4H), 7.51 – 7.40 (m, 4H), 7.33 (t, *J* = 7.3 Hz, 1H), 6.74 (s, 1H). **^{13}C NMR** (101 MHz, $CDCl_3$) δ 140.71, 140.54, 136.55, 136.19, 128.77, 127.30, 127.21, 126.95, 126.63. **IR**(neat): 3056 (w), 3033 (w), 2996 (w), 1577 (w), 1485 (s), 1400 (m), 936 (m), 839 (s), 766 (s), 732 (s), 690 (s) cm^{-1}. (^{1}H NMR and ^{13}C NMR spectra were in accordance with reference: Ilchenko N. O., Tasch B. O. A., Szabó K. J., *Angew. Chem. Int. Ed.*, **2014**, 53, 12897.)

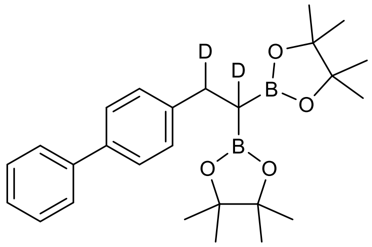

2,2'-(2-([1,1'-biphenyl]-4-yl)ethane-1,1-diyl-1,2-d2)bis(4,4,5,5-tetramethyl-1,3,2-dioxaborolane) (29-*d*$_2$)

Prepared according to the general procedure E using **29a-*d*$_2$** (36.5 mg, 0.2 mmol) and B_2pin_2 (0.4 mmol, 101.6 mg) with 5% Ni(COD)$_2$ (2.8 mg), 10% PCy_3 (5.6 mg) for 1h. The crude reaction mixture was purified on silica gel (7-10% EtOAc in petroleum ether) to afford the deuterated product as a white solid (34.9 mg, 40% yield). (One of the benzylic hydrogen atoms in **29-*d*$_2$** was 92% deuterated according to ^{1}H NMR) **^{1}H NMR** (400 MHz, $CDCl_3$) δ 7.56 (d, *J* = 7.7 Hz, 2H), 7.46 (d, *J* = 7.9 Hz, 2H), 7.40 (t, *J* = 7.5 Hz, 2H), 7.31 (d, *J* = 7.7 Hz, 3H), 2.92 (t, *J* = 7.4 Hz, 1H), 1.18 (s, 24H). **^{13}C NMR** (101 MHz, $CDCl_3$) δ 143.64, 141.35, 138.24, 128.76, 128.64, 126.93, 126.81, 126.71, 83.15, 30.95, 24.79, 24.51, 12.51. **IR**(neat): 2978 (s), 2929 (m), 1487 (m), 1371 (s), 1355 (s), 1268 (m), 1140 (s), 971 (m), 852 (m), 765 (m), 699 (m) cm^{-1}. **HRMS** Calcd for $C_{26}H_{35}D_2B_2O_4$ $[M+H]^+$ 437.2998; Found: 437.2997.

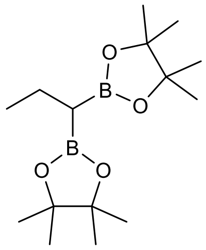

2,2'-(propane-1,1-diyl)bis(4,4,5,5-tetramethyl-1,3,2-dioxaborolane) (43)

Following general procedure F: In a glove box, to a 10 mL Schlenk tube equipped with a stir bar were added B_2pin_2 (2.0 equiv.), 5% $Ni(COD)_2$, 5% Cy-XantPhos and LiOMe (1.0 equiv.). Sealed with a teflon cap, the tube was removed from the glove box. *Then the tube was evacuated under vacuum and propylene was fully charged (three cycles), followed by NEt_3 (0.5 equiv.) and 1.1 mL PhMe/THF (v/v = 10:1).* Capped with the teflon cap, the reaction mixture was allowed to stir at r.t. for seconds, then transferred into a 130°C oil bath. After 12 hours stirring, the reaction completed and was cooled to room temperature. The reaction mixture was then diluted with EtOAc, filtered through a short pad of silica gel with copious washings (EtOAc), concentrated, and purified by column chromatography (7-10% EtOAc in petroleum ether, stained by $KMnO_4$ in water) to afford the desired diboration product **43** as a colorless oil (97 mg, 82%). **^{1}H NMR** (400 MHz, CDCl3) δ 1.64 – 1.51 (m, 2H), 1.23 (d, J = 3.7 Hz, 24H), 0.92 (t, J = 7.3 Hz, 3H), 0.66 (t, J = 7.8 Hz, 1H). **^{13}C NMR** (101 MHz, $CDCl_3$) δ 82.86, 24.88, 24.51, 19.06, 17.02, 12.64. ; **IR**(neat): 2978 (s), 1369 (s), 1308 (m), 1142 (s), 970 (m), 851 (m) cm^{-1}; **HRMS** Calcd for $C_{15}H_{31}B_2O_4$ $[M+H]^+$ 297.2403; Found: 297.2408.

Supplementary Table 5. Crystal data and structure refinement for compound **41**

Empirical formula	$C_{71}H_{115}B_4F_4O_8$
Formula weight	1215.86
Temperature	150(1)
Wavelength	1.54184
Crystal system, space group	triclinic, 2 P -1 -P 1
Unit cell dimensions	a = 11.5338(4) Å α = 87.998(2)<br>b = 14.1735(3) Å β = 84.767(2)<br>c = 22.2327(5) Å γ = 81.745(2)
Volume	3580.95(17) $Å^3$
Z, Calculated density	2, 1.128 Mg/m^3
Absorption coefficient	0.617
F(000)	1322
Crystal size	0.26 × 0.22 × 0.21 mm^3
Theta range for data collection	3.694 to 71.461^o
Limiting indices	-13<=h<=14, -17<=k<=15, -25<=l<=27
Reflections collected / unique	13299/11177 [R(int) = 0.0293]
Completeness to theta = 67.684	98.6%
Absorption correction	multi-scan
Max. and min. transmission	0.86136 and 1.00000
Refinement method	Full-matrix least-squares on F^2
Data / restraints / parameters	13299/55/827
Goodness-of-fit on F^2	1.060
Final R indices [I>2sigma(I)]	R = 0.0757, wR = 0.2051
R indices (all data)	R = 0.0857, wR = 0.2148
Radiation type	Cu/Kα
Largest diff. peak and hole	0.613 and -0.822 e.$Å^{-3}$

Supplementary Table 6. Selective data of atomic coordinates (× 10^4) and equivalent isotropic displacement parameters ($Å^2$ × 10^3) for compound **41**

	x	y	z	U(eq)

O1	4818.1(15)	4408.9(12)	12929.7(9)	38.4(4)
O2	6588.2(15)	4192.9(12)	12379.6(8)	36.2(4)
O3	4552.7(15)	6816.5(13)	13473.6(7)	33.7(4)
O4	6478.4(14)	6196.8(12)	13526.3(7)	32.2(4)
B1	5675(2)	4834.4(19)	12608.4(11)	27(5)
B2	5547(2)	6343.9(18)	13179.1(11)	26.4(5)
B3	623(2)	454(2)	2738.7(11)	27.6(5)
B4	348(2)	1893(2)	3406.9(12)	31.4(6)
H1	6311	6093	1229.3	32
H2A	3817	6221	12426	34
H2B	0.4490	0.7046	12160	34
H3A	5221	6108	11308	38
H3B	4511	5299	11573	38
H4A	3434	7145	11270	40
H4B	2750	6311	11503	40
H5	4004	6366	10352	38
H6A	2332	7493	10408	58
H6B	1549	6728	10663	58
H7A	1270	7009	9645	61
H7B	2627	6785	9467	61
H8	1204	5427	9947	38
C1	5595.3(19)	5942.8(16)	12525.9(10)	26.9(5)
C2	4523(2)	6358.2(16)	12187.8(10)	28.3(5)
C3	4514(2)	5983.2(17)	11551.3(10)	31.6(5)
C4	3441(2)	6460.4(18)	11256.4(11)	33.6(5)
C5	3312(2)	6210.9(17)	10604.3(10)	32.1(5)
C6	2229(3)	6828(2)	10389.8(13)	48(7)
C7	1989(3)	6616(2)	9746.6(14)	50.9(8)
C8	1870(2)	5570.1(17)	9671.0(11)	31.6(5)
C9	2971(2)	4958.1(18)	9874.8(11)	33.9(5)

C10	3210(2)	5166.4(18)	10520.6(11)	33.5(5)
C11	1609(2)	5339.0(17)	9026.6(11)	32.7(5)
F1	-1414(4)	4206(3)	5863.9(14)	146.8(15)
F2	673(2)	3752(3)	6049.9(12)	108.5(10)

Supplementary Table 7. Selective data of bond lengths [Å] for compound **41**

Bonds	Bond lengths/[Å]	Bonds	Bond lengths/[Å]
O1-B1	1.365(3)	C2-C1	1.540(3)
O2-B1	1.362(3)	C2-C3	1.530(3)
O3-B2	1.366(3)	C4-C3	1.516(3)
O4-B2	1.367(3)	C3-H3A	0.9700
C1-B1	1.566(3)	C3-H3B	0.9700
C1-B2	1.571(3)	C4-H4A	0.9700
C1-H1	0.9800	C4-H4B	0.9700
C2-H2B	0.9700	C2-H2A	0.9700
C5-C10	1.521(3)	C5-C4	1.530(3)
C5-C6	1.522(4)	C5-H5	0.9800
F2-C21	1.316(4)	C7-H7A	0.9700
C20-F1	1.313(4)	C7-H7B	0.9700
C13-H13A	0.9700	C12-H12A	0.9700
C13-H13B	0.9700	C12-H12B	0.9700
C11-C12	1.515(4)	C11-C16	1.527(3)
C11-C8	1.546(3)	C11-H11	0.9800
C38-H38A	0.9700	C38-H38B	0.9700
C9-C8	1.526(3)	C9-H9A	0.9700
C9-H9B	0.9700	C8-C7	1.525(4)
C8-H8	0.9800	C3-H3A	0.9700
C3-H3B	0.9700	C56-C55	1.398(4)
C10-C9	1.533(3)	C10-H10A	0.9700
C10-H10B	0.9700	C14-C15	1.513(4)
C14-C13	1.525(4)	C14-H14	0.9800

Supplementary Table 8. Selective data of bond angles [°] for compound **41**

	Angles/°		Angles/°
B2-O3-C30	107.19(18)	B2-O4-C29	107.31(18)
B3-O5-C58	107.23(19)	B3-O6-C57	108.11(19)
B1-O2-C23	107.36(19)	B1-O1-C24	107.28(19)
C18-C17-C14	119.9(2)	C18-C17- C22	117.5(2)
C22-C17-C14	122.6(2)	C15-C14-C17	115.4(2)
B3-C35-H35	108.3	B4-C35-H35	108.3
C3-C2-C1	115.16(19)	C3-C2-H2A	108.5
C1-C2-H2A	108.5	C3-C2-H2B	108.5
C1-C2-H2B	108.5	H2A-C2-H2B	107.5
C15-C14-C13	108.6(2)	C17-C14-C13	110.8(2)
C15-C14-H14	107.2	C17-C14-H14	107.2
C13-C14-H14	107.2	C2-C1-B1	111.20(18)
C2-C1-B2	112.81(19)	B1-C1-B2	105.96(18)
C2-C1-H1	108.9	B1-C1-H1	108.9
B2-C1-H1	108.9	C10-C5-C6	109.3(2)
C10-C5-C4	113.9(2)	C6-C5-C4	108.7(2)
C10-C5-H5	108.3	C6-C5-H5	108.3
C4-C5-H5	108.3	C5-C10-C9	112.6(2)
C5-C10-H10A	109.1	C9-C10-H10A	109.1
C9-C10-H10B	109.1	C5-C10-H10B	109.1
C8-C9-C10	112.7(2)	C8-C9-H9A	109.0
C10-C9-H9A	109.0	C8-C9-H9B	109.0
C10-C9-H9B	109.0	H9A-C9-H9B	107.8
H10A-C10-H10B	107.8	C3-C4-C5	118.1(2)
C3-C4-H4A	107.8	C5-C4-H4A	107.8
C3-C4-H4B	107.8	C5-C4-H4B	107.8
H4A-C4-H4B	107.1	O3-C30-C31	109.1(2)
O3-C30-C32	106.3(2)	C31-C30-C32	110.4(2)

O3-C30-C29	102.22(17)	C31-C30-C29	115.0(2)
C32-C30-C29	113.1(2)	C12-C11-C16	109.4(2)
C12-C11-C8	112.5(2)	C16-C11-C8	111.8(2)
C12-C11-H11	107.7	C16-C11-H11	107.7
C8-C11-H11	107.7	C7-C8-C9	108.5(2)
C7-C8-C11	113.4(2)	C9-C8-C11	112.68(19)
C7-C8-H8	107.3	C9-C8-H8	107.3
C11-C8-H8	107.3	C4-C3-C2	110.52(19)
C4-C3-H3A	109.5	C2-C3-H3A	109.5
C4-C3-H3B	109.5	C2-C3-H3B	109.5

Supplementary Table 9. Selective data of torsion angles [^{o}] for compound **41**

B2-O4-C29-C33	-98.3(2)	B2-O4-C29-C34	142.7(2)
C6-C5-C4-C3	-175.6(2)	C10-C5-C4-C3	62.3(3)
C4-C5-C10-C9	174.8(2)	C6-C5-C10-C9	53.0(3)
C35-C36-C37-C38	175.7(2)	C41-C42-C43-C44	56.3(3)
B2-O4-C29-C30	21.3(2)	C50-C45-C46-C47	-56.2(3)
C42-C45-C46-C47	175.74(19)	C48-C47-C46-C45	56.9(3)
C18-C17-C14-C15	143.8(3)	C22-C17-C14-C15	-38.2(4)
C18-C17-C14-C13	-92.2(3)	C22-C17-C14-C13	85.8(3)
B4-C35-C36-C37	-172.3(2)	B3-C35-C36-C37	66.5(3)
C46-C45-C42-C41	69.3(3)	C5-C45 C42 C41	-55.7(3)
C49-C48-C51-C52	137.7(2)	C47-C48-C51-C52	-99.1(3)
C49-C48-C51- C56	-44.0(3)	C47-C48-C51-C56	79.2(3)
C46-C45-C42-C43	-164.87(19)	C50-C45-C42-C43	70.1(3)
C3-C2-C1-B1	-60.4(3)	C3-C2-C1-B2	-179.25(19)
C41-C42-C43-C44	-52.9(3)	C45-C42-C43-C44	179.13(19)
C46-C47-C48-C51	179.2(2)	C46-C47-C48-C49	-55.1(3)
C38-C39-C44-C43	176.7(2)	C42-C43-C44-C39	56.3(3)
C40-C39-C44-C43	-57.2(3)	B1-O1-C24-C23	-24.3(3)
B3-O5-C58-C57	-21.6(3)	B3-O5-C58-C59	96.7(3)

B3-O5-C58-C60	-145.5(3)	C14-C17-C22-C21	-178.8(3)
C18-C17-C22-C21	-0.7(4)	B3-O6-C57-C58	-21.3(3)
B3-O6-C57-C62	96.4(3)	B3-O6-C57-C61	-145.5(3)
C47-C48-C49-C50	55.7(3)	C51-C48-C49-C50	-179.9(2)
C45-C50-C49-C48	-58.2(3)	C27-C24-C23-C26	160.2(3)
C28-C24-C23-C26	31.4(3)	O1-C24-C23-C26	-85.6(3)
C27-C24-C23-C25	32.1(3)	C28-C24-C23-C25	-96.8(3)
B3-O5-C58-C57	-21.6(3)	B3-O5-C58-C59	96.7(3)
B3-O5-C58-C60	-145.5(3)	C14-C17-C22-C21	-178.8(3)
B1-O1-C24-C28	-145.3(2)	B1-O1-C24-C27	94.2(3)
O1-C24-C23-C25	146.2(2)	C27-C24-C23-O2	-85.7(3)
C28-C24-C23-O2	145.4(2)	O1-C24-C23-O2	28.5(2)
B1-O2-C23-C26	96.0(3)	B1-O2-C23-C24	-23.5(3)
B1-O2-C23-C25	-146.3(2)	C39-C40-C41-C42	-56.5(3)
C45-C42-C41-C40	179.8(2)	C43-C42-C41-C40	53.1(3)
C14-C17-C18-C19	177.7(3)	C22-C17-C18-C19	-0.4(4)
B4-C35-B3-O5	86.5(3)	C10-C5-C6-C7	-53.3(3)
C5-C6-C7-C8	56.1(4)	C11-C8-C7-C6	179.2(3)
C9-C8-C7-C6	-54.8(3)	C17-C22-C21-C20	1.0(5)
C17-C22-C21-F2	-175.8(3)	C19-C20-C21-C22	-0.1(5)
F1-C20-C21-C22	177.2(4)	C19-C20-C21-F2	177.0(3)
F1-C20-C21-F2	-5.7(5)	C17-C14-C13-C12	176.1(3)
C15-C14-C13-C12	-56.1(4)	C18-C19-C20-C21	-1.1(5)
C18-C19-C20-F1	-178.1(4)	C17-C18-C19-C20	1.4(5)
C2-C1-B2-O4	-169.4(2)	B1-C1-B2-O4	68.7(3)
B1-C1-B2-O3	-106.6(2)	C2-C1-B2-O3	15.3(3)
C29-O4-B2-C1	176.8(2)	C29-O4-B2-O3	-7.4(3)
C30-O3-B2-C1	164.6(2)	C30-O3-B2-O4	-11.2(3)
C4-C5-C6-C7	-178.1(2)	C23-O2-B1-O1	9.1(3)
C2-C1-B1-O2	118.8(2)	C24-O1-B1-C1	-168.8(2)

C24-O1-B1-O2	10.6(3)	C23-O2-B1-C1	-171.5(2)
B2-C1-B1-O2	-118.3(2)	C2-C1-B1-O1	-61.8(3)
C16-C11-C12-C13	-52.6(4)	B2-C1-B1-O1	61.1(3)

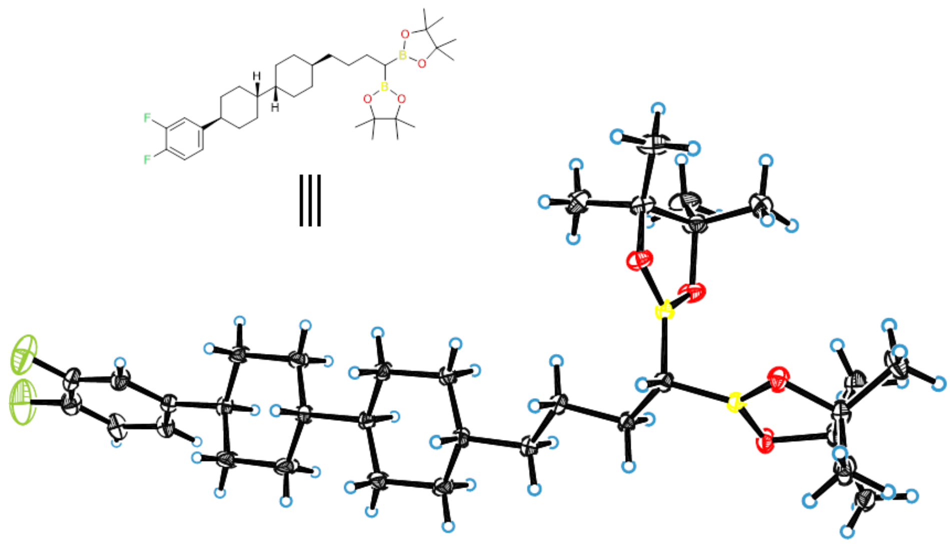

Supplementary Figure 63. Crystal structure of compound 41
